# High Glycemia and Soluble Epoxide Hydrolase in Females: Differential Multiomics in Murine Brain Microvasculature

**DOI:** 10.3390/ijms232113044

**Published:** 2022-10-27

**Authors:** Saivageethi Nuthikattu, Dragan Milenkovic, Jennifer E. Norman, John Rutledge, Amparo Villablanca

**Affiliations:** 1 Division of Cardiovascular Medicine, University of California, Davis, CA 95616, USA; 2 Department of Nutrition, University of California, Davis, CA 95616, USA

**Keywords:** female sex, dementia, high glycemic diet, soluble epoxide hydrolase inhibitor, EETs, multi-omics, microvascular, hippocampus

## Abstract

The effect of a high glycemic diet (HGD) on brain microvasculature is a crucial, yet understudied research topic, especially in females. This study aimed to determine the transcriptomic changes in female brain hippocampal microvasculature induced by a HGD and characterize the response to a soluble epoxide hydrolase inhibitor (sEHI) as a mechanism for increased epoxyeicosatrienoic acids (EETs) levels shown to be protective in prior models of brain injury. We fed mice a HGD or a low glycemic diet (LGD), with/without the sEHI (t-AUCB), for 12 weeks. Using microarray, we assessed differentially expressed protein-coding and noncoding genes, functional pathways, and transcription factors from laser-captured hippocampal microvessels. We demonstrated for the first time in females that the HGD had an opposite gene expression profile compared to the LGD and differentially expressed 506 genes, primarily downregulated, with functions related to cell signaling, cell adhesion, cellular metabolism, and neurodegenerative diseases. The sEHI modified the transcriptome of female mice consuming the LGD more than the HGD by modulating genes involved in metabolic pathways that synthesize neuroprotective EETs and associated with a higher EETs/dihydroxyeicosatrienoic acids (DHETs) ratio. Our findings have implications for sEHIs as promising therapeutic targets for the microvascular dysfunction that accompanies vascular dementia.

## 1. Introduction

Cardiovascular diseases (CVD) are a group of largely preventable diseases and the leading causes of death worldwide [1]. It is increasingly recognized that there is an important contribution of diet to the health of the vasculature in not just CVD, but also in the development of dementia [2]. Cardiovascular and brain health represent a lifetime of exposures and influences, and although affecting different vascular beds, the same vascular risk factors that predispose to CVD (e.g., obesity, dyslipidemia, diabetes, and hypertension) are being increasingly recognized to also compromise cerebrovascular health [3]. One such important modifiable risk factor is diet [1,4]. Our work is at the intersection of heart and brain health, and specifically diet as a modifiable risk factor for both.

The integrity of the endothelium is critical to cardiovascular disease prevention [5,6]. Impaired vascular function is characterized by a number of processes including abnormal endothelial cell permeability [7], vascular adhesion [8], inflammation [9], apoptosis [10,11], and angiogenesis [12]. Disruption of the endothelium is therefore of critical importance to the health of the cardiac and brain vasculature. In the brain, the microvasculature and blood–brain barrier function are particularly central to the development of dementia [13,14,15,16,17] and ischemia [16]. Diet has been shown to contribute to vascular health in the heart and brain including by changes in blood–brain barrier function and inflammation [18]. Downstream effects from diet, such as insulin, have also been shown to lead to inflammatory and neurodegenerative effects. For example, insulin signaling both in the periphery and the brain have been shown to activate pathways (such as PI3K and MAPK) which play a role in inflammatory reaction, insulin resistance, and neurodegeneration [19].

In addition, and as an additional layer of complexity, there are marked sex disparities in the prevalence and risk of CVD and dementia, yet the basis for these differences is not fully understood [20,21,22,23]. Although research in females is less abundant than it is in males, and more research in women needs to be done, it has been shown that diet is a modifiable risk factor for the development of dementia in both sexes [24,25]. Using multiomic approaches and several murine models of lipid stress, including ApoE and LDL-R -/- mice, our prior work has focused on hyperlipidemia in brain microvascular health and demonstrated that the Western diet and dietary lipid stress induce changes in gene expression relating to important vascular functions, including cell adhesion, permeability, angiogenesis, and apoptosis [26,27,28,29]. However, the Western diet contains both elevated fat and glycemic content making it difficult to discern the individual contribution of these dietary components that is necessary to better understand their specific effects. Unfortunately, there is a dearth of literature on high glycemic diets (HGD) in the absence of high fat content in the diet. 

A HGD increases the levels of blood sugar and is associated with a higher risk of type 2 diabetes [30], cardiovascular diseases [30,31], and stroke [32]. We recently demonstrated in male mice that the HGD induces a detrimental transcriptomic response in the microvessels of the hippocampus [33], the region central to the formation of memory [34]. Our studies demonstrated changes in the transcriptome consistent with microvascular dysfunction, oxidation, inflammation, and alterations in mitochondrial function. In an in vitro study of brain microvascular endothelial cells, others have demonstrated that high glucose exposure induces the expression of inflammatory proteins [35]. 

A few studies have investigated the effects of the HGD on the brain and cognitive function showing that a HGD can contribute to vascular dysfunction and reduced cognitive function [36,37,38,39,40,41,42,43]. We have shown deleterious effects of the HGD diet on the male brain microvasculature as a consequence of differential gene expression that is associated with hyperpermeability, apoptosis, and neurovascular inflammation [33]. Yet, only a few studies have addressed the response of the brain of females to a HGD [44,45]. This is an important area of study, as it is known that there are sex differences in cerebrovascular function and its contribution to cognitive impairment and dementia [46].

Soluble epoxide hydrolase (sEH) regulates the activity of a major group of bioactive lipids, the epoxygenated fatty acids such as eposyeicosatrienoic acids (EETs), by degrading these to their less active and sometimes pro-inflammatory corresponding diols [47]. EETs are produced by actions of several P450 isoenzymes which are also a branch of the arachidonic acid cascade [47]. The two most important effects of EETs in the CNS relate to attenuation of inflammatory responses and regulation of vascular tone [48]. In contrast, to products of the cyclooxygenase and lipoxygenase branches of the arachidonic acid cascade, P450-derived EETs, have broad anti-inflammatory actions [48]. Furthermore, inhibition of sEH contributes to stabilization of EETs [49]. Thus, sEH inhibitors have been proposed as a candidate therapeutic target for inflammatory conditions and CNS disorders [48,49]. Based on the effects of sEH inhibitors on suppressing inflammation, we hypothesized that inhibition of sEH would be neuroprotective against the HGD, and that this would be revealed by a multiomic analysis that comprehensively assessed the molecular footprint of the HGD with and without a sEH inhibitor on the brain microvasculature in an important brain memory center, the hippocampus. 

We previously reported that treatment with a sEHI reversed many of the transcriptomic changes induced by a HGD in the hippocampal microvessels of male mice [33]. sEH enzyme converts the vasodilatory and neuro-protective epoxyeicosatrienoic acids (EETs) to dihydroxyeicosatrienoic acids (DHETs) which are biologically less active [47,48]. However, given that there are sex differences in the sEH pathway [50], similar responses as those noted in males might not necessarily be predicted for females. For example, cultured cerebral endothelial cells from female mice have lower levels of sEH expression and higher levels of EETs than endothelial cells from male mice [51]. Furthermore, estrogen suppresses cerebral sEH expression [52]. Others have demonstrated that in mice with experimentally induced cerebral ischemia, females had smaller infarcts and higher blood flow in comparison to males and these sex differences were absent in sEH knockout mice [53]. However, we are unaware of studies examining the effects of inhibiting sEH on the response to a HGD in females. 

To our knowledge, there are also no published studies examining the effects of a HGD and sEHI on hippocampal microvascular multiomics in females. Our previous findings that the HGD induces detrimental changes in gene expression in brain memory centers in males, which can be ameliorated by inhibition of sEH, raises the possibility that these same phenomena could also happen in females. Thus, in the present study we aimed to characterize the effect of a HGD on female mice hippocampal microvascular transcriptomics, and to assess the effects and mechanisms of inhibiting sEH on the multiomics response of female mice. We hypothesized that, in females, a HGD would result in detrimental changes in gene expression when compared to a low glycemic diet (LGD). Further, we hypothesized that inhibition of sEH in the context of high and low glycemic diets would produce a transcriptomic response that would modulate, and perhaps mitigate, the effects of the HGD. This work could help reveal the molecular mechanisms by which vascular risk factors, specifically high glycemia, contribute to gene changes in brain microvessels in females that may provide critical targets for sex specific clinical therapeutics.

## 2. Results

The present study is one in a series of studies investigating the effect of diets on the brain microvasculature in brain memory centers and potential correlates to vascular dementia. We have designed our studies to provide a chronic model of dietary stress of hyperlipidemia, high glycemia (as in the present study), and other dietary stresses. These studies are also focused on understanding the sex differences in the brain microvascular multigenomic response. Accordingly, the study model used for the present studies is consistent with the one we have previously developed and well characterized [26,27,28,29,33].

**Figure 1:**
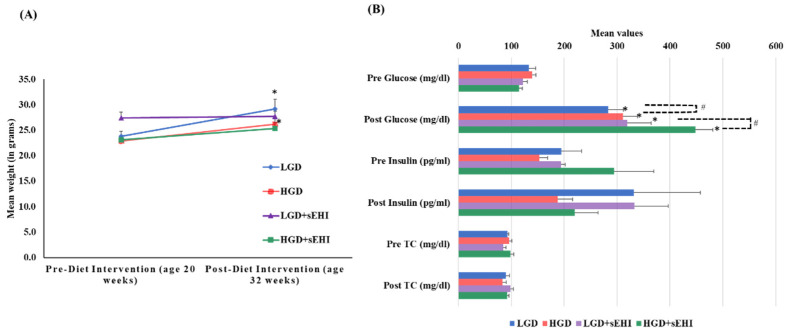
Mean body weight, plasma glucose, insulin, and total cholesterol levels of female mice pre- and post-feeding with the low glycemic diet (LGD) and the high glycemic diet (HGD) with or without soluble epoxide hydrolase inhibitor (sEHI). (**A**) Line graph shows mean weight (grams) of female mice before feeding (at age 20 weeks) and after feeding (at age 32 weeks) with the low glycemic diet (LGD, blue line) and the high glycemic diet (HGD, red line), LGD with soluble epoxide hydrolase inhibitor (LGD+sEHI, purple line), and HGD with sEHI (green line). Error bars are denoted as standard error of the mean (SEM). Weight increased for mice fed the LGD and the HGD post diet intervention when compared to pre-diet intervention (* *p* < 0.05, using unpaired students *t*-test). Two-way ANOVA analysis showed no significant effect of the diet or inhibitor on the body weight in all four groups (LGD, HGD, LGD+sEHI, and HGD+sEHI) post diet intervention. (**B**) Bar graph illustrates the mean values of plasma glucose (mg/dl), insulin (pg/mL), and total cholesterol (mg/dl) of fasted female mice pre and post diet intervention in the following four diet/inhibitor groups: low glycemic diet (LGD, blue), high glycemic diet (HGD, red), LGD with soluble epoxide hydrolase inhibitor (LGD+sEHI, purple), and HGD+sEHI (green). Error bars are denoted as standard error of the mean (SEM). Glucose levels increased at the end of feeding period when compared to baseline in all the groups (* *p* < 0.05, using unpaired students *t*-test). Two-way ANOVA analysis showed statistically significant (# *p* < 0.05) effect of the diet and inhibitor on glucose levels post dietary intervention. At the end of feeding, no statistically significant effect of the diet or inhibitor was observed for the total cholesterol and insulin levels.

At 20 weeks of age (baseline) female mice fed with chow diet on average weighed 24 ± 1.0 g. As expected, after 12 weeks of dietary treatment, the mean weight of mice increased significantly (*p* < 0.05) with both the LGD (29 ± 1.9 g) and the HGD (26 ± 1.3 g). Post diet intervention, there were no statistically significant differences (two-way ANOVA) in body weight between the two diets or the diets with and without the sEHI, Figure 1A. 

Total cholesterol (TC) levels did not statistically differ at baseline between the LGD (92.9 ± 1.8 mg/dL) and the HGD (96.2 ± 4.8 mg/dL). TC levels also did not statistically differ post diet intervention between the LGD (89.7 ± 6.5 mg/dL) and the HGD (83.2 ± 7.8 mg/dL). The sEHI had no significant effect on total cholesterol levels (Figure 1B). 

At the end of feeding period, glucose levels increased significantly (*p* < 0.05) in both the diet groups (LGD 282.9 ± 29.1 mg/dL and HGD 311 ± 27.2 mg/dL) compared to baseline (LGD 133.6 ± 12.2 mg/dL and HGD 134.9 ± 6.6 mg/dL). We also observed statistically significant increases in glucose levels in the HGD compared to the LGD post diet intervention using two-way ANOVA (*p* < 0.05). Similarly, glucose levels also increased significantly (*p* < 0.05) in both the diet groups with inhibitor (LGD+sEHI 319.4 ± 44.6 mg/dL and HGD+sEHI 448.6 ± 32.3 mg/dL) and were significantly higher (*p* < 0.05) in the HGD+sEHI compared to the HGD (Figure 1B). Insulin levels did not statistically differ in the LGD and the HGD groups with or without sEHI (Figure 1B). 

There was no significant difference in estrous cycle phase for mice in either dietary or inhibitor study group.

### 2.1. Effect of the High Glycemic Diet on the Hippocampal Microvascular Genome

#### 2.1.1. Global Gene Expression and Hierarchical Clustering

**Figure 2:**
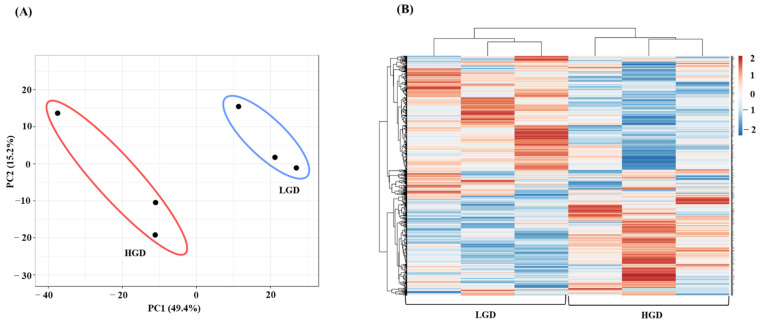
Principal Component Analysis (PCA) and hierarchical clustering of genes expressed in hippocampal microvasculature of female mice fed with the low glycemic diet (LGD) and the high glycemic diet (HGD). (**A**) The PCA plot demonstrates the hippocampal microvascular global gene expression profile trends in the low glycemic diet (LGD) and the high glycemic diet (HGD) shown as blue and red ovals, respectively. PCA plot depicts the dataset variance as principal components (PC), and the x and y axes represent the most significant differences. The *x* axis indicates the percent total variation by PC1, and *y*-axis indicates the percent total variation by PC2. (**B**) Hierarchical clustering shows opposite global gene expression profiles in the HGD compared to the LGD. Genes with higher levels of expression are in red and genes with lower levels of expression are in blue.

To study the molecular effect of the HGD on the hippocampal microvasculature in female mice, we first evaluated global gene expression using a genetic distance visualization technique termed principal component analysis (PCA). PCA displays similarities among the populations. PCA plot demonstrated that the global gene expression profiles of mice on the LGD and HGD were clearly distinct from each other (Figure 2A).

Next, we performed hierarchical clustering of global gene expression profiles, which organizes the clusters into a hierarchy by grouping similar data points together. This analysis further confirmed that in general, genes with lower levels of expression with the LGD had higher levels of expression with the HGD, and vice versa (Figure 2B). Hence, female mice fed with the HGD and the LGD, had global hippocampal microvascular gene expression profiles that were opposite to each other.

#### 2.1.2. Differential Gene Expression

**Figure 3:**
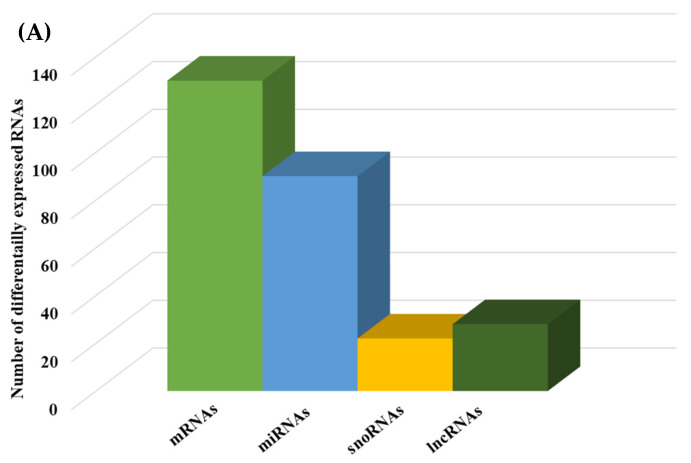

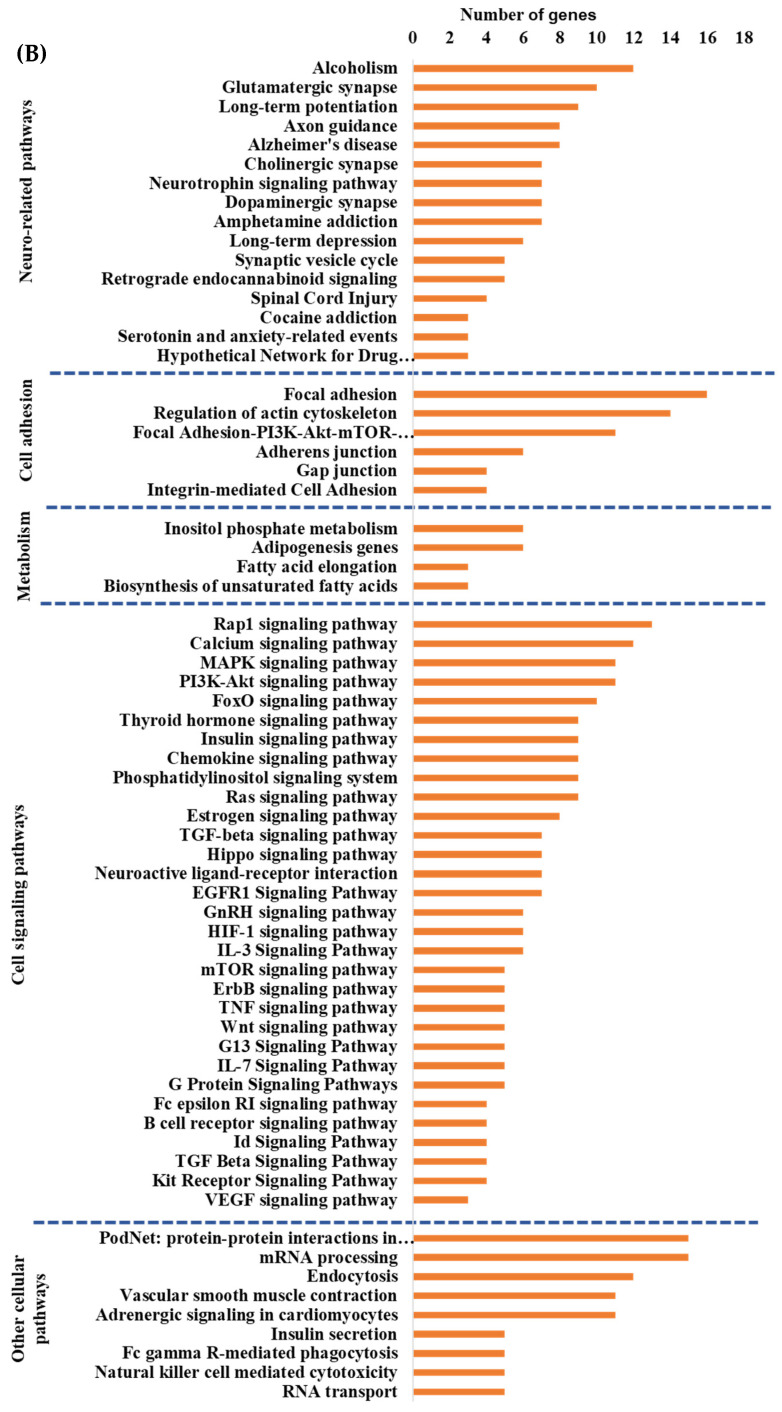
Integrated cellular pathways of differentially expressed mRNAs and targets of miRNAs and lncRNAs in the hippocampal microvessels of the high glycemic diet (HGD) compared to the low glycemic diet (LGD). (**A**) Distribution of differentially expressed protein coding and noncoding RNAs in the female mice hippocampal microvasculature of the high glycemic diet (HGD) compared to the low glycemic diet (LGD). The number of differentially expressed protein coding messenger RNAs (mRNAs, light green), non-protein coding differentially expressed RNAs (microRNAs = miRNAs, light blue; small nucleolar RNA = snoRNAs, yellow; and long noncoding RNA = lncRNAs, dark green) in the HGD vs. LGD. (**B**) A subset of the important pathways regulated by the differentially expressed mRNAs, miRNA targets, and LncRNA targets in the hippocampal microvessels of the HGD when compared to the LGD are shown in the histogram. Genetrail2 database was used to identify significant cellular pathways with *p* value less than 0.05. These pathways were categorized into cell signaling, cell metabolism, cell adhesion, and neuronal functions.

We then compared the HGD to the LGD to investigate the effect of the HGD on differential gene expression. Using statistical analysis of microarray data, we identified 506 differentially expressed genes (DEGs) in hippocampal microvessels following the HGD, with most of the DEGs being downregulated by the HGD (465 genes downregulated vs. 41 genes upregulated) when compared to the LGD. The fold-change ranged between −35.12 to −2 for downregulated DEGs, and from 2.0 to 14.49 for upregulated DEGs, as shown in Supplemental Table S1 To our knowledge, we further demonstrate for the first time that the HGD modulates the expression of nearly equal numbers of protein coding genes (130) and non-coding genes (140) in female murine hippocampal microvessels (Figure 3A), including 90 microRNAs (miRNAs), 22 small nucleolar RNAs (snoRNAs), and 28 long non-coding RNAs (lncRNAs). The remaining 96 DEGs were other genes such as transfer RNA (tRNA), miscellaneous RNAs (miscRNA), noncoding transcripts with and without gene symbols, and pseudogenes.

#### 2.1.3. Pathways and Networks for Coding and Non-Coding Differentially Expressed Genes

To identify the cellular pathways involving differentially expressed protein coding genes we performed Genetrial2 database analysis. Among the 60 cellular pathways we identified were those involved in the regulation of cell signaling (e.g., insulin signaling), cell adhesion (e.g., focal adhesion), cellular metabolism (e.g., adipogenesis), neurodegenerative diseases (e.g., Alzheimer’s disease), and cellular stress (e.g., oxidative stress and redox pathway), Supplemental Figure S1. We then performed Enrichr database analysis of DEGs to identify potential transcription factors (TFs) and their target genes whose activity could be modulated by the HGD with results shown in Supplemental Figure S2. The most statistically significant transcription factors were PITX2 (Paired-like homeodomain transcription factor 2), TCF12 (Transcription factor 12), ZBTB7B (Zinc finger and BTB domain-containing protein 7B), ATF6 (Activating Transcription Factor 6), and RUNX1 (Runt-related transcription factor 1).

As previously mentioned, we identified that the HGD also modulates differentially expressed non-protein coding RNAs; predominantly miRNAs, lncRNAs and snoRNAs. The majority of the 90 DE miRNAs were downregulated (fold change −32.73 to −2.01), with only 1 miRNA upregulated (3.41-fold change) by the HGD compared to the LGD, (Supplemental Table S2). Using MIENTURNET software and database interrogation, we were able to reveal 192 potential target genes for only 30 of the 90 miRNAs and constructed an interaction network between these (Supplemental Figure S3). Although the majority of the genes were targeted by a single miRNA, we found redundancy in that some genes were targeted by 2 or up to 8 distinct miRNAs. For example, Akap2 (A-Kinase Anchoring Protein 2) was targeted by miRNA 466f, miRNA 297c, miRNA 297a, miRNA 466h, miRNA 466j, miRNA 669m, and miRNA 466k. We performed miRNA target gene pathway analyses and identified that these miRNA target genes were involved in apoptosis, cell adhesion and permeability, and signal transduction and neurofunction related pathways, Supplemental Figure S4. Interestingly, seven of the pathways regulated by the miRNA target genes were in common with the pathways regulated by the differentially expressed protein coding mRNAs, including insulin signaling, regulation of actin cytoskeleton, and Alzheimer’s disease. Of the miRNA target specific pathways, most were associated with signal transduction, inflammation, and cell death. On the other hand, pathways unique to differentially expressed protein coding genes were predominantly related to oxidative stress, metabolism, and cell signaling.

In addition to miRNAs, we also found 24 differentially expressed lncRNAs (Supplemental Table S3) that were regulated by the HGD. The majority of them (23) were downregulated (fold-change −4.77 to −2.02), and only 1 lncRNA was upregulated (2.14-fold-change). Using LncRRIsearch and Rtools CBRC databases we were able to identify 249 potential target genes for 3 of the 24 lncRNAs: Gm11008, Gm15758, and Gm27243, (Supplemental Figure S5). Pathway analysis of these target genes showed that they were involved in pathways such as nitric oxide signaling and Alzheimer’s disease (Supplemental Figure S6), showing some commonality with the 6 miRNA targets pathways.

Next, we studied the differential expression of snoRNAs in the HGD compared to LGD. Among the 22 differentially expressed snoRNAs, 5 were upregulated (fold change 2.03 to 14.49) and 17 were downregulated (fold change −15.76 to −2.02), Supplemental Table S4. We were not able to identify any published known target genes for the DE snoRNAs in our literature review.

#### 2.1.4. Integrated Analysis of Differentially Expressed Genes and Key Pathways

To determine whether the hippocampal microvascular differential gene expression pattern of the HGD when compared to the LGD had functional coordination at the molecular level, we followed the individual omic analysis with an integrated pathway analysis of protein coding DEGs and targets of miRNAs and lncRNAs. This analysis revealed four important cellular pathways that were differentially modulated by the HGD including: cell signaling pathways (including MAPK signaling, PI3K-Akt signaling, Rap1 signaling, and VEGF signaling), neurodegenerative diseases (such as Alzheimer’s disease), cell adhesion (including focal adhesion, regulation of actin cytoskeleton, and Adherens junction), and cellular metabolism (such as Adipogenesis and Inositol phosphate metabolism), Figure 3B.

### 2.2. Effect of the on the Hippocampal Microvascular Genome of Mice Fed the Low and High Glycemic Diets

**Figure 4:**
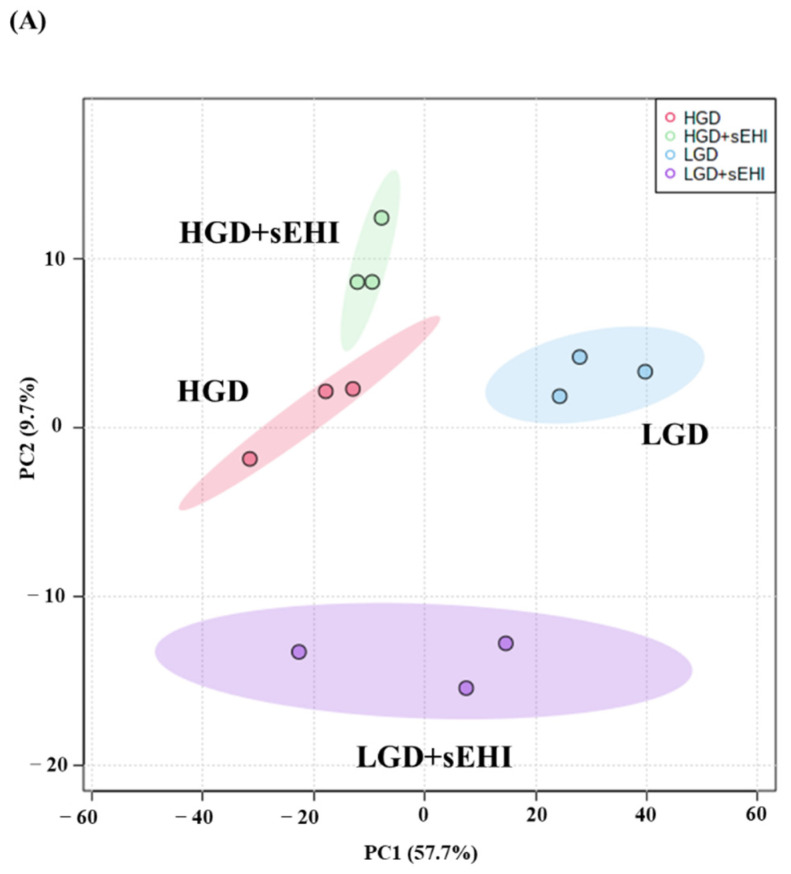

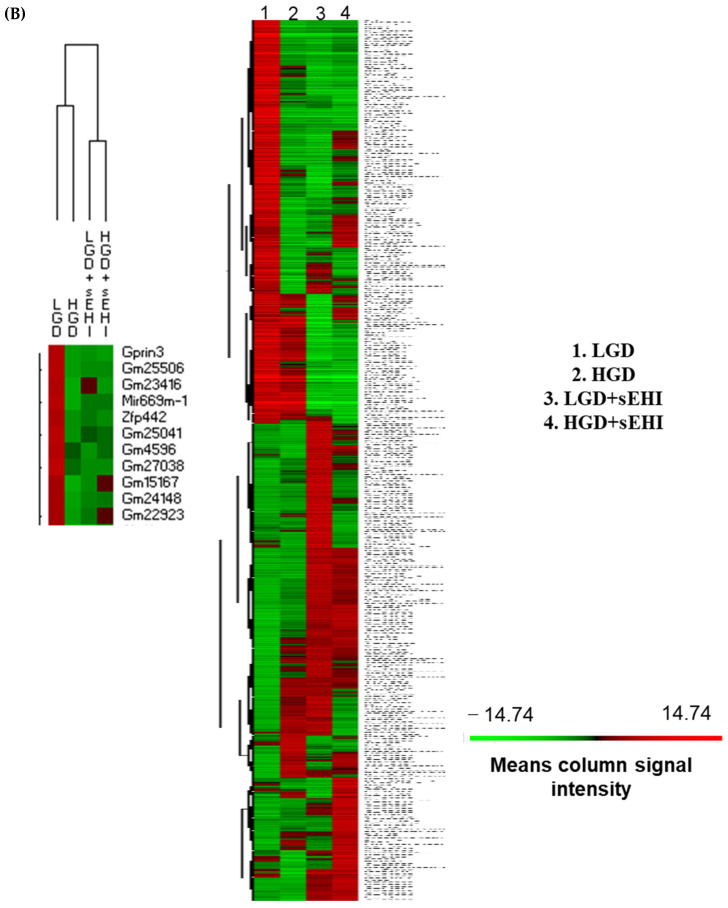

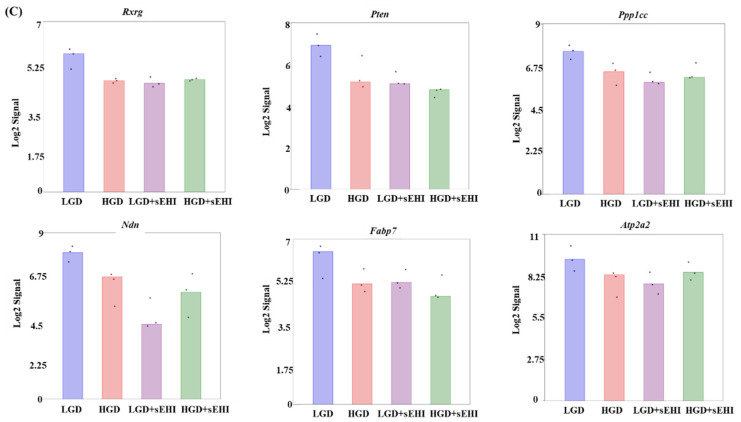
Principal Component Analysis (PCA) and heat map of genes expressed in hippocampal microvessels of female mice fed with the low glycemic index diet (LGD) and the high glycemic index diet (HGD) in the presence or absence of the soluble epoxide hydrolase inhibitor (sEHI). (**A**) PCA plot demonstrates the hippocampal microvascular global gene expression profile trends in the low glycemic diet (LGD) and the high glycemic diet (HGD) shown as blue and red circles, respectively, the LGD+sEHI and the HGD+sEHI, shown as purple and green circles, respectively. PCA plot depicts the dataset variance as principal components (PC), and the x and y axes represent the most significant differences. The *x* axis indicates the percent total variation by PC1, and *y*-axis indicates the percent total variation by PC2. (**B**) Heat map showing average signal intensities of genes in rows, and the four different experimental treatment groups in columns, as follows: column 1: low glycemic diet (LGD); column 2: high glycemic diet (HGD); column 3: LGD with soluble epoxide hydrolase inhibitor (sEHI); column 4: HGD+sEHI. Genes with higher means column signal intensity are shown in red and genes with lower means column signal intensity are shown in green. List of genes in this heat map and their mean signal intensities are provided in Supplemental Table S5. (**C**) Bar graphs show average signal intensities of genes in the LGD (blue), the HGD (red), LGD+sEHI (purple), and the HGD+sEHI (green) groups. Black dots represent log2 signal intensity for each biological replicate, n = 3 mice per group. *Rxrg*, *Pten*, *Ppp1cc*, *Ndn*, *Fabp7*, and *Atp2a2* genes are significantly altered (*p* < 0.05) in the LGD+sEHI vs. LGD.

To assess whether the sEHI could inhibit the seemingly detrimental molecular effects of the HGD on hippocampal microvessels (downregulation of genes involved in pathways that play a key role in endothelial cell function and integrity such as focal adhesion, regulation of actin cytoskeleton, and Rap 1 signaling), we again did PCA analysis of the global gene expression profiles of the diets with the inhibitor (LGD with sEHI and the HGD with sEHI), Figure 4A.

PCA plots showed that the global gene expression profiles of the LGD and the HGD groups with inhibitor were distinctly different when compared to without inhibitor. Further, heat map revealed that the sEHI groups (LGD+sEHI and HGD+sEHI) tended to cluster together (Figure 4B and Supplemental Table S5). Interestingly, the gene expression profile of the LGD+sEHI was nearly completely opposite to that of the LGD alone. For example, the expression of genes including *Rxrg*, *Pten*, *Ppp1cc*, *Ndn*, *Fabp7*, and *Atp2a2* was significantly downregulated in the LGD with inhibitor compared to the LGD alone (Figure 4C). In contrast, the gene expression profile of the HGD+sEHI differed much less from that of the HGD without the inhibitor. Thus, the inhibitor had an opposite profile in the LGD group, but much less so in the HGD.

**Figure 5:**
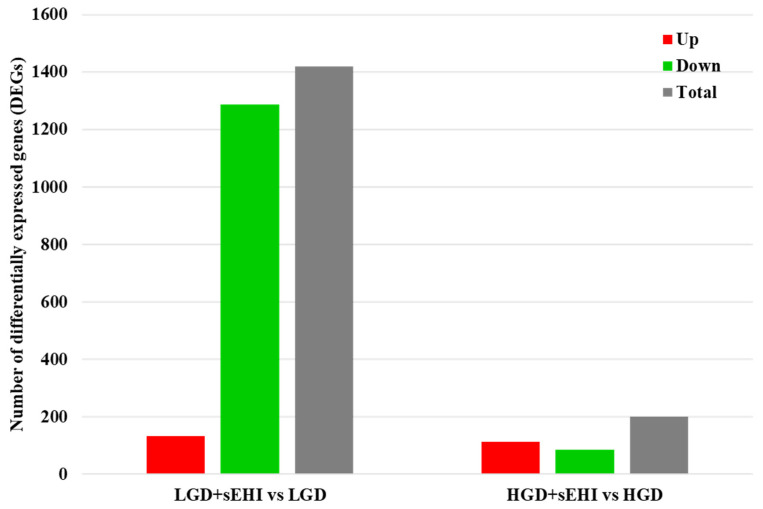
Differentially expressed genes (DEGs) in hippocampal microvessels of the low glycemic index diet (LGD) and the high glycemic index diet (HGD) with and without soluble epoxide hydrolase inhibitor (sEHI). Histogram shows the number of differentially expressed genes (DEGs) in hippocampal microvessels for the low glycemic diet (LGD) with soluble epoxide hydrolase inhibitor (sEHI) compared to the LGD, and the high glycemic diet (HGD) with sEHI compared to the HGD. Upregulated DEGs are shown in red, downregulated DEGs in green, and total DEGs in grey.

Next, we did statistical analysis of the microarray data for the effect of the inhibitor in the LGD and showed that in the presence of the inhibitor, a much larger number of genes were differentially expressed (1420) compared to the LGD without the inhibitor (Figure 5 and Supplemental Table S6), and when compared to the inhibitor in the HGD (Supplemental Table S7), with again most genes downregulated.

**Figure 6:**
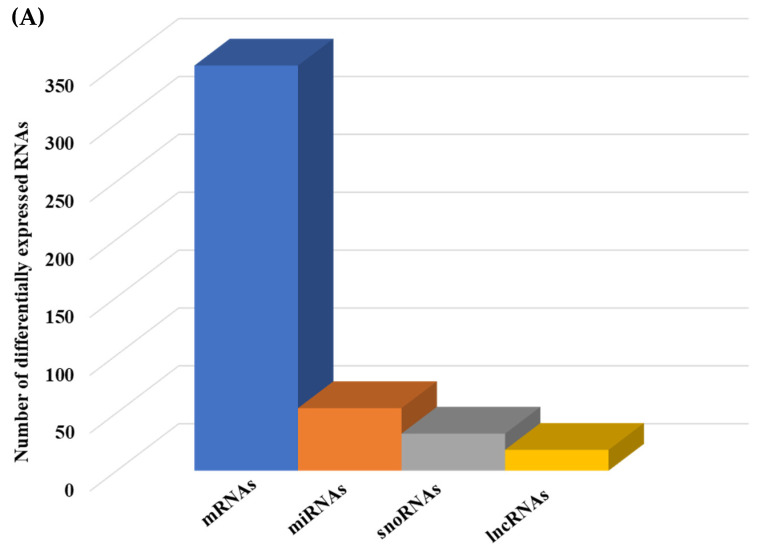

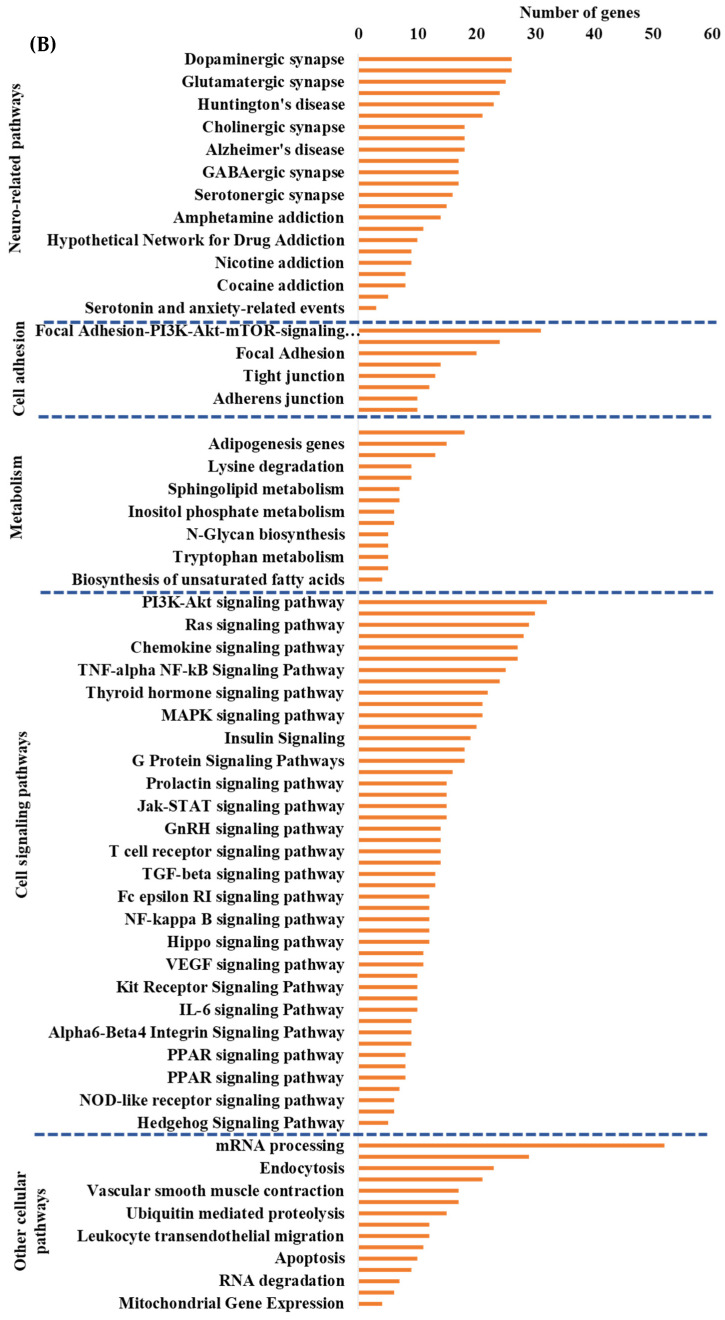
Integrated cellular pathways of differentially expressed mRNAs and targets of miRNAs and lncRNAs in the hippocampal microvessels of the low glycemic diet with soluble epoxide hydrolase inhibitor (LGD+sEHI) compared to the LGD. (**A**) Differentially expressed protein coding and noncoding RNAs in the female mice hippocampal microvasculature of the low glycemic diet in the presence of soluble epoxide hydrolase inhibitor (LGD+sEHI) compared to the LGD. Number of differentially expressed protein coding (messenger RNAs = mRNAs, light green), non-protein coding RNAs (microRNAs = miRNAs, light blue; small nucleolar RNA = snoRNAs, yellow; and long noncoding RNA = lncRNAs, dark green) in the LGD+sEHI vs. LGD. (**B**) A subset of the important pathways regulated by the differentially expressed mRNAs, miRNA targets, and LncRNA targets in the hippocampal microvessels of the LGD+sEHI when compared to the LGD are shown in the histogram. Genetrail2 database was used to identify cellular pathways with statistical significance of *p* < 0.05. These pathways were categorized into cell signaling, cell metabolism, cell adhesion, and neuronal functions.

Since the effect of the sEHI on differential gene expression was much greater in the LGD, we further examined the effect of the inhibitor in the LGD and showed that although there was downregulation of both protein coding and noncoding DEGs, the impact was primarily on protein coding mRNAs (Figure 6A and Supplemental Figure S7). In addition, in the LGD group, the inhibitor regulated a larger number of protein coding genes (365) than it did in the HGD (28).

Bioinformatics analyses of DEGs comparing the LGD+sEHI to the LGD condition alone revealed potential TFs and their targets whose activity could be modulated by the inhibitor (Supplemental Figure S8). We identified 23 statistically significant TFs such as RUNX1 (Runt-related transcription factor 1), NEUROD1 (Neuronal Differentiation 1), ATF6 (Activating Transcription Factor 6), TCF3 (Transcription Factor 3), and ZBTB7B (Zinc finger and BTB domain-containing protein 7B).

In looking at the effect of the LGD+sEHI on non-protein coding genes, we found that the sEHI modulated 54 miRNAs, 32 snoRNAs, and 18 lncRNAs (Figure 6A, Supplemental Tables S8–S10. Most of these DE miRNAs were also downregulated and targeted 899 genes (Supplemental Figure S9) that were involved in cell signaling pathways (such as PI3K-Akt signaling, Ras signaling, and MAPK signaling) (Supplemental Figure S10), and pathways related to endothelial cell function (such as focal adhesion).

The majority of DE lncRNAs were downregulated by the inhibitor in the LGD and targeted 805 genes (Supplemental Figure S11) mainly involved in Alzheimer’s disease and focal adhesion (Supplemental Figure S12).

Next, we performed integrated pathway analysis of differentially expressed protein coding genes and targets of DE miRNAs and lncRNAs from the LGD+sEHI compared to the LGD diet groups. This analysis revealed that in the LGD, the inhibitor regulated neuro-related pathways, cell adhesion, and cellular metabolism pathways. However, the effect of the inhibitor in the LGD condition seemed to be most pronounced in regulating important and major cellular signaling pathways including PI3K-Akt signaling, Ras signaling, and TNF-alpha NF-Kb signaling, and cellular metabolic pathways including adipogenesis and sphingolipid metabolism (Figure 6B).

**Figure 7:**
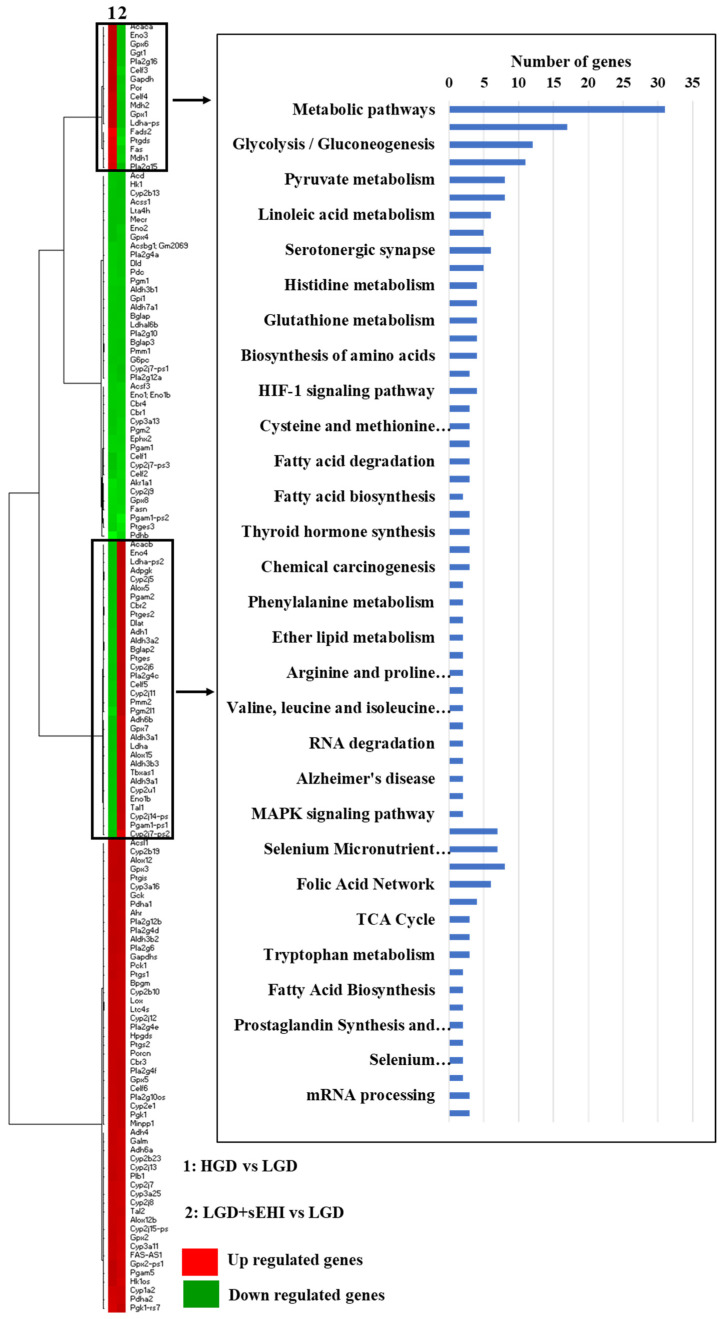
Heat map of genes involved in metabolic pathways upstream of soluble epoxide hydrolase (sEH). Heat map showing fold change of genes in rows, and the different dietary and experimental treatment comparison groups in columns, as follows: column 1: high glycemic diet (HGD) vs. low glycemic diet (LGD); column 2: LGD with soluble epoxide hydrolase inhibitor (sEHI) vs. LGD. Upregulated genes are shown in red and downregulated genes are shown in green. Genes with opposite expression pattern are shown in rectangle boxes and are involved in various cellular metabolic pathways upstream of soluble epoxide hydrolase (sEH) as shown in the histogram. Statistically significant pathways (*p* < 0.05) were identified using Genetrial2 online database.

Lastly, we performed heat map of genes from the HGD vs. LGD and the LGD+sEHI vs. LGD and identified genes with opposite expression patterns in both these comparisons. They were involved in several cellular metabolic pathways upstream of sEH including arachidonic acid metabolism, glycolysis/gluconeogenesis, linoleic acid metabolism and eicosanoid metabolism (Figure 7).

### 2.3. Brain Epoxyeicosatrienoic Acids to Dihydroxyeicosatrienoic Acids Ratio

**Figure 8:**
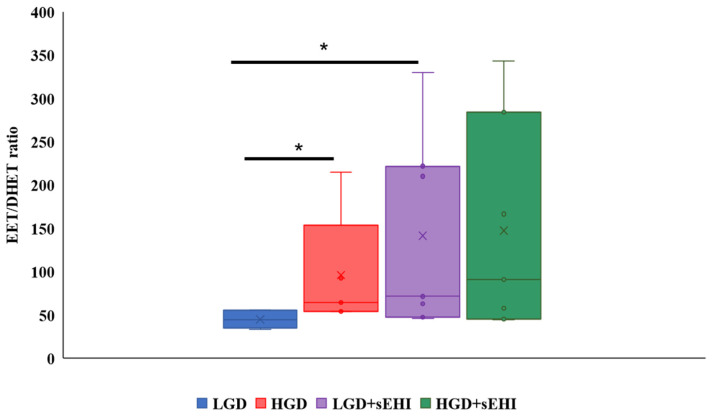
Epoxyeicosatrienoic acid to dihydroxyeicosatrienoic acid (EET/DHET) ratios within brain free oxylipins. The ratio of all detected epoxyeicosatrienoic acids (EETs) to all detected dihydroxyeicosatrienoic acids (DHETs) within the pool of brain free oxylipins was determined for each mouse. The EET/DHET ratio was greater in high glycemic diet (HGD) fed mice compared to low glycemic diet (LGD) fed mice. Mice consuming a LGD while receiving the soluble epoxide hydrolase inhibitor (sEHI) had a greater EET/DHET ratio than mice fed the LGD without inhibitor. The EET/DHET ratio in mice consuming a HGD while receiving the sEHI did not differ from mice fed the HGD without inhibitor. The top and bottom of the box indicate the upper and lower quartiles, respectively. The line through the box indicates the median, while the X indicates the mean. Statistical differences were determined by Mann–Whitney U test, * *p* < 0.05.

To confirm that the sEHI was working as expected, and to better understand the reason for the greater impact on the multiomic response to the sEHI in the LGD, we determined the ratio of epoxyeicosatrienoic acids to dihydroxyeicosatrienoic acids (EETs/DHETs) within the brain free oxylipin pool. We found that mice on the HGD had a greater brain EETs/DHETs ratio when compared to the LGD, and that ratio was increased further and significantly (*p* < 0.05) in the presence of the sEHI for mice on the LGD diet (Figure 8). However, there was no significant effect of the sEHI on the EETs/DHETs ratio in brains of mice on the HGD (Figure 8).

**Figure 9:**
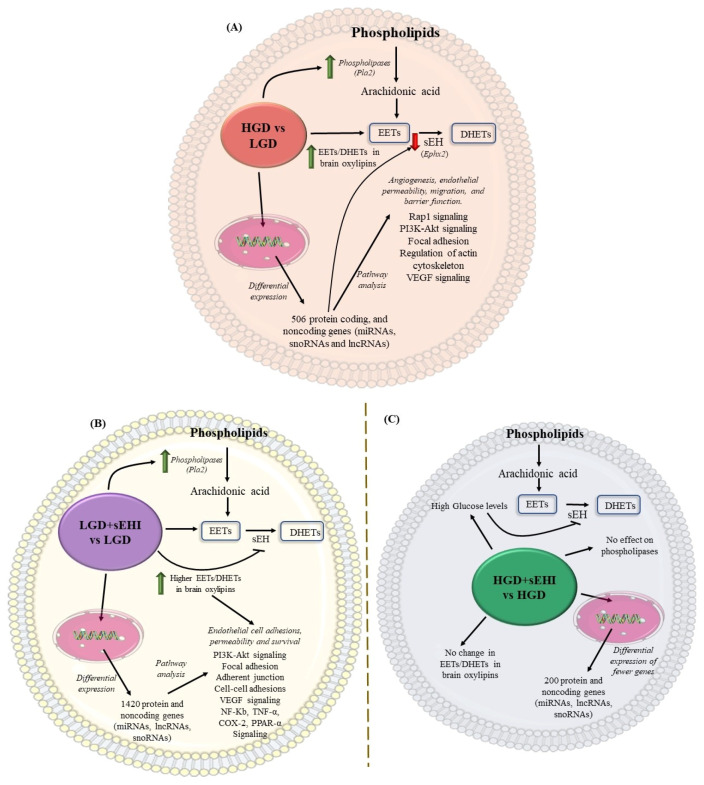
Conceptual schematic summary of the differential effect of the high glycemic diet (HGD) vs. the low glycemic diet (LGD), and of the soluble epoxide hydrolase inhibitor (sEHI) on the HGD as compared to the LGD. (**A**) The schematic summarizes the effect of the high glycemic diet (HGD) on modulating the expression of protein coding and protein noncoding genes (microRNAs, long noncoding RNAs and small nucleolar RNAs) involved in cellular pathways that could impair angiogenesis, endothelial migration, permeability and barrier function in female hippocampal microvessels. The HGD had increase in Epoxyeicosatrienoic acids (EETs) to dihydroxyeicosatrienoic acids (DHETs) ratio in brain oxylipins when compared to the LGD. (**B**) The schematic summarizes the effect of the soluble epoxide hydrolase inhibitor (sEHI) on the LGD when compared to without inhibitor in female hippocampal microvessels. The sEHI on the LGD modulated greater number of differentially expressed protein and noncoding genes (miRNAs, lncRNAs, and snoRNAs) involved in cellular pathways that could promote endothelial cell adhesion, permeability, and survival in female hippocampal microvessels. The sEHI had higher EETs/DHETs ratios on the LGD. (**C**) The schematic summarizes the effect of the soluble epoxide hydrolase inhibitor (sEHI) on the HGD when compared to without inhibitor in female hippocampal microvessels. The sEHI had no effect on the EETs/DHETs ratios on the HGD. Moreover, the sEHI had lesser impact on the differential expression on the HGD and modulated fewer protein and noncoding genes.

To assess whether these EETs/DHETs ratios correlated to differential gene expression in the HGD vs. LGD and the LGD+sEHI vs. LGD comparisons, we performed spearman correlation analyses of the DEGs involved in the significant cellular pathways shown in Figure 9A,B with the EETs/DHETs ratio.

**Figure 10:**
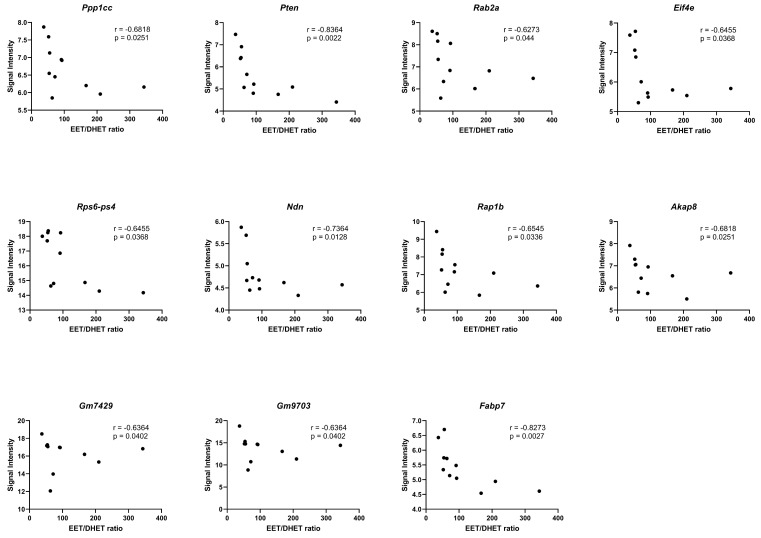
Correlation of the EETs/DHETs ratio to differential gene expression. Spearman correlation (r) analyses of the EETs/DHETs ratio and the signal intensities of the differentially expressed genes (DEGs) in the HGD vs. LGD and the LGD+sEHI vs. LGD using graphpad prism software. DEGs involved in the regulation of actin cytoskeleton (*Ppp1cc*), PI3K-Akt signaling (*Pten*, *Rab2a*, *Eif4e*, and *Rps6-Ps4*), adipogenesis (*Ndn*), focal adhesion (*Rap1b*), TNF-alpha NF-kB Signaling Pathway (*Akap8*, *Gm7429*, and *Gm9703*), and PPAR signaling (*Fabp7*) showed significant negative correlation (*p* < 0.05) with the EETs/DHETs ratio. Black dots represent signal intensity (*y*-axis) and EETs/DHETs ratio (*x*-axis) for each mouse.

We identified significant negative correlations (*p* < 0.05) between the EETs/DHETs ratio and genes involved in the regulation of actin cytoskeleton (*Ppp1cc*), PI3K-Akt signaling (*Pten*, *Rab2a*, *Eif4e*, and *Rps6-Ps4*), adipogenesis (*Ndn*), insulin signaling (*Ppp1cc*, and *Pten*), focal adhesion (*Ppp1cc*, *Pten*, and *Rap1b*), TNF-alpha NF-kB Signaling Pathway (*Akap8*, *Gm7429*, and *Gm9703*), and PPAR signaling (*Fabp7*), Figure 10.

### 2.4. Cognitive Function

We assessed cognitive performance in female mice using the y-maze. The y-maze measures spatial cognition (hippocampal), learning, and memory in mice by quantifying alternating triplets (animal visits to all three arms of the y-maze sequentially). Cognition was evaluated for differences in spontaneous alternation behavior (SAB) in the Y-maze linked to the number of arm entries by mean % alternation triplets (# alternating triplets/total # triplets), Table 1. We did not observe any statistically significant differences in cognitive function as assessed by Y-maze for female mice on the HGD (48.7 ± 2.2% alternation triplets) compared to the LGD (44.3 ± 2.4% alternation triplets). Moreover, there were no statistically significant differences in the performance with the sEH inhibitor on the HGD (57.5 ± 5.5% alternation triplets) compared to the HGD alone. In contrast, the sEH inhibitor on the LGD averaged statistically significant (*p* < 0.05) higher alternation triplets (58 ± 4.2%) in comparison to the LGD alone. Thus, cognitive performance assessed by Y-maze improved with the sEH inhibitor on the LGD condition.

### 2.5. Clinical Association of Genomic Data

Our next step of bioinformatic analysis consisted of identifying associations with known human diseases for the identified differentially expressed genes following different diet/inhibitor treatments. As presented in Table 2, this analysis showed that identified differentially expressed genes in our study were predominantly associated with nervous system diseases, such as neurodegenerative diseases, dementia, or intellectual disability; cardiovascular diseases, including vascular diseases or myocardial ischemia; as well as metabolic diseases. No significant association was observed for genes identified as differentially expressed in HGD+sEHI vs. HGD, probably because of very small number of genes were identified to be differentially expressed in this condition.

## 3. Discussion

We have recently shown using a male murine model of hyperglycemia that the HGD has detrimental effect on the hippocampal microvasculature, which can be mitigated by sEH inhibition [33]. However, no studies to date have explored the effect of the HGD on hippocampal microvessels in female mice. In this present study, we characterized for the first time the effect of a HGD on female murine brain hippocampal microvessels using large-scale transcriptomics, assessed the effect of sEH inhibition on the multiomics response, the extent of change in relevant brain oxylipins and cognition, and association of the multiomic data with clinical disease.

Below, we discuss specific examples of key protein coding and non-protein coding genes, cellular pathways, and transcription factors modulated by the HGD, and also characterize the multiomics action of the sEH inhibition in response to low and high glycemic diet, and in the context of implications for cognitive function and clinical disease.

### 3.1. Effects of the HGD on Female Brain Microvascular Endothelium

In this present study, we characterized for the first time the effect of a HGD on female mice brain hippocampal microvessels using large-scale transcriptomics. In our experimental mice, we did not observe any statistically significant differences in the body weight, total cholesterol, glucose, or insulin levels in the HGD compared to the LGD at the end of dietary feeding, consistent with previously published studies for these experimental models [43,45,54,55,56,57]. Our current study shows for the first time that the HGD had opposite gene expression patterns compared to the LGD, characterized by mostly downregulation of differentially expressed protein coding and non-protein coding genes including miRNAs, snoRNAs and lncRNAs. We also identified transcription factors (TFs) whose activity could be modulated by the HGD. Among them was ATF6 (Activating Transcription Factor 6) which targets protein coding DEG downregulated by the HGD, such as PPP1CB (Protein Phosphatase 1 Catalytic Subunit Beta). ATF6 also worsens endoplasmic reticulum stress induced vascular endothelial cells apoptosis [58] which is implicated in diseases such as diabetes, atherosclerosis and ischemia [59,60,61]. The HGD also downregulated differentially expressed miR-130 that targets PLC (Phospholipase C). Decreased miR-130 expression promotes endothelial cell death and impairs angiogenesis [62]. PLC in turn is associated with vascular inflammation and disruption of barrier [63], all suggesting a deleterious vascular molecular response to stress.

Integrative analysis of differentially expressed protein-coding genes with the targets of differentially expressed non protein coding miRNAs and lncRNAs, revealed that DEGs were primarily involved in 4 important cellular pathways including: cell signaling pathways (such as Rap1 signaling, MAPK signaling, PI3K-Akt signaling, and VEGF signaling), cell adhesion (including focal adhesion and regulation of actin cytoskeleton), cellular metabolism, and neurodegenerative diseases (such as Alzheimer’s disease). Among these, focal adhesion and regulation of actin cytoskeleton and Rap1 signaling were the most over-represented pathways. They are known to be key regulators of permeability and barrier function of microvascular endothelial cells [16,64,65]. PPP1CB, Rac (small GTPase) and MLCP (myosin light chain phosphatase) were additional differentially expressed protein coding genes downregulated by the HGD involved in the 4 integrated pathways. PPP1CB inhibition impairs focal adhesion, cell migration [66], and barrier function in endothelial cells [67]. Furthermore, inhibition of RAC and MLCP is known to increase endothelial cells permeability and disrupt barrier integrity [68]. Thus, our findings suggest that a HGD has deleterious effects on the brain microvasculature specifically by promoting a molecular cascade associated with hyperpermeability, apoptosis, and neurovascular inflammation, and also by impairing endothelial cell barrier, migration, and angiogenesis, that proceed via downregulation of pathway-associated TFs and protein coding and noncoding genes. Interestingly, these genomic effects of the HGD in female mice are independent of changes in glucose, insulin, lipids, or estrous status.

### 3.2. Effects of sEHI on the LGD

Inhibition of sEH has been suggested to exert health protective properties including therapeutic properties against cardiovascular disorders [69,70,71,72,73]. It is believed that the benefits seen with inhibition of the sEH are due to an increase in EETs which have been reported to result in a lowering of blood pressure, improvement in cerebral blood flow, increase in neural activity, and angiogenesis [74]. EETs exert important beneficial actions like vasodilation, anti-inflammation, and anti-platelet aggregation that could combat vascular-related diseases via these mechanisms [47,75,76,77]. Several studies have also reported that inhibitors of sEH can modulate the expression of specific genes. For example, administration of the sEHi (1-trifluoromethoxyphenyl-3-(1-propionylpiperidin-4-yl) urea (TPPU)) resulted in elevated mRNA levels of Cpt1a, Acox1, and Mcad by activating PPAR-α [78]. In addition, the sEH inhibitor GSK1910364A has been reported to downregulate expression of 8 proinflammatory marker genes, suggesting both cytoprotective and anti-inflammatory effects mediated by sEHI treatment [79]. To the best of our knowledge, ourstudy using trans-4-[4-(3-adamantan-1-yl-ureido)-cyclohexyloxy]-benzoic acid (t-AUCB), is the first to assess and report the global genomic impact of sEH inhibition in female murine brain hippocampal microvascular endothelium. Our results suggest that inhibition of sEH in vivo can modulate large numbers of genes in the brain microvasculature, including not only protein coding RNAs but also protein non-coding genes, such as miRNAs, snoRNAs, lncRNAs, and other RNAs.

In addition, our work shows that the sEHI regulated expression of genes that can regulate various cellular processes in the brain microvasculature. Our integrative bioinformatic analysis of protein-coding DEGs, miRNA targets, and lncRNA targets showed cell signaling, particularly PI3K-Akt signaling, was the most statistically significant pathway. The PI3K/Akt signaling pathway promotes angiogenesis, growth, cell survival, proliferation, and metabolism in response to extracellular signals. It can also regulate other cell signaling processes via PIP3 and Akt, such as Nf-kB, mTOR, eNOS, and also Ras1. In concert, these signaling pathways regulate tight junctions, and consequently endothelial barrier and blood–brain barrier, as well as induce inflammation [80]. Furthermore, PI3K plays an important role in mediating the effects of sEH inhibition [81,82]. In relation to this observation, in our study, the sEH inhibitor also significantly affected pathways including focal adhesion, tight junctions, adherent junctions and transendothelial migration, all pathways that regulate endothelial permeability. Vascular endothelial growth factor, VEGF, was another significantly modulated cell signaling pathway in our studies. This observation can be corroborated a previous study that suggested that the sEH inhibitor can increase EETs concentrations to levels capable of increasing VEGF which enhances cell survival signaling in neurons leading to less neuronal cell death and presenting a potential mechanism underlying the neuroprotection properties of sEH inhibition [83]. Therefore, the use of a transcriptomic approach allowed us to reveal that the sEH inhibitor possesses not just a gene specific, but rather a complex mode of global multiomic protective action, largely at the cell signaling level for endothelial barrier integrity and neuroinflammation, greatly adding to our understanding of the role of sEH inhibition at the genomic level in the brain microvascular endothelium as it relates to glycemia. In addition, the inhibitor effects were not correlated to lipids or insulin levels or to the female estrous cycle in mice.

In this study, we also showed that the effect of the sEHI was different for the LGD vs. the HGD. To our surprise, the effects of the sEH inhibitor were primarily manifest in the LGD and to a much lesser degree in the HGD. In understanding this difference, we first considered that the actions of the sEH could reflect effects that are mediated by EETs. EETs are synthesized from arachidonic acid and are metabolized to dihydroxyeicosatrienoic acids (DHETs) through hydration by sEH [84]. Thus, Inhibition of sEH will result in increased levels of EETs [84,85]. Several previous studies have shown that EETs themselves can modulate expression of genes. Some of these genes are the same as those observed in our study or are involved in the regulation of the same cellular processes as those identified using our genomic data. For example, our genomic study revealed that the sEHI regulated expression of genes coding for cyclic AMP, the cyclooxygenase family of proteins, as well as genes involved in interleukin, TNF-α, and NF-κB pathways. In other systems, such as in cultured macrophages treated with 14,15-EET, a significant reduction of mRNA expression of IL-1β and TNF-α has been observed [86]. The mechanisms regulating vasorelaxation/constriction and anti-inflammatory activities of EETs also involve inhibition of IκB kinase (IKK), which causes activation of nuclear factor κB (NF-κB) and consequently regulation of the expression of cell adhesion molecules. EETs also cause inhibition of cyclooxygenase 2 (COX-2) expression and inducible nitric oxide synthase (iNOS) expression [87]. It has also been observed that EETs can activate PPARα [88], suggesting that EET-induced regulation of gene expression occurs through an intracellular mechanism. Additional data suggests that the 14, 15 EET receptor was linked to a cyclic AMP and protein kinase A (PKA) signal transduction pathway that subsequently downregulates the EET receptor [89]. Furthermore, our correlation analysis contributed to our understanding and identified significant negative correlations of the DEGs involved in some of these key cellular pathways including the PI3K-Akt signaling (*Rab2a*, *Eif4e*, and *Rps6-Ps4*), focal adhesion (*Rap1b*), TNF-alpha NF-kB Signaling Pathway (*Akap8*, *Gm7429*, and *Gm9703*), and PPAR signaling (*Fabp7*). Considering that our transcriptome analysis identified differentially expressed genes involved in the regulation of these important cellular pathways, and these DEGs negatively correlated to EETs/DHETs ratio, we could hypothesize that the genomic effects observed are at least partially due to increase level of EETs. In agreement with this hypothesis, our brain oxylipin metabolic data revealed that mice on the LGD, but not on the HGD, that were treated with the sEHI indeed had higher EETs/DHETs ratios.

### 3.3. Effects of the sEHI on the HGD

In our study, inhibition of sEH in the HGD diet condition had a very limited effect on genomic modifications in brain hippocampal microvessels in female mice. In mice fed the HGD, treatment with the sEHI resulted in changes in the expression of only 200 genes, 10 times fewer than when the sEH inhibitor was given to mice on the LGD. The absence of a statistically different effect of the sEH inhibitor on mice on the HGD, compared to mice on the LGD, is interesting and was also surprising to us. Although we are not clear on the operative mechanism(s), we hypothesize that the HGD can affect expression of sEH itself. Indeed, it has been observed both in vivo and in vitro that high glucose levels suppress sEH expression at both the level of mRNA and protein expression [90]. Regarding this observation, we showed that glucose levels were in fact significantly higher in the HGD+sEHI compared to the HGD, whereas we observed no significant difference in glucose levels in the LGD+sEHI compared to the LGD. Thus, we could postulate that the higher glucose levels in HGD fed mice decrease levels of sEH, and as a consequence, the inhibitor given to the mice on the HGD had less target protein to inhibit, and consequently less ability to exert its effect.

Another possible explanation for the observed difference in the genomic responses to sEH inhibitor depending on diet, whether LGD or HGD, is the impact on expression of genes involved in the pathways related to EETs production. EETs are synthetized from arachidonic acid obtained from hydrolysis of phospholipids, like sphingolipids, present in the cellular membrane. Our genomic analysis identified several cellular metabolic pathways in mice on the LGD treated with the inhibitor related to fatty acid and AcetylCoA and phospholipid synthesis, pathways not identified in mice with inhibitor on HGD. Moreover, our genomic data also revealed an increase in the expression of genes coding for phospholipases in mice on the LGD with sEHI, such as *PLA2g2a*, *PLA2g2e* and *PLA2g3*. In contrast, these genes presented no change in expression in mice on the HGD with sEHI. This observation suggests the effect of the inhibitor in mice on the LGD results in increased expression of phospholipases which could result in an increase in arachidonic acids, and therefore production in EETs levels. In accordance with the genomic results, our metabolic data showed that indeed the sEHI increased the EETs/DHETs ratios in mice on the LGD but had no statistical effect in the mice on the HGD.

## 4. Materials and Methods

### 4.1. Experimental Animals, Diet, and Treatment

Throughout this study, mice were housed in the University of California, Davis Mouse Biology Program with 2 to 3 mice per cage with a 12-h light/dark cycle in a temperature- and humidity-controlled environment. Food and water were provided ad libitum throughout the study except where indicated as part of experimental procedures. Vivarium staff monitored food intake, water, and activity. The study was carried out in compliance with the policy of Public Health Service on the Humane Use and Care of Laboratory Animals and the University of California, Davis, Institutional Animal Care and Use Committee (IACUC) approved protocol number 20,943 on 18 April 2019.

C57BL/6J female mice (Jackson Laboratories, stock 000664) were obtained at 19 weeks of age and were fed a standard chow diet (catalog no 0915 from Envigo Teklad Diets, Madison, WI) and acclimated for one week before starting the study procedures. Mice were then randomly assigned and fed one of two experimental diets for 12 weeks. The experimental diets consisted of a low glycemic diet (LGD, catalog number TD.08485 from Teklad Envigo, Madison, WI made of 67.9% carbohydrate, 19.1% protein, 13% fat, represented as percent kcal, with 12% sucrose by weight), or a high glycemic diet (HGD, catalog number TD.05230, Teklad Envigo, Madison, WI made of 68.7% carbohydrate, 18.7% protein, 12.6% fat, represented as percent kcal, with 34% sucrose by weight). A detailed dietary composition of the diets is provided in Supplemental Table S11. The HGD diet is rich in sucrose and was chosen since sucrose has a high glycemic index, and compared to LGD, the quantity of sucrose is double. A diet based on sucrose enrichment has been well studied in the context of neurodegenerative brain diseases such as Alzheimer’s disease [91], of interest to our work, and sucrose can be responsible for cognitive disorders and dementia [92].

An additional group of mice receiving each diet also received t-AUCB (trans-4-[4-(3-adamantan-1-yl-ureido)-cyclohexyloxy]-benzoic acid, Cayman Chemical, Ann Arbor, MI), a soluble epoxide hydrolase inhibitor (sEHI), provided in the drinking water using polyethylene glycol 400 (PEG400) (Millipore, Burlington, MA) as a vehicle throughout the experimental diet feeding. PEG400 vehicle control was used in the sEHI study groups. Consistent with prior protocols, the final contents of the drinking water were 1% (by volume) PEG400 and 10mg/L t-AUCB [93,94]. Consistent with previously published work [95], mice consumed approximately 7 to 7.5 mL of water each day, corresponding to 2.5 to 3 mg of t-AUCB (sEHI) per kg per day. The PEG400 vehicle was not added to drinking water of the control mice; however, 1% PEG400 is a low amount and in prior studies has been shown to not have a biological effect [96]. Mice were sacrificed at 32 weeks of age at the end of the 12-week dietary intervention period. Body weight was measured at baseline and at the completion of the experimental diet exposure. There were the four experimental treatment groups (n = 7 mice per group): LGD only, LGD with sEHI (LGD+sEHI), HGD only, and HGD with sEHI (HGD+sEHI).

### 4.2. Serum Lipid, Glucose, and Insulin Assays

Following completion of the dietary feeding period, mice were fasted overnight for eight hours, and blood was extracted via submandibular nick for the pre-diet samples, and through ventricular puncture in the post-diet samples at the time of sacrifice and stored at −80 °C. Fasted serum samples were used to measure glucose, insulin, and lipid concentrations. Glucose levels were determined utilizing enzymatic assays from Fisher Diagnostics (Middleton, VA, USA), and insulin levels were measured via electrochemiluminescence from Meso Scale Discovery (Rockville, MD, USA) in accordance with the manufacturer’s protocols. Enzymatic assays from Fisher Diagnostics (Middleton, VA, USA) and precipitation separation from AbCam (Cambridge, MA, USA), modified for a microplate setup were used to measure low-density lipoprotein cholesterol (LDL), high-density lipoprotein cholesterol (HDL), and total cholesterol (TC). The UC Davis Mouse Metabolic Phenotyping Center (MMPC) Metabolic Core conducted all serum assays in this study.

### 4.3. Vaginal Lavage and Assessment of Estrus Cycle

Following completion of the 12-week dietary feeding period, mice were anesthetized by intraperitoneal xylazine/ketamine. Prior to sacrifice, vaginal lavage was performed using sterile phosphate-buffered saline (PBS). The PBS with vaginal cells was then applied to a glass slide and allowed to dry. Slides were stored at room temperature until staining with 0.1% cyrstal violet. Phase of estrus cycle was assessed by examining the stained cells collected by the lavage using light microscopy. Samples were categorized as proestrus, estrus, metestrus, or diestrus based on the cell types observed as previously described [97].

### 4.4. Isolation and Cryosection of Murine Brain Hippocampus

Tissue collection was performed at the end of the 12-week dietary feeding, during the light phase of the light/dark cycle. After euthanasia by exsanguination under anesthesia, intact brains were removed rapidly and sliced into regions comprising the temporal lobe segment containing the hippocampus and embedded in HistoPrep Frozen Tissue Embedding Media (Fisher Scientific, Pittsburgh, PA, USA) under RNAse free conditions. The hippocampus and hippocampal neurons were identified by hematoxylin staining of the brain sections in the medial aspect of the temporal lobe and visualized using microscope as described previously [26]. The hippocampus was then subjected to coronal cryosectioning (8 µm, Leica Frigocut 2800n Cryostat, Leica Biosystems, Buffalo Grove, IL, USA) and captured on charged RNA-free PEN Membrane Glass slides, coated with RNAlater^®^-ICE (Life Technologies, Grand Island, NY, USA) to hinder RNA degradation, and kept at −80 °C until needed.

### 4.5. Laser Capture Microdissection (LCM) of Hippocampal Microvessels

When ready for use, cryosections from the hippocampal segments were submerged in nuclease-free water and dehydrated in desiccant. Alkaline phosphatase staining with 5-bromo-4-chloro-3-indolyl phosphate/nitro blue tetrazolium chloride (BCIP/NBT) substrate was used to identify hippocampal endothelial microvessels (<20 um) in the cryosections of the hippocampus for transcriptomic analysis as formerly described [98]. Laser capture microdissection (LCM) was subsequently performed to extract the microvascular endothelium from hippocampal cryosections via capture of the entire vessel wall under direct microscopic visualization using a Leica LMD6000 Laser Microdissection Microscope (Leica Microsystems, Wetzlar, Germany). Microvessels were not specified by hippocampal region or subregion, but they did largely represent endothelial enriched regions in hippocampus dorsal segments that would have contained CA1 and CA3 regions.

### 4.6. RNA Extraction from Laser Captured Brain Microvessels

Total RNA was isolated from the laser-captured hippocampal microvessels (300 microvessels per mice, n = 3 mice per diet/inhibitor group) utilizing an Arcturus PicoPure™ RNA Isolation Kit (Thermo Fisher Scientific, Santa Clara, CA, USA) in accordance to the manufacturer’s protocols. RNA quality of the LCM-derived microvessels was determined using Nanodrop. RNA quantification was conducted as per Affymetrix RNA quantification kit with SYBR Green I and ROX™ Passive Reference Dye protocol from Affymetrix, Santa Clara, CA. The entire amount of micro dissected tissue was subjected to RNA preparation for microarray analysis yielding 122.3 pico grams of RNA per array.

### 4.7. Microarray Hybridization and Transcriptome Analysis

For transcriptomics analysis we used Clariom D Mouse Array from Thermo Fisher, Santa Clara, CA. (one array per mouse, n = 3 array biological replicates per diet/inhibitor group), which includes more than 7 million probes for protein and non-protein coding genes including microRNAs (miRNAs), long non-coding RNAs (LncRNAs), and small nucleolar RNAs (snoRNAs). 122.3 pico grams of RNA was utilized to generate complimentary RNA (cRNA) and sscDNA with GeneChip^®^ WT Pico Kit (Thermo Fisher, Santa Clara, CA). The amount of RNA yielded from 300 brain microvessels from each animal was sufficient to yield the required amount of sscDNA (5.5 ug) suggested by the manufacturer for microarray analysis. SscDNA was fragmented by uracil-DNA glycosylase (UDG) and apurinic/apyrimidinic endonuclease 1 (APE 1) and labeled by terminal deoxynucleotidyl transferase (TdT) using the DNA Labeling Reagent that is covalently linked to biotin. Fragmented and labeled sscDNA samples were then hybridized, stained, and scanned by the UC Davis Genome Center shared resource core according to the Thermo Fisher Scientific WT array hybridization protocol. Hybridization of the fragmented and labeled sscDNA samples was performed with GeneChip™Hybridization Oven 645, and then the GeneChip™ Fluidics Station 450 was used to wash and stain the samples. The arrays were scanned with GeneChip™ Scanner 3000 7G (Thermo Fisher Scientific, Santa Clara, CA, USA). Quality check of the data analysis and microarrays was done using Thermo Fisher Scientific Transcriptome Analysis Console software version 4.0.2. The microarray data in this study has been deposited in the GEO with the accession number GSE195975.

### 4.8. Bioinformatic Analysis

Two of the study investigators (SN and DM) performed bioinformatic analysis of differentially expressed genes using multiple software tools. To determine the effect of the HGD diet, we compared the HGD to the LGD groups. To determine the effect of the inhibitor, we compared the HGD+sEHI to the HGD alone, and then the LGD+sEHI to the LGD alone.

We used ClustVis [99] and Metaboanalyst [100,101] to obtain the Principal Component Analysis (PCA) plot of identified differentially expressed genes (DEGs). ShinyGo online database [102] was used to identify different types of RNAs (coding and non-coding). Targets of differentially expressed miRNAs were identified using Mienturnet [103]. Targets of differentially expressed LncRNAs were identified using LncRRIsearch [104] and Rtools CBRC [105]. We used GeneTrial2 online database [106,107] to conduct canonical pathway analysis. Cytoscape software (version 3.7.1) [108,109] was used to construct and visualize networks. Enrichr [110] was used to identify transcription factors and their targets. PermutMatrix software [111,112] was used to perform hierarchical clustering and heat map representations of differentially expressed genes (DEGs). The significant associations (corrected *p* values < 0.05) of the identified DEGs with human diseases were analyzed using Comparative Toxicogenomics database [113]. This database consists of literature-based, manually curated interactions that are integrated to provide a knowledge base that connects chemicals, genes, phenotypes, diseases, and exposures information.

### 4.9. Determination of EETs/DHETs Ratio within Brain Free Oxylipins

The right hemispheres of the brains from each experimental animal study group (n = 7/group) were homogenized in 200 μL of methanol containing 0.1% butylated hydroxytoluene and 0.1% acetic acid. The samples were spiked with deuterated surrogate standards. The samples were then precooled for 30 min at −80 °C and homogenized with a bead homogenizer (Nextadvance Bullet Blender Strom 24, Troy, NY, USA) using zirconia beads. The homogenized samples were centrifuged at a speed of 15,871× *g* for 10 min at 4 °C. The supernatant was loaded onto 60 mg Waters Oasis HLB 3cc solid phase extraction (SPE) columns (Waters, Milford, MA, USA). The columns were rinsed twice with SPE buffer and subjected to 20 min of vacuum (≈15 psi). Oxylipins were eluted with methanol and ethyl acetate, dried under nitrogen and reconstituted in methanol. Ultra-high pressure liquid chromatography tandem mass spectrometry (UHPLC-MS/MS) using Agilent LC system 1290 (Agilent Corporation, Santa Clara, CA, USA), coupled to an Agilent 6460 Triple Quadrupole MS system (Agilent Corporation) was used for oxylipins analysis. The sum of all detected EETs was divided by the sum of all detected DHETs for each sample to generate the EETs/DHETs ratio for each experimental group.

### 4.10. Assessment of Cognitive Function

At the completion of dietary feeding, 32 weeks old female mice from each of the 4 diet/inhibitor treatment groups (LGD, HGD, LGD+sEHI, and HGD+sEHI), n = 7 mice/group, were subjected to cognitive function test using Y-maze spontaneous alternation behavior (SAB) assessment, Y-maze is a behavioral test for measuring the willingness of rodents to explore new environments and a commonly used tool to measure of spatial working and short term memory in mice [114]. Mice were adapted to the testing room for 30 min, then placed in the center of the Y-shaped maze with three white plastic arms (35L × 8W × 15H cm) at 120° angles from each other. Mice were allowed to freely explore the three arms of the Y-maze for 8 min and tracked with an overhead camera to record the number of entries into each arm. Entry into each arm (arm 1–3) after entry to the center (arm 4), total distance traveled, latency to entry and frequency, and an alternation score was computed as the number of times the three arms were sequentially entered. Data were expressed as the % alternation score, the number of alternations divided by maximum alteration triplets and presented as means ± SEM. The Y-maze studies were performed by the UC Davis Mouse Biology Program, Phenotyping Center (MMPC).

### 4.11. Statistical Analysis

For microarray, statistical analysis of microvessel transcriptomes was conducted using ANOVA ebayes (Thermo Fisher Scientific Transcriptome Analysis Console software, Santa Clara, CA) with false discovery rate (FDR) correction. Differentially expressed genes (DEGs) from the microarray had ±2.0-fold change with significant *p* value less than 0.05. Mean body weight and plasma glucose, insulin, and lipid levels at the end of dietary feeding period were presented as means ± standard error of the mean (SEM), and significance determined at *p* ≤ 0.05 using two-way ANOVA (GraphPad Prism, San Diego, CA, USA). Pre and post diet intervention body weight, total cholesterol, glucose and insulin levels were represented as means ± standard error of the mean (SEM) and statistical significance (*p* ≤ 0.05) was determined using unpaired student’s *t*-tests (GraphPad software, La Jolla, CA, USA). EET/DHET ratios were compared using the Mann–Whitney U test as the data were not normally distributed; significance was determined by a *p* value < 0.05. We used Prism software (GraphPad, San Diego, CA, USA) to perform correlation analysis of the EETs/DHETs ratio to the differentially expressed genes involved in the significant cellular pathways in the diet/inhibitor comparison groups. We determined significance using spearman correlation (r) and *p* value < 0.05 at 95% confidence interval. Y maze cognitive function data was analyzed by unpaired student’s *t*-test (GraphPad software, La Jolla, CA, USA) and expressed as means ± SEM.

## 5. Conclusions

In this study, we showed for the first time in female hippocampal microvessels that the HGD modulated differentially expressed protein coding and noncoding genes involved in cellular pathways that could impair endothelial permeability, migration, endothelial barrier, and angiogenesis in female mice. As such, this study builds upon our recent work defining the role of a HGD on the global and multilevel molecular response in males [33], and in males compared to females [115], in the brain microvasculature.

In the present work, we also observed an increase in the EETs/DHETs ratio in the brain oxylipins of mice fed the HGD when compared to the LGD. We found that the DEGs involved in the regulation of actin cytoskeleton (*Ppp1cc*), PI3K-Akt signaling (*Pten*), adipogenesis (*Ndn*), insulin signaling (*Ppp1cc*, and *Pten*) and focal adhesion (*Ppp1cc*, and *Pten*) negatively correlated with EETs/DHETs ratio. This may have a protective effect against the harmful effects of high glycemia on the cellular pathways that could impede endothelial cell functions. Furthermore, we revealed that the sEHI on the LGD modulated a greater number of differentially expressed protein and noncoding genes involved in cellular pathways that could foster endothelial cell adhesion, permeability, and survival. We also showed that the sEHI had higher EETs/DHETs ratios on the LGD and no effect on the HGD. Moreover, the sEHI had lesser impact on the differential expression on the HGD and modulated fewer protein and noncoding genes. Furthermore, these molecular changes were associated with changes in measures of cognitive function in female mice and clinical cardiovascular and neurovascular diseases.

In alignment with our hypothesis, our results show that a HGD has deleterious effects on the female brain microvasculature by downregulating protein coding genes and noncoding genes involved in cellular functional pathways including apoptosis, inflammation, angiogenesis, and focal adhesion, which could result in endothelial barrier disruption and hyperpermeability. We also showed for the first time a multiomic response of the sEH inhibitor which modulated key cellular signaling pathways involved in regulation of endothelial permeability, cell survival, and barrier function. Surprisingly, the effect of the sEHI was greater with the LGD than the HGD and regulated metabolic pathways involved in anti-inflammatory EETs synthesis, associated with a higher EETs/DHETs ratio. In clinical trials, sEHIs reduce inflammation [116], improve vascular endothelial function [117,118], and play a protective role in diabetes [119] and hypertension [48,120], and in animal models of stroke and diabetes where they are neuroprotective [94,96,97].

Based on our findings, a conceptual schematic summary of the differential effect of the HGD vs. the LGD, and of the sEHI on the HGD as compared to the LGD, is provided in Figure 9A–C, and a summary about the postulated effects between the groups is provided in Table 3.

Thus, our findings suggest that sEHIs may also be promising therapeutic targets in the microvascular endothelial dysfunction that accompanies cardiovascular diseases and vascular dementias, including in response to high glycemic stress. However, clinical, and therapeutic consequences of molecular data remain to be determined.

## Figures and Tables

**Table 1:** Effect of the high glycemic diet (HGD) and the low glycemic diet (LGD) with and without soluble epoxide hydrolase inhibitor (sEHI) on cognitive performance in female mice. Values are mean ± SEM.

Y Maze Test	LGD	LGD+sEHI	HGD	HGD+sEHI
**Alternation triplets (%)**	44.3 ± 2.4	58.0 ± 4.2 *	48.7 ± 2.2	57.5 ± 5.5

* *p* < 0.05 when compared to LGD without inhibitor.

**Table 2:** Disease associations of the differentially expressed genes in the diet and inhibitor comparison groups in female mice fed a high and low glycemic diet with and without a soluble epoxide hydrolase inhibitor.

Disease	Corrected *p*-Value
**HGD vs. LGD**	
Brain Diseases	0.00781
Central Nervous System Diseases	0.02359
Intellectual Disability	0.00071
Metabolic Diseases	0.02285
Nervous System Diseases	0.000266
Neurobehavioral Manifestations	0.0000724
Neurologic Manifestations	0.000573
Vascular Diseases	0.03423
**HGD+sEHI vs. HGD**	
ND	ND
**LGD+sEHI vs. LGD**	
Amyotrophic Lateral Sclerosis	0.04935
Brain Diseases	0.0000137
Cardiovascular Diseases	0.0000134
Central Nervous System Diseases	0.000000035
Dementia	0.01501
Dyskinesias	0.000000946
Heart Diseases	0.000208
Hyperkinesis	0.04363
Intellectual Disability	0.0023
Movement Disorders	0.000136
Myocardial Ischemia	0.00386
Nervous System Diseases	1.2 × 10^−12^
Neurobehavioral Manifestations	0.0000162
Neurodegenerative Diseases	0.0000918
Neurologic Manifestations	1.13 × 10^−10^
Neuromuscular Diseases	0.00162
Vascular Diseases	0.0000724

ND: No disease associations.

**Table 3:** Summary of the differential effects of the high glycemic diet (HGD) and the low glycemic diet (LGD) comparison groups with and without soluble epoxide hydrolase inhibitor (sEHI).

Female Brain Hippocampal Microvessels	HGD vs. LGD	LGD+sEHI vs. LGD	HGD+sEHI vs. HGD
Differentially expressed genes (DEGs)	506	1420	200
Phospholipases (*Pla2*)	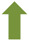	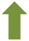	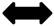
EETs/DHETs (brain oxylipins)	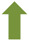	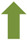	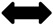
sEH (*Ephx2*)	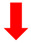	Blocked	Blocked
Pathways	*Angiogenesis*, *endothelial permeability, migration, and barrier function*. Rap1 signaling PI3K-Akt signaling Focal adhesion Regulation of actin cytoskeleton VEGF signaling	*Endothelial* *cell adhesions,* *permeability and* *survival*. PI3K-Akt signaling Focal adhesion Adherent junction Cell–cell adhesions VEGF signaling NF-Kb, TNF-α, COX-2, PPAR-α Signaling	*Fewer cellular* *pathways*. Proteasome degradation Mapk signaling

## Data Availability

The microarray data in this study has been deposited in the GEO with the accession number GSE195975.

## Supplementary Materials

The following supporting information can be downloaded at: https://www.mdpi.com/article/10.3390/ijms232113044/s1.
