# Supplementary material for: High Glycemia and Soluble Epoxide Hydrolase in Females: Differential Multiomics in Murine Brain Microvasculature
Source: Int J Mol Sci. 2022 Oct 27;23(21):13044. doi: 10.3390/ijms232113044 (PMC9655872; doi:10.3390/ijms232113044)
Supplement: Supplementary file 1 [file ijms-23-13044-s001.zip › ijms-1965252-supplementary.pdf]

Supplemental Figure S1: Histogram of differentially expressed protein coding genes pathways in hippocampal microvessels for the high glycemic diet (HGD) when compared to the low glycemic diet (LGD).

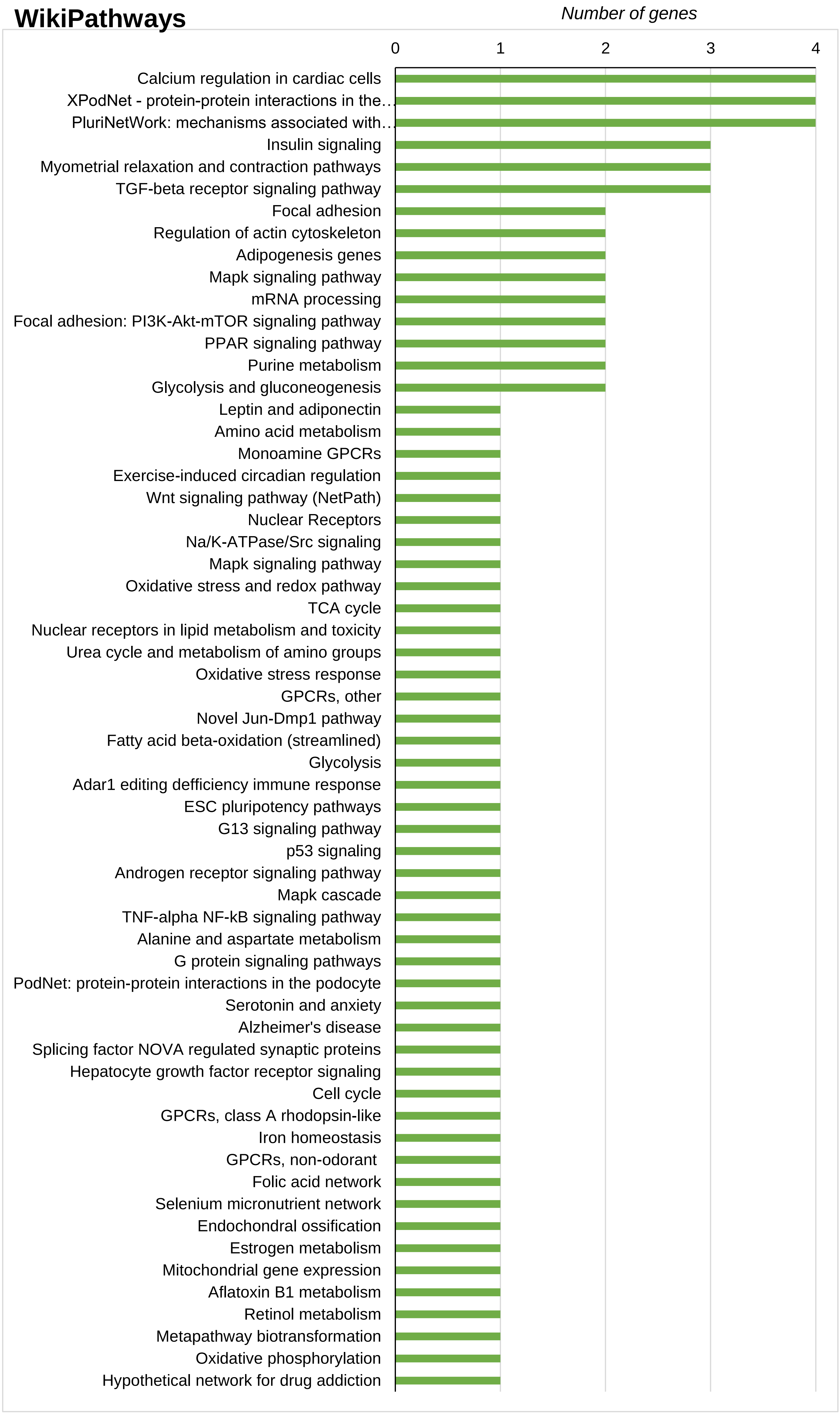

Supplemental Figure S2: Target gene networks of differentially expressed transcription factors (TFs) in hippocampal microvessels of female mice fed with the high glycemic diet (HGD) compared to the low glycemic diet (LGD).

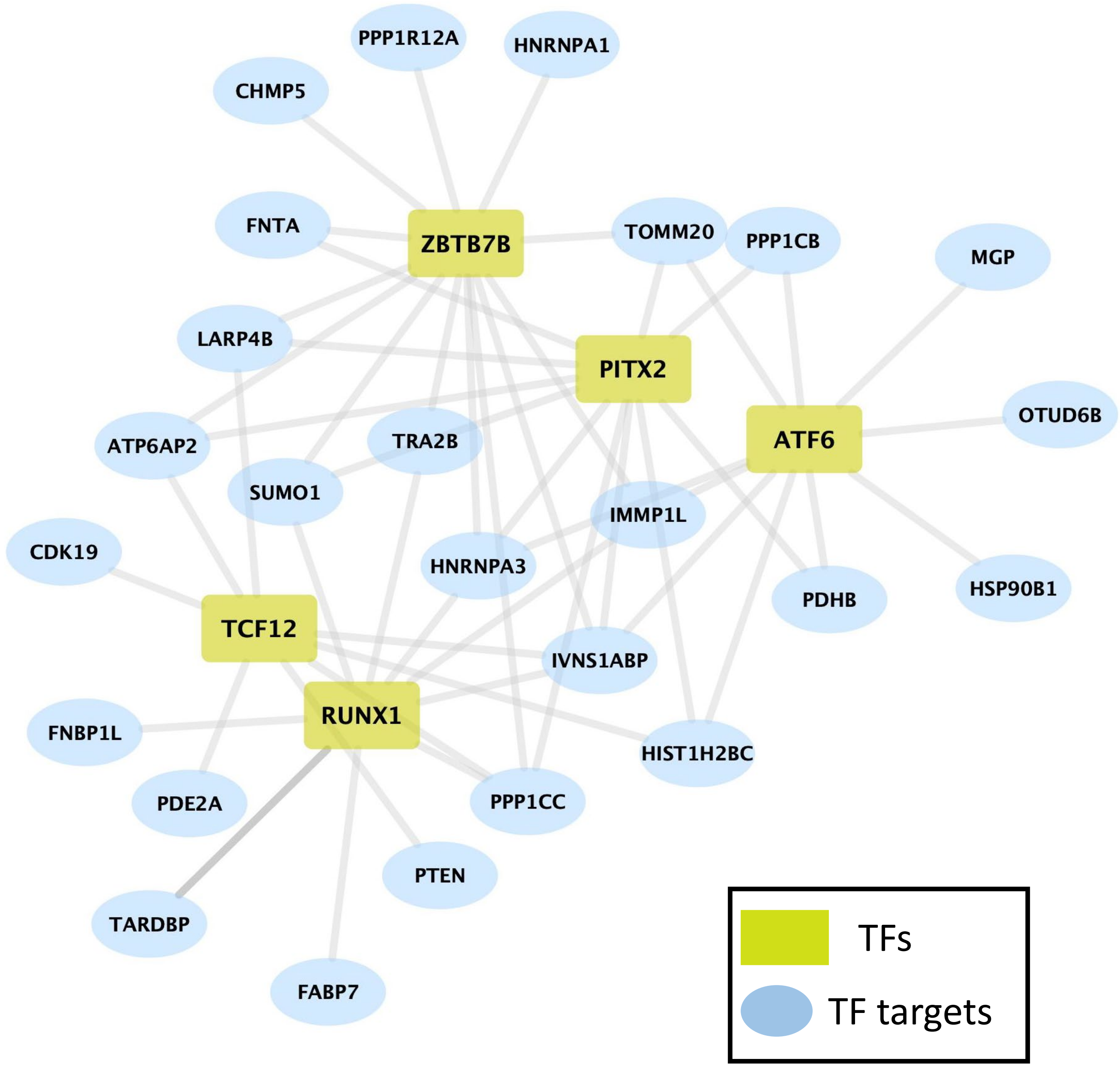

Supplemental Figure S3: Target gene networks of differentially expressed miRNAs in hippocampal microvessels of female mice fed with the high glycemic diet (HGD) compared to the low glycemic diet (LGD).

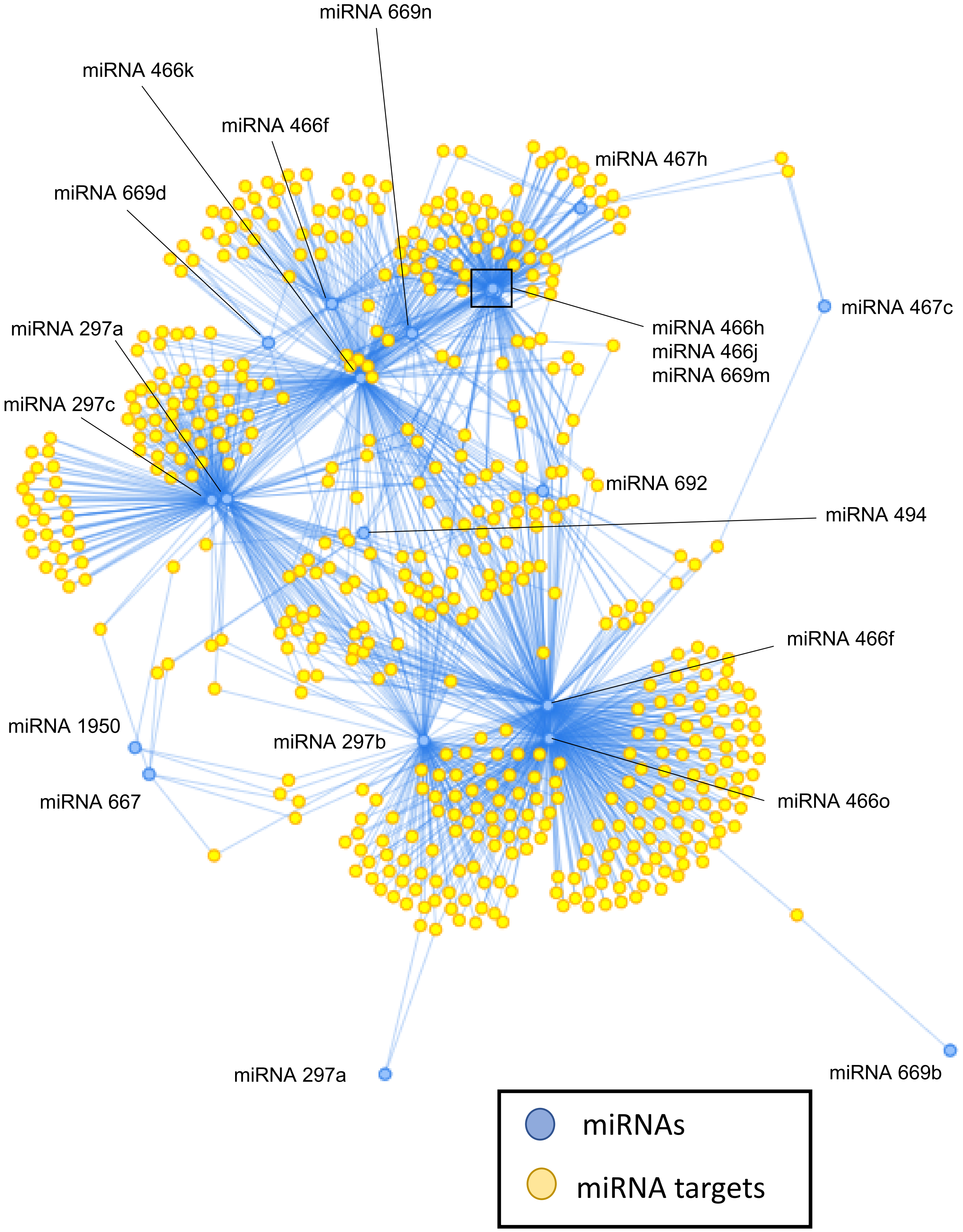

Supplemental Figure S4: Pathways of miRNA targets (HGD vs LGD)

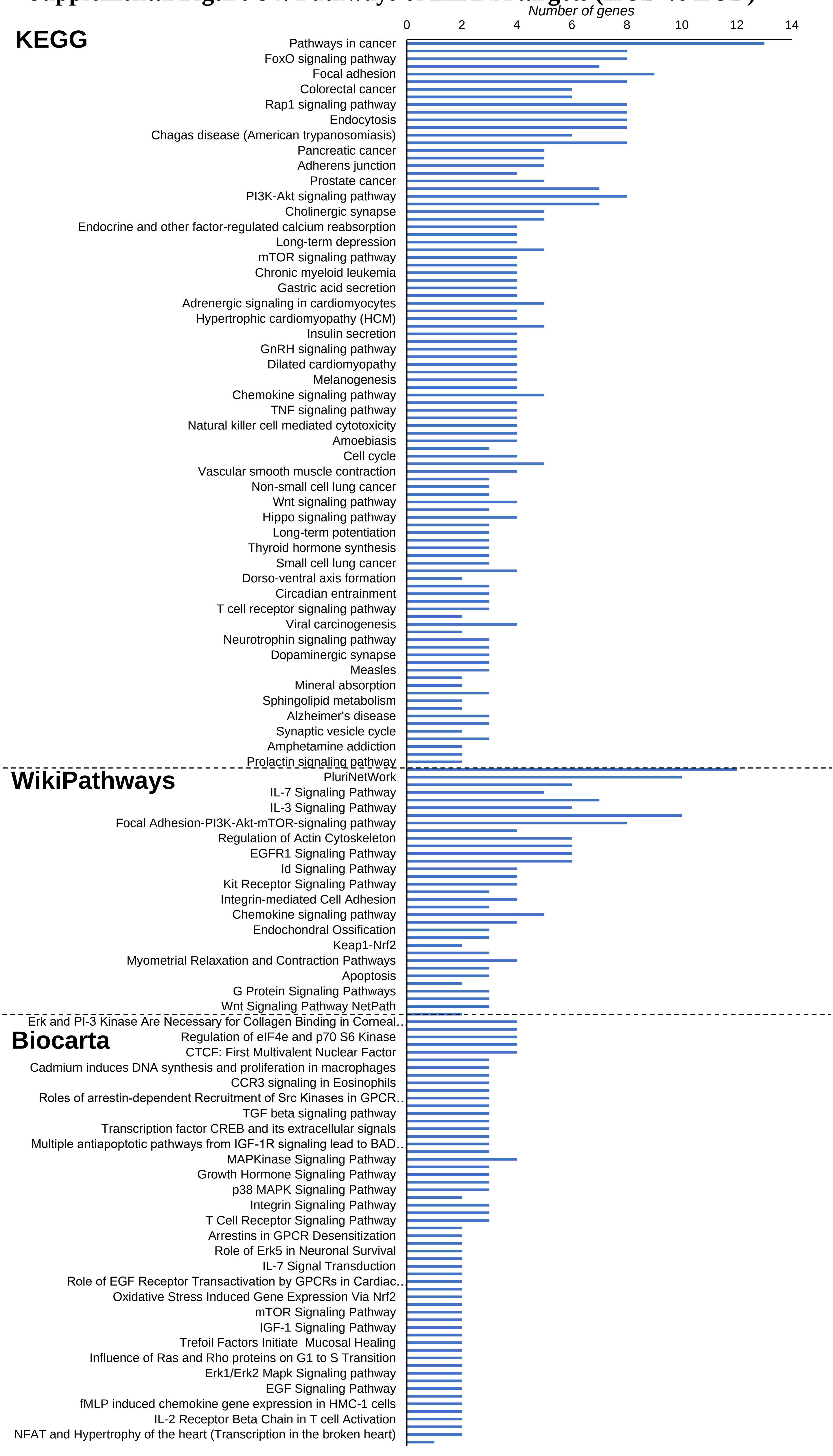

Supplemental Figure S5: Target gene networks of differentially expressed LncRNAs in hippocampal microvessels of female mice fed with the high glycemic diet (HGD) compared to the low glycemic diet (LGD).

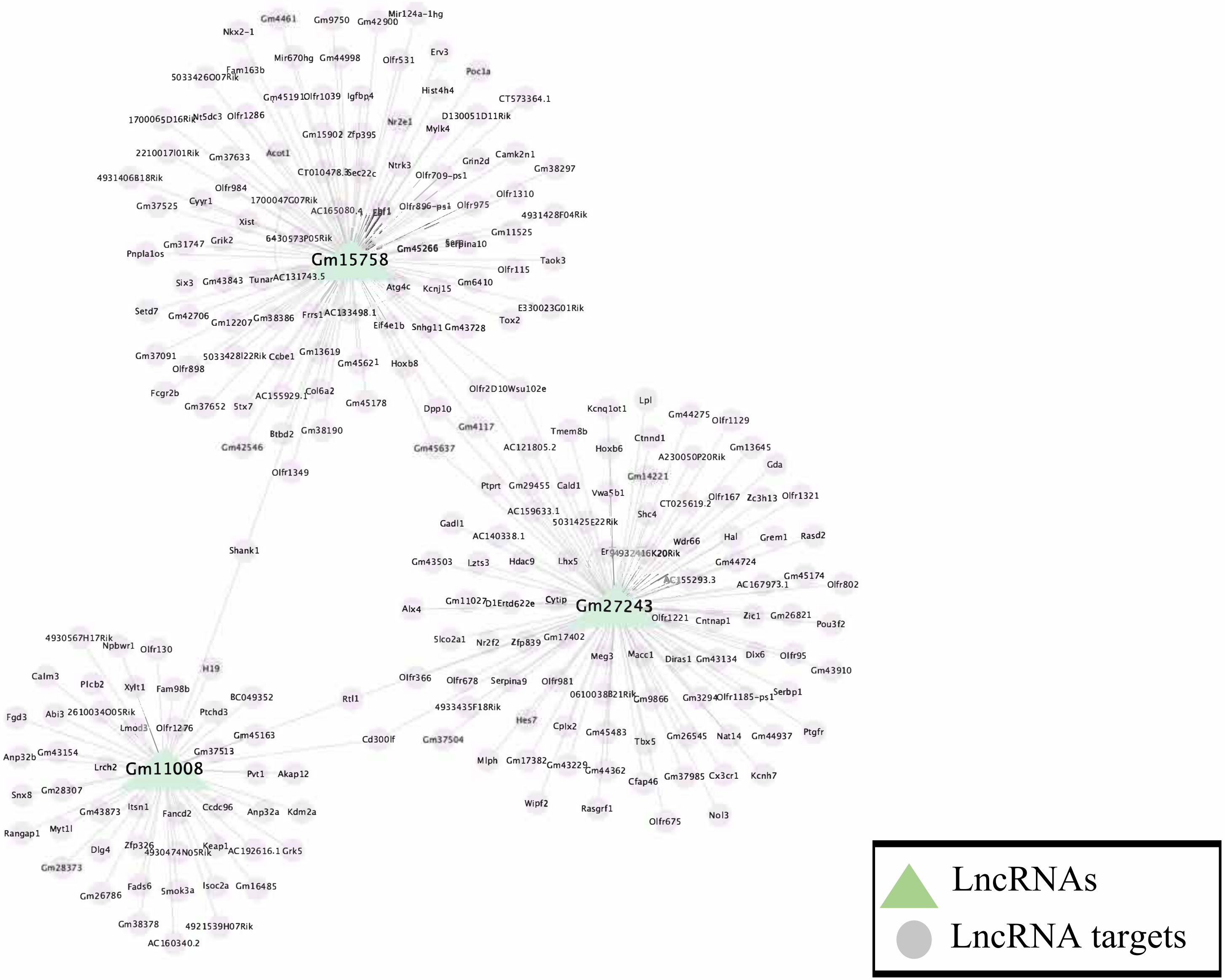

Supplemental Figure S6: Histogram of differentially expressed lncRNA targets pathways in hippocampal microvessels with the high glycemic diet (HGD) when compared to the low glycemic diet (LGD).

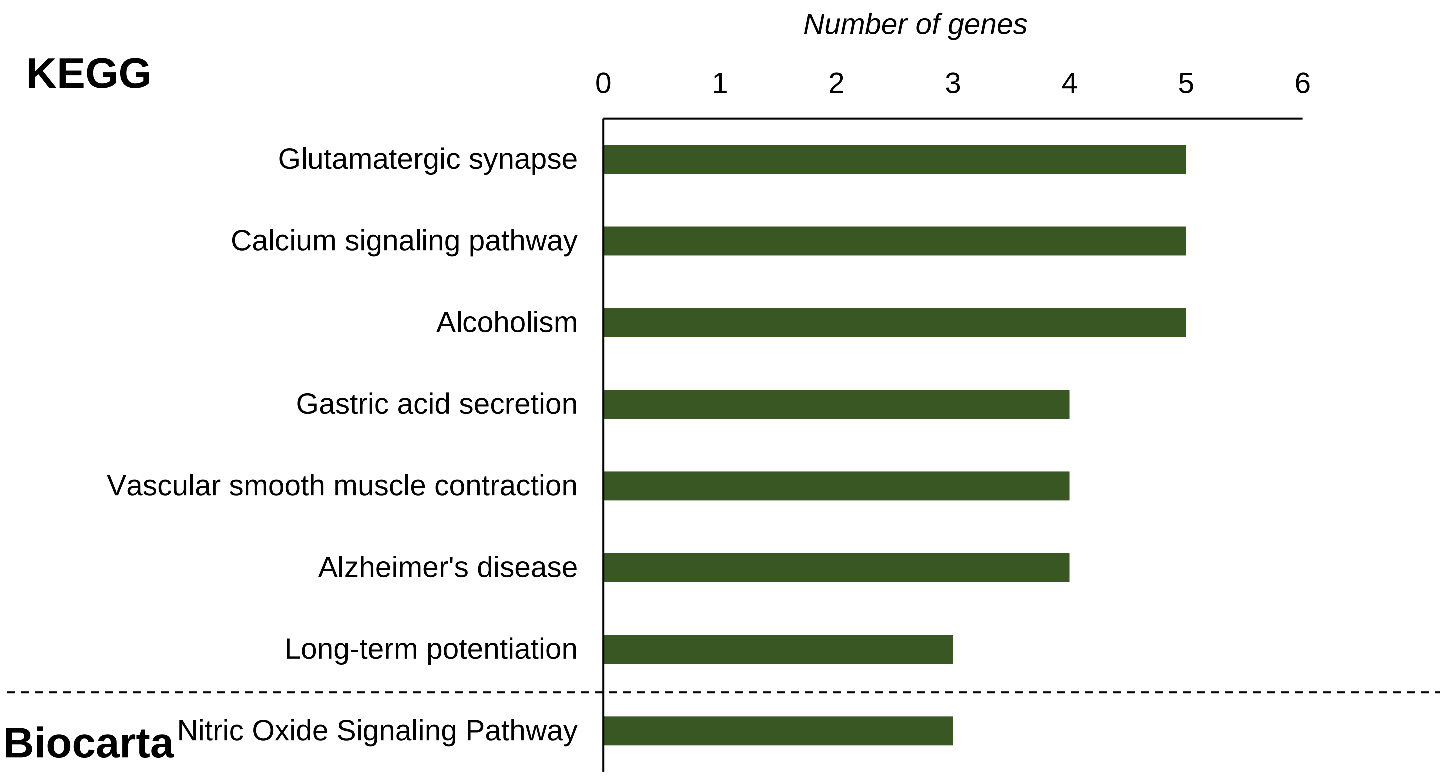

Supplemental Figure S7: Histogram of protein coding differentially expressed genes pathways in hippocampal microvessels for the low glycemic diet (LGD) with soluble epoxide hydrolase inhibitor (sEHI) compared to without sEHI treatment.

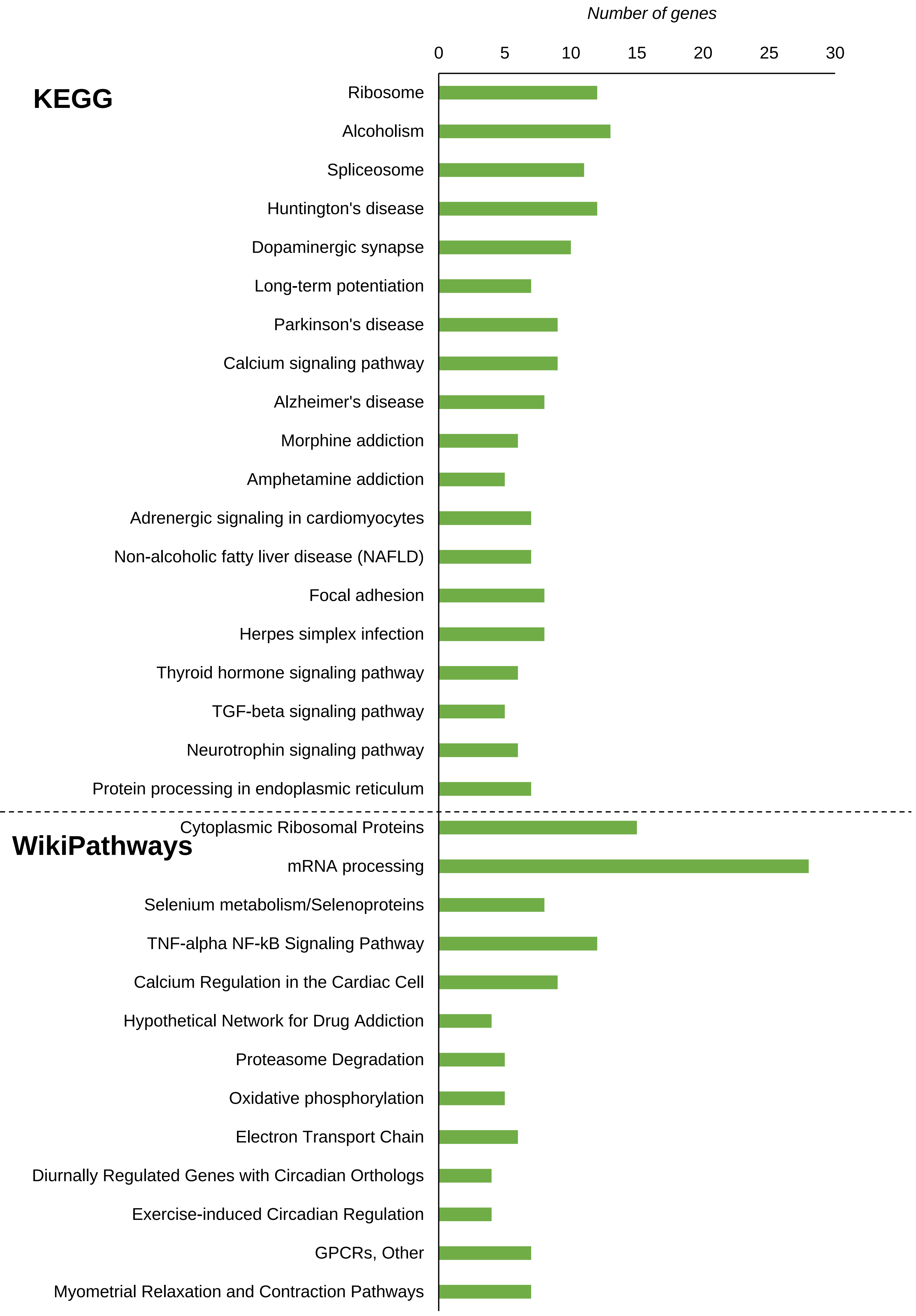

Supplemental Figure S8: Target gene networks of differentially expressed transcription factors (TFs) in the LGD+sEHI vs LGD.

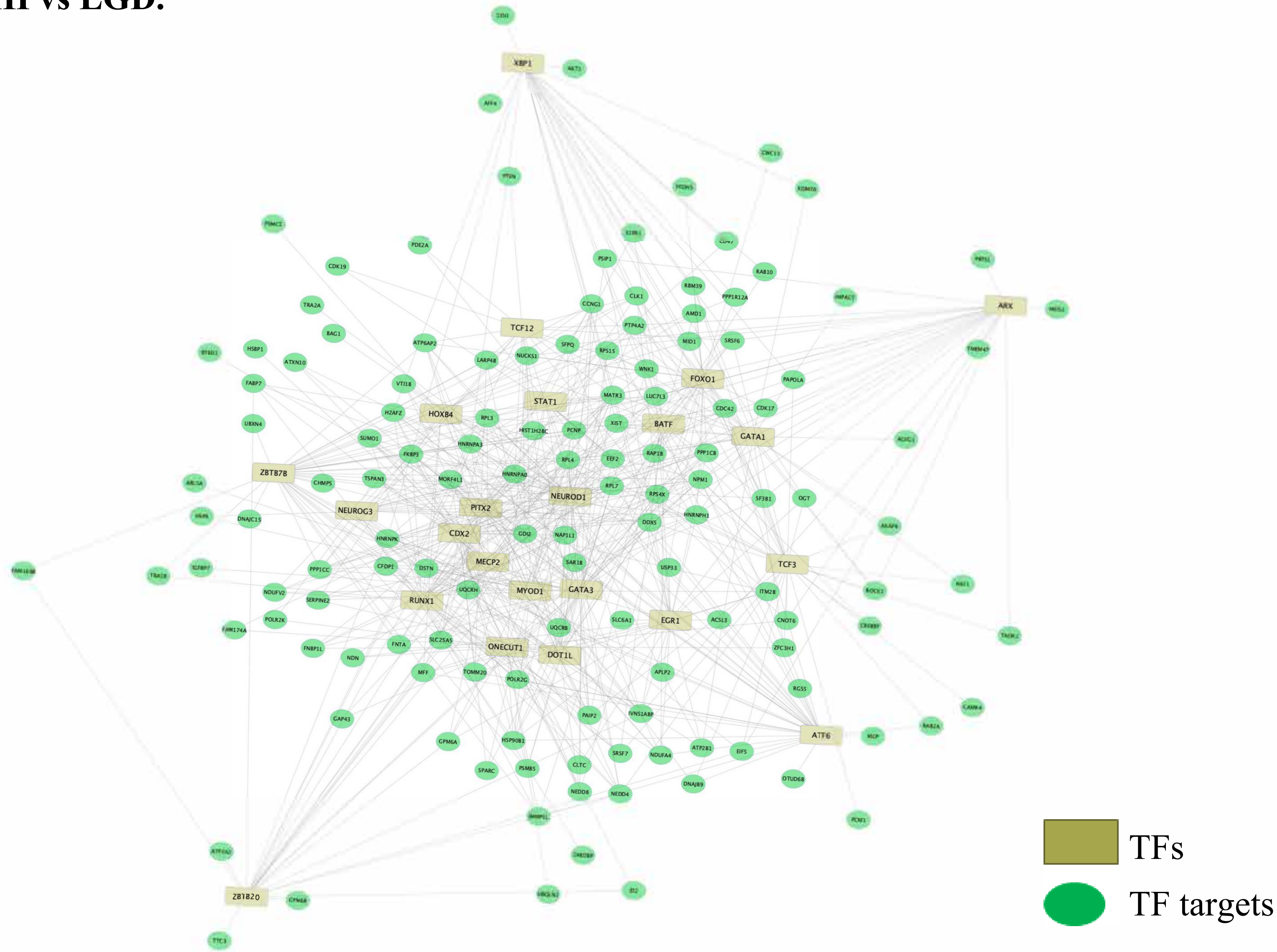

Supplemental Figure S9: Target gene networks of differentially expressed miRNAs in hippocampal microvessels for the low glycemic diet (LGD) with and without soluble epoxide hydrolase inhibitor (sEHI).

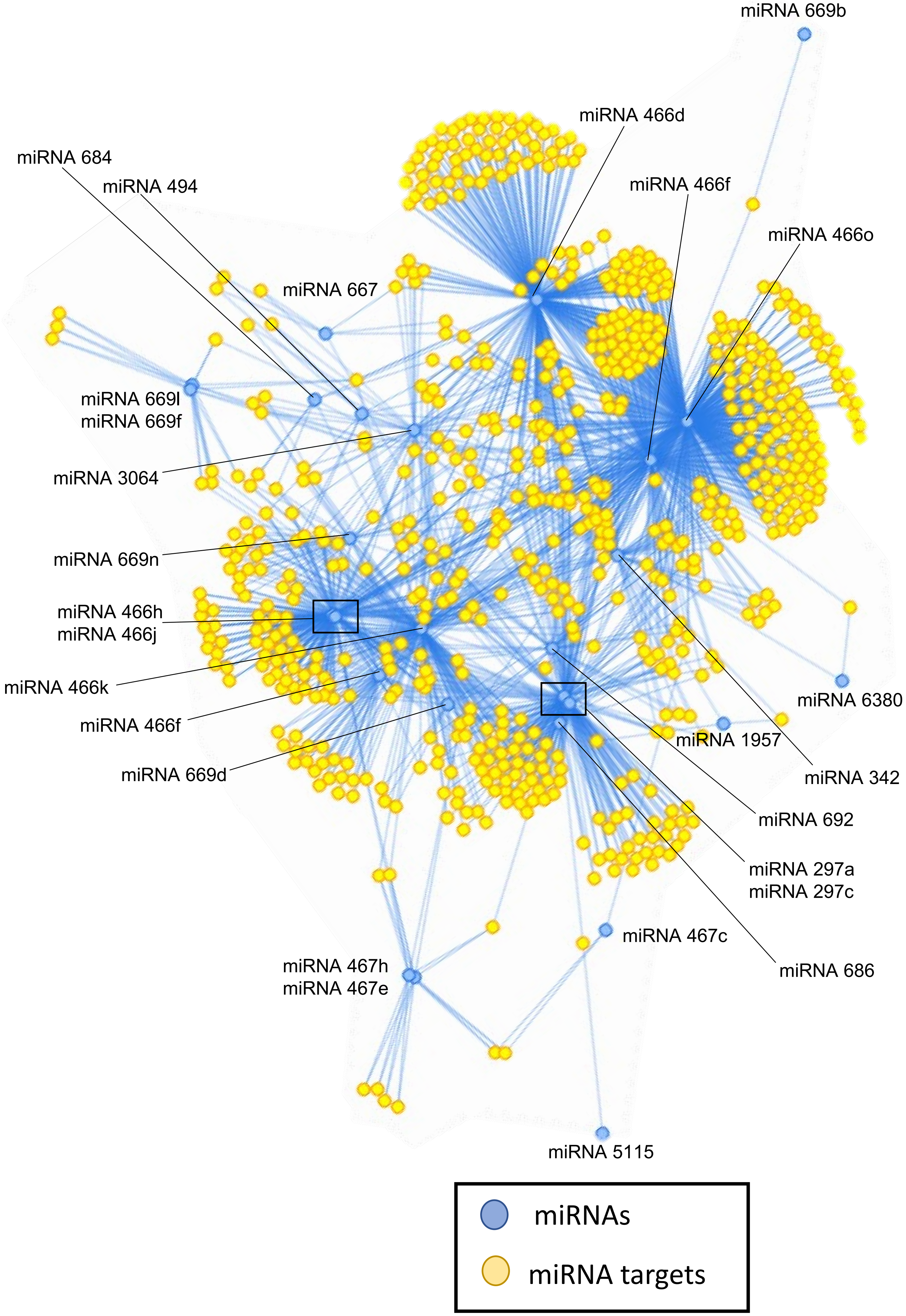

Supplemental Figure S10: Pathways of miRNA targets (LGD+sEHI vs LGD)

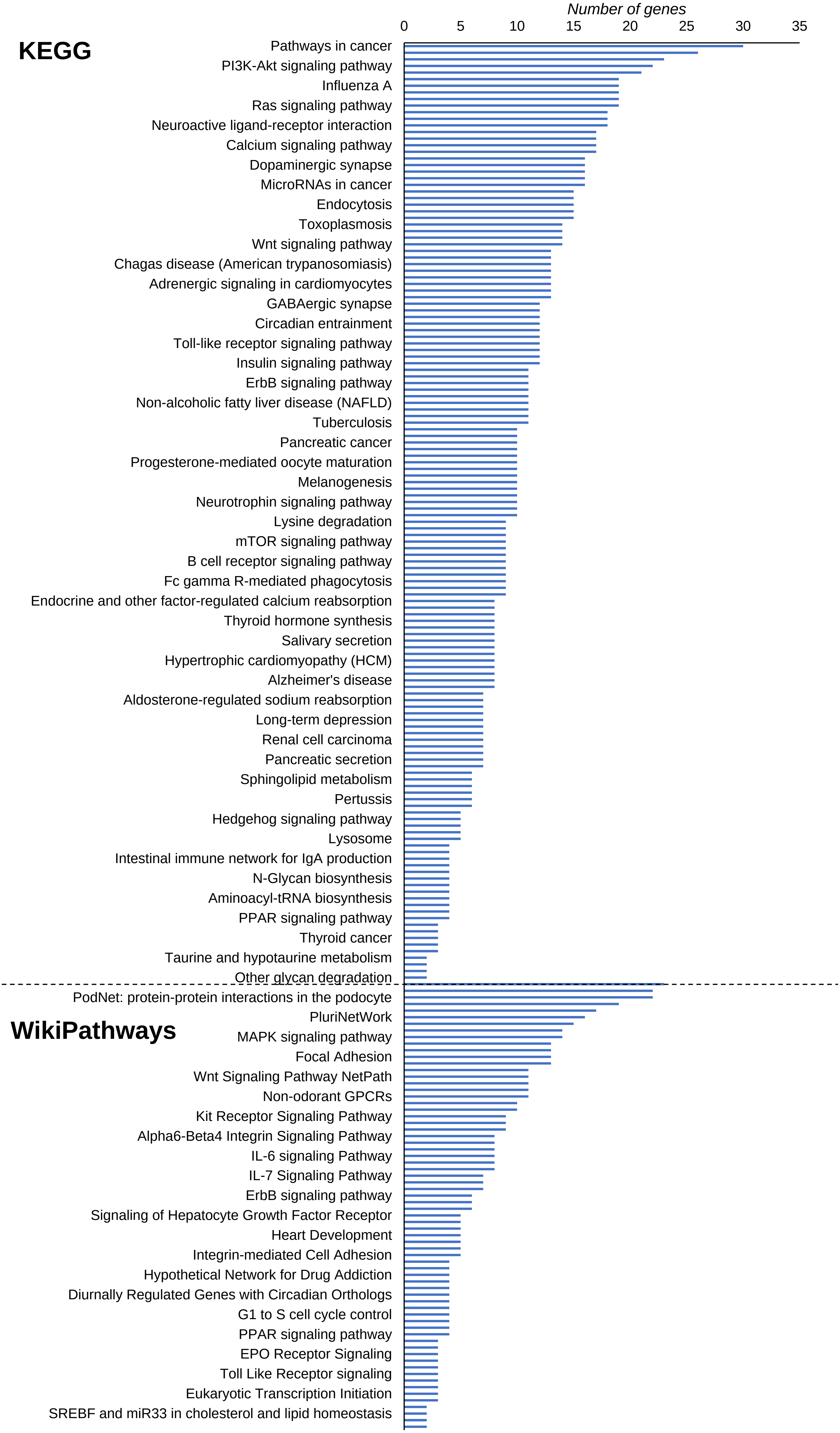

Supplemental Figure S11: Target gene networks of differentially expressed LncRNAs in the LGD+sEHI vs LGD).

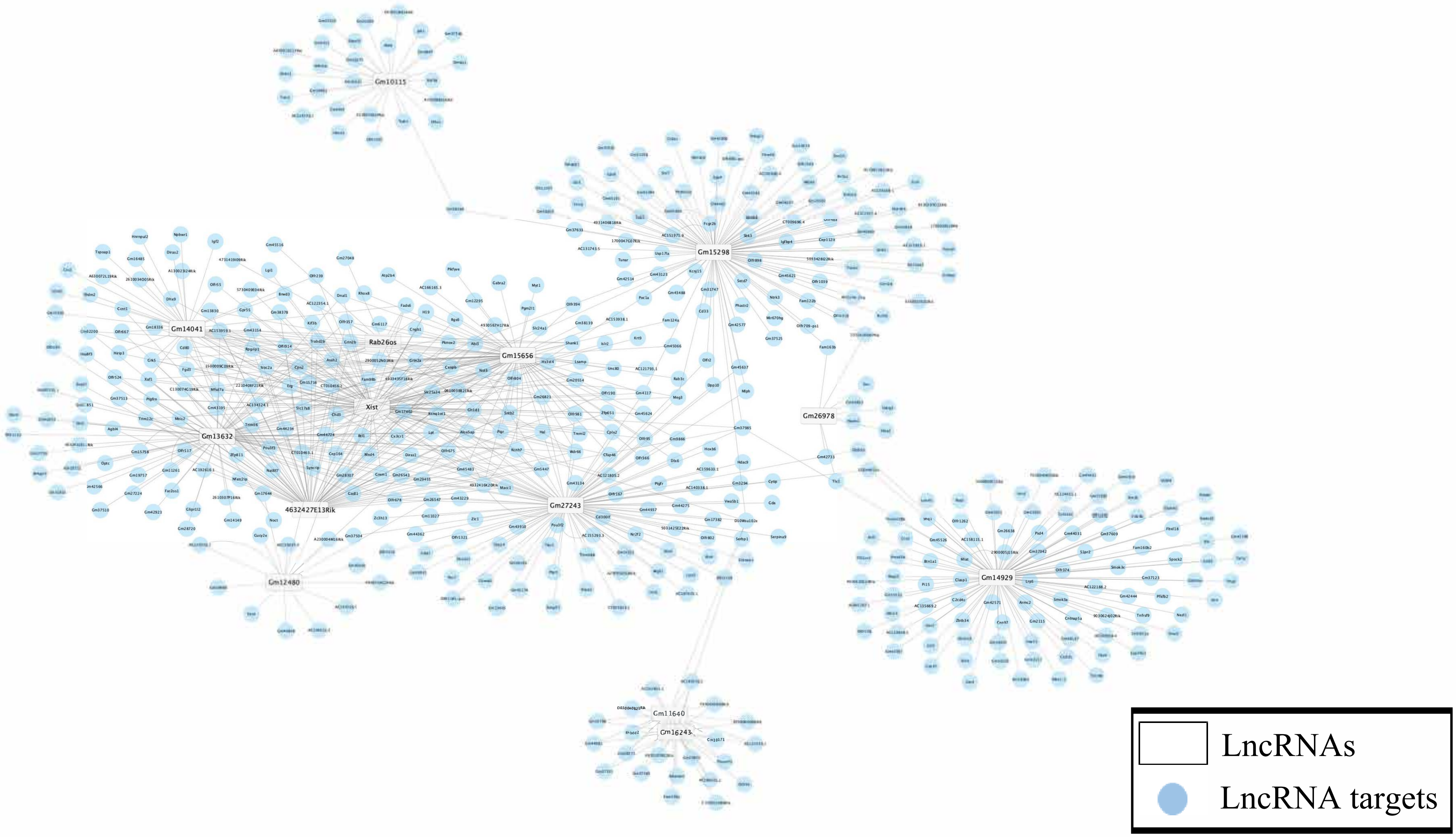

Supplemental Figure S12: Histogram of differentially expressed lncRNA targets pathways in hippocampal microvessels for the low glycemic diet (LGD) with soluble epoxide hydrolase inhibitor (sEHI) compared to without sEHI treatment.

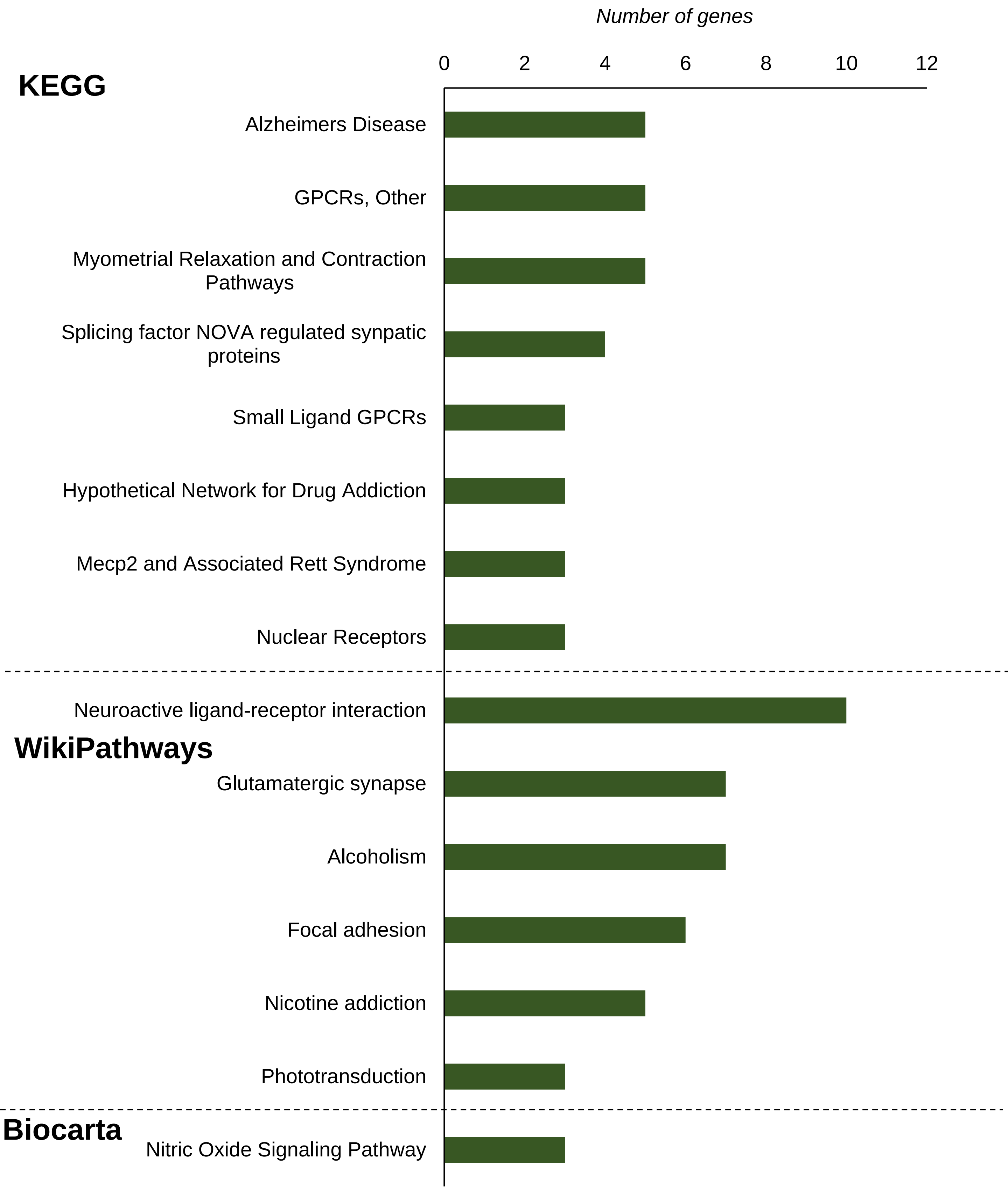

Supplemental Figure S1: Histogram of differentially expressed protein coding genes pathways in hippocampal microvessels for the high glycemic diet (HGD) when compared to the low glycemic diet (LGD). Cellular pathways of differentially expressed protein coding genes in hippocampus microvessels from the high glycemic diet (HGD) compared to low glycemic diet (LGD). The data are shown for three biological replicates for each dietary group. WikiPathways were identified using transcriptomics analysis console software.

Supplemental Figure S2: Target gene networks of differentially expressed transcription factors (TFs) in hippocampal microvessels of female mice fed with the high glycemic diet (HGD) compared to the low glycemic diet (LGD). The network of interactions between differentially expressed transcription factors (TFs, yellow-green boxes) and their target genes (blue ovals) of the high glycemic diet (HGD) compared to the low glycemic diet (LGD). TFs and their targets were identified using Enrichr database.

Supplemental Figure S3: Target gene networks of differentially expressed miRNAs in hippocampal microvessels of female mice fed with the high glycemic diet (HGD) compared to the low glycemic diet (LGD). The network of interactions between differentially expressed miRNAs (blue circles) and their target genes (yellow circles) of the high glycemic diet (HGD) compared to the low glycemic diet (LGD). miRNA targets were identified using Mienturnet database.

Supplemental Figure S4: Histogram of differentially expressed miRNA targets pathways in hippocampal microvessels with the high glycemic diet (HGD) when compared to the low glycemic diet (LGD). Significant cellular pathways ($p<0.05$) of differentially expressed miRNA target genes in hippocampus microvessels from the high glycemic diet (HGD) compared to low

glycemic diet (LGD). KEGG, WikiPathways, and Biocarta were identified using Genetrial2 online database. The data are shown for three biological replicates for each dietary group.

Supplemental Figure S5: Target gene networks of differentially expressed LncRNAs in hippocampal microvessels of female mice fed with the high glycemic diet (HGD) compared to the low glycemic diet (LGD). The network of interactions between differentially expressed lncRNAs (green triangles) and their target genes (grey circles) of the high glycemic diet (HGD) compared to the low glycemic diet (LGD). LncRNA targets were identified using LncRRIsearch and Rtools CBRC databases.

Supplemental Figure S6: Histogram of differentially expressed lncRNA targets pathways in hippocampal microvessels with the high glycemic diet (HGD) when compared to the low glycemic diet (LGD). Significant cellular pathways ($p<0.05$) of differentially expressed lncRNA target genes in hippocampus microvessels from the high glycemic diet (HGD) compared to low glycemic diet (LGD). The data are shown for three biological replicates for each dietary group. KEGG, and Biocarta were identified using Genetrial2 online database.

Supplemental Figure S7: Histogram of protein coding differentially expressed genes pathways in hippocampal microvessels for the low glycemic diet (LGD) with soluble epoxide hydrolase inhibitor (sEHI) compared to without sEHI treatment. Significant cellular pathways ($p<0.05$) of differentially expressed protein coding genes in hippocampus microvessels from the low glycemic diet (LGD) with soluble epoxide hydrolase inhibitor (sEHI) compared to without sEHI treatment. The data are shown for three biological replicates for each dietary group. KEGG and WikiPathways were identified using Genetrial2 online database.

Supplemental Figure S8: Target gene networks of differentially expressed transcription factors (TFs) in hippocampal microvessels for the low glycemic diet (LGD) with and without soluble epoxide hydrolase inhibitor (sEHI). The network of interactions between differentially expressed transcription factors (TFs, olive green) and their target genes (green) of the low glycemic diet (LGD) with and without soluble epoxide hydrolase inhibitor (sEHI). TFs and their targets were identified using Enrichr database.

Supplemental Figure S9: Target gene networks of differentially expressed miRNAs in hippocampal microvessels for the low glycemic diet (LGD) with and without soluble epoxide hydrolase inhibitor (sEHI). The network of interactions between differentially expressed miRNAs (blue circles) and their target genes (yellow circles) of the low glycemic diet (LGD) with soluble epoxide hydrolase inhibitor (sEHI) when compared to LGD without inhibitor. miRNA targets were identified using Mienturnet database.

Supplemental Figure S10: Histogram of differentially expressed miRNA targets pathways in hippocampal microvessels for the low glycemic diet (LGD) with soluble epoxide hydrolase inhibitor (sEHI) compared to without sEHI treatment. Significant cellular pathways ($p<0.05$) of differentially expressed miRNA target genes in hippocampus microvessels of the low glycemic diet (LGD) with soluble epoxide hydrolase inhibitor (sEHI) compared to without sEHI treatment. The data are shown for three biological replicates for each dietary group. Biocarta, KEGG and WikiPathways were identified using Genetrial2 online database.

Supplemental Figure S11: Target gene networks of differentially expressed LncRNAs in hippocampal microvessels for the low glycemic diet (LGD) with and without soluble epoxide hydrolase inhibitor (sEHI). The network of interactions between differentially expressed lncRNAs (black boxes) and their target genes (blue circles) of the low glycemic diet (LGD) with

soluble epoxide hydrolase inhibitor (sEHI) when compared to LGD without inhibitor. LncRNA targets were identified using LncRRIsearch and Rtools CBRC databases.

Supplemental Figure S12: Histogram of differentially expressed lncRNA targets pathways in hippocampal microvessels for the low glycemic diet (LGD) with soluble epoxide hydrolase inhibitor (sEHI) compared to without sEHI treatment. Significant cellular pathways ($p<0.05$) of differentially expressed lncRNA target genes in hippocampus microvessels of the low glycemic diet (LGD) with soluble epoxide hydrolase inhibitor (sEHI) compared to without sEHI treatment. The data are shown for three biological replicates for each dietary group. Biocarta, KEGG and WikiPathways were identified using Genetrial2 online database.

Table S1. Differentially expressed genes for the high glycemic diet (HGD) compared to low glycemic diet (LGD).

Gene Symbol	Description	Fold Change	P-value
		-3.43	1.19E-06
		-2.69	5.00E-06
Gprin3	GPRIN family member 3	-2.67	6.59E-06
Gm15167	predicted gene 15167 [Source:MGI Symbol;Acc:MGI:3705805]	-2.75	2.47E-05
Gm19852	PREDICTED: predicted gene, 19852 (Gm19852), miscRNA.	-3.58	3.25E-05
		-2.1	4.33E-05
Gm14429	predicted gene 14429 [Source:MGI Symbol;Acc:MGI:3649510]	-3.02	5.67E-05
Wdr48	WD repeat domain 48	-2.18	6.74E-05
Gm23416	predicted gene, 23416 [Source:MGI Symbol;Acc:MGI:5453193]	-2.91	7.73E-05
		-2.47	8.36E-05
		-2.81	0.0001
Thoc1	THO complex 1	-2.43	0.0001
Larp4b	La ribonucleoprotein domain family, member 4B	-2.03	0.0001
Gtf3c6	general transcription factor IIIC, polypeptide 6, alpha	-4.99	0.0002
		-5.06	0.0002
		-3.08	0.0002
		-4.28	0.0002
		-4.28	0.0002
Vps41	vacuolar protein sorting 41 (yeast)	-2.1	0.0002
Alg11	asparagine-linked glycosylation 11 (alpha-1,2-mannosyltransferase)	-2.57	0.0002
Gm25529	predicted gene, 25529 [Source:MGI Symbol;Acc:MGI:5455306]	-3.92	0.0002
Fnta	farnesyltransferase, CAAX box, alpha	-2.18	0.0003
		-2.1	0.0003
Gm23989	predicted gene, 23989 [Source:MGI Symbol;Acc:MGI:5453766]	-5.85	0.0003
Gm14670	predicted gene 14670 [Source:MGI Symbol;Acc:MGI:3705370]	2.35	0.0003
Erdr1	erythroid differentiation regulator 1	-2.97	0.0003
		-2.38	0.0003
4933433G08Rik	RIKEN cDNA 4933433G08 gene; PREDICTED: RIKEN cDNA 4933433G08 gene (4933433G08Rik), miscRNA.	2.14	0.0003
Slc35b1	solute carrier family 35, member B1	-2.28	0.0003
		-2.38	0.0003
		-2.1	0.0004
Zfp442	zinc finger protein 442 (Zfp442), mRNA.; zinc finger protein 442	-2.32	0.0004
		-2.25	0.0005
		-2.19	0.0005

Gm26050	predicted gene, 26050 [Source:MGI Symbol;Acc:MGI:5455827]	-4.63	0.0005
Rxrg	retinoid X receptor gamma	-2.15	0.0005
Papola	poly (A) polymerase alpha; Synthetic construct Mus musculus clone IMAGE:100062187, MGC:190402 poly (A) polymerase alpha (Papola) mRNA, encodes complete protein.	-2.4	0.0005
Gm22117	predicted gene, 22117 [Source:MGI Symbol;Acc:MGI:5451894]	-5.45	0.0005
Babam1	BRISC and BRCA1 A complex member 1	-2.08	0.0005
Tspyl2	TSPY-like 2	-4.09	0.0006
Gm9795	predicted pseudogene 9795 [Source:MGI Symbol;Acc:MGI:3704361]	-2.5	0.0006
		-2.31	0.0006
Gm15446	predicted gene 15446	-2.33	0.0007
		-3.09	0.0007
		-2.68	0.0007
Gm13803	predicted gene 13803	-2.84	0.0007
Atp13a5	ATPase type 13A5	-2.16	0.0008
		-2.85	0.0008
Cp	ceruloplasmin	-3.67	0.0008
		-3.46	0.0009
Gm25041	predicted gene, 25041 [Source:MGI Symbol;Acc:MGI:5454818]	-2.73	0.0009
Gm6758	predicted gene 6758 [Source:MGI Symbol;Acc:MGI:3643216]	-3.06	0.0009
Mbip	MAP3K12 binding inhibitory protein 1	-2.31	0.0009
Gm8991	predicted pseudogene 8991 [Source:MGI Symbol;Acc:MGI:3644227]	-5.19	0.001
Gm12458	predicted gene 12458 [Source:MGI Symbol;Acc:MGI:3650856]	-2.01	0.001
N4bp2l2	NEDD4 binding protein 2-like 2	-2.23	0.001
Nostrin	nitric oxide synthase trafficker	-2.66	0.0011
Gm24132	predicted gene, 24132 [Source:MGI Symbol;Acc:MGI:5453909]	-2.01	0.0011
Gm25559	predicted gene, 25559 [Source:MGI Symbol;Acc:MGI:5455336]	-27.4	0.0011
Hnrnpa1; Gm5643; Gm10052	heterogeneous nuclear ribonucleoprotein A1; predicted gene 5643 (Gm5643), non-coding RNA.; heterogeneous nuclear ribonucleoprotein A1 pseudogene; predicted pseudogene 10052 (Gm10052), non-coding RNA.; heterogeneous nuclear ribonucleoprotein A1 (Hnrnpa1), transcript variant 2, mRNA.; heterogeneous nuclear ribonucleoprotein A1 (Hnrnpa1), transcript variant 1, mRNA.	-2.23	0.0011
Gm24056	predicted gene, 24056 [Source:MGI Symbol;Acc:MGI:5453833]	-5.22	0.0012
Supt4a	suppressor of Ty 4A	-3.56	0.0012
Otud6b	OTU domain containing 6B	-3.11	0.0012

Mir297b; Mir297a-3; Mir297a-4	microRNA 297b; microRNA 297a-3; microRNA 297a-4	-7.29	0.0013
Pde2a	phosphodiesterase 2A, cGMP-stimulated	-2.26	0.0014
Chmp5	charged multivesicular body protein 5	-2.4	0.0014
Mir494	microRNA 494	-2.77	0.0014
Gm24646	predicted gene, 24646 [Source:MGI Symbol;Acc:MGI:5454423]	-4.35	0.0014
Gm25325	predicted gene, 25325 [Source:MGI Symbol;Acc:MGI:5455102]	-2.36	0.0014
		-2.39	0.0014
Magi2	membrane associated guanylate kinase, WW and PDZ domain containing 2	2.05	0.0015
Smim11	small integral membrane protein 11	-2.29	0.0015
		2.71	0.0015
		2.93	0.0015
		-5.57	0.0015
9330159F19Rik	RIKEN cDNA 9330159F19 gene	-2.93	0.0015
Mir667	microRNA 667	-2.89	0.0016
		-2.24	0.0016
Got2	glutamatic-oxaloacetic transaminase 2, mitochondrial; glutamate oxaloacetate transaminase 2, mitochondrial	-2.14	0.0017
		2.07	0.0017
Gm22734	predicted gene, 22734	-3.15	0.0017
Gm25249	predicted gene, 25249 [Source:MGI Symbol;Acc:MGI:5455026]	-2.27	0.0018
Abhd2	abhydrolase domain containing 2	-2.51	0.0019
Gm16261	predicted gene 16261 [Source:MGI Symbol;Acc:MGI:3826544]	-2.77	0.0019
		-4.61	0.0019
		-2.37	0.002
		2.09	0.0021
Gm25506	predicted gene, 25506 [Source:MGI Symbol;Acc:MGI:5455283]	-2.9	0.0022
		-2.4	0.0023
		2.18	0.0023
AF357426	snoRNA AF357426; snoRNA AF357426 (AF357426), small nucleolar RNA.	14.49	0.0024
Chmp7	charged multivesicular body protein 7	-2.03	0.0024
Hacd2	3-hydroxyacyl-CoA dehydratase 2	-2.34	0.0024
Gm23542	predicted gene, 23542 [Source:MGI Symbol;Acc:MGI:5453319]	-3.08	0.0025
Car2	carbonic anhydrase 2	-2.01	0.0025
Mir467h	microRNA 467h [Source:MGI Symbol;Acc:MGI:3783381]	-4.33	0.0025
Mir7059	microRNA 7059	-2.69	0.0026
Gm26195	predicted gene, 26195 [Source:MGI Symbol;Acc:MGI:5455972]	-3.52	0.0026

		-2.17	0.0027
Gm24834	predicted gene, 24834	-9.97	0.0027
		-2.24	0.0029
		-6.1	0.0029
		-2.05	0.0029
		-2.25	0.003
Gm22030	predicted gene, 22030 [Source:MGI Symbol;Acc:MGI:5451807]	-12.67	0.003
Gvin1; Gm4070	GTPase, very large interferon inducible 1; predicted gene 4070 (Gm4070), transcript variant 1, mRNA.; predicted gene 4070 (Gm4070), transcript variant 2, mRNA.; GTPase, very large interferon inducible 1 (Gvin1), transcript variant 1, mRNA.; Synthetic construct Mus musculus clone IMAGE:100069525, MGC:199427 GTPase, very large interferon inducible 1 (Gvin1) mRNA, encodes complete protein.	-2.02	0.0031
		-2.47	0.0031
Gm25128	predicted gene, 25128 [Source:MGI Symbol;Acc:MGI:5454905]	-15.76	0.0031
		-14.83	0.0032
Fnbp1l; Mir7657	formin binding protein 1-like; microRNA 7657	-2.83	0.0032
Mir6915	microRNA 6915	3.41	0.0033
		-3.34	0.0033
		-3.08	0.0034
LOC100861894	PREDICTED: uncharacterized LOC100861894 (LOC100861894), miscRNA.	-2.16	0.0034
Ociad1	OCIA domain containing 1	-2.05	0.0035
		-2.35	0.0036
		-2.35	0.0036
		-2.35	0.0036
		-2.35	0.0036
Lrrc4c	leucine rich repeat containing 4C	-2.93	0.0037
Arl5a	ADP-ribosylation factor-like 5A	-2.84	0.0037
		-2.14	0.0037
Mir669m-1	microRNA 669m-1	-3.17	0.0038
		-3.85	0.0038
Gm22923	predicted gene, 22923 [Source:MGI Symbol;Acc:MGI:5452700]	-2.29	0.0038
		-2.09	0.0039
		-3.51	0.0039
		-3.23	0.0039
		-3.92	0.0039
Gm23637	predicted gene, 23637 [Source:MGI Symbol;Acc:MGI:5453414]	-5.32	0.004
Gm26202	predicted gene, 26202 [Source:MGI Symbol;Acc:MGI:5455979]	-11.13	0.004

Pcna-ps2	proliferating cell nuclear antigen pseudogene 2 [Source:MGI Symbol;Acc:MGI:97505]	3.54	0.0041
Agfg1	ArfGAP with FG repeats 1	-2.12	0.0041
Pten	phosphatase and tensin homolog	-3.4	0.0041
Olfr102; Olfr100	olfactory receptor 102; olfactory receptor 100	2.08	0.0042
Gm15352	predicted gene 15352 [Source:MGI Symbol;Acc:MGI:3641922]	-2.04	0.0042
Gm23190	predicted gene, 23190 [Source:MGI Symbol;Acc:MGI:5452967]	-22.94	0.0042
Mir669d	microRNA 669d [Source:MGI Symbol;Acc:MGI:3783382]	-2.8	0.0043
Ppp1cb	protein phosphatase 1, catalytic subunit, beta isoform; protein phosphatase 1, catalytic subunit, beta isoform (Ppp1cb), mRNA.	-3.88	0.0043
		-2.45	0.0044
Gm16363	predicted gene 16363 [Source:MGI Symbol;Acc:MGI:3840139]	-2.71	0.0044
Gm7381	predicted gene 7381 [Source:MGI Symbol;Acc:MGI:3643743]	-5.06	0.0045
		-2.89	0.0045
Gm23278	predicted gene, 23278 [Source:MGI Symbol;Acc:MGI:5453055]	-3.37	0.0046
Gm13161	predicted gene 13161 [Source:MGI Symbol;Acc:MGI:3650656]	-2.15	0.0046
Mir466j	microRNA 466j [Source:MGI Symbol;Acc:MGI:3783376]	-7.76	0.0048
		-2.05	0.0049
		-4.87	0.0049
Gm12960	predicted gene 12960	-3.18	0.0049
		-2.05	0.005
		-2.07	0.005
Gm13168	predicted gene 13168 [Source:MGI Symbol;Acc:MGI:3650212]	-3.25	0.0051
Mir6391	microRNA 6391	-4.81	0.0052
Gm11843	predicted gene 11843 [Source:MGI Symbol;Acc:MGI:3651166]	-6.22	0.0052
Gm24148	predicted gene, 24148	-2.46	0.0052
Aplp2	amyloid beta (A4) precursor-like protein 2	-2.41	0.0053
Rps4l	ribosomal protein S4-like	2.47	0.0053
		-7.01	0.0053
Gm12238	predicted gene 12238	-2.16	0.0053
		-4.38	0.0054
Gm20438	predicted gene 20438 [Source:MGI Symbol;Acc:MGI:5141903]	2.48	0.0054
Gm14421	predicted gene 14421 [Source:MGI Symbol;Acc:MGI:3652254]	-2.8	0.0055
		-2.9	0.0055
		-6.51	0.0056
		2.09	0.0057

		-2.77	0.0058
		-2.03	0.0058
Tprgl	transformation related protein 63 regulated like	-2.03	0.0059
Gm22854	predicted gene, 22854 [Source:MGI Symbol;Acc:MGI:5452631]	-6.34	0.006
		-3.32	0.0061
		-2.24	0.0061
Snord45b	small nucleolar RNA, C/D box 45B	2.58	0.0061
LOC100862081	PREDICTED: uncharacterized LOC100862081 (LOC100862081), miscRNA.	-3.6	0.0061
Dram2	DNA-damage regulated autophagy modulator 2; VDNA-damage regulated autophagy modulator 2	-2.3	0.0062
Ppp1cc	protein phosphatase 1, catalytic subunit, gamma isoform	-2.11	0.0063
Gm27975	predicted gene, 27975 [Source:MGI Symbol;Acc:MGI:5531357]	2.06	0.0063
Gm22696	predicted gene, 22696 [Source:MGI Symbol;Acc:MGI:5452473]	-2.5	0.0064
Gm23990	predicted gene, 23990 [Source:MGI Symbol;Acc:MGI:5453767]	-2.39	0.0067
		-4.36	0.0067
Gm22048	predicted gene, 22048	-3.99	0.0067
Kidins220	kinase D-interacting substrate 220	-2.3	0.0067
Tra2b	transformer 2 beta homolog (Drosophila)	-2.23	0.0067
Ralgapa1	Ral GTPase activating protein, alpha subunit 1; Ral GTPase activating protein, alpha subunit 1 (Ralgapa1), transcript variant 3, mRNA.; Ral GTPase activating protein, alpha subunit 1 (Ralgapa1), transcript variant 1, mRNA.	-2.05	0.0068
Gm25224	predicted gene, 25224 [Source:MGI Symbol;Acc:MGI:5455001]	-3.2	0.0068
Gm14392	predicted gene 14392 [Source:MGI Symbol;Acc:MGI:3649574]; predicted gene 14392	-2.36	0.007
		-2.73	0.0072
Gm19609	PREDICTED: predicted gene, 19609 (Gm19609), miscRNA.	-2.21	0.0072
		-3.23	0.0072
		-2.16	0.0074
		-2.22	0.0076
5430403G16Rik	RIKEN cDNA 5430403G16 gene [Source:MGI Symbol;Acc:MGI:1924450]	-2.69	0.0077
Hspa4	heat shock protein 4	-2.39	0.0077
Gm13798	predicted gene 13798 [Source:MGI Symbol;Acc:MGI:3652036]	-2.04	0.0078
Gm6265	predicted pseudogene 6265	-2.69	0.0078
LOC100505062	PREDICTED: uncharacterized LOC100505062 (LOC100505062), miscRNA.	-3.18	0.0079
Gm6161	predicted gene 6161 [Source:MGI Symbol;Acc:MGI:3644104]	-2.35	0.0081

		-2.8	0.0083
Xdh	xanthine dehydrogenase; xanthine dehydrogenase (Xdh), mRNA.	-2.08	0.0083
Pdhb	pyruvate dehydrogenase (lipoamide) beta	-2.61	0.0084
Nacc1	nucleus accumbens associated 1, BEN and BTB (POZ) domain containing	-2.12	0.0085
		-2.34	0.0086
		-3.39	0.0086
		-2.02	0.0087
Gm4479	predicted gene 4479 [Source:MGI Symbol;Acc:MGI:3782663]	-2.59	0.0087
Tardbp	TAR DNA binding protein	-2.94	0.0088
		-2.09	0.0089
Mir669m-2	microRNA 669m-2	-5.89	0.0089
Mir669b	microRNA 669b	-4.57	0.009
Adrb2	adrenergic receptor, beta 2	-2.95	0.0091
Rnaset2a; Rnaset2b	ribonuclease T2A; ribonuclease T2B (Rnaset2b), mRNA.; ribonuclease T2B, mRNA (cDNA clone MGC:27782 IMAGE:3156549), complete cds.; ribonuclease T2B, mRNA (cDNA clone MGC:107248 IMAGE:6703307), complete cds.; ribonuclease T2B, mRNA (cDNA clone MGC:117616 IMAGE:30527517), complete cds.; ribonuclease T2B	-2.27	0.0091
		-4.18	0.0091
		-2.5	0.0092
Camk4	calcium/calmodulin-dependent protein kinase IV	-2.28	0.0093
Gm20253	PREDICTED: predicted gene, 20253 (Gm20253), miscRNA.	-2.03	0.0094
Mir487b	microRNA 487b	-2.81	0.0096
Mir466k	microRNA 466k [Source:MGI Symbol;Acc:MGI:3783377]	-7.06	0.0096
		2.2	0.0098
		-2.28	0.0098
Fam189a2	family with sequence similarity 189, member A2	-2.1	0.0098
LOC100861984	PREDICTED: uncharacterized LOC100861984 (LOC100861984), miscRNA.	-2.2	0.0098
Gm27243; RP23-35K5.2	predicted gene 27243 [Source:MGI Symbol;Acc:MGI:5521086]; novel transcript	-2.02	0.0099
		-4.18	0.0099
		-2.23	0.01
Impact	impact, RWD domain protein; imprinted and ancient	-2.93	0.0102
Gm22232	predicted gene, 22232 [Source:MGI Symbol;Acc:MGI:5452009]	-3.37	0.0103
Gm11295	predicted gene 11295 [Source:MGI Symbol;Acc:MGI:3651595]	-3.4	0.0103
Cdc42bpa	CDC42 binding protein kinase alpha	-2.42	0.0105
		2.17	0.0105
Gm26070	predicted gene, 26070 [Source:MGI Symbol;Acc:MGI:5455847]	-2.59	0.0106

Mir692-3; Ftl1; Ftl2-ps	microRNA 692-3; ferritin light polypeptide 1; ferritin light polypeptide 2, pseudogene; ferritin light chain 1 (Ftl1), mRNA.	-2.04	0.0107
		-7.95	0.0107
		-2.99	0.0109
		-4.72	0.011
		-8.79	0.0111
		-2.4	0.0112
		-2.15	0.0112
Mid1	midline 1	-2.13	0.0115
Med13	mediator complex subunit 13	-2.1	0.0116
Gm5093	predicted gene 5093	-2.05	0.0117
		-2.83	0.0118
Gm9242	predicted pseudogene 9242 [Source:MGI Symbol;Acc:MGI:3645172]	-2.4	0.0118
Gm24627	predicted gene, 24627 [Source:MGI Symbol;Acc:MGI:5454404]	-24.7	0.012
Mgp	matrix Gla protein	-6.86	0.012
		-3.05	0.012
		-5.23	0.0123
		-2.12	0.0123
Gm24510	predicted gene, 24510 [Source:MGI Symbol;Acc:MGI:5454287]	-3.62	0.0124
		-3.09	0.0124
		-3.59	0.0125
Gm24811	predicted gene, 24811 [Source:MGI Symbol;Acc:MGI:5454588]	-5.05	0.0125
Atp6ap2	ATPase, H+ transporting, lysosomal accessory protein 2	-2.32	0.0126
		-2.16	0.0128
		-2.27	0.0128
Rps15a-ps5	ribosomal protein S15A, pseudogene 5	-2.51	0.0131
		-2.32	0.0131
		-2.49	0.0132
		-2.14	0.0132
		-2.12	0.0135
		-2.72	0.0135
		-2.71	0.0136
		-3.48	0.0138
Gm22243	predicted gene, 22243 [Source:MGI Symbol;Acc:MGI:5452020]	-6.1	0.014
Slc22a8	solute carrier family 22 (organic anion transporter), member 8	-2.54	0.014
		-2.58	0.0143
		-7.23	0.0144
		-3.43	0.0147
Gm19491	PREDICTED: predicted gene, 19491 (Gm19491), miscRNA.	-2.47	0.0148
Gm11794	predicted gene 11794 [Source:MGI Symbol;Acc:MGI:3650853]	2.16	0.0148

		2.11	0.0149
		2.77	0.015
Gm23487	predicted gene, 23487	-2.61	0.0152
		2.42	0.0153
Psme2b	protease (prosome, macropain) activator subunit 2B	-2.95	0.0155
Gm24093	predicted gene, 24093	-3.5	0.0155
		4.38	0.0156
Gm27450	predicted gene, 27450 [Source:MGI Symbol;Acc:MGI:5530832]	-2.87	0.0158
Mir130c	microRNA 130c	-2.17	0.0159
Mir466h	microRNA 466h [Source:MGI Symbol;Acc:MGI:3718537]	-2.93	0.0161
Prkag1	protein kinase, AMP-activated, gamma 1 non-catalytic subunit	-2.03	0.0165
		-2.18	0.0166
		-2.39	0.0166
Mir466f-4	microRNA 466f-4 [Source:MGI Symbol;Acc:MGI:3783374]	-4.89	0.0167
		-2.13	0.0167
		-3.07	0.0167
Gm8822	predicted gene 8822	-2.54	0.0168
mt-Tr	mitochondrially encoded tRNA arginine [Source:MGI Symbol;Acc:MGI:102476]	-35.12	0.0168
		-2.64	0.0174
		-2.5	0.0174
Snord82	small nucleolar RNA, C/D box 82	-2.26	0.0174
Gm25410	predicted gene, 25410 [Source:MGI Symbol;Acc:MGI:5455187]	-2.06	0.0176
Gm23700	predicted gene, 23700 [Source:MGI Symbol;Acc:MGI:5453477]	-2.13	0.0177
Gm24557	predicted gene, 24557 [Source:MGI Symbol;Acc:MGI:5454334]	-7.02	0.0177
Gm26135	predicted gene, 26135 [Source:MGI Symbol;Acc:MGI:5455912]	-2.07	0.0178
Gm10088	predicted gene 10088	-3.8	0.0179
		-5.69	0.018
Gm2310	predicted gene 2310 [Source:MGI Symbol;Acc:MGI:3780481]	-2.06	0.018
Gm19645	PREDICTED: predicted gene, 19645 (Gm19645), miscRNA.	-2.43	0.0181
		-3.25	0.0181
Cdk19	cyclin-dependent kinase 19	-2.4	0.0181
Ndn	necdin	-2.48	0.0182
Zfp955b	zinc finger protein 955B	-2.53	0.0186
Atp2a2	ATPase, Ca++ transporting, cardiac muscle, slow twitch 2	-2.04	0.0187
Gm4540	predicted gene 4540 [Source:MGI Symbol;Acc:MGI:3782724]	-2.12	0.0187
Mir669n	microRNA 669n [Source:MGI Symbol;Acc:MGI:3837039]	-3.38	0.0189
		-2.08	0.0189
		-2.03	0.0189

1700020I14Rik	RIKEN cDNA 1700020I14 gene	-2.02	0.019
		-2.6	0.019
Gm12117	predicted gene 12117 [Source:MGI Symbol;Acc:MGI:3652019]	-2.18	0.019
Mir297a-2	microRNA 297a-2	-5.96	0.0191
Mir466f-3	microRNA 466f-3	-8.06	0.0192
Gm24608	predicted gene, 24608 [Source:MGI Symbol;Acc:MGI:5454385]	-3.04	0.0193
Gm12372	predicted gene 12372 [Source:MGI Symbol;Acc:MGI:3650289]	-2.75	0.0193
		-2.15	0.0195
Gm10974	predicted gene 10974 [Source:MGI Symbol;Acc:MGI:3779185]	-3.75	0.0195
		-7.38	0.0197
LOC100862086	PREDICTED: uncharacterized LOC100862086 (LOC100862086), miscRNA.	-4.77	0.02
Gm25567	predicted gene, 25567	-2.83	0.0201
		-2.11	0.0202
Gm25935	predicted gene, 25935 [Source:MGI Symbol;Acc:MGI:5455712]	-2.25	0.0202
Pts	6-pyruvoyl-tetrahydropterin synthase	-2.07	0.0202
Gm13240	predicted gene 13240 [Source:MGI Symbol;Acc:MGI:3650771]	-2.56	0.0202
Gm25071	predicted gene, 25071 [Source:MGI Symbol;Acc:MGI:5454848]	-6.84	0.0203
Rgs4	regulator of G-protein signaling 4	-4.34	0.0205
Gm24463	predicted gene, 24463 [Source:MGI Symbol;Acc:MGI:5454240]	-5.24	0.0205
		-2.04	0.0205
LOC100862198	PREDICTED: uncharacterized LOC100862198 (LOC100862198), miscRNA.	-2.57	0.0206
Bub3	budding uninhibited by benzimidazoles 3 homolog (S. cerevisiae)	-2.25	0.0207
		-2.27	0.021
		-7.89	0.0212
		-7.89	0.0212
		-7.89	0.0212
		-7.89	0.0212
Mir297c	microRNA 297c [Source:MGI Symbol;Acc:MGI:3718506]	-2.48	0.0216
Ddost	dolichyl-di-phosphooligosaccharide-protein glycotransferase	-2.51	0.0219
Gm24085	predicted gene, 24085 [Source:MGI Symbol;Acc:MGI:5453862]	-2.84	0.022
Gm23159	predicted gene, 23159 [Source:MGI Symbol;Acc:MGI:5452936]	-3.56	0.022
		-2.08	0.0222
		-9.73	0.0223
Rab28	RAB28, member RAS oncogene family	-2.02	0.0226

Gm22441	predicted gene, 22441	-2.58	0.0227
Rpl35a-ps2	ribosomal protein L35A, pseudogene 2	-4.53	0.0227
Gm25003	predicted gene, 25003	-2.41	0.0227
Mir669m-2	microRNA 669m-2 [Source:MGI Symbol;Acc:MGI:3837027]	-2.14	0.023
		-2.08	0.0231
		-2.41	0.0231
Gm23578	predicted gene, 23578 [Source:MGI Symbol;Acc:MGI:5453355]	-2.65	0.0231
Gm20349	PREDICTED: predicted gene, 20349 (Gm20349), miscRNA.	-3.5	0.0235
Gm26153	predicted gene, 26153 [Source:MGI Symbol;Acc:MGI:5455930]	-2.02	0.0236
		-2.95	0.0236
		-2.07	0.0238
Gm20417; RP24-458F14.3	predicted gene 20417 [Source:MGI Symbol;Acc:MGI:5141882]; novel transcript	-2.53	0.0239
		-2.22	0.0239
Gm26233	predicted gene, 26233 [Source:MGI Symbol;Acc:MGI:5456010]	-6.1	0.024
Vimp	VCP-interacting membrane protein	-2.37	0.024
		-3.63	0.024
Ly6c2	lymphocyte antigen 6 complex, locus C2	-2.26	0.024
Mir297-1	microRNA 297-1	-32.73	0.0241
Gm24026	predicted gene, 24026 [Source:MGI Symbol;Acc:MGI:5453803]	-6.21	0.0241
Rras	Harvey rat sarcoma oncogene, subgroup R	-2.31	0.0243
Gm25510	predicted gene, 25510 [Source:MGI Symbol;Acc:MGI:5455287]	-5.69	0.025
Gm7551	predicted gene 7551 [Source:MGI Symbol;Acc:MGI:3648704]	-3.21	0.0255
Gatm	glycine amidinotransferase (L-arginine:glycine amidinotransferase)	-2.08	0.0257
Gm5428	predicted gene 5428 [Source:MGI Symbol;Acc:MGI:3647789]; predicted gene 5428	-2.18	0.0257
		2.19	0.0258
Rpl35a-ps5	ribosomal protein L35A, pseudogene 5	-4.83	0.026
Snord61	small nucleolar RNA, C/D box 61	2.39	0.026
Hist1h2bc	histone cluster 1, H2bc	-3.41	0.0261
Apoo-ps	apolipoprotein O, pseudogene [Source:MGI Symbol;Acc:MGI:3649039]	-2.99	0.0261
Slco1c1	solute carrier organic anion transporter family, member 1c1	-2.12	0.0262
		-3.13	0.0266
Gm10254	predicted gene 10254 [Source:MGI Symbol;Acc:MGI:3708673]	-4.55	0.0269
Crh	corticotropin releasing hormone	-2.05	0.027
		2.43	0.0275

		-2.42	0.0277
		2.1	0.028
Gm26303	predicted gene, 26303 [Source:MGI Symbol;Acc:MGI:5456080]	-4.45	0.0281
Atp6v0e	ATPase, H+ transporting, lysosomal V0 subunit E	-2.06	0.0282
Gm11008; RP23-83O23.1	predicted gene 11008 [Source:MGI Symbol;Acc:MGI:3779224]; novel transcript	-4.68	0.0282
Gm6187; Gm10052	predicted gene 6187 [Source:MGI Symbol;Acc:MGI:3648954]; predicted pseudogene 10052 [Source:MGI Symbol;Acc:MGI:3704348]	-2.01	0.0285
		-4.07	0.0285
		-2.22	0.0288
		2.14	0.0288
Ivns1abp	influenza virus NS1A binding protein	-2.25	0.0289
Gm14681; LOC100861774	predicted gene 14681 [Source:MGI Symbol;Acc:MGI:3705734]; PREDICTED: uncharacterized LOC100861774 (LOC100861774), miscRNA.	-2.09	0.029
		-3.1	0.0291
		-4.66	0.0293
		2.06	0.0295
Map4k3	mitogen-activated protein kinase kinase kinase kinase 3; Synthetic construct Mus musculus clone IMAGE:100069494, MGC:199396 mitogen-activated protein kinase kinase kinase kinase 3 (Map4k3) mRNA, encodes complete protein.	-2.14	0.0296
Cdk13	cyclin-dependent kinase 13	-2.68	0.0296
Gm12643	predicted gene 12643 [Source:MGI Symbol;Acc:MGI:3649676]	-2.04	0.0298
LOC100861936	PREDICTED: uncharacterized LOC100861936 (LOC100861936), miscRNA.	-2.13	0.0299
		2.06	0.0299
Gm3625	predicted gene 3625	-4.86	0.0305
		-2.42	0.0308
Immp1l	IMP1 inner mitochondrial membrane peptidase-like (S. cerevisiae)	-8.25	0.0309
		-9.13	0.0311
		-2.11	0.0311
		2.03	0.0311
Gm11244	predicted gene 11244 [Source:MGI Symbol;Acc:MGI:3651883]	-7.73	0.0312
Gm13162	predicted pseudogene 13162 [Source:MGI Symbol;Acc:MGI:3701119]	-3.16	0.0312
Dynlt1b	dynein light chain Tctex-type 1B	-2.4	0.0314
		2.03	0.0314
Gm14335	predicted gene 14335 [Source:MGI Symbol;Acc:MGI:3801741]	-2.32	0.032
		-2.75	0.032

Gm6397	predicted gene 6397 [Source:MGI Symbol;Acc:MGI:3648542]	-2.32	0.0321
Erc2	ELKS/RAB6-interacting/CAST family member 2	-2.18	0.0323
Gm19474	PREDICTED: predicted gene, 19474, transcript variant 2 (Gm19474), miscRNA.	-4.11	0.0325
Gm26302	predicted gene, 26302 [Source:MGI Symbol;Acc:MGI:5456079]	-2.42	0.0325
		-2.6	0.0326
Anapc10	anaphase promoting complex subunit 10	-2.62	0.033
Habp4	hyaluronic acid binding protein 4	-2.04	0.0331
Mir1950	microRNA 1950	-2.14	0.0333
		-3.46	0.0333
		2.08	0.0336
Acbd6	acyl-Coenzyme A binding domain containing 6	-2.17	0.0337
Gm14308; Gm14430; Gm14434; 0610010B08Rik; Gm4724; Gm11007	predicted gene 14308 (Gm14308), mRNA.; predicted gene 14430 (Gm14430), mRNA.; predicted gene 14434 (Gm14434), mRNA.; RIKEN cDNA 0610010B08 gene (0610010B08Rik), mRNA.; predicted gene 4724 (Gm4724), mRNA.; predicted gene 11007; RIKEN cDNA 0610010B08 gene	-2.04	0.0338
		-2.88	0.0339
Gm23482	predicted gene, 23482 [Source:MGI Symbol;Acc:MGI:5453259]	2.5	0.034
Gm26199	predicted gene, 26199	-3.55	0.0342
		-3.17	0.0344
Gm23334	predicted gene, 23334 [Source:MGI Symbol;Acc:MGI:5453111]	-9.69	0.0346
Gm23617	predicted gene, 23617 [Source:MGI Symbol;Acc:MGI:5453394]	-2.12	0.0346
		-2.87	0.0349
		2.09	0.0349
Gm22403	predicted gene, 22403 [Source:MGI Symbol;Acc:MGI:5452180]	-2.54	0.0351
		2.11	0.0354
Sumo1	small ubiquitin-like modifier 1; SMT3 suppressor of mif two 3 homolog 1 (yeast)	-2.57	0.0356
Gm23881	predicted gene, 23881 [Source:MGI Symbol;Acc:MGI:5453658]	-9.6	0.0358
Gm5269	predicted gene 5269 [Source:MGI Symbol;Acc:MGI:3646793]	-2.01	0.0359
Fabp7	fatty acid binding protein 7, brain	-2.58	0.0359
Hnrnpa3; Gm6793	heterogeneous nuclear ribonucleoprotein A3; heterogeneous nuclear ribonucleoprotein A3 pseudogene	-2.67	0.0359
		-2.18	0.0362
Gm10175	predicted gene 10175 [Source:MGI Symbol;Acc:MGI:3704287]	-2.17	0.0364
		-2.22	0.0366

		-2.76	0.0371
Hsp90b1	heat shock protein 90, beta (Grp94), member 1	-2.09	0.0373
		-2.03	0.0374
Eogt	EGF domain-specific O-linked N-acetylglucosamine (GlcNAc) transferase	-2	0.0377
Gm23566	predicted gene, 23566 [Source:MGI Symbol;Acc:MGI:5453343]	-4.24	0.0378
Tomm20	translocase of outer mitochondrial membrane 20 homolog (yeast)	-2.18	0.0378
Mir467c	microRNA 467c	-12.36	0.0378
Atp6v1c1	ATPase, H+ transporting, lysosomal V1 subunit C1	-2.24	0.0378
		-2.53	0.0379
Gm23086	predicted gene, 23086 [Source:MGI Symbol;Acc:MGI:5452863]	-2.43	0.0382
		5.85	0.0385
Gm14412	predicted gene 14412 [Source:MGI Symbol;Acc:MGI:3652251]	-2.13	0.0387
Gm26397	predicted gene, 26397 [Source:MGI Symbol;Acc:MGI:5456174]	-2.21	0.0389
Gm23051	predicted gene, 23051	-4.74	0.039
		-3.07	0.0397
		-2.27	0.0398
		-2.48	0.0399
Arxes2	adipocyte-related X-chromosome expressed sequence 2	-2.89	0.04
		-2.62	0.04
Snora75	small nucleolar RNA, H/ACA box 75	2.03	0.0402
		-6.6	0.0402
		-2.11	0.0402
Gm6274	predicted gene 6274 [Source:MGI Symbol;Acc:MGI:3645367]	-2.11	0.0402
Ppp1r12a	protein phosphatase 1, regulatory (inhibitor) subunit 12A	-2.19	0.0403
Gm6304	predicted gene 6304 [Source:MGI Symbol;Acc:MGI:3647929]	-2.26	0.0406
Gm16199	predicted gene 16199 [Source:MGI Symbol;Acc:MGI:3801797]	-5.91	0.0408
Gm2026	predicted gene 2026 [Source:MGI Symbol;Acc:MGI:3780195]; predicted gene 2026	-2.26	0.0409
Gstm1	glutathione S-transferase, mu 1	-2.24	0.0413
Gm26097	predicted gene, 26097 [Source:MGI Symbol;Acc:MGI:5455874]	-10.12	0.0416
Gm26052	predicted gene, 26052 [Source:MGI Symbol;Acc:MGI:5455829]	-2.39	0.0419
Gm10243	predicted gene 10243	-2.32	0.0421
		-2.02	0.0424
		-3.73	0.0425
Gm27038	predicted gene, 27038 [Source:MGI Symbol;Acc:MGI:5504153]	-2.6	0.0425

Gm24588	predicted gene, 24588 [Source:MGI Symbol;Acc:MGI:5454365]	-2.3	0.0427
		-2.31	0.0427
Gm25544	predicted gene, 25544 [Source:MGI Symbol;Acc:MGI:5455321]	-4.03	0.0428
		-3.43	0.0428
		-2.21	0.0428
Gm13378	predicted gene 13378 [Source:MGI Symbol;Acc:MGI:3650302]	-4.2	0.0429
Gm24586	predicted gene, 24586 [Source:MGI Symbol;Acc:MGI:5454363]	-5.36	0.0431
Gm23677	predicted gene, 23677 [Source:MGI Symbol;Acc:MGI:5453454]	-2.51	0.0433
A130040M12Rik	RIKEN cDNA A130040M12 gene (A130040M12Rik), non-coding RNA.	-2.48	0.0438
D8Ertd738e	DNA segment, Chr 8, ERATO Doi 738, expressed	-2.03	0.0438
Gm25361	predicted gene, 25361 [Source:MGI Symbol;Acc:MGI:5455138]	-2.22	0.0439
Mir466o	microRNA 466o	-2.57	0.0444
Mir466f-1	microRNA 466f-1 [Source:MGI Symbol;Acc:MGI:3718533]	-2.71	0.0447
		-2.34	0.0448
D10Jhu81e	DNA segment, Chr 10, Johns Hopkins University 81 expressed	-2.25	0.0452
Gm19660	PREDICTED: predicted gene, 19660, transcript variant 1 (Gm19660), miscRNA.	-2.83	0.0452
Abcb1a	ATP-binding cassette, sub-family B (MDR/TAP), member 1A	-3.2	0.0454
Gm23237	predicted gene, 23237 [Source:MGI Symbol;Acc:MGI:5453014]	-3.18	0.0454
Gm25000	predicted gene, 25000 [Source:MGI Symbol;Acc:MGI:5454777]	-3.28	0.0455
Gm15758; AC109200.1	predicted gene 15758 [Source:MGI Symbol;Acc:MGI:3783201]; Novel transcript	-2.53	0.0456
		-2.19	0.0459
Gm23415	predicted gene, 23415 [Source:MGI Symbol;Acc:MGI:5453192]	-4.68	0.0459
Gm22698	predicted gene, 22698 [Source:MGI Symbol;Acc:MGI:5452475]	-2.2	0.0468
Ly6a	lymphocyte antigen 6 complex, locus A; lymphocyte antigen 6 complex, locus A (Ly6a), mRNA.	-2.75	0.0469
Supt4b	predicted gene 3258	-2.05	0.0471
Kcnab1	potassium voltage-gated channel, shaker-related subfamily, beta member 1	-2.61	0.048
		2.2	0.0486
Gm2962	predicted pseudogene 2962 [Source:MGI Symbol;Acc:MGI:3781140]	-6.04	0.0488
		2.03	0.0489
Pinx1	PIN2/TERF1 interacting, telomerase inhibitor 1	-3.63	0.049

Gm10224	predicted pseudogene 10224	-2.88	0.0491
		-2.04	0.0494
		-3.89	0.0494
		-2.21	0.0495
LOC100862094	PREDICTED: uncharacterized LOC100862094 (LOC100862094), miscRNA.	-3.7	0.0496
Itpka	inositol 1,4,5-trisphosphate 3-kinase A	-2.09	0.0496
1810022K09Rik	RIKEN cDNA 1810022K09 gene	-2.12	0.05

Table S2. Effect of the high glycemic diet (HGD) compared to the low glycemic diet (LGD) on the expression of microRNAs (miRNAs) in hippocampal microvessels.

Gene Symbol	Fold Change	P-value
Gm23416	-2.91	7.73E-05
Gm25529	-3.92	0.0002
Gm23989	-5.85	0.0003
Gm26050	-4.63	0.0005
Gm22117	-5.45	0.0005
Gm25041	-2.73	0.0009
Gm24132	-2.01	0.0011
Gm25559	-27.4	0.0011
Mir297b	-7.29	0.0013
Mir297a-3	-7.29	0.0013
Mir297a-4	-7.29	0.0013
Mir494	-2.77	0.0014
Gm25325	-2.36	0.0014
Mir667	-2.89	0.0016
Gm22734	-3.15	0.0017
Gm25249	-2.27	0.0018
Gm23542	-3.08	0.0025
Mir467h	-4.33	0.0025
Mir7059	-2.69	0.0026
Gm26195	-3.52	0.0026
Gm24834	-9.97	0.0027
Gm22030	-12.67	0.003
Mir6915	3.41	0.0033
Mir669m-1	-3.17	0.0038
Gm22923	-2.29	0.0038
Gm23637	-5.32	0.004
Gm23190	-22.94	0.0042
Mir669d	-2.8	0.0043
Mir466j	-7.76	0.0048
Mir6391	-4.81	0.0052
Gm22854	-6.34	0.006
Gm22696	-2.5	0.0064
Gm23990	-2.39	0.0067
Gm22048	-3.99	0.0067
Mir669m-2	-5.89	0.0089
Mir669b	-4.57	0.009
Mir487b	-2.81	0.0096
Mir466k	-7.06	0.0096
Gm22232	-3.37	0.0103
Gm24627	-24.7	0.012
Gm24510	-3.62	0.0124
Gm24811	-5.05	0.0125

Gm22243	-6.1	0.014
Gm23487	-2.61	0.0152
Gm24093	-3.5	0.0155
Gm27450	-2.87	0.0158
Mir130c	-2.17	0.0159
Mir466h	-2.93	0.0161
Mir466f-4	-4.89	0.0167
Gm23700	-2.13	0.0177
Gm24557	-7.02	0.0177
Mir669n	-3.38	0.0189
Mir297a-2	-5.96	0.0191
Mir466f-3	-8.06	0.0192
Gm25567	-2.83	0.0201
Gm25071	-6.84	0.0203
Mir297c	-2.48	0.0216
Gm24085	-2.84	0.022
Gm23159	-3.56	0.022
Gm22441	-2.58	0.0227
Gm25003	-2.41	0.0227
Mir669m-2	-2.14	0.023
Gm23578	-2.65	0.0231
Gm26233	-6.1	0.024
Mir297-1	-32.73	0.0241
Gm24026	-6.21	0.0241
Gm25510	-5.69	0.025
Gm26303	-4.45	0.0281
Gm26302	-2.42	0.0325
Mir1950	-2.14	0.0333
Gm26199	-3.55	0.0342
Gm23334	-9.69	0.0346
Gm23617	-2.12	0.0346
Gm22403	-2.54	0.0351
Gm23881	-9.6	0.0358
Gm23566	-4.24	0.0378
Mir467c	-12.36	0.0378
Gm23086	-2.43	0.0382
Gm23051	-4.74	0.039
Gm26052	-2.39	0.0419
Gm24588	-2.3	0.0427
Gm24586	-5.36	0.0431
Gm25361	-2.22	0.0439
Mir466o	-2.57	0.0444
Mir466f-1	-2.71	0.0447
Gm23237	-3.18	0.0454
Gm25000	-3.28	0.0455

Gm23415	-4.68	0.0459
Gm22698	-2.2	0.0468
Mir692-3	0.7341	-2.04

Table S3. Effect of the high glycemic diet (HGD) compared to the low glycemic diet (LGD) on the expression of long noncoding RNAs (lncRNAs) in hippocampal microvessels.

Gene Symbol	Fold Change	P-value
Gm19852	-3.58	3.3E-05
4933433G08Rik	2.14	0.0003
LOC100861894	-2.16	0.0034
LOC100862081	-3.6	0.0061
Gm19609	-2.21	0.0072
LOC100505062	-3.18	0.0079
Gm20253	-2.03	0.0094
LOC100861984	-2.2	0.0098
Gm20349	-3.5	0.0235
Mid1	-2.13	0.0115
Gm19491	-2.47	0.0148
Gm19645	-2.43	0.0181
1700020I14Rik	-2.02	0.019
LOC100862086	-4.77	0.02
LOC100862198	-2.57	0.0206
Gm20417	-2.53	0.0239
RP24-458F14.3	-2.53	0.0239
Gm11008	-4.68	0.0282
RP23-83O23.1	-4.68	0.0282
LOC100861936	-2.13	0.0299
Gm19474	-4.11	0.0325
A130040M12Rik	-2.48	0.0438
Gm19660	-2.83	0.0452
LOC100862094	-3.7	0.0496
AC109200.1	-2.53	0.0456
Gm15758	-2.53	0.0456
RP23-35K5.2	-2.02	0.0099
Gm27243	-2.02	0.0099

Table S4. Effect of the high glycemic diet (HGD) compared to the low glycemic diet (LGD) on the expression of small nucleolar RNAs (snoRNAs) in hippocampal microvessels.

Gene Symbol	Fold Change	P-value
AF357426	14.49	0.0024
Gm24148	-2.46	0.0052
Gm12238	-2.16	0.0053
Gm25506	-2.9	0.0022
Gm25128	-15.76	0.0031
Gm26202	-11.13	0.004
Gm23278	-3.37	0.0046
Snord45b	2.58	0.0061
Gm25224	-3.2	0.0068
Gm26070	-2.59	0.0106
Snord82	-2.26	0.0174
Gm25410	-2.06	0.0176
Gm26135	-2.07	0.0178
Gm24608	-3.04	0.0193
Gm24463	-5.24	0.0205
Gm26153	-2.02	0.0236
Snord61	2.39	0.026
Gm23482	2.5	0.034
Gm26397	-2.21	0.0389
Snora75	2.03	0.0402
Gm26097	-10.12	0.0416
Gm25544	-4.03	0.0428

Table S5. Mean signal intensities of diet and inhibitor groups.

Gene Symbol	HGD	HGD+sEHI	LGD	LGD+sEHI
Gprin3	4.48	4.51	5.9	4.58
Gm14429	5.62	4.93	7.22	4.45
Erdr1	5.87	5.43	7.44	4.82
Gm19852	4.95	4.97	6.79	4.62
Gm14745	3.93	4.21	4.05	4.8
H2afv	5.88	5.65	6.63	5.46
Gm23553	4.76	5.12	5.71	4.59
Larp4b	5.21	5.12	6.23	4.99
Nedd8	7.93	7.78	8.42	7.19
Cnot2	5.63	5.36	6.31	5.58
Med28	4.61	4.63	5.31	4.48
Gm21887	4.92	4.58	5.48	4.26
Gm15167	4.22	4.92	5.67	4.63
Wdr48	5.2	5.17	6.33	5.22
Gm6415	4.82	4.68	4.54	3.86
Gm2799	4.16	5.04	3.9	3.72
Gm5346	3.85	3.93	3.54	4.55
Ak3	4.8	4.67	5.56	4.91
Slc16a1	4.05	4.19	4.7	4.24
Gm23989	5.9	5.85	8.45	5.44
Gm14894	5.32	5.3	4.48	5.59
Gtf3c6	5.79	6.29	8.11	5.88
Cdc73	4.96	4.93	5.53	4.98
Gm26219	5.03	4.61	5.73	4.79
Gm19789	5.95	5.22	6.41	5.14
Gm24132	5.71	5.35	6.72	5.1
Mir6338	3.9	4.73	3.85	4.86
3300002I08Rik	4.68	4.67	5.39	4.63
Reps2	5.28	5.29	6.03	5.29
Gm25617	5.19	6.7	6	6.19
Gm19491	6.89	5.63	8.2	5.32
Fnta	4.79	4.52	5.92	4.38
Atp13a5	5.26	5.07	6.37	5.21
Gm26829	3.84	3.93	3.79	4.66
Gm25529	6.12	6.6	8.1	5.89
Gm22117	7.44	6.43	9.88	6.47
Thoc1	5.27	5.39	6.56	5.43
Olfr160	3.82	4.32	3.73	4.23
Trav6n-5	3.85	4.27	3.61	4.24
Rhox11-ps3	4.39	4.24	4.11	4.72
Gm23416	4.31	4.55	5.85	4.93
Gm9531	7.26	7.49	8.12	7.4

Vps41	5.13	5.21	6.19	5.14
Gm14733	6.22	6.17	6.38	5.73
Ktn1	5.1	5.45	5.9	5.22
Abhd4	5.77	4.91	5.34	4.79
Gm25694	4.35	4.59	4.15	4.8
Hnrnpa0	7.2	6.54	8.16	6.53
Gm8991	8.45	8.05	10.83	8.23
Gm28022	4.02	3.77	4.77	3.96
Mir1933	4.24	3.88	4.84	4.02
Gm23134	10.95	7.49	11.48	7.21
Gm23253	6.04	4.72	5.65	5.36
Thrb	4.69	5.07	5.64	4.93
Atg4a	4.19	4.47	4.89	4.45
Lrrc8dos	4.4	3.71	4.1	4.55
Ap3m2	4.76	4.88	5.46	4.67
Fars2	4.86	4.82	5.41	4.77
Gm16579	3.7	4.08	3.55	4.31
Papola	6.54	6.27	7.8	5.92
Rxrg	4.58	4.62	5.68	4.47
Gm12192	4.37	4.77	4.13	4.92
Gm22702	5.95	5.87	6.5	5.2
Gm17268	12.53	13.22	13	14.46
Pten	5.15	4.79	6.92	5.08
Gm2085	4.62	4.6	4.41	4.93
Olfr1419	3.54	3.96	3.51	4.39
4930473D10Rik	4.21	4.39	4.04	4.61
Gm25325	5.13	5.13	6.37	5.01
Mbip	5.22	5.67	6.43	5.09
Gm15446	5.46	5.9	6.69	5.41
Gng11	5.65	4.86	6.31	5.47
3110006O06Rik	3.9	3.92	3.59	4.51
LOC100861830	3.8	4.37	3.98	3.95
Gm24213	4.44	4.21	4.17	5.07
Gm24930	5.93	6.03	5.21	6.04
Trav10n	4.18	4.3	3.8	4.42
Gm25716	3.86	4.03	3.76	4.48
Gm23571	4.45	5.13	4.94	4.71
LOC100862313	7.26	6.08	7.72	5.11
Gm13803	5.64	6	7.14	5.38
Myo1b	5	5.09	5.76	5.02
n-R5s179	3.92	4.42	4.1	4.91
Gm14849	3.87	4.51	3.89	3.61
Gm24373	5.46	6.46	5.99	5.83
Vrk1	4.72	4.63	5.27	4.62

Trav14d-2	4.27	4.91	4.46	4.2
Trav14n-2	4.27	4.91	4.46	4.2
Senp6	5.3	5.36	5.97	5.27
Alg11	4.24	4.71	5.6	4.41
Drd2	5.54	4.5	6.43	4.66
Gna13	4.96	5.02	5.81	4.82
Gm16494	5.96	5.6	5.57	5.09
Zfp442	4.6	4.68	5.81	4.75
Atrnl1	4.95	4.88	5.64	4.8
Ndufs6	7.15	7.3	7.55	6.83
Gm24831	4.22	4.6	3.66	4.35
Olfr1153	4.01	4.19	3.96	4.8
Vstm4	4.8	4.8	5.4	4.93
Gm13738	4.3	4.4	4	4.86
Gm14670	5.66	5.4	4.43	5.04
Matr3	6.04	6.07	6.64	5.33
Gm26050	5.16	5.81	7.37	4.73
Cp	4.71	4.9	6.58	5.11
Kpna1	4.64	4.67	5.06	4.64
4933433G08Rik	4.89	4.52	3.79	4.06
Gm10010	3.65	4.01	3.43	3.89
Olfr30	4.62	4.19	4.82	4.82
n-R5s213	6.21	6.51	6.29	6.97
Gm11192	4.49	4.51	5.03	4.17
Xbp1	5.3	4.94	5.42	4.59
Dhdds	4.85	4.89	5.27	4.8
Olfr343-ps1	4.14	4.1	4.07	4.62
Gm14882	3.83	4.71	3.95	4.34
Gm25559	4.82	4.78	9.6	5.7
Gm15226	3.86	4.45	3.96	4.08
Mir669d	5.61	5.77	7.09	5.04
Gm26208	4.31	4.91	4.33	4.26
Olfr757-ps1	4.16	3.89	3.73	4.34
LOC100861956	4.59	4.05	4.63	4.24
Fam168b	5.33	5.53	6.07	5.06
Gm5909	5.35	5	5.46	4.73
Mir669l	4.75	5.05	5.65	4.43
Gm23995	4.48	5.21	4.9	4.49
Gm26061	4.38	4.7	4.31	4.81
Kbtbd11	5.96	6.1	6.89	6.01
Ighv5-15	3.64	4.9	4.2	4.28
Magi2	5.07	4.23	4.04	4.79
Mir692-3	5.25	5.02	6.28	4.98
Gm22030	6.71	6.76	10.38	5.51

Fig4	4.5	4.54	5.2	4.64
Gm23955	4.17	4.05	3.93	4.46
Gm23851	4.19	4.84	3.98	4.62
Mir6344	6.1	6.23	7.06	6.23
Mir297b	4.92	5	7.79	5.72
Mir667	5.07	5.03	6.6	5.11
Slc35b1	4.96	5.36	6.15	4.98
Gm24078	5.14	5.31	6.11	5.14
l7Rn6	4.77	4.71	5.61	4.86
Ankrd50	5.15	5.24	5.66	5.16
Gm12953	4.49	5.26	4.83	4.54
Mir466d	6.41	6.62	7.09	5.28
Gm19595	9.46	6.17	10.37	6.59
LOC100861894	4.1	4.15	5.21	4.18
Gm14398	8.89	8.61	9.4	9.29
Eln	5.69	5.64	6.19	5.44
Timm21	4.62	5.22	5.58	4.82
Supt4a	5.16	5.5	6.99	5.05
1700023F02Rik	4.03	4.37	4.51	3.98
Gm648	3.84	4.3	3.75	3.84
Gm13665	7.25	7.17	7.22	6.3
Smim11	4.27	4.4	5.47	4.24
Gm22734	4.98	5.09	6.64	5.57
Rnu73b	3.95	4.41	3.62	4.44
Kidins220	6.16	5.99	7.36	5.59
4933400A11Rik	4.39	4.56	4.1	4.87
Gm11928	3.93	4.51	3.72	4.09
Mir1188	4.84	5.6	5.2	5.35
Nxf1	5.97	5.65	6.67	5.53
Tspyl2	6.58	7.02	8.62	6.99
Gm26765	4.32	4.41	4.09	3.66
Klc1	5.82	5.83	6.47	5.74
2310036O22Rik	5.86	5.6	6.24	4.9
Gm25747	8.71	8.93	9.2	8.75
Mettl5	4.8	4.46	5.19	4.53
Gm24793	3.78	4.02	3.66	4.16
Gm22509	4.39	5.24	4.51	4.1
Cyp2j7-ps2	4.33	4.23	4.34	4.99
Babam1	5.72	5.85	6.78	6.42
Otud6b	6.94	7.11	8.57	6.78
Myo6	4.73	4.75	5.2	4.85
Gm23210	4.22	4.48	3.88	4.55
Gm27255	3.77	4.06	3.82	4.53
Ndn	6.62	5.79	7.94	4.05

Dstyk	4.61	4.85	5.26	4.82
Vmn1r-ps54	4.18	4.95	4.24	4.53
Ddah1	5.97	5.35	6.78	5.67
Gm24916	4.85	4.96	4.01	4.48
Trav8d-2	3.65	4.06	3.64	3.62
Pard3bos1	3.82	4.19	3.73	4.18
Serping1	4.77	4.55	5.09	4.54
Larp7	5.59	5.35	6.39	5.28
Gm24283	3.75	3.79	3.7	4.19
Gm10775	3.96	4.13	3.48	3.99
Hipk1	5.15	5.21	5.96	5.24
Gm14616	4.4	4.7	4.15	4.85
2410015M20Rik	6.91	6.54	7.53	6.21
Gm16363	4.65	4.09	6.09	4.81
LOC100861951	4.33	4.16	4.68	3.8
Mir7223	4.62	4.48	4.1	5.43
Itlnb	4.66	4.46	4.49	5.26
Gm9742	5.08	4.82	4.92	4.55
Gm14431	6.17	5.61	6.82	5.82
Sp3	4.91	4.79	5.02	4.49
Gm5601	7.61	6.67	8.03	6.14
Lmbrd1	4.79	5.18	5.36	4.75
BC049762	3.8	3.94	3.61	4.12
N4bp2l2	5.13	5.61	6.29	5.33
Ptprtos	4.53	4.48	5.02	4.75
Nipbl	5.15	5.29	5.77	5.04
Gm7803	4.75	4.57	4.85	5.68
Gm6586	3.95	4.51	3.71	4.19
Gm23181	4.94	4.81	5.53	4.32
Ighv8-12	4.9	4.09	4.48	5.08
Gm6758	10.79	11.13	12.41	11.4
Gm8116	5.2	5.29	5.44	4.71
Defa26	4.72	4.4	4.27	4.13
Gm13483	4.3	4.44	4.01	4.53
Ei24	4.59	4.74	5.45	4.75
Magea1	4.21	4.01	3.91	4.55
Aff4	5.6	5.62	6.47	5.43
Gm21283	4.29	4.39	4.07	4.68
Gm24837	3.93	4.58	3.9	4.22
Gm14392	5.51	5.17	6.75	5.36
Uba5	4.8	5.05	5.41	4.81
Gm24056	6.13	6.52	8.52	6.26
Gm25354	4.81	4.4	4.1	4.87
LOC100862279	3.74	4.16	3.9	4.3

Gm19960	4.3	4.5	5.24	4.99
Gm10349	5.17	5.17	5.96	4.98
Gm18828	3.77	4.39	3.7	4.03
Prpf18	5.94	6.43	6.93	5.59
4930571K23Rik	3.99	4.41	3.89	4.61
Gm11655	4.16	4.52	4.04	4.72
Gm13062	4.37	4.46	4.29	4.95
4933428M09Rik	4.38	4.25	4.32	4.74
Gm15882	3.77	4.59	4	4.62
Gm15325	4.11	3.9	4.21	4.74
Mir344d-1	3.47	3.72	3.5	4.09
Gm11845	4.05	3.75	3.83	4.45
Ubr1	5.04	5.03	5.48	4.72
Foxd4	3.77	3.98	3.35	3.67
Nppc	3.81	4.02	3.44	3.66
Vmn1r11	4.31	4.47	3.94	5.27
Shtn1	4.93	5.07	5.45	4.89
Nalcn	4.83	5.07	5.39	5.03
Rplp1-ps1	5.82	5.83	6.63	5.74
Gm24747	4.38	4.53	4.47	5.24
Gm12960	8.03	7.98	9.7	8.36
Gm25533	4.14	4.28	5.08	4.25
Mtdh	5.48	5.79	6.1	5.64
Hamp2	5.29	5.16	5.57	5.14
Mrgpra9	4.4	4.73	3.96	4.3
Lrp6	4.78	4.89	5.3	4.77
Tas2r137	3.95	4.69	3.95	4.84
Mir6346	3.86	3.94	3.52	4.49
Mir6346	3.86	3.94	3.52	4.49
Gm23327	4.23	5.09	4.49	4.5
Gm14420	6.12	5.93	6.93	5.48
Gm15445	4.98	5.89	5.09	4.89
Gm12495	4.18	3.92	4.06	4.68
Tle4	5.29	5.07	5.81	4.99
Chmp5	5.22	5.56	6.49	5.29
Snord82	5.07	6.67	6.25	6.68
9130008F23Rik	3.89	4.12	3.85	4.53
Gm16261	4.51	4.94	5.99	4.51
Tcstv3	3.87	4.18	3.93	4.41
Gm22015	5.86	4.69	6.79	5
Serpinb9f	4	4.2	4.01	4.43
Olfr692	5.45	5.72	5.43	6.47
Gm26735	4	4.46	4.33	4.51
Olfr788	3.98	4.38	4.13	3.91

Usp31	4.72	5.18	5.37	5
Gm19684	4.22	4.25	4.24	4.95
Aamp	5.45	5.56	6.13	5.45
Styx	4.99	4.94	5.56	4.71
Hnrnpa1	7.07	7.38	8.23	7.27
Pde2a	6.13	6.06	7.31	6.25
Dnajc1	5.11	5.18	5.67	4.77
Mir692-2	5.93	5.03	6.57	5.44
Tspan13	5.21	5.6	6.05	5.65
Snrpd3	5.51	5.41	6.27	5.31
Got2	4.62	4.87	5.72	4.47
Eid2	5.08	4.62	4.67	4.54
4930555B11Rik	4.17	3.95	3.91	4.38
Gm9795	3.97	4.31	5.29	4.46
Egln2	4.73	5.15	5.03	4.82
LOC100861805	5.37	4.91	5.22	4.26
Gm25249	5.47	5.8	6.66	5.11
Gm5800	4.15	4.05	3.91	4.44
Gm25376	4.73	4.57	5.2	4.59
Gm13903	4.18	4.77	3.8	4.3
Vmn1r123	3.87	4.3	4.57	4.09
Mrgprb8	3.73	3.79	3.59	4.41
4930572K03Rik	4.3	4.31	3.99	3.94
Gm8858	5.54	5.03	6.32	4.85
Dnajc19	5.43	5.19	5.77	5.19
Mup2	4.42	4.55	4.3	5.01
Zfp595	4.21	4.24	4.65	4.32
Fundc2	5.64	5.19	5.83	4.9
Scgb1b20	4.71	4.77	5.37	5.08
Gm4784	4.73	5.28	4.97	5.73
Gm16420	4.21	4.19	4.23	4.9
Nostrin	4.88	5.39	6.29	5.23
Atp13a3	5.05	5.01	5.7	5.04
4930480E11Rik	3.89	4.87	3.58	4.72
Vmn1r46	3.92	3.99	3.59	4.35
2310043L19Rik	4.48	4.35	4.34	4.92
Mir494	6.06	6.38	7.53	5.91
Mrgprb13	4	4.72	3.89	4.02
Crygs	4.06	3.99	3.77	4.46
Napg	4.85	4.98	5.56	4.84
Idh3b	5.67	5.88	6.29	5.87
Tmed10	4.85	4.78	5.3	4.5
Zfyve9	4.58	4.72	5.18	4.82
Gm22005	3.82	4.11	3.78	4.42

Mir7076	6.05	6.68	6.68	6.46
Chmp7	4.61	4.66	5.63	4.41
Gm27975	5.38	4.89	4.34	5.47
9130011E15Rik	4.63	4.68	5.15	4.77
Gm7209	4.41	3.87	3.74	4
Insr	5.42	5.39	6.02	5.23
Krt39	4.09	4.36	3.93	4.5
Krtap1-5	3.96	3.8	4.08	4.71
Wnk1	5.94	5.68	6.56	5.51
Gm25485	3.83	4.4	3.48	3.82
Gm13161	4.3	5.12	5.4	4.62
Cldn34d	4.07	4.51	3.93	4.93
Ppp1r1b	6.3	5.77	6.4	5.37
Gm23975	5.12	5.8	4.92	5.55
Gm12255	5.69	5.19	5.26	4.99
Gm1604b	4.83	4.83	5.46	4.65
Gm2516	4.14	4.21	3.96	4.43
Stt3b	5.28	4.87	5.73	5.21
Gm15544	4.26	3.79	3.84	4.36
Gm26614	3.87	4.56	3.81	3.88
Gm16442	4.01	4.24	3.88	4.67
Gm24325	5.42	6.18	5.62	5.47
Olfr1186	3.99	4.67	3.99	4.63
Armc1	5.3	5.13	5.53	4.45
Gm16193	4.37	4.29	4.04	4.72
Gm26733	4.13	4.63	4.1	4.01
Fchsd2	5.04	4.77	5.34	4.82
Cir1	5.12	5.33	5.84	5.2
Mir669f	5.27	5.46	6.14	5.12
Gm3555	4.82	5.07	4.64	4.3
Apoa5	4.04	4.43	3.97	4.55
Pnrc2	5.28	5.38	6.13	5.35
Tra2b	5.31	5.6	6.46	5.19
Serpine2	5.7	5.54	6.21	5.04
Gm15178	4.7	4.44	4.44	4.97
Vti1b	6.78	6.26	7.43	5.91
Olfr1371	5.48	5.95	5.73	6.68
Gm11564	4.29	3.88	4.29	3.75
Snx14	5.01	5.62	5.39	5
Olfr183	3.75	4.34	3.76	4.51
Zeb1	6.13	5.33	6.88	5.5
Gm13914	4.03	4.33	4.24	4.68
LOC100861675	6.27	5.16	6.82	5.26
Car2	4.81	4.52	5.81	5.34

LOC100862094	5.84	5.26	7.73	5.33
Gm23060	5.44	5.17	5.94	5.3
Smek1	5.04	5.16	5.53	5.18
Ptx4	3.98	3.83	3.52	3.84
Terf2	4.95	4.66	5.42	4.84
Gm9972	4.08	4.14	4.33	5.29
Gm10282	5.88	4.94	6.28	4.78
Gm8151	4.7	4.27	4.35	5.01
Gm6802	3.72	4.26	3.76	4.05
Gm25041	4.58	4.81	6.03	4.87
Arl2bp	6.42	6.06	6.98	5.95
Gm24646	3.96	4.64	6.08	4.04
Gm24741	5.09	5.42	4.79	5.38
Mras	5.62	6.16	6.53	5.75
Nmt2	4.67	4.98	5.16	4.78
Gm21936	4.22	4.67	4.12	4.41
Gm20417	7.15	5.84	8.49	5.59
Gm6161	8.81	8.63	10.04	9.42
Gm22051	4.96	4.6	5.17	4.63
Tpt1-ps6	6.63	6.3	6.85	5.83
Gm12571	6.42	5.23	6.19	5.87
Scn2a1	5.34	5.73	6.07	5.19
Hcar2	4.16	3.89	4.02	4.34
Gm9857	3.78	4.23	3.7	3.5
Gm23316	4.26	4.22	4.2	4.81
AY036118	8.29	7.14	9.11	7.38
Gm14996	3.92	4.19	4.09	4.34
Gm20253	4.69	3.75	5.72	4.27
Mbd3l2	4.37	4.63	4.87	4.21
Gm15863	3.76	3.91	3.58	4.48
Mir3964	4.33	3.66	3.56	4.51
Dmrt3	3.85	3.99	3.75	4.21
Rad1	4.15	4.28	3.83	4.23
Arl5a	5.06	5.37	6.57	5.01
Olfr862	4.51	4.7	4.81	5.54
Mir669h	5.16	4.68	5.3	4.99
Pde8b	5.68	5.67	6.36	5.42
Gm24315	5.1	4.2	5.3	4.9
AF357426	7.46	4.23	3.6	4.17
Gm8019	5.76	5.55	6.27	5.27
4930548H24Rik	4.02	4.43	3.86	4.5
Gm11438	6.41	6.22	6	5.1
Igkv4-90	4.64	4.35	3.9	5.22
Gm27012	4.13	4.02	3.91	4.57

Ptprg	6.3	5.8	7.16	5.7
Chmp3	5.42	5.9	6.07	5.62
Mir697	4.66	5.73	5.51	5.76
Gm8358	7.1	5.86	7.01	5.1
Baz1b	5.21	5.17	5.73	5.06
Ppp1cc	6.48	6.18	7.55	5.91
Olfr1449	4.84	4.17	4.06	4.78
Enpp5	5.44	5.39	6.34	5.6
Arnt2	5.21	4.85	5.35	4.82
Ubxn4	5.37	5.47	6.03	5.01
Gm4222	4.28	4.13	3.68	4.21
Rhot1	4.81	4.75	5.2	4.81
Abhd2	5.94	6.48	7.27	6.13
9530051G07Rik	4.14	4.11	3.98	4.43
Prss34	4.26	4.29	4.13	4.73
Pik3c2g	4.35	4.37	4.29	4.65
Gm13368	5.32	5.47	5.35	4.52
Mir298	4.59	4.66	4.51	5.22
Chrnb3	3.87	4.18	3.86	4.18
Gm10309	4.12	4.19	3.77	4.52
Gm26326	5.26	4.84	4.28	4.54
Ankle2	5	5.04	5.8	5.08
Gm2962	7.01	7.67	9.6	5.61
Igkv1-110	4.42	4.28	4.88	4.5
Gm15143	4.14	4.22	3.69	4.4
Gm16394	4.11	3.74	3.61	4.23
Gm25506	4.43	4.64	5.97	4.62
Rab39b	4.13	4.52	3.99	4.18
2010320O07Rik	4.18	4.57	4.56	4.56
Gm10356	5.12	4.03	4.86	5.04
Gm22934	7.03	7.93	7.74	7.76
Rhox7-ps1	6.03	6.8	6.38	6.48
Gm12504	5.83	5.29	6.1	5.49
Slco1c1	7.42	6.88	8.5	6.63
Hacd2	4.5	4.64	5.72	4.65
Gm14587	4.61	3.77	4.06	4.53
Gm25246	6.51	6.59	7	5.71
Gm24245	17.83	11.48	17.64	10.11
Gm24270	17.83	11.48	17.64	10.11
Gm14618	3.52	3.93	3.76	4.19
Gm14409	5.72	4.8	6.43	5.18
Gm24187	18.64	11.59	18.7	9.16
LOC100862384	5.71	4.3	5.72	4.22
Gm13621	3.7	4.12	3.6	4.05

Olfr140	4.57	3.98	4.15	4.53
Gm25911	13.91	9.53	15.07	8.64
C1qtnf3	4.41	4.73	4.26	4.5
Akap2	5.01	5.5	5.75	5.29
4930452N14Rik	3.79	4.23	4.16	4.18
Gm1974	5.12	4.84	5.48	4.74
Gm14371	3.89	3.95	3.68	4.13
Gm26678	3.98	4.4	3.74	3.82
Hdac3	4.75	4.61	5.31	4.59
Ndufa9	4.69	4.78	5.32	4.89
Gm26066	3.95	4.02	3.95	4.77
Gm24556	5.16	5.13	6.14	4.78
Mir3079	3.59	4.42	3.03	2.91
Igkv1-117	4.48	4.17	5.03	4.54
Txndc12	5.22	5.27	5.96	4.98
Esp3	4.15	4.14	3.75	4.43
Resp18	6.4	5.43	5.89	5.64
Cmtm8	4.38	4.32	4.68	4.34
Gm20372	7.45	6.94	8.2	5.94
1700112K13Rik	3.95	4.21	4.26	4.46
Gm25081	4.71	5.33	4.91	4.21
Gm24054	4.61	5.07	5.52	4.49
Gm24352	3.93	4.5	4.35	4.35
Ube2d3	5.35	5.31	5.78	5.06
Igkv4-68	4.22	4.5	3.6	3.95
Eif1ax	5.5	5.57	6.13	5.28
Gm4540	7.74	8.14	8.83	6.52
Gm12458	4.4	4.81	5.41	4.95
Gm5428	8.17	8.36	9.29	7.26
LOC100861984	5.28	4.91	6.42	5.29
Rnaset2a	5.21	5.31	6.4	4.81
Gm8203	7.39	6.07	8.76	6.56
Ighv1-63	6.5	8.01	7.27	7.8
Cd47	5.66	5.28	6.23	4.98
Gm14405	5.28	5.07	6.25	4.88
Gm21562	4.41	4.08	3.76	4.07
Gm21572	4.41	4.08	3.76	4.07
Gm21599	4.41	4.08	3.76	4.07
Gm12511	3.74	3.86	3.77	4.21
Mid1	5.2	5.31	6.3	4.55
Vmn1r-ps146	4.16	4.05	3.98	4.56
Gm25683	5.19	4.94	5.78	6.12
Gm17801	3.67	3.97	3.49	4.12
Polk	5.07	5.43	5.75	5.29

Atp6v1h	4.95	4.53	5.25	4.51
Gm15537	4.38	4.93	4.63	4.7
Spink6	4.19	4.25	4.32	4.67
Vmn2r-ps78	3.71	4.02	3.85	4.56
Gm3230	4.42	4.43	4	4.54
4930545H06Rik	4.25	4.25	4.07	4.94
Cdc42	6.55	5.87	6.8	5.43
9130015L21Rik	3.76	4.06	3.96	4.57
Kcnh7	4.35	5	4.42	4.78
Ptch1	5.25	5.01	5.56	4.93
Serpina3b	4.13	4.02	3.91	4.37
Gm25151	4.11	4.59	3.79	3.93
Gm23894	4.26	4.28	3.97	4.62
Mir7059	3.85	3.89	5.28	4.48
Rnu3a	3.93	3.94	3.7	4.1
Gm23996	3.53	4.12	3.7	3.81
Gm23977	4.08	4.81	4.22	4.69
Trav10d	4.12	4.21	3.87	4.36
Vmn1r208	5	4.45	4.58	4.91
Mir7679	3.49	3.9	3.43	3.97
Gm13163	5.64	5.16	6.26	5.22
Rps6-ps1	9.02	7.16	9.49	6.83
Vmn2r81	4.24	4.19	4.05	4.83
Gm13489	4.06	4.2	3.61	4.1
Olfr1168	4.38	4.54	3.8	3.97
Mir669b	4.83	4.9	7.03	4.76
Gm14175	4.33	4.42	4.94	4.35
Ier3	3.72	4.24	3.69	3.83
Samd9l	4.27	4.98	4.88	4.98
Gm9875	4.29	4.31	3.86	4.53
Gm24162	4.22	4.51	4.28	4.83
Gm14821	4.26	4.49	4.41	4.82
Gm20599	4.79	5.2	4.75	4.71
Gabarapl2	5.89	5.67	6.6	5.4
Gm27297	4.3	4.25	3.94	4.83
Pcna-ps2	6.31	4.53	4.49	4.68
Gm10807	4.19	4.53	4.1	4.36
Gm11988	4.33	5.03	4.44	4.77
Rbm4	4	4.6	4.85	4.39
Gm25868	4.66	4.5	5.16	4.67
Gm22233	4.66	4.5	5.16	4.67
Gm23085	5.38	5.2	4.99	5.84
Pyy	3.99	4.11	3.63	4.06
Firre	4.91	4.81	5.2	4.73

9330159F19Rik	5.46	6.34	7.01	6.2
Ighv1-66	4.53	4.87	4.32	4.04
Bhlhb9	4.69	4.86	5.22	4.87
Kcnh1	5.27	5.65	5.26	5.38
Qdpr	5.07	5.14	5.55	5.06
Gm13550	4	4.02	4	4.56
Mir6239	4.89	5	5.73	4.71
Gm25567	6.43	6.1	7.93	5.95
Foxp1	5.59	5.59	5.98	5.26
Zfp365	4.8	4.74	5.12	4.73
Wasf1	4.8	4.82	5.35	4.71
Tgds	4.34	4.39	5.05	4.44
Olfr1193	4.31	4.16	4.28	4.89
Vmn2r-ps75	3.88	4.2	3.5	3.88
Dbi	6.1	5.27	6.03	5.23
Gm17430	4.62	4.99	5.21	4.73
Gm24041	4.98	4.29	4.38	4.94
Gm24764	4.38	4.82	4.32	5.11
Tex261	4.7	4.83	5.19	4.85
Gm27030	3.97	5.45	4.68	4.36
Gm5356	4.47	4.42	4.77	4.4
LOC100861827	4.41	4.62	4.12	4.25
Agfg1	6.6	6.81	7.68	6.59
Olfr1102	4.39	4.88	4.26	4.12
Olfr816	4.04	3.98	3.6	4.13
Myo10	4.99	4.76	5.4	4.98
Arhgap29	5.66	5.47	6.46	5.56
Gm27243	4.72	4.16	5.74	4.17
LOC100861936	5.18	5.13	6.26	4.5
Gm22684	5.05	4.88	5.49	5.26
Gm5457	7.03	7.32	9.27	5.21
Cdc27	5.14	4.93	5.93	5.24
Zfc3h1	5.08	5.25	6.06	4.99
Gm22519	4.34	5.57	4.72	4.49
Ptpn12	5	4.92	5.39	4.89
Nlrp9c	4.26	4.05	3.97	4.6
Gm22163	3.83	4.23	3.62	4.19
Slc35f1	4.66	4.52	5.07	4.38
Mir684-2	11.8	5.99	16.9	8.08
Bcl2l1	5.44	5.67	5.97	5.36
Gm14981	3.89	4.48	4.08	4.66
H2afz	7.09	6.48	8.11	6.14
Mup2	4.29	4.33	4.2	4.71
Olfr664	4.75	4.76	4.37	5.12

Ccndbp1	4.65	4.82	5.06	4.78
Gm14391	6.08	5.97	6.65	5.63
Gm25128	6.29	6.63	10.27	6.17
Mup2	4.27	4.28	4.08	4.55
Mir297a-3	5.51	4.65	5.88	4.62
Snn	5.2	5.11	5.87	5.22
Havcr1	4.12	4.01	3.82	4.57
Amd2	7	6.5	7.72	5.94
Nsrp1	4.53	4.78	4.96	4.94
Kcnk18	3.59	3.73	3.59	4.07
Gm10184	3.99	3.95	3.75	4.52
Gm25701	4.91	4.5	5.08	4.53
Gm26431	4.91	4.5	5.08	4.53
Gm24488	4.91	4.5	5.08	4.53
Ubap2l	5.52	5.42	6.01	5.37
Gm6265	6.7	6.88	8.13	6.5
Prpf40a	5.16	5.2	5.61	4.96
Tra2a	6.55	5.65	7.27	5.73
Nxn	4.75	4.59	5.15	4.81
Gm7534	4.09	4.13	3.85	4.49
Vmn1r-ps65	4.6	4.79	4.18	4.41
Zfy1	4.4	4.51	4.02	4.24
Gm23190	9.11	9.16	13.63	8.95
Ppp1r14b	5.98	5.71	6.44	5.44
Uqcrb	6.81	6.69	7.58	5.52
Gm4732	4.74	4.56	4.49	4.32
Cers5	5.44	5.01	5.69	5.07
Olfr1166	4.37	4.51	4.26	5
Gm14334	4.4	4.68	4.26	5.34
Mir669m-2	4.7	4.34	5.8	5.06
LOC100862038	5.05	4.63	5.31	4.62
Olfr65	4.04	4.26	4.1	4.58
Gm24631	4.37	4.81	4.41	4.31
Olfr481	3.79	4.6	3.72	4.15
Gm24834	6.91	7.81	10.23	7.81
Gm22713	4.44	4.48	4.31	5.38
Rps15a-ps5	5.64	5.68	6.97	5.41
Chad	3.94	4	3.6	4.45
Gm23266	4.44	4.44	5.04	4.4
Btbd1	5.66	5.48	6.52	5.27
Gm4879	4.33	4.9	4.72	5.35
Gm13798	5.25	5	6.28	4.98
Gm14198	4	3.96	3.72	4.21
Gm13245	4.99	4.67	5.58	4.62

Dnajc13	4.76	4.79	5.18	4.7
Klk1	4.07	4.04	3.81	4.4
3830417A13Rik	4.15	4.1	3.97	4.43
Tpt1-ps5	9.38	8.8	9.86	7.67
Ppp1r9a	4.99	5.2	5.69	5.07
Tgoln1	5.4	5.49	6.31	5.2
Gm15161	4.52	5.21	4.49	4.82
4930488L21Rik	4.27	4.01	3.92	4.28
Gm11835	4.4	4.54	4.15	5.03
Mir136	4.08	4.29	4.14	4.7
Gm12374	4.07	4.43	4.17	4.56
Gm16430	4.13	4.5	4.48	5.08
Gm20706	4.26	4.22	3.74	3.66
Gapvd1	5.17	4.98	5.4	4.93
Gm13795	4.1	4.1	3.86	4.41
Gm13763	4.29	4.54	3.98	4.48
Gm10860	4.06	4.57	4.01	4.38
Gm25495	4.14	4.38	4.85	4.5
LOC100862081	6.88	7.09	8.73	7.38
Gm15030	4.46	5.02	4.27	6.06
Gm19974	6.46	5.37	7.05	5.67
Gm12481	6.91	6.32	7.46	6.07
Mir7212	8.75	9.36	9.33	9.99
Gm12529	7.08	6.36	7.06	5.71
Gm14421	6.03	6.13	7.51	5.74
Gm12238	3.94	5.19	5.05	4.98
Gm23393	5.09	4.9	5.62	4.91
Esco1	4.78	5.03	5.62	4.77
Gm23769	4.86	5.23	5.07	5.61
Gm24791	4.44	5.59	4.71	4.47
Btg1-ps1	4.6	4.1	3.91	4.16
Vmn2r25	4.36	4.55	4.1	4.28
Gm12782	4.17	4.48	3.92	4.29
Id1	5.23	4.96	5.49	5.27
Gm11686	4.75	4.57	5.34	4.45
Ear10	3.96	4.21	3.63	4.29
8430437L04Rik	4.08	3.9	3.74	4.32
Gm12251	5.96	6.23	6.53	4.9
Prom1	5.11	4.92	6.08	5.26
Necab2	5.19	5.05	5.46	5.03
Alkbh8	5.28	4.94	5.43	4.81
Gm22403	5.31	5.35	6.66	5.35
Vmn1r-ps18	3.91	4.1	3.77	4.39
Mir466j	7.8	8.16	10.76	7.75

Gm15084	3.89	4.02	3.59	3.7
Mtag2	3.55	3.7	3.43	3.92
Mbd5	4.73	4.91	5.22	4.67
Tardbp	5.73	6	7.29	5.21
Arih1	5.19	5.22	5.86	5.22
Nae1	5.29	5.31	5.71	5.03
Gltscr1l	4.59	4.73	4.95	4.49
Gm14411	6.73	5.9	7.3	5.89
Rbm33	5.21	5.12	5.71	5.1
Cep350	5.23	5.02	5.59	5.05
Olfr1381	4.65	4.83	4.8	5.53
Paip2	7.56	6.46	8.44	6.31
Olfr384	4.34	4.45	4.39	5.48
Igkv18-36	4.07	3.83	4.11	4.38
Gm17113	6.52	7.33	6.98	6.94
Plpp3	5.49	5.08	5.57	4.61
Gm27248	7.85	7.76	8.26	6.64
Scarna8	3.99	4.06	3.92	4.61
Gm12843	4.38	4.45	4.01	4.28
Hist2h2aa1	6.91	5.51	6.67	5.71
n-R5s70	4.94	4.74	4.65	5.17
Gm22686	4.3	4.93	4.56	4.6
Gm15806	4.18	4.46	3.97	4.26
Traj14	9.98	14.03	12.27	14.1
Gm14418	7.59	6.64	7.38	5.8
Gm14589	4.21	4.41	3.9	4.44
Gm10813	4.08	3.89	3.44	4.79
Gm5086	4.24	4	3.76	4.38
Gm16063	5.2	5.28	5.65	5.06
Poll	3.93	4.06	3.97	4.32
Gm21994	4.52	4.48	3.97	4.48
Mrgpre	4.01	4.19	3.83	3.97
Pnmal1	5.45	4.89	5.26	5.02
Gm15739	4.13	4.15	4.32	6.22
Gm829	3.97	4.4	3.82	4.27
Gm25275	4.06	4.5	4.21	4.23
Olfr486	4.07	3.9	3.72	4.43
Ube2nl	4.48	4.78	4.58	4.08
Gm24482	6.08	5.74	7.06	5.7
Rnf148	4.29	4.48	3.82	4.36
Gm7134	4.13	4.45	4.13	4.65
Rps19-ps7	5.69	5.32	6.09	5.34
Mir346	4.62	4.55	5.24	4.73
Gm24253	4.62	4.23	3.92	4.25

Trpd52l3	3.51	3.73	3.63	3.96
Zfp445	5.59	5.43	5.88	4.95
Gm12883	4.13	4.94	4.1	4.2
Maoa	5.01	4.86	5.34	4.99
Trim30e-ps1	4.3	4.1	3.81	4.29
Olfr494	4.8	4.14	4.45	5.1
Gm15963	4.12	3.67	4.55	4.29
Gm12745	4.26	4.5	4.39	4.03
Gm4221	4.28	4.28	3.87	4.42
Gm4925	4.1	3.86	3.63	3.94
Esyt2	5.2	5.19	5.85	5.24
Ammecr1l	4.51	4.92	5.17	4.59
Gm6023	6.94	6.41	7.16	5.51
Gm14643	4.2	4.24	4.27	4.61
Vmn2r-ps60	4.13	4.32	3.98	4.57
Lta	4.27	4.1	4.04	4.55
Olfr473	3.9	4.35	3.78	4.21
5830428H23Rik	4.86	5.15	5.31	4.77
Gm14308	6.79	6.5	7.82	6.05
Pabpc1	5.55	5.23	5.92	5.1
Siah1a	4.92	4.81	5.29	4.59
Gm25435	4.3	5.26	4.65	5
Mir3471-1	4.63	4.79	5.49	4.55
Gm16238	4.92	5.16	5.78	4.51
Gm21111	4.03	4.65	4.12	4.04
Ighv1-43	4.73	4.24	3.8	4.36
Gm23026	4.61	5.08	4.27	4.89
Neurod4	4.21	4.81	4.05	4.21
Inpp4b	4.7	4.73	5.17	4.65
Zscan5b	4.57	4.56	4.39	4.8
Zbtb20	5.75	5.1	6.24	5.55
Gm12885	3.68	4.13	3.72	4.04
Vmn1r200	4.33	4.65	4.69	5.04
Gm12432	4.4	5.27	5.39	5.22
Olfr834	3.86	3.72	3.91	4.53
Gm7381	5.24	6.09	7.58	5.24
Gm16045	4.49	4.42	4.11	4.5
Cox11	4.83	4.5	4.87	4.19
4921518K17Rik	3.88	3.93	3.96	4.35
Hsd3b6	4.31	4.48	4.1	4.46
Gm16025	4.38	4.4	4.22	4.92
Plp2	4.35	4.47	4.33	4.78
LOC100861752	5.47	4.49	6	4.46
C6	4.24	4.42	4.17	4.56

Nudt2	4.57	4.04	4.08	3.94
Pdzd9	4.57	4.73	4.26	4.54
Gm27551	3.58	4.14	3.41	3.82
Rnf113a1	4.13	4.43	3.78	4.03
Gm20317	4.01	4.16	3.69	4.01
Olfr875	3.71	3.94	3.47	4.51
Kif5b	6.97	6.87	7.71	6.48
Gm11295	3.91	5.63	5.67	4.77
Vmn2r13	4.3	4.88	4.25	4.46
Gm12480	5.46	5.28	5.7	4.46
Jag1	4.79	4.76	5.42	4.75
Spata31d1a	3.87	3.83	4.33	4.05
Gm20349	5.03	4.48	6.83	4.51
Gm9109	4.38	4.1	4.01	4.4
Gm24284	4.62	4.4	4.21	3.93
Gm14391	6.47	6.38	7.4	5.92
F830045P16Rik	4.1	4.25	3.81	4.36
Lsm12	6.33	6.32	6.93	6.09
Arel1	5.24	5.15	5.92	5.17
Gm14369	4.54	4.84	5.27	4.8
Gm23644	4.44	5.02	4.26	5.31
S1pr1	6.67	6.82	7.19	6.13
4930407I10Rik	3.9	4.21	3.78	3.89
Gvin1	5.36	5.64	6.37	5.26
Olfr1392	4.01	4.14	4.03	4.89
1700018M17Rik	4.41	4.36	3.93	4.46
Epha4	4.88	5.14	5.65	5
Cdk13	5.66	5.21	7.08	5.11
Gm13261	4	4.08	3.69	4.07
Gm27199	4.25	4.01	3.81	4.21
Nsd1	5.84	5.79	6.45	5.84
Gm23312	4.48	4.48	4.26	5.09
Gm27161	3.83	4.07	3.75	4.15
Gm24037	4.26	4.62	4.53	5.36
Ap4b1	4.25	4.25	4.55	4.22
Gm2174	4.78	4.41	5.92	4.11
Gm22784	4.29	4.69	4.13	4.45
Gm7457	4.28	4.65	4.24	4.49
Mir467h	8.19	8.86	10.31	8.53
Ppp1r2	5.55	5.35	5.96	5.22
Gm22337	3.73	3.99	3.96	4.26
Gm15303	3.84	3.82	4.19	4.59
Gm17180	5.23	4.99	5.41	5.24
Sycp1-ps1	4.49	4.44	3.95	4.35

Mctp1	4.99	5.06	5.6	4.93
Gm16345	4.67	4.44	5.47	4.83
Gm23542	3.95	4.44	5.57	4.87
Gm14980	4.78	4.74	4.37	4.97
Gm22491	4.2	4.2	4.1	4.75
Mup-ps12	4.21	4.24	4.16	4.7
Gprc5d	3.96	3.94	3.84	4.23
Ly6d	4.1	4.11	3.91	3.61
Gm25935	5.57	6.54	6.74	5.25
Arfgef2	4.74	4.76	5.09	4.64
Ifit1bl2	3.84	4.4	3.84	4
Mpped2	4.98	5.15	5.54	5
Kpna4	4.62	4.75	5.39	4.59
Zfp638	5.75	5.68	6.7	5.08
Srsf7	5.76	4.9	6.01	4.99
Olfr215	3.66	3.94	3.87	4.21
Olfr503	4.12	4.11	4.02	4.46
Gm22078	4.03	4.21	3.61	4.37
Gm24623	4.1	4.47	3.94	4.55
Gpr82	4.15	4.3	3.82	4.13
Gm23278	5.4	5.68	7.15	5.41
Eif5	5.54	5.45	5.87	4.86
Aplp2	6.66	6.66	7.93	6.61
Gm22268	4.35	4.23	4.63	4.06
Vmn1r-ps40	3.91	3.82	4.1	4.46
Gm26513	4.05	4.4	3.66	3.84
Csnk2a2	4.58	4.74	5.45	4.71
Clec5a	4.2	4.23	3.88	4.26
Gm14275	4.17	4.57	4.73	4.47
1700013G24Rik	4.31	4.79	4.21	4.79
Gm11843	5.91	5.23	8.54	7.14
Parp2	4.52	5.15	4.9	4.5
LOC100862198	5.2	4.13	6.56	4.91
Gm11462	4.34	4.47	4.72	4.19
Gm24001	5.6	4.47	4.6	4.62
Mir669m-1	4.3	4.78	5.96	4.73
Gm13782	4.21	5.12	3.91	4.46
Rpl36-ps7	3.94	4.34	3.91	4.01
Tmem126a	4.61	4.9	5.18	4.95
Scml1	4.5	4.01	3.69	4.21
1700029J07Rik	4.86	4.66	5.32	4.44
Ociad1	5.29	5.7	6.33	5.48
Cryl1	3.94	4.14	3.88	4.21
Fopnl	4.73	4.77	5.11	4.41

Gm19863	3.91	4.44	4.35	4.17
Gpbp1	5.26	5.33	6	5.16
Klk1b21	4.26	4.34	3.98	4.48
Disp1	4.38	4.27	4.6	4.29
Gm10232	8.15	7.53	9.11	7.25
Impact	7.02	7.11	8.57	6.43
Ppp1cb	5.8	6.77	7.75	6
Gm8062	7.89	6.39	7.73	6.01
4930470H14Rik	4.31	4.11	4.19	4.49
Gm17976	3.91	4.08	3.62	4.27
Gm25233	7.06	5.66	7.47	5.35
Gm22301	4	4.73	4.15	4.52
Skil	5.15	5.01	5.54	5.18
Plxna4	4.9	4.99	5.39	4.89
Fasn	4.82	4.89	5.26	4.77
Gm14782	4.34	4.2	4	4.52
Gm21435	4.39	4.75	4.34	4.42
Gm13860	4.11	4.46	3.73	4.5
FMR1-AS1_1	4.05	4.03	3.81	4.55
Wwp1	5.55	5.57	6.19	5.57
Megf9	5.17	5.15	5.24	4.73
LOC100861706	3.93	4.28	3.75	4.09
Gm25253	4.35	5.2	4.61	5.05
Gm11470	4.03	4.27	3.86	4.42
B3gnt6	3.66	4.12	3.24	3.51
Gm22232	7.59	8.04	9.35	7.19
Gm2862	4.2	4.3	4.2	5.02
Gm14985	4.55	4.51	4.08	5
Tnfaip8l2	4.45	4.79	3.95	4.14
Vmn1r84	4.14	4.82	3.89	5.09
Gm23042	3.95	5.13	4.02	4.36
Mir1943	4.44	4.58	5.19	4.4
Gm18397	4.84	4.34	4.22	4.32
Gm24457	4.74	5.03	5.04	4.55
Gm6280	3.66	4.18	4.02	4.21
Gm25665	5.42	5.26	5.9	5.22
Gm23885	4.53	4.51	3.96	4.35
Bcl11b	5.33	4.75	5.18	4.66
Gm16206	3.85	3.97	3.51	3.94
Gm2026	7.37	6.97	8.54	6.47
3110021N24Rik	4.25	4.46	4.41	4.77
2810404F17Rik	4.01	4.7	4.14	4.15
Bet1	4.24	4.31	3.98	4.51
Mup19	4.3	4.34	4.21	4.76

6530401F13Rik	3.79	4.07	3.53	3.99
A830039N20Rik	5.75	5.41	6.42	5.76
Gm25525	4.4	5.77	4.23	4.43
Gm19560	3.93	4.72	4.08	4.12
Mir216b	4.01	4.02	3.82	4.58
LOC100505062	5.1	5	6.77	5.05
Lrrc4c	5.62	6.04	7.17	5.48
Hdhd1a	3.99	4.2	3.64	4.05
LOC100862216	7.6	5.67	7.65	5.36
Gm26140	5.29	5.16	5.81	5.33
Gm14269	6.26	5.69	7.12	5.42
Olfr1397-ps1	4.03	4.01	3.9	4.43
Gm16050	4.32	3.98	4	4.47
Gm15912	4.61	4.97	5.01	4.67
Gm16086	3.98	3.72	3.93	4.42
Grxcr1	4.2	4.28	3.99	4.47
Gm23782	4.07	4.64	3.94	4.5
Sumo1	5.88	6.19	7.24	5.09
Olfr1	3.92	4.19	3.99	4.44
Gm21979	3.65	4.22	4.1	3.87
LOC100861900	4.69	4.75	5.6	4.53
Gm12846	3.94	4.54	4.43	4.03
Gm24947	4.4	4.21	4.03	4.4
Gm11798	4.76	4.45	3.96	4.69
Gm23062	5.22	4.27	4.25	4.87
Tbc1d22b	4.72	4.45	4.27	4.51
Gm13246	4.8	4.68	5.2	4.31
Gm16270	4.11	4.17	4.17	4.5
Gm16142	4.53	4.69	4.25	4.5
Gm23159	9.54	11.69	11.37	10.17
1500026H17Rik	4.16	4.19	4	4.63
Gm19854	4.19	4.18	3.61	4.14
Gm26588	4.24	4.58	4.11	4.47
Dennd4a	4.73	4.83	5.27	4.78
Vmn1r48	4.07	4	3.77	3.99
Numa1	5.95	5.25	6.08	5.57
Selk	5.36	5.1	5.83	5.14
4930555B12Rik	4.59	4.73	4.47	4.77
Zfp932	5.88	5.88	6.88	5.41
Gm11583	4.96	4.39	5.22	4.51
Serpinb5	4.14	4.44	4.01	4.16
LOC100862107	5.26	4.86	5.68	4.61
Gm15566	4.56	4.83	4.58	5.02
Serpina3l-ps	4.2	4.45	3.98	4.28

Rbm4	4.02	4.44	4.66	4.64
Ralgapa1	5.48	5.73	6.52	5.65
Mir767	3.86	4.27	3.86	4.37
2900055J20Rik	5.1	6.07	4.92	4.6
Tas2r106	4.24	4.33	3.81	4.73
Akip1	4.59	4.58	4.52	5.06
Gm23349	4.28	5.02	4.21	4.7
Snora41	7.38	7.11	7.07	7.98
Lrrd1	4.07	3.98	3.78	4.13
Gm23315	4.51	4.68	4.18	5.28
Gm20275	4.36	4.06	4.06	4.51
Rapgef5	4.64	4.99	5.49	4.73
Tlr9	4.81	4.38	4.21	4.24
Baalc	5.45	5.7	6.06	6.19
Vmn2r108	4.12	4.26	4.14	4.78
Gm10110	4.8	4.41	4.55	4.18
Gm5094	4.22	4.17	3.91	4.67
Lmod2	3.85	4.15	3.73	4.13
Gm23334	4.67	5.19	7.94	4.74
Gm26874	4.53	4.86	5.08	4.81
Gm4883	8.73	8.65	9.44	8.34
A130040M12Rik	6.43	6.45	7.74	5.55
Gm19316	4.26	4.15	4.18	4.84
Gm3272	7.25	7.19	8.22	6.37
Rasd1	3.79	4.33	3.65	3.62
Ube2v2	5.15	4.91	6.14	5.35
Kat2b-ps	5.55	6.01	5.31	5.87
Gm15157	4.73	5	4.12	4.85
Speer8-ps1	4.35	4.32	4.76	4.56
Mir493	3.82	4.06	4.8	4.23
Gm5400	4.65	5.01	4.57	5.2
Nab2	5.33	5.15	5.63	5.18
Olfr781	3.97	4.27	3.8	4.14
Olfr775	4.06	4.28	3.74	4.38
Gm26723	5.43	4.66	5.65	4.85
Gm20407	4.47	4.75	4.58	5.24
Vmn1r-ps58	4.12	4.85	3.99	4.28
Ccnd3-ps	4.2	4.2	4.63	4.15
Rps4l	5.26	4.27	3.96	3.95
Gm23881	6.1	6.15	9.36	6.11
Gpr142	3.89	3.67	3.81	4.15
Slc25a3	5.28	5.18	5.47	4.84
Rspo3	4	4.69	4.06	4.42
Gm21860	4.07	4.05	4.43	3.91

Gm21748	4.07	4.05	4.43	3.91
Vmn2r-ps45	4.22	4.65	4.32	4.3
Gm23853	5.41	5.9	5.19	6.31
Gm22694	7.78	7.72	8.46	7.08
Igkv4-92	3.75	4.37	3.59	3.93
Timm8a2	3.84	4.33	3.77	4.13
A330102I10Rik	4.25	4.73	4.38	4.38
Olfr1289	4.05	4.29	3.91	3.65
Gm23408	3.96	4.14	4.28	4.59
Vmn2r4	4.28	4.12	4	4.4
Mir684-1	11.95	5.9	16.91	8
Mir684-1	11.95	5.9	16.91	8
Mir684-1	11.95	5.9	16.91	8
Mir684-1	11.95	5.9	16.91	8
Mir684-1	11.95	5.9	16.91	8
Gm12214	4.33	4.61	3.98	4.27
Gm22953	9.19	9.83	10.09	9.53
Gm23997	5.31	5.42	5.26	6.25
Olfr780	4.21	4.53	3.93	4.17
Gm26793	3.66	3.71	3.38	4.06
Sdha	5.36	5.45	6	5.37
Olfr1214	4.3	4.19	3.87	4.03
Zfp628	3.85	4.03	3.88	4.34
Gm6963	4.14	4.32	4.8	4.45
Gm12377	4.18	4.45	3.75	4.29
0610038B21Rik	3.75	3.89	3.49	3.68
Zscan30	4.52	4.81	4.33	4.66
Gm23459	4.06	4.27	4.56	4.59
Gm5129	3.65	4.39	3.97	3.83
Gm15897	4.04	4.27	3.85	4.44
Gm24665	4.7	4.8	5.22	5.3
Riok2	4.55	4.8	4.35	4.43
Gm7020	4.05	4.44	4	4.52
Slc25a37	4.26	4.72	4.65	4.53
Mir466k	7.85	7.86	10.67	7.62
Gm9242	8.79	8.49	10.06	9.06
Gm13035	4.41	4.12	4.55	3.9
Gm4773	4.21	4.61	4.19	4.06
Gm26933	3.65	3.94	3.63	3.91
Lrrfip2	5.18	4.94	5.55	5.1
Ighv1-67	4.74	4.09	4.19	4.09
Fam120a	6.55	6.36	6.97	6.12
Ighv8-14	3.58	3.66	3.68	4.19
Gm17728	3.66	3.68	3.52	4.23

Gm22257	4.08	4.54	3.74	4.4
Ighv6-3	4.68	4.26	5.28	4.54
Gm25490	3.97	4.54	4.48	4.41
Nup153	5.3	5.24	5.88	5.14
Arl8a	6.08	6.35	6.86	6.19
Olfr1066	4.32	4.61	3.94	4.51
Prex2	5.08	5.17	5.8	5.06
Gm27510	4.39	4.36	4.09	4.59
Gm26486	3.82	4.22	3.75	4.2
Tas2r123	4.26	4.31	3.76	4.34
Gm26792	4.28	4.68	4.12	4.24
Snhg3	4.8	4.5	5.11	4.63
Gm24070	3.79	4.04	3.75	4.49
Gm23136	4.24	4.47	4	4.53
C030014I23Rik	3.96	4.36	4.07	4.08
Gm13064	4.01	4.17	3.73	4.23
Gm6897	3.86	4.16	3.8	4.24
Gm4899	4.2	4.4	4.56	5.41
Ttc14	5.23	5.28	5.49	5.1
Phgdh	5.07	4.91	5.5	4.91
Grpel2	4.33	4.68	5.05	4.63
Esp8	4.55	4.5	3.99	4.37
Zfp106	5.56	5.82	6.25	5.4
Gm13749	4.4	4.84	5.17	5.34
Srrt	6.17	5.82	6.54	5.87
Pllp	4.21	3.99	4.69	4.13
Dzip1	4.77	4.55	5.09	4.62
Ndufaf1	4.65	4.77	4.8	5.09
Nipal1	3.92	4.38	3.98	4.27
Gm15340	4.12	3.79	3.98	4.47
Gm24557	7.36	7.03	10.18	6.78
Gm19461	4.17	4.13	4.07	4.36
Gm13460	4.25	4.26	4.17	4.7
Gm20751	4.73	4.72	4.74	5.07
Szrd1	5.71	5.39	6.58	5.48
4922502B01Rik	4.64	5.14	4.74	4.97
Gm23406	4.09	4.8	4.28	4.42
Gm25012	4.7	4.65	4.67	5.44
LOC100861943	4.67	4.15	3.95	4
Gm12150	4.62	4.67	4.27	3.84
Rps6-ps3	11.27	9.09	11.13	8.61
Arhgap12	4.66	4.87	5.29	4.69
Gm8540	4.13	4.57	4.25	4.46
F830016B08Rik	3.88	4.71	4.11	5.07

Gm26583	4.4	4.39	3.94	4.24
LOC100861833	7.26	5.1	8.65	5.42
Arih2	4.56	4.59	4.91	4.49
Ivns1abp	7.22	7.12	8.39	7.03
Obox4-ps2	4.23	4.41	4.34	4.81
Gm15609	3.9	4.01	3.64	3.99
Trav15-1-dv6-1	3.87	4.23	4.46	4.09
Gm13249	6.03	6.26	6.94	5.66
Ptprz1	5.57	5.03	5.8	5.09
Trmt5	4.5	4.36	4.04	4.44
Mier3	4.77	4.72	5.14	5
Mycs	4.07	4.07	3.97	4.45
Gm26195	5.27	6.28	7.09	6.32
Anp32b	5.79	5.76	6.55	5.93
4931408D14Rik	4.05	4.51	3.83	4.16
Gm4825	4.01	4.11	4.52	4.31
Neu2	4.2	4.54	4.17	4.75
3110039I08Rik	5.04	4.96	5.5	4.86
4933409K07Rik	5.19	5.5	5.46	5.44
1700084M14Rik	3.97	4.48	3.76	3.82
Gm9506	4.66	4.07	4.23	4.3
Acly	4.71	4.98	5.1	4.84
Atp6ap2	5.48	5.39	6.69	5.44
Pgam1-ps1	4.57	5.07	4.7	4.89
Mir411	3.78	4.44	3.41	3.81
Olfr1170	4.06	4.25	4.29	4.83
Tmem123	4.74	4.94	5.27	5.03
Gm5093	6.53	6.41	7.56	6.37
Gm19938	4.85	4.8	5.14	4.72
Gm11423	4.6	4.99	4.43	4.94
Ddx25	5.15	5.43	5.54	5.27
Tbc1d8	4.33	4.27	4.64	4.21
Tram1l1	4.43	3.91	4.14	3.85
Pphln1	5.11	4.74	4.97	4.69
Gm6923	3.75	4.6	3.97	3.98
Gm24985	4.6	5.39	5.58	4.85
Gm24248	3.79	4.41	3.98	4.11
Gm23637	6.36	7.22	8.77	6.74
Cyp2a12	4.22	4.33	4.04	4.55
Ear-ps3	4.21	4.15	4.23	4.71
Krtap4-6	3.94	4.21	3.87	4.14
Gm6189	3.65	4.22	3.71	3.74
Amfr	5.19	5.39	6.04	5.37
Fnbp1l	5.31	6.23	6.8	5.8

Kcne3	4.2	4.19	4.11	4.53
Gm5136	4.46	4.65	4.17	5.18
Gm24402	7.94	8.57	8.44	8.54
Enpp6	3.96	4.08	3.81	4.3
Ypel5	5.26	5.2	5.46	4.85
Guf1	4.78	5.1	5.28	4.9
Lin7c	4.92	5.23	5.81	5.07
Bmp5	4.25	4.3	4.77	4.49
5430440P10Rik	4.13	4.54	4.62	4.16
Gm12162	4.06	4.13	3.74	4.33
Olfr402	3.77	3.99	3.4	4.08
1700019O17Rik	3.8	3.89	3.85	4.37
Reep5	5.39	5.54	6.29	5.64
Usp4	4.73	4.95	5.31	4.62
Clmp	4.32	4.26	4.72	4.32
Selt	7.01	6.7	7.76	6.34
Hnrnpdl	5.07	5.22	5.48	5
Psmb7	6.3	5.13	5.88	5.03
Abhd5	4.8	5.08	5.12	4.77
Gm26401	4.04	4.24	3.85	4.42
Btf3l4	5.95	5.39	5.97	5.25
Supt16	4.95	5.07	5.51	4.93
Defb5	4.03	4.35	4.79	5.03
Gm23892	3.78	4.66	3.79	3.9
Hoxb9	4.32	4.46	4.05	4.29
Gm14412	6.52	5.96	7.61	5.43
Gm26233	7.96	8.12	10.57	8.06
Gm4201	4.44	4.18	4.03	4.36
Gm4201	4.44	4.18	4.03	4.36
Gm27942	4.53	3.81	4.28	4.53
Apc	4.9	5.17	5.4	4.91
4930428E07Rik	4.37	4.33	4.04	4.49
Gm26202	5.72	6.52	9.19	7.77
Gm9785	3.89	4.09	3.74	4.29
Rpl35a-ps5	8.62	7.39	10.89	7.39
4833420G17Rik	5.23	5.18	5.75	4.99
Tas2r139	4.18	3.78	3.77	4.26
Mir30f	3.75	3.66	3.54	4.17
Gm26809	5.07	4.37	5.5	4.49
Mir7226	8.42	9.09	8.96	9.58
Gm8036	4.78	4.39	4.06	4.15
n-R5s194	3.77	4.18	3.64	4.08
Pcp4l1	5.26	5.34	5.54	4.8
Fam122a	5.4	4.95	5.56	5.23

F2rl1	3.7	3.61	3.66	4.12
D330020A13Rik	4.38	4.66	4.35	4.33
Mir692-1	5.3	4.7	5.66	4.72
Gm23024	4.03	4.23	4.13	4.6
Gm14180	4.16	4.22	5.09	3.96
Gm14913	4.38	4.65	3.72	4.14
Gnb5	5.51	5.48	6.07	5.11
Mup7	4.37	4.51	4.22	4.89
Tdgf1-ps2	4.29	3.84	4.39	3.66
Gm24268	4.2	4.67	3.92	4.53
Gm13535	4.04	4.15	3.72	4.26
Arrdc3	4.65	4.67	5.48	4.59
Gm11351	4.03	4.19	3.97	4.52
Gm22048	7.18	8.06	9.18	7.09
Lrrc58	5.21	5.24	6.14	5.19
Rbm4	3.86	4.36	4.65	4.58
Gm19471	3.85	4.25	3.74	3.8
Fus	7.65	7.56	8.14	7.33
Phkb	4.59	4.82	5.13	4.66
Gm7166	4.06	4	4.03	4.57
LOC106029237	3.93	4.7	4.19	4.16
Fbxw18	4.11	4.34	4.18	4.49
Gm13811	4.7	5.26	4.69	4.51
Stox2	5.25	5.11	5.72	5.05
Akr1e1	5.19	5.23	5.87	4.97
Ighv3-8	4.26	4.44	3.94	4.78
Hist1h2bc	4.57	4.79	6.33	4.77
Snora47	7.04	7.07	7.21	8.01
Mir6915	7.45	7.08	5.68	6.45
Gucy1a3	5.25	5.39	5.95	5.01
Gbgt1	3.92	4.04	3.69	4.11
Gm24883	4.46	4.31	4.24	4.7
Gm4265	4.3	4.14	4.02	4.51
Gm10254	7.98	6.94	10.17	6.98
Tas2r118	4.01	4.55	4.25	4.72
Mir297a-4	4.63	5.16	5.48	4.8
Gm14405	5.25	4.69	6.2	4.94
Olfr394	4.18	4.47	4.18	5.21
Ptpn14	4.91	4.58	5.22	4.78
Mir878	4.1	4.39	3.85	4.51
Gm14427	4.23	5.04	4.71	4.71
AA545190	4.23	4.56	4.07	4.27
Terf1	4.42	4.55	4.83	4.45
Ube2w	4.77	4.99	5.28	4.98

Map6	4.9	4.39	4.46	4.51
Gm2310	5.29	5.77	6.33	3.95
Btg3	4.87	4.68	5.44	4.56
Gm13991	4.5	4.96	5.14	4.79
C130026L21Rik	4.02	4.16	4.01	4.41
Gm8122	4.79	4.55	4.32	4.77
Olfr549	4.03	3.95	4.54	3.86
Gm15065	5.25	5.17	4.47	4.86
Usp32	5.39	5.36	5.91	5.22
Olfr161	4.44	4.26	3.94	4.1
Cldn34c4	3.77	4.95	3.92	4.04
Gm12183	6.21	5.54	6.09	4.88
LOC100862246	5.09	4.2	5.45	4.97
Mfge8	4.83	4.84	5.06	4.66
PVT1_2	4.79	5.04	4.8	5.65
Ighv10-1	8.71	9.93	8.16	9.83
Aqp6	3.99	4.01	3.94	4.28
Hgsnat	5.02	4.87	5.5	4.92
Gm20463	8.29	8.45	8.8	8.68
Gm26594	4.56	4.34	4.37	4.78
Pdx1	3.84	3.88	3.85	4.22
Sprr2i	4.41	4.15	3.88	4.6
LOC100862137	4.09	4.8	4.12	4.52
Gm23487	6.45	7.18	7.83	6.28
Vmn2r75	3.81	4.06	3.92	4.87
Gm5767	3.83	4.19	3.76	4.24
Rxfp2	4.03	4.31	3.87	4.28
Olfr169	4.35	4.39	4.12	4.77
Ergic2	4.68	4.82	5	4.67
Hoxc5	4.21	4.35	3.93	4.15
Gm26440	5	4.47	5.41	4.5
Gm24705	4.5	5.45	4	4.52
Gm24591	5.45	4.5	4.88	3.72
LOC100862058	4.27	4.07	4	4.39
Rab5b	5.01	5.37	5.68	5.4
Lyzl4	4.24	4.41	4.12	4.5
Gm14082	4.26	3.85	3.74	4
Serpina7	4.15	4.22	3.78	4.29
Mff	5.74	5.46	6.31	4.82
Gm23018	4.36	4.37	5.23	4.17
2310014F06Rik	4.18	4.2	4.18	4.46
A730020E08Rik	4.25	4.07	3.82	4.36
Gm6938	3.86	4.09	3.71	4.14
Gm17711	4.23	4.53	4.1	4.39

Gm11008	6.57	6.21	8.8	6.1
Gm15033	3.69	3.86	3.54	4.07
Olfr1308	4.22	4.45	4.09	4.33
Gm22849	3.97	4.14	4.03	4.38
Gm8580	6.66	5.99	7.51	5.91
Gm13542	3.88	4.34	3.73	3.86
Adam10	5.66	5.59	6.34	5.41
Mir681	4.15	4.15	4.51	4.1
Fbxl17	4.56	4.8	5.18	4.59
Gm15213	4.28	4.09	3.69	3.96
Gm16060	7.02	6.98	7.63	5.67
Gm23384	4.36	4.81	3.93	4.58
Gm12696	6.2	5.09	5.6	5.79
Ttc6	4.36	4.35	4.12	4.6
Gm5523	5.48	5.31	6.22	5.46
Tmprss3	3.87	3.92	3.77	4.09
1810022K09Rik	4.89	5.35	5.98	4.18
Insl5	4.23	4.14	5.06	3.96
Foxo6	3.8	3.76	3.65	4.04
Hspa4	5.65	6.05	6.91	5.74
Gm26165	5.12	5.17	4.94	5.55
Gm12643	6.44	6.4	7.47	6.14
Pten	6.69	6.64	7.78	6
Pacsin2	5.58	5.8	6.16	5.79
Gm22693	4.59	4.9	4.11	4.81
Mlh1	4.79	4.92	5.35	4.9
Pfdn6	5.59	5.13	5.23	5.05
Vmn1r174	3.99	4.18	3.76	3.8
Gm19609	4.33	5.41	5.47	4.77
2410016O06Rik	4.86	5.42	5.05	5.34
Klf1	4.6	4.29	4.35	4.8
Eml4	4.42	4.7	4.98	4.52
Gm24907	3.99	4.58	4.02	3.78
Mgp	5	5.41	7.78	5.75
Vmn2r49	4.11	4.32	3.94	4.04
Vmn2r-ps17	3.86	4.12	3.95	4.57
Gas6	4.68	4.93	5.22	4.92
Olfr5	4.15	3.96	3.62	4.02
Gm12022	3.95	4.01	3.88	4.23
Tspan4	4.1	4.6	4.41	4.22
Gm13878	4.75	4.3	4.32	4.1
Gm17170	3.73	4.33	3.93	4.14
Clptm1	5.32	5.49	6.21	4.95
Gm14641	4.03	4.12	3.83	4.07

Olfr333-ps1	4.03	4.13	4.13	4.45
Nudt10	4.21	4.32	4.03	4.49
Ypel3	5.24	5.03	5.51	5.24
Creb3l4	4.33	4.64	4.24	4.56
Pdgfrb	5.93	5.36	6.17	5.4
Glg1	5.36	5.28	5.95	5.18
Gm23388	14.76	10.21	14.76	8.83
Gm23439	3.62	4.15	4.16	4.08
Tssk6	4.61	4.52	4.23	4.48
Gm12591	4.41	4.79	4.24	4.24
Gm17251	3.95	4.6	4.24	4.39
Cd38	4.51	4.44	4.96	4.58
Sprr2g	4.03	3.73	3.6	3.97
Dmrtc1a	4.79	4.72	4.25	4.8
Gm15935	4.13	4.51	4.23	4.36
Gm5830	6.98	7.88	7.79	6.69
Gm23518	4.2	4.72	4.64	5.14
D030025P21Rik	4.39	4.08	4.26	4.81
Vmn1r74	4.17	4.22	3.51	4.62
Gm20712	4.09	4.49	4.21	4.18
Ddc	4.62	4.52	4.91	4.52
Gm21859	4.42	3.94	4.04	4.47
Znfx1	4.52	4.47	4.74	4.55
Ezh2	5.01	4.8	5.25	4.74
Hcn4	3.75	3.86	3.71	4.07
Gm17518	3.81	3.95	3.74	4.12
Rock1	5.52	5.78	6.39	5.25
Gm19861	4.19	4.61	4.21	4.26
Olfr495	4.21	4.21	3.96	4.47
C1qb	3.97	4.2	4.86	3.83
Olfr345	3.8	4.15	3.55	4.12
Zfp91	4.92	5.06	5.35	4.92
Gpc5	4.66	4.82	4.88	4.59
Mir15b	4.05	4.01	4.23	5
Gm22696	4.62	5.16	5.94	4.5
Naaladl1	3.9	4.02	3.73	4.18
Gvin1	5.39	5.25	5.8	5.04
Gm5564	5.68	5.48	5.7	4.64
Pom121l2	4.01	3.85	3.65	3.86
Gm20899	6.94	7.31	7.94	6.56
Scgb1b2	4.24	4.21	4	4.36
Gabrr2	4.08	4.06	4.01	4.34
Gm25782	4.67	4.62	4.32	4.25
Gm26135	5.52	5.59	6.57	5.11

Fmr1nb	4.32	4.29	4.32	4.67
4932435O22Rik	4.72	4.9	4.68	5.11
Olfr975	3.92	3.93	3.79	4.3
n-R5s172	3.78	3.89	3.52	3.98
Mdm4	4.82	4.91	5.63	4.91
Gm13411	4.26	4.44	4.04	4.69
LOC100503279	7.13	4.79	7.26	4.67
Tead1	4.86	4.57	5.02	4.67
Olfr1159-ps1	4.16	4.68	4.44	4.42
Gm12399	4.03	4.47	4.31	4.61
Gm23990	5.6	6.38	6.86	5.76
Rps12-ps5	8.9	7.32	9.3	7.7
Mir669o	6.06	5.7	6.77	5.96
Golph3	4.51	4.66	4.79	4.37
Gm14308	6.55	6.5	7.01	5.84
Gm25071	7.14	7.16	9.92	6.26
Mir684-1	12.3	7.81	17.06	8.18
Tas2r135	4.88	4.76	4.82	4.33
Gm12881	5.12	4.85	5.34	4.48
Asah1	5.33	5.4	5.52	5.11
Gm8822	6.19	7.24	7.53	5.83
Pik3c2b	4.55	4.61	4.84	4.5
Mlec	5.6	5.01	5.69	4.86
Gm16111	7.92	6.35	7.67	6.4
Gm18779	3.94	4.39	3.91	3.67
Mir3105	3.91	3.63	3.78	4.04
Mir3105	3.91	3.63	3.78	4.04
1700125H03Rik	4.14	4.29	4.04	4.36
Gprasp1	5.56	5.58	5.87	5.22
Gm24627	3.95	3.79	8.57	3.91
Mir487b	3.68	3.99	5.17	4.28
Capn7	5.85	5.96	6.75	5.77
Olfr1202	4.4	4.41	3.73	3.86
Aqp12	3.81	4.3	4.06	4.31
Gm17778	3.81	4.18	4.02	4.41
Gm12792	4.29	3.71	3.97	4.37
Gm17109	4.35	3.91	3.8	4.43
Gm15921	8.06	7.7	8.61	6.22
Gm12338	5.61	4.44	5.05	4.25
4933425L06Rik	4.08	4.32	3.95	4.31
Vmn1r-ps56	3.87	4.42	4.11	4.2
LOC100502866	3.75	3.78	3.35	3.49
LOC100862029	3.75	3.78	3.35	3.49
LOC100504492	3.75	3.78	3.35	3.49

Gm22143	4.01	3.91	3.78	4.36
Gm2431	5.94	5.52	6.58	5.68
Eprs	6.06	5.39	6.43	5.11
4933417G07Rik	4.48	4.11	4.07	4.62
Gm12084	3.85	4.01	3.72	4.51
Gm15268	5.17	5.08	6.14	5.16
Ranbp2-ps8	4.75	4.75	5.47	4.35
Gm7334	4.47	4.5	5.4	4.58
Ptges3	5.76	5.47	6.05	5.36
Agpat5	4.44	4.61	5.04	4.4
Dnajc27	4.86	4.93	5.27	4.68
Gm26606	3.49	3.92	3.36	3.51
Akap8	6.99	6.61	7.35	5.87
Gm23630	4.69	4.74	4.82	5.62
Gm27415	4.16	3.73	3.93	4
Ighv5-17	3.67	4.25	3.95	4.16
Gm26586	4.34	4.46	4.21	5.03
Nuak1	4.98	4.9	5.41	4.71
Gm11222	4.12	4.6	4.43	4.18
Rpl35a-ps2	9.34	9.32	11.52	8.75
4932429P05Rik	4.93	5.08	4.67	5.24
Rb1cc1	4.89	4.73	5.34	4.87
Tmod1	4.62	4.64	5.29	4.75
Usp48	4.87	4.92	5.32	4.86
Xrn1	4.6	4.75	4.97	4.59
Gm20438	5.09	4.73	3.78	4.4
Sycp1	4.39	4.34	4.1	4.45
Gm11694	3.92	4.51	4.08	3.92
Ighv3-6	3.4	3.99	3.94	4.03
Gm11650	4.16	4.45	4.23	4.63
Gm19474	6.15	5.43	8.19	5.16
Serf2	5.43	5.31	5.66	5.15
Apela	3.98	4.12	3.72	4.15
Nap1l4	6.65	5.88	7.14	6.47
Ighv7-1	5.05	6.28	4.61	4.82
Gm12320	4.28	4.45	4.25	4.86
Gm22868	4.46	4.79	4.74	5.02
Olfr102	5.18	4.39	4.12	4.58
Them5	4.19	4.29	3.98	4.29
Gm14008	5.09	4.88	5.55	4.96
Mtor	4.58	4.71	4.93	4.71
Olfr968	4.19	4.23	3.83	3.89
Gm6644	5.34	5.07	5.95	5.29
Gm27343	3.67	3.8	4.09	4.32

Gm14346	4.45	4.52	4.03	4.17
Ccl4	4.05	4.16	3.91	4.34
2700012I20Rik	3.81	3.88	3.51	4.09
Cdk17	6.13	5.83	6.86	5.62
LOC100502805	3.74	3.8	3.34	3.49
C330004P14Rik	4.03	4.35	4.25	4.57
Sbno1	5.08	5.23	5.77	5.08
Mir363	4.01	4.01	4.08	4.46
Sry	5.54	5.34	6.08	5.52
Fat3	4.56	4.81	5.25	4.71
Snord85	4.74	4.51	4.22	4.98
4930548G14Rik	4.26	4.04	3.89	4.35
Gm4657	4.17	4.19	4.11	4.64
Trav6d-5	4.23	4.42	3.87	4.2
Gm3623	4.37	4.81	4.2	4.53
adapt33_4	3.86	3.91	3.56	3.91
Ccdc169	4.03	4.15	3.85	4.27
Gm19803	4.4	4.73	4.17	4.69
Gm12005	6.39	5.8	6.58	5.86
Sema3c	5.05	5.13	5.65	5.19
Gm22925	5.32	4.98	5.86	5.84
Gm14705	4.22	4.13	3.75	4.35
Gm14122	4.65	4.84	4.94	5.07
Vmn1r160	3.92	4.35	4.62	4.07
Hrsp12	4.03	4.09	4.39	4.29
D8Ertd738e	5.97	5.71	7	5.5
5430403G16Rik	4.91	5.18	6.34	4.68
Gm25406	8.12	9.03	8.88	8.6
Papss1	4.56	4.52	5.11	4.69
Rps15a-ps7	10.46	9.58	10.09	7.98
Mir326	4.34	4.27	3.9	4.37
4930503E24Rik	4.12	4.58	3.73	4.48
Slc9a6	5.56	5.04	5.24	5.01
Gm23974	4.07	4.73	4.14	4.09
Golim4	5.39	5.13	5.77	5.35
Itga1	4.75	4.83	5.51	4.64
Gm25166	4.49	4.23	4.77	4.33
Traj8	5.05	5.43	5.95	5.47
Gm15411	4.07	4.1	3.83	4.3
Gm3355	9.49	9.32	10.13	8.43
Gm5060	4.64	5.32	4.77	5.55
Klf7	5.19	5.11	5.54	4.85
Arl13a	4.36	4.22	4.04	4.61
Snord45b	4.85	3.99	3.48	3.68

Olfr109	4.28	3.91	3.89	4.18
Mir21b	4.26	4.05	3.84	4.29
4930488N24Rik	4.55	4.84	4.61	4.78
Olfr1436	4.43	4.64	3.92	4.19
Unc13c	5.65	5.06	5.97	4.84
Stam2	5.05	5.17	5.77	4.92
Mir3068	4	4.34	3.8	4.68
Gm17387	4.32	3.78	4.06	4.48
Adm	3.77	3.87	3.7	4.33
Gm13124	4.62	4.52	4.33	5.07
Gm22175	9.29	9.49	9.85	9.45
Gm13444	4.74	4.96	4.85	5.25
Gm16260	4.36	4.13	4.28	4.63
Gm20433	5.05	5.16	5.23	5.46
Gm19203	4.21	4.53	4.43	4.8
A530010F05Rik	3.9	3.92	3.55	4.13
Gm11618	3.98	4.05	4.31	4
Aqp7	4.29	4.58	4.33	4.68
Rfxap	4.19	4.71	4.55	4.35
Olfr1496	4.42	4.64	4.24	4.06
Gm19894	4.51	4.95	4.21	4.92
Gm884	4.05	4.16	3.73	4.21
Gm4279	3.75	4.18	3.69	4.04
Gm16077	3.61	3.96	4.34	3.79
Lmo3	5.08	5.24	5.77	4.7
Gm12943	7.75	6.57	7.86	5.94
Gm10616	3.71	3.9	3.57	3.79
Gm22021	5.39	5.43	4.87	5.68
Tmem181b-ps	4.32	4.7	4.86	4.61
Ccnh	4.84	4.57	5.35	4.44
Gm24148	4.4	4.8	5.7	4.68
Ssna1	4.99	5.07	5.51	4.93
Bola1	4.09	4.53	4.12	4.34
Cbx3-ps7	6.08	5.47	6.41	5.5
4930598A11Rik	4.43	4.69	4.5	5.13
Glipr2	4.03	4.41	4.33	4.11
n-R5s157	3.74	4.05	3.63	4.58
Gm14155	3.87	3.83	3.92	4.33
Tmem138	4.61	4.32	4.16	4.15
Hnrnpa2b1	7.44	7.76	8.2	7.35
Cplx1	5.39	5.13	5.88	5.81
Gm20345	4.4	4.6	4.15	4.52
Six3os1_7	4.1	4.29	4.05	4.5
G630064G18Rik	4.6	4.26	3.97	4.46

Eogt	5.86	5.36	6.86	5.61
AV051173	4.08	4.33	3.81	4.44
Gm15644	7.99	7.15	8.11	6.04
Fgf21	3.6	4	3.6	3.83
Gm13830	3.97	4.24	3.99	4.37
Sgms1	5.34	5.19	5.92	5.28
Cdc37	5.31	5.01	5.21	4.92
Gm25111	4.23	4.69	4.01	4.78
Gm15352	4.44	4.78	5.47	4.83
Gm8840	5.81	5.92	6.3	5.2
Gm3750	4.07	4.31	3.82	3.9
4930546C10Rik	4	4.41	3.77	4.06
Csprs	4.21	4.43	4.27	4.96
Fam162a	4.03	4.36	4.89	4.08
Fam174a	5.43	4.97	6.2	4.9
Gm14441	5.56	5.36	6.14	5.47
Gm24954	4.17	4.64	4.75	4.29
Ubr4	4.95	4.93	5.31	4.88
Csl	4.09	4.07	4.04	4.48
AA387200	4.16	4.21	3.72	4.21
Gm16355	6.95	6.18	7.53	5.39
Mir466f-4	7.52	6.82	9.81	6.94
Gng12	5.77	5.25	6.25	5.38
Gm22854	6.51	7.92	9.18	7.71
Qars	4.94	5.04	5.44	5.03
Hbb-bh0	5.89	5.34	6	5.12
Tas2r136	4.66	4.33	4.35	4
Mir6917	5.69	6.72	5.93	6.7
Cryba2	3.96	4.09	3.79	4.21
Cadps	5.81	5.99	6.6	5.91
Gm23299	3.74	4.39	3.68	4.02
Dusp21	4.48	4.48	4.01	4.51
Olfr1206	4.12	4.13	3.68	4.09
Fam104a	4.32	4.29	4.82	4.45
A230046K03Rik	4.5	4.75	4.74	4.44
Gm13419	5.44	5.16	4.83	5.07
Sap30	3.75	4.25	4.03	4.02
Rapgef2	5.12	5.25	5.64	4.95
Dstn	6.33	5.23	6.09	5
Obp2b	4.39	4.32	4.2	4.82
Ss18	4.55	4.95	4.86	5.03
Ccpg1os	4.63	4.09	4.03	4.75
Mir744	4.03	4.55	3.99	4.29
Olfr130	4.35	4.15	4.03	4.46

Abcg2	4.84	5.16	5.42	5
Gm23706	5.72	6.08	6.24	6.14
Gm26599	4.09	4.48	3.74	4.8
Gtf2e1	4.21	4.13	4	4.49
Gdf9	3.89	4.17	4.07	4.38
Gm22923	4.48	4.95	5.68	4.74
Gm24593	4.31	4.7	3.83	3.72
Olfr1359	3.74	4	3.65	3.53
Gm14592	4.51	4.32	4	4.62
1700025M24Rik	4.01	4.21	3.97	4.39
Scgb1b29	4.11	4.18	4.58	4.5
mt-Tr	14.09	19.62	19.22	19.62
Rasl10b	5.02	4.72	5.13	4.65
Ush1g	4	4.05	3.8	4.19
Gm26070	5.84	5.65	7.22	5.62
Gimd1	3.83	3.93	4.01	4.24
Bzw1	4.51	4.66	5.32	4.47
1700018C11Rik	3.83	4.12	3.67	3.99
Gm12222	4.93	4.52	5.09	4.45
Gm6664	4.12	4.02	4.23	4.83
Gm15094	3.83	4	3.8	4.24
Gria4	4.79	5.33	5.3	4.93
4933409K07Rik	5.21	5.62	5.89	5.57
4933409K07Rik	5.21	5.62	5.89	5.57
Gm15838	4.87	4.56	5.01	4.5
Trav5n-3	4.01	4.36	3.94	4.13
Trav5d-3	4.01	4.36	3.94	4.13
Gm25410	5.48	4.97	6.52	5.03
Gm14276	4.47	4.15	4.04	4.32
Gm9755	4.43	4.39	4.19	4.04
Ublcp1	5.35	4.68	5.67	4.74
Eomes	4.12	4.32	3.99	4.33
Haghl	4.48	4.69	5.01	4.62
Gng7	5.38	5.53	6.98	4.72
Gm24321	3.72	4.13	3.39	3.9
4930421P07Rik	3.93	3.93	3.62	4.06
1700024P04Rik	3.56	3.91	3.49	4.19
Pts	5.24	5.13	6.3	4.75
Gm26709	3.77	4.4	3.7	4.1
Gm8526	7.12	7.25	7.46	6.99
Cltc	6	5.62	7.11	5.31
Dram2	4.45	4.56	5.65	4.75
Gm25598	4.47	5.25	4.82	4.74
Gm24099	4.19	4.8	4.06	4.82

Ttc39d	3.89	4.13	3.91	4.3
Olfr871	4.31	4.43	4.33	4.74
AI314278	4.28	4.44	4.06	4.58
Snora68	3.93	4.19	4.07	4.62
Gm2436	4.15	4.6	4.02	4.25
Gm2446	4.15	4.6	4.02	4.25
Gm3828	4.56	4.36	4.76	4.3
Skiv2l2	5.19	5.27	5.6	5.14
Snx2	5.69	6	6.55	5.67
Prl7a1	4.38	4.28	4.09	4.59
Dgat2l6	4	4.23	3.85	4.19
Gm13457	4.22	5.11	4.39	5.31
Olfr15	4.36	4.49	4.25	4.77
Ube3a	4.87	4.77	5.02	4.72
Bcan	5.68	5.36	5.98	5.42
Gm26796	4.91	5.01	4.62	4.92
Tppp	5.25	5.44	5.74	5.1
Gm12444	5.82	6.23	6.73	5.85
Gm5560	4.37	5.01	5.2	4.25
Gm25437	5.48	5.63	5.75	5.25
Gm3601	10.1	7.73	11.42	5.76
Gm22055	3.86	4.43	3.76	4.09
Timm44	4.72	4.9	5.03	4.72
Gm11543	4.16	4.41	4.03	4.22
Glipr1	4.23	4.47	3.96	4.25
Mir1192	4.21	4.44	5.12	4.76
Gm17919	4.61	4.15	3.74	4.36
Akirin2	5.43	4.99	5.51	4.87
Cyp2c55	4.11	4.28	3.83	4.21
Olfr609	3.88	4.11	3.9	4.27
Slc25a5	5.26	5.12	6.3	4.99
Gm11800	4.43	4.33	4.1	4.37
Gm5106	3.97	4.39	4.18	4.56
Klhl9	4.31	4.59	4.2	4.46
Gm8545	4.59	4.78	4.18	4.89
Gm20721	4.42	4	4.47	4.13
Gm10688	4.17	4.67	4.22	4.48
Gpr158	4.64	5.26	5.78	4.35
Sucnr1	3.72	3.61	3.54	4.21
Olfr344	3.83	3.83	4.04	4.32
Gm23230	4.68	4.49	4	4.74
Tcp1	5	4.88	5.23	4.93
Gja8	4.16	4.39	4.1	4.52
Fpr1	3.63	3.98	3.47	3.83

Gm14713	4.73	4.39	3.87	4.84
Gm14015	4.22	4.08	3.61	3.71
Hnrnpa3	8.7	8.23	10.12	8.44
Gm14115	4.45	4.42	4.56	3.89
Gm14197	4.05	4.44	4	4.54
Tbx20	3.98	4.15	3.77	4.18
Mir6391	6.37	7.74	8.64	7.82
Usp33	5.41	5.33	6.18	5.15
Rpl34-ps1	6.73	6.13	7.26	5.81
Gm24336	4.36	4.58	5.16	4.32
Zeb2os	4.6	4.52	3.96	4.07
Gm4479	7.09	7.49	8.46	6.85
Aebp2	5.34	5.7	5.8	5.27
Gm24077	4.65	4.37	4.31	4.16
Gm24868	4.65	4.37	4.31	4.16
Gm26847	5.42	5.73	5.06	5.77
Gm16251	5.33	5.37	5.81	5.25
Tmem37	3.85	4	3.62	3.83
Gm26565	4.04	4.39	4.27	4.52
Gm25335	3.89	4.23	3.9	4.87
Gm15704	4.02	3.97	4.1	4.49
Olfr1410	3.8	3.85	3.96	4.34
A630077J23Rik	6.04	5.64	5.99	5.25
Vmn2r-ps38	4.38	4.18	4.15	4.49
Gm13168	5.46	6.25	7.17	6.41
Tprgl	4.92	5.41	5.94	5.43
Ranbp9	6.39	7.54	7.24	5.91
Gm13081	3.69	3.94	3.61	4.09
Uhrf1bp1l	5.45	5.95	5.84	5.36
Gm25427	4.12	4.55	3.68	4.67
Rab20	4.26	4.44	4.21	4.52
Gm12486	4.11	4.35	4.02	4.66
Cebpe	4.13	4.18	3.64	4.39
Necap1	4.88	4.59	5.03	4.58
Phactr1	6.07	5.83	6.44	5.64
Ocln	5.02	5	5.36	4.73
Gm19998	4.15	4.55	4.05	4.13
Gm13606	4.92	4.59	4.77	4.97
Jmjd1c	5.39	5.38	5.99	5.28
Gm20747	4.48	4.44	4.75	4.17
Thsd7a	4.55	4.49	5.04	4.55
Mir654	3.95	3.96	4.42	3.69
Gcsh	5.58	5.69	6.05	5.52
Gm15484	8.4	7.67	8.04	6.05

Atp4b	4.25	4.33	4.06	4.59
Ttc3	6.62	6.57	7.21	5.99
1700083H02Rik	3.89	4.14	3.9	4.39
Gm7714	3.68	4.49	3.88	4.14
Gm13793	4.51	4.68	4.37	4.75
Mirlet7c-1	4.24	4.23	4.44	4.92
Gm3625	6.15	6.03	8.43	5.57
Gm24606	5.26	5.49	5.88	5.27
Adprm	4.96	4.83	5.08	4.58
Vmn2r-ps84	4.03	4.67	3.96	4.23
Arpc1a	5.64	5.63	6.08	5.4
Gm14602	5.76	6.23	6.58	6.09
Gm10175	6.48	6.21	7.6	6.06
Tmprss11a	4.07	4.02	3.83	4.14
Diaph2	6.12	5.46	6.72	5.33
Gm24494	4.02	3.97	3.61	3.73
Gm5250	3.79	3.98	4.28	4.23
Gm8138	4.58	4.44	4.15	4.76
Gm10787	4.59	4.43	4.64	4.96
Mir7013	4.61	5.21	4.79	4.69
Gm5258	4.67	4.49	4.51	5.26
Igkv14-126	4	4.09	3.89	4.52
1700091H14Rik	4.61	4.46	4.59	4.9
Thoc7	5.27	4.86	5.53	4.59
Mir467a-3	4.7	5.16	5.44	5
Mir467a-6	4.7	5.16	5.44	5
Mir467a-10	4.7	5.16	5.44	5
Gm20467	4.8	5.08	4.85	5.08
Gm25876	4.6	4.28	4.99	4.76
Gm11846	4.83	4.63	4.7	5.16
Mir7681	4.68	4.29	4.02	5.06
5-Mar	4.56	4.39	4.97	4.31
Gm25622	3.94	4.36	4.02	4.34
Mir465	4.62	4.14	3.85	4.41
E330034G19Rik	4.21	4.38	4.27	4.74
Mir1983	4.9	5.42	4.92	5.44
Gm14308	6.42	6.33	7.05	6.05
Gprin1	4.4	4.57	4.39	4.64
Abhd15	4.04	4.09	3.89	4.34
Gm15579	4	4.27	3.86	4.08
Gcnt7	4.3	4.61	4.55	4.4
Ndufs3	5.9	6.04	6.23	5.53
Bpifa3	3.86	4.08	3.89	4.19
Gm20073	4.63	4.52	4.88	4.12

Rbm27	5.05	5.06	5.48	4.94
Rassf4	4.03	4.17	3.84	4.18
Utp14b	6.52	5.55	6.65	5.35
Gm20849	4.58	4.69	4.22	4.79
Gm19463	3.81	3.75	4.15	4.02
Rftn1	4.43	4.74	4.52	4.43
Gm15274	3.86	4.26	4.11	3.89
Hoxa1	4.03	4.17	3.81	4.31
Tug1	4.94	4.88	5.6	4.91
Xpo7	4.69	4.69	5.04	4.53
Gm10031	7.07	5.39	6.94	5.3
Zfp934	4.67	5.19	4.92	4.47
Nono	6.38	5.67	6.14	5.49
Dthd1	4.2	4.17	3.82	4.34
Defa-ps7	4.59	4.44	4.47	3.92
Ddx3y	4.35	4.26	4.18	4.51
Cdk19os	3.63	3.86	3.64	3.86
Bcas3	4.91	4.79	5.2	4.74
Olfr1454	4.13	4.31	3.67	3.87
Olfr450	4.12	4.17	3.71	4.02
Mir32	3.74	3.91	3.64	4.06
4930544G11Rik	4.7	4.48	4.18	4.65
2310034O05Rik	4.04	4.41	4.06	4.35
Gm15565	4.02	4.1	4.11	4.61
SMAD5-AS1_1	4.28	4.6	4.31	4.86
Lce1a1	4.15	3.91	3.88	4.9
Mir297-1	11.67	12.89	16.7	12.64
Gm25317	4.31	4.91	4.5	5.27
Gm16035	4.4	4.79	4.39	4.59
Gm19279	4.26	4.03	3.94	4.4
Mdm4-ps	3.95	4.51	4.12	4.23
Gm11703	10.27	9.93	10.84	8.17
Snord14e	4.9	7.59	3.87	4.3
Gm22966	4.25	5.32	4.53	5.06
Gm21795	4.25	4.21	3.79	4.08
Atxn3	4.47	4.6	4.88	4.59
Gm14964	6.34	6.35	6.48	6.11
Olfr766	3.89	3.98	3.59	4.03
Gpr171	3.98	4.14	4.23	4.53
Plin3	4.27	4.23	4.79	4.19
Hmgcs1	4.3	4.87	4.47	4.42
Gm11889	4.89	4.82	4.74	4.4
Gm8662	6.63	6.1	7.21	4.72
Fbxl5	4.46	4.55	5.1	4.53

Gm19868	6.04	5.11	6.71	5.05
Gm14976	4.4	4.5	4.33	4.97
Gm15512	4.21	4.34	3.94	4.51
Gm12469	8.79	6.91	9.04	6.55
Gm17709	4.52	4.97	5.68	4.17
Zic1	5.14	4.88	5.58	4.96
Snora62	4.35	3.83	4.05	4.99
LOC100861998	4.23	4.31	4.14	4.55
Dtd2	4.81	4.81	5.52	5.07
Mir466o	8.63	7.63	9.99	4.94
Vmn1r-ps128	3.95	4.26	3.75	4.03
Twistnb	4.35	4.8	4.65	4.36
1700019A02Rik	4.57	4.3	4.18	4.73
Mat2b	4.67	4.99	4.96	4.87
Pih1d2	4.13	4.22	3.97	4.34
Gm6104	5.06	4.9	5.6	5.23
Crygn	3.84	4.09	3.68	3.94
Ighv8-13	5.52	5.14	4.69	5.02
Gm27410	4.42	4.41	3.86	4.37
Gm5422	4.8	5.02	5.47	4.68
Mir653	3.98	4.26	3.82	4.23
Sash1	5.19	4.99	6.15	5.4
Olfr619	4.07	4.02	3.56	4.15
Sntn	4.08	4	3.74	4.22
Olfr846	4.07	4.71	3.79	4.29
Tdpoz4	3.98	4.35	3.96	4.32
Gm4862	4.02	4.04	3.52	3.91
Scn2b	5.36	5.46	5.93	5.56
Gm16160	3.92	3.92	3.67	4.01
A630023A22Rik	4.08	4.07	3.85	4.27
Krtap21-1	4.27	5.04	4.31	4.28
Rgs8	4.77	4.8	5.07	4.7
Gm25102	4.59	4.79	4.51	5.23
Gm5477	4.2	3.86	3.85	4.23
4933427G17Rik	3.97	4.08	3.81	4.09
Gm13182	5.16	5.59	5.72	4.95
Gm11443	5.82	5.56	6.09	4.88
Zfp407	4.6	4.53	4.97	4.56
n-R5s154	3.84	4.02	3.82	4.37
LOC100862129	4.44	4.39	4.61	4.86
Tgfbr2	5.01	4.88	5.7	4.71
Gm19965	4.65	4.74	4.61	5.15
Adrb2	4.17	4.92	5.73	3.99
Stam	4.8	5.21	5.43	4.85

Gm20678	3.75	5.19	4.68	4.56
Ighv1-50	3.54	4.73	3.97	4.13
Gm23873	3.86	4.11	3.7	4.46
Olfr193	4.45	5.13	4.49	4.91
Cdkl3	4.81	4.95	5.21	4.82
Cdc123	4.82	4.92	5.29	4.74
Rbm26	5.08	5.11	5.62	5.28
Crygc	4.28	4.28	3.86	4.36
Mir466f-3	8.31	7.65	11.32	7.29
Camk4	5.67	5.58	6.86	5.72
Gm22908	5.84	6	6.51	5.87
Gm21924	4.42	4.35	3.91	4.57
9330162012Rik	3.96	4.36	3.78	4.01
Gm14510	4.57	4.62	4.8	5.05
Gm1141	4.29	4.22	3.94	4.33
Gm23502	5.61	5.37	5.49	5.11
Dpp3	4.61	4.73	4.72	4.98
LOC100861719	4.77	4.28	4.15	4.81
Zfp683	4.17	4.14	3.82	3.97
Trav13n-4	4.69	4.45	5.45	4.48
Dennd5b	4.77	4.91	5.23	4.96
2310035C23Rik	4.35	4.44	4.75	4.39
Gm24668	4.07	4.48	3.86	4.41
Hnrnpu	5.48	5.33	5.92	5.42
Gm14128	4.48	4.39	4.14	4.75
Ube2d2b	4.1	4.38	4.07	4.43
Zfp644	5.41	5.52	5.75	5.38
Gm15132	3.72	4.1	3.87	4.27
Adcy5	5.74	5.38	6.15	5.27
Plxna4os2	3.87	4.13	3.91	3.77
Actr1a	4.79	6.19	5.57	5.42
Gm5570	4.66	4.49	4.13	4.38
Gm26704	3.52	4.58	3.82	4.43
Mir574	5.04	5.25	5.88	5.17
Gm7271	4.43	4.01	3.86	4.27
Herc1	5.38	5.51	6	5.28
Gm26306	3.94	4.43	4.2	4.57
Uxs1	5	5.55	5.23	5.05
Gm24890	4.59	5.16	5.31	4.68
Klra9	4.48	4.27	4.07	4.5
Gm7076	4.55	4.2	4.63	4.5
Errfi1	4.51	4.33	4.99	4.64
Gm11984	4.09	4.75	4.04	4.51
Arxes2	5.05	4.95	6.59	4.29

Lin52	4.42	4.47	4.1	4.74
Mir466h	9.37	6.4	10.37	6.86
Psenen	8.09	6.39	7.09	6.15
Gm14372	4.57	4.43	4.06	4.51
Gm5073	4.53	4.16	4.13	4.86
Gm25642	4.3	4.67	4.64	4.45
Gm26653	3.84	4.27	3.73	4.32
Zfp488	4.06	3.87	3.72	4.27
Atg4a	4.85	4.61	5	4.5
Gm26882	4.39	4.72	4.09	4.19
Gm20558	4.13	4.03	3.63	3.79
Gm23666	5.92	5.66	6.13	5.74
Olfr937	3.94	4.53	3.87	3.98
Josd1	4.9	5.12	5.49	5.22
Gm22677	5.16	4.92	6.06	4.85
LOC100862175	4.9	4.4	5.51	4.14
Olfr617	3.65	3.91	3.65	4.26
Cdo1	5.81	4.89	5.3	5.19
Olfr1302	4.04	4.41	4.01	4.49
Gm7292	5.53	5.3	6.24	5.28
Alg8	4.45	4.68	4.74	4.74
9330199G10Rik	4.18	4.4	4.37	4.02
Gm3839	5.74	6.06	6.63	5.36
Gm14325	6.58	5.49	6.68	5.49
Nrp1	4.98	4.64	5.11	4.86
Gm5528	7.67	7.15	8.2	6.72
Gm13868	4.77	4.52	4.93	4.27
Gm11278	3.96	4.05	4.05	4.45
Rps6ka2	5.1	4.82	5.24	4.9
Gm11668	3.59	4.06	3.63	3.97
Fermt2	5.43	5.18	5.72	5.15
Gm8232	4.6	4.48	4.19	4.55
Rtcb	4.84	5.01	5.22	4.75
Hmx2	4.47	4.33	4.63	4.6
Nr2c2	4.71	4.92	5.29	5.04
Gm25915	4.51	4.73	4.78	3.99
Mir367	3.82	3.95	3.65	4.01
Gm5394	4.31	4.27	4.06	4.57
Abat	5.64	5.41	5.95	5.33
Evi5	4.87	5.1	5.2	5.01
Rsf1	5.35	5.33	5.76	5.3
Ighv2-6-8	3.98	4.11	3.74	3.77
Cwc15	5.91	5.59	6.17	5.13
Olfr1458	4.1	4.24	4.07	4.63

Adam23	5.72	5.54	6.03	5.34
A430078I02Rik	4.4	4.33	4.32	4.54
Gm14284	6.57	5.29	6.62	5.55
Mir7220	4.38	4.66	4.28	4.14
Mup8	4.19	4.3	4	4.49
Gm21717	4.57	4.39	4.02	3.84
Tmem47	5.93	5.42	6.65	5.28
Gm17076	4.18	4.22	3.92	4.7
Gm22368	4.32	4.48	4.49	4.88
Mpst	3.87	3.8	3.62	4.06
Rgs4	6.22	7.17	8.34	6.91
Cacna2d2	4.55	4.48	4.92	4.5
Zfand3	5.01	4.62	5.28	4.55
Gm20730	3.79	3.92	3.85	4.18
Elf2	4.96	4.88	5.29	4.91
Csnk1g1	4.84	4.65	5.11	4.73
Gm26779	4.9	5.02	5.31	4.83
Gm14690	4.15	4.1	3.98	4.39
Pptc7	4.37	4.29	4.81	4.27
Gm25761	3.82	4.12	4.07	4.45
Mtch2	4.61	4.76	5.04	4.73
Nub1	4.74	4.98	5.28	5.01
Gm23501	4.37	5.22	4.64	4.6
Gm13459	4.12	4.58	3.9	4.43
St8sia6	4.82	4.96	5.2	4.77
Olfr836	3.85	4.56	3.68	4.6
Gm12267	4.44	3.92	4.64	4.04
4930599N23Rik	4.2	4.27	3.91	4.23
Krtap7-1	3.88	3.81	3.67	4.05
Creg2	4.36	4.52	4.54	4.81
Vmn2r28	4.21	4.21	4.05	4.46
Zfr	5.96	5.55	6.22	5.47
Vmn1r-ps120	4.09	4.18	4.02	4.74
Lipo4	4.33	4.67	4.71	4.24
Gm23311	4.37	4.48	4.81	4.52
A930018M24Rik	4.28	4.15	4.2	4.5
Gm15678	4.65	4.76	4.78	5.28
Adnp	5.11	4.88	5.28	4.82
Ubqln2	4.23	4.01	5.06	3.87
Myt1l	5.64	5.74	6.22	5.24
Gm23390	4.85	4.92	4.4	4.51
Gm12665	4.22	4.41	4.16	4.47
4930565D16Rik	3.9	4.11	3.8	3.95
Gm26396	4.3	4.53	4.41	5

Arpc5l	4.55	4.62	5.07	4.5
Gm12760	6.19	5.94	6.07	5.06
Gm16445	3.97	3.92	3.5	3.96
4932438A13Rik	5.49	5.49	5.89	5.17
Gm12372	4.33	5.9	5.79	4.1
Aftph	5.33	5.25	5.52	4.99
Pfdn2	6.62	6.34	6.8	6.27
Gm20099	4.54	4.66	4.14	4.99
Vmn2r57	4.55	4.17	4.39	4.65
Tssk1	3.94	3.7	3.53	3.54
Gm24216	4.26	4.31	4.63	4.07
Gm15229	3.91	4.15	3.75	4.26
Gm7857	6.34	5.89	6.08	5.6
Med13	5.78	6.01	6.85	5.83
Gm12117	4.51	4.82	5.64	4.37
Snx13	4.5	4.43	5.17	4.57
Gm2214	7.02	6.84	7.77	6.07
Arhgef7	5.28	4.97	5.63	4.99
Gm13521	3.83	4.06	3.45	4.22
Snai1	3.78	4.04	3.81	3.89
Gm10146	5.56	5.89	6.16	5.03
LOC100504971	5.39	4.94	5.16	4.55
Gm21818	4.09	4.48	4	4.02
Gfm1	4.6	4.76	4.92	4.7
Svs4	4.35	4.48	4.49	4.82
Gm14992	4.52	4.81	4.7	4.78
AI314180	4.96	5.03	5.34	4.62
Gm20004	4.67	4.19	4.64	4.32
Gm11969	7.38	5.04	6	5.13
Gm10377	4.89	4.73	4.48	4.33
Trbv10	3.91	3.74	3.79	4.25
Rimkla	4.69	5.09	4.93	4.92
Gm22455	4.66	4.71	5.01	4.36
Gm13672	4.06	4.28	3.81	4.39
M6pr-ps	4.78	4.3	4.34	4.17
LOC100862086	6.93	6.81	9.18	6.86
Tmem131	4.98	5.19	5.98	5.53
Gm21396	4.03	4.35	4.12	3.86
A630019I02Rik	4	3.87	3.6	3.9
Mir452	4.37	4.25	3.64	4.01
Kdm5a	5.04	4.94	5.4	4.72
Mup-ps17	4.35	4.14	3.84	4.57
Hmox1	4.02	4.01	3.82	4.15
Olfr57	4.29	4.25	4.03	4.53

Gm14929	7.4	6.09	7.87	5.19
Gm14110	5.26	4.89	4.8	5.47
Tsta3	4.7	4.99	4.97	4.68
Gm20115	4.28	4.59	4.33	4.99
Slc10a1	4.11	4.09	3.89	4.13
Gm17449	5.23	5.16	5.96	5.18
Bub3	5.8	5.95	6.96	5.6
Olah	4.22	4.08	3.89	4.44
Epyc	4.2	4	3.99	4.19
Gm13852	4.99	5.31	4.16	4.67
Olfr1510	3.87	4.06	3.97	4.34
Gm11667	3.9	4.05	4.04	4.25
Gm13560	3.91	3.96	3.87	4.81
Sftpd	4.28	4.61	4.22	4.63
Ighv5-12-4	7.7	8.14	7.37	7.97
Gm24279	5.58	5.78	6	5.4
Ackr4	3.8	4.13	3.72	4.06
Gm16541	4.19	4.02	3.79	4.04
Bach1	4.7	4.77	5.08	4.71
Olfr670	4.49	4.58	4.55	5.34
LOC380994	4.66	4.74	4.46	4.92
LOC380994	4.66	4.74	4.46	4.92
Pin1rt1	3.99	4.24	3.8	4.15
Gm5725	4.03	4.36	4.05	4.61
Gm5725	4.03	4.36	4.05	4.61
Gm25901	4.22	4.51	4.23	4.74
Gm6468	3.88	4.24	3.82	4.44
Gm15215	4.08	3.75	3.62	4.24
Gm12398	4.56	4.7	5.01	5.04
Gm3233	4.81	4.3	4.49	4.2
Tspan1	4.19	4.41	4.02	4.14
Gm19425	7.92	7.1	8.66	5.86
Gm24626	5.25	5.54	5.44	5.83
Meis2	6.69	6.16	7.22	5.83
Gm26923	4.47	4.39	4.25	4.76
Gm15613	3.94	3.94	3.44	3.99
Arpp19	5.45	5.27	5.96	5.09
Gm19831	10.09	7.18	12.16	7.32
Dusp23	4.49	4.61	4.04	4.37
LOC100861907	4.12	4.6	3.76	4.15
Azin1	4.62	4.98	5.08	4.97
Gm21778	4.24	4.46	3.82	4.28
Vmn1r19	3.9	4.37	3.79	4.07
Coprs	5.78	5.45	5.99	5.52

Vmn1r168	4.26	3.93	4.09	4.9
Gm11567	4.25	4.41	3.77	4.8
Gm21786	4.1	4.36	4.19	4.74
Nat3	4.75	5.03	4.64	5
Gm5039	5.39	5.17	5.11	5.58
Rpl13a-ps1	5.4	4.76	5.38	5.07
Gm27178	5.27	5	5.95	4.93
Rpl5-ps2	4.57	4.25	5.01	4.68
Zeb2os	4.36	3.92	3.75	4
Morf4l1-ps1	4.49	4.9	4.48	4.36
Chrdl1	4.22	4.4	4.18	4.68
Prps1	7.12	5.85	7.11	5.59
1700064J06Rik	4.19	4.69	4.19	4.41
Gm26981	3.93	4.1	3.38	3.86
Entpd4	5.07	5.16	5.39	4.87
Gm14389	5.32	4.96	5.5	5.36
Top2b	4.85	5.24	5.34	5
Vmn1r55	3.77	4.08	3.58	3.99
Krit1	4.44	4.82	4.93	4.59
Gm19935	4.76	5.28	4.34	4.86
Gm23536	3.96	4.39	3.78	4.19
Gm4907	3.99	4.11	3.67	4.05
Gm26457	3.61	4.21	3.46	3.9
Gm14431	6.06	5.55	6.7	5.67
Gm14431	6.06	5.55	6.7	5.67
Rnase12	4.4	4.62	4.5	4.98
Anapc10	5.12	4.88	6.51	4.76
Gm4524	3.91	4.49	4.08	4.11
Gm9703	14.95	14.55	14.77	10.63
Gm3750	4.75	4.76	4.22	4.4
Rpl30	5.5	5.44	5.48	4.86
Gm20747	4.21	4.37	4.26	3.93
Gm11274	4.82	4.65	4.46	4.64
Gm25365	4.58	4.32	4.54	5
Gm26600	3.94	4.27	3.85	4.2
Bsph2	4.65	4.63	4.85	4.48
Fxn	4.3	4.24	4.84	4.75
Gm16675	4.27	4.42	4.69	4.53
Gm26464	3.97	4.42	4.06	4.02
Gm9913	3.39	3.91	3.34	3.55
Gm14240	6.23	6.86	7.02	5.88
Gm26177	4.66	4.86	4.43	5.12
Utp11l	6.04	5.45	6.15	5.28
Tlk2	5.33	5.69	5.56	5.49

Gm26836	4.26	4.86	4.25	4.59
Gm10182	7.24	5.33	6.94	5.19
Psmc6	5	4.82	5.42	4.86
Cntn1	5.75	5.68	6.22	5.66
Trappc11	5.19	5.39	5.55	5.07
n-R5s45	4.05	3.92	3.78	4.13
Tial1	4.99	5.24	5.32	4.98
Gm25629	3.79	4.58	3.86	4.35
Gm24588	5.08	5.41	6.28	4.97
Psmc5	6.48	6.17	7.06	6.03
Gm25631	4.85	5.47	5.82	5.18
Gm10130	4.36	4.57	5.22	4.7
1700071M16Rik	4.03	4.14	3.94	4.22
Gm24935	4.05	4.52	4.29	3.93
Rpl21-ps14	8.88	8.14	9.16	6.92
LOC100043315	4.22	4.33	4.01	3.89
Gm25853	4.25	4.23	4.08	3.84
Olfr434	4.06	4.06	3.7	4.36
Ube2n	4.97	5.02	5.16	4.51
Gm22724	5.21	5.58	5.15	5.64
Gsdmd	4.54	4.44	5.13	4.52
Mylk3	4.14	4.39	4.23	4.45
Gm25267	4.06	4.05	3.64	4.53
Gm23196	4.55	4.58	4.52	5.26
P2ry1	4.13	4.47	4.23	4.46
Mir297a-2	7.1	6.94	9.68	4.96
Gm24463	4.72	5.61	7.11	4.86
Gm22932	5.01	4.91	5.72	5.01
Prdm13	4.02	4.18	3.75	4.08
Gm22829	4.54	5.47	4.92	4.56
Fam189a2	4.1	4.26	5.17	4.14
Mrpl36	3.87	3.93	3.9	4.15
Asphd2	6.31	4.82	5.68	5.24
Anp32-ps	5.04	4.39	4.88	5
A530088E08Rik	5.16	5.96	5.59	5.54
Pdgfd	4.25	4.83	4.64	4.31
Lgals8	4.57	4.71	5.38	4.54
Mir5709	3.98	4.18	3.85	4.42
Clasp2	5.27	5.27	5.52	4.89
Gm12120	4.05	4.07	3.95	4.3
Kcnq5	4.87	5.27	5.53	5.08
Rxfp1	4.51	4.41	4.08	4.28
Gm25883	4.01	4.13	3.92	4.54
Gm12146	3.99	4.1	3.72	4.23

Gm11842	4.17	3.97	3.65	4.11
Gm10647	4.33	4.12	3.94	3.74
Gm8172	11.06	8.12	11.19	7.63
Gm25371	4.69	4.71	4.61	5.13
Mir374c	4.23	4.51	3.58	4.47
Ereg	4.07	4.3	3.92	4.23
Pik3c3	4.97	4.76	5.17	4.65
H2-DMb1	4.32	4.38	4.01	4.54
Gm7790	4.14	4.24	4.1	4.57
Gm8068	4.56	4.56	4.48	4.79
Erg	4.3	4.47	4.64	4.5
Gm22205	9.42	8.72	8.85	4.31
Gm4219	4.17	4.62	4.54	4.61
Lce1e	3.8	4.2	4.1	4.8
Gm14500	6.57	6.06	6.06	6.53
Vmn2r58	4.14	4.72	4.01	4.08
Gm20320	3.8	4.04	3.62	4.19
Gm23709	5.19	5.07	6.08	5.26
Gm22967	4.5	4.58	4.54	5.12
1700010K24Rik	4.55	4.47	4.06	4.54
Rab2a	8.11	6.46	8.55	6.28
Gm15371	4.16	4.29	4.37	4.93
Gm13471	4.28	3.95	3.92	4.49
Lrrc34	4.24	4.24	3.96	4.2
Gm5763	4.97	5.52	4.88	5.07
Zfa-ps	4.37	3.91	3.84	4.2
4930486I03Rik	4.2	4.09	3.82	4.33
Il18	4.44	4.76	5.03	4.51
C330022C24Rik	3.81	4.35	4.04	4.07
Gm24106	4.6	5.55	4.82	4.59
Gm23790	4.6	5.56	5.08	4.8
Gm11978	3.95	4.17	3.83	4.1
Thsd1	5.17	4.98	5.92	5.22
Epo	4.46	4.4	4.13	4.58
Atp2b4	10.12	10.78	10.97	10.79
Baat	4.44	4.24	3.86	4.43
Gm19660	6.73	6.13	8.23	6.1
Olfr866	4.33	4.91	4.34	4.99
Gm15978	3.88	4.15	3.88	4.4
Stard3nl	4.57	4.39	4.68	4.42
Olfr808	3.96	4.33	3.83	4.26
1810018F18Rik	4.39	4.29	4.18	4.82
Gm14661	4.29	4.43	4.1	4.29
Gm21801	4.45	4.22	3.77	4.62

Gm21914	4.45	4.22	3.77	4.62
LOC100862026	3.87	3.84	4.26	4.34
Gm14531	3.88	4.03	3.71	4.2
Mir1190	5.44	5.88	5.95	5.06
Lsm5	4.86	4.82	4.54	4.88
4933400B14Rik	5.08	4.41	4.27	4.22
Gm13361	4.1	4.31	3.79	4.18
Ncs1	4.76	5	5.63	5.2
Ucp3	4.13	4.14	3.84	4.27
Gm22378	4.47	4.57	4.49	4.82
Gm12973	4.06	4.48	4.41	4.45
Serpina1b	4.16	4.17	3.87	4.31
LOC100861795	4.1	4.49	4.68	4.7
1700016K19Rik	7.28	7.28	7.21	6.87
Trav11d	5.62	5.54	6.15	5.64
Klhl14	4.47	4.6	4.29	4.94
Olfr461	4.1	4.33	3.93	4.07
Zfp622	4.4	4.34	5.04	4.45
Vmn1r-ps15	3.75	4.33	3.67	3.93
Gm10509	4.53	4.6	4.06	4.41
Psme2b	5.88	6.07	7.44	5.79
Mphosph8	4.88	4.96	5.2	4.77
Gm24061	3.93	4.09	3.72	4.08
Trdv1	3.92	4.4	3.93	3.99
Nfat5	4.99	5.13	5.37	4.95
Gm25487	5.02	4.67	4.45	4.58
Vezf1	6.13	5.88	6.95	5.95
Gm5274	4.51	4.36	4.26	4.84
Gm15550	4.39	3.8	4.34	4.66
Vmn2r-ps13	4.03	4.22	4.16	4.53
Gm11940	4.06	3.96	4.04	4.58
Rmdn3	5.11	5.1	5.5	5.3
Olfr1135	3.85	3.84	3.72	4.15
Pcdhb13	4.23	4.46	4.27	4.8
Nfe2l3	4.18	4.5	4.21	4.43
Gm19956	3.63	3.93	3.85	4.2
Gm24679	4.65	5.27	5.08	5.23
4930433I11Rik	3.78	4.09	3.65	4.23
Gm19986	3.68	4.1	3.88	4.17
Gm13331	6.01	6.41	6.75	5.36
Prr16	3.92	4.41	3.93	4.24
Vmn1r191	4.33	4.5	3.97	4.76
Rimklb	4.74	4.77	5.28	4.78
Rbms2	5.25	5.23	5.81	5.46

Sec23a	4.74	4.64	5.09	4.65
Gm15359	4.25	4.73	4.18	4.35
Gm24048	5.82	5.85	5.9	5.67
Asb3	4.18	4.35	4.08	4.29
Gm15709	4.76	4.88	5.23	5.54
Gm24767	8.19	8.28	8.42	8.93
C1qtnf1	4.37	4.34	4.13	4.44
Cldnd2	4.31	4.18	4.29	4.53
Bcr	5.89	5.45	6.25	5.09
Dennd4c	4.77	4.7	5.15	4.7
Strbp	5.86	5.93	6.2	5.7
Gm25345	3.82	4.34	3.73	3.98
Olfr615	3.88	4.3	3.77	3.88
Gm22018	4.3	4.42	4.71	4.18
Pex3	4.5	4.48	4.81	4.57
Vmn2r-ps49	4.32	4.77	4.26	4.74
Olfr901	3.93	3.99	3.59	4.09
LOC100862318	6.64	5.39	6.85	5.81
Gm15318	4.1	4.2	4.11	4.39
4930511A02Rik	4.01	4.61	4.11	4.76
Gm9938	4.2	4.27	5.04	4.39
Olfr955	3.97	4.31	3.78	4.08
Gm6397	8.7	8.74	9.92	9.21
BC048507	4.39	4.6	4.03	4.43
Gm6421	7.38	7.36	7.98	7.22
Olfr727	4.49	4.24	3.96	4.21
Gm12946	4.13	4	3.88	4.35
Olfr281	4.34	4.33	3.85	4.28
Ccl12	3.9	4.27	3.96	4.5
Zp3r	4.05	4.09	4	4.34
Gm8652	5.62	5.16	5.99	5.53
A330049N07Rik	4.49	4.64	4.65	4.96
Gm18006	4.16	4.43	3.92	4.66
n-R5s210	4.1	4.18	4.15	4.58
Rps6-ps2	6.6	5.39	6.41	5.56
Gm24195	3.93	4.1	4.28	4.14
Rpl9-ps3	4.57	4.22	4.79	4.48
Gm25599	4.56	4.54	4.12	4.19
Gm15749	4.39	3.98	3.91	4.4
Redrum	4.2	4.15	3.99	4.55
4932411N23Rik	4.07	4.43	3.85	4.39
Olfr474	4.23	4.28	3.96	4.42
Krtap3-3	3.9	4.17	3.84	3.84
Clk3	5.69	5.3	6.53	5.14

Sec61g	5.43	5.37	5.83	5.12
Arhgap27os2	4.39	4.25	4.14	4.35
Brcc3	4.55	4.7	5.16	4.43
Itlnb	3.75	3.65	3.69	4.26
Olfr849	4.56	4.46	4.1	4.59
Ccng1	5.39	5.39	5.84	4.73
Vmn1r192	4.72	4.6	4.13	4.56
Pafah1b2	5.24	5.57	5.9	5.5
Spint5	4.26	4.48	4.04	4.76
Olfr147	4.28	4.19	4.27	4.93
Eif4e	5.52	5.72	7.12	5.58
5430434I15Rik	3.81	3.84	3.55	4.01
Trmt12	3.97	3.92	3.69	4.01
Pea15b	4.46	4.43	3.94	4.48
2310050C09Rik	3.68	3.91	3.53	3.9
Cstb	5.61	5.13	4.95	5.41
Gm22608	5.84	5.93	5.59	6.39
Gm5900	4.46	4.59	5.02	4.47
Siah3	3.78	3.9	3.67	4.34
Gm5873	4.09	4.46	3.8	4.14
Lims1	4.67	5.03	5.41	5
Gm11810	8.12	7.6	8.37	7.39
Gm27332	5.33	4.97	5.38	4.57
Taok2	5.15	5.16	5.5	5.18
Ppp1r42	4.49	4.26	4.1	4.59
Gm26904	9.78	9.18	10.46	9.39
Acrbp	4.73	4.86	5.24	4.88
Gm13373	4.35	3.97	3.89	4.31
Inpp1	4.46	4.56	4.72	4.41
Gm18486	3.81	4.18	4.12	4.01
n-R5s166	5.35	5.5	5.39	5.82
Gm19363	4.04	4.71	4.66	4.42
Gm7735	5.85	5.61	6.68	5.34
Gm4596	4.84	4.81	5.93	4.53
Cmtm4	4.45	4.7	4.97	4.77
Trbv15	4.6	4.5	5.18	4.38
Mir133a-2	3.82	3.87	3.53	3.95
Tmem86b	4.05	4.15	3.83	4.1
Gm12816	5.5	5.35	6.05	5.28
Slc17a2	4.06	4.21	4.08	4.38
Vmn2r-ps70	3.93	4.26	3.68	4.15
Gm24669	4.87	5.4	5.41	5.12
Gm14520	4.61	5.06	5.15	5.04
Trav12n-2	3.98	4.17	4	4.27

Gm19976	5.47	4.54	5.58	5.28
Appbp2	4.46	4.43	4.89	4.53
Usp16	4.8	4.86	5.33	4.84
Gm11565	3.99	4.37	3.98	4.15
Sugt1	5.06	4.93	5.51	4.59
Gm22441	7.56	7.49	8.93	8.04
Snai2	3.8	4.19	3.93	4.09
1700024N05Rik	4.16	4.37	4.43	4.65
Gm12773	4.26	4.36	4.11	4.46
Gm16021	6.6	5.74	6.72	5.57
Yipf7	4.15	4.31	4.05	4.66
Gm23077	6.39	6.49	6.92	6.71
Mir6380	5.8	4.76	5.6	4.58
Zfp653	4.84	4.33	4.81	4.44
Gm26722	4.33	4.6	4.21	4.62
Runx1t1	4.83	4.58	5.29	4.75
Cyp2b23	4.02	4.21	3.9	4.13
Kctd19	4.05	4.12	3.93	4.21
Gm27989	9.99	9.44	10.02	8.76
Gm22725	4.02	3.88	3.46	3.76
Sycn	3.81	3.72	3.74	4.09
Mrpl47	4.74	4.67	5.58	4.93
Gm24510	4.69	6.4	6.55	5.38
Asphd1	4.35	4.64	5.16	4.59
Gm10417	3.85	4.21	3.69	4.05
Gm6594	7.17	6.84	7.42	7.19
Tex37	4.7	4.95	4.74	5.31
Gm8880	6.07	5.14	6.72	4.81
Mir743b	3.84	3.81	3.71	4.42
4930509J09Rik	4.73	4.41	4.4	4.58
Ctxn1	5.73	5.78	6.02	5.4
A230006K03Rik	5.69	5.88	6.28	5.9
Nedd4	7.91	7.17	8.53	7.04
Enpp2	5.18	5.3	5.56	5.15
Morf4l1	6.38	6.22	6.83	5.42
Gm25224	7.4	7.57	9.08	7.98
Gm19475	4.58	4.63	5.19	4.11
Hoxb13	3.89	3.77	3.58	3.79
Efcab3	4.2	4.36	4.13	4.41
Gm25117	6.98	7.9	7.47	6.73
Ndufaf7	4.65	4.59	4.97	4.7
2010300F17Rik	4.36	4.26	4.22	4.74
Gm20881	4.12	4.33	4.21	3.91
Gm21617	4.12	4.33	4.21	3.91

Gm22243	8.44	8.7	11.05	8.5
Fam26d	3.61	4.24	3.61	4
Gm6177	6.83	5.93	7.24	5.86
Gm23381	4.18	4.25	3.68	3.99
Hipk2	5.24	4.8	5.35	4.9
LOC100862193	5.64	5.61	6.61	5.84
Hoxb4	4.17	4.39	4.01	4.52
Nucks1	6.69	6.62	7.28	5.93
Gm20120	6.25	5.83	6.09	5.19
Gm26184	4.78	5.56	4.91	5.52
Klf9	4.42	4.94	4.62	4.79
Gm15384	7.49	7.28	7.78	7.28
D930048G16Rik	4.18	4.09	4.19	3.93
Noxred1	4.36	4.56	4.1	4.81
Mir7224	4.19	4.2	3.59	4.26
Sod3	4.26	4.34	4.21	4.51
Gm14598	4.3	4.41	4.43	4.96
Olfr1129	4.1	4.55	3.99	4.6
Trak1	4.83	4.79	5.11	4.8
Gm21903	4.01	4.33	4.77	4.48
Vapb	4.95	5.07	5.28	4.78
Runx2os1	4.22	4.31	4.38	3.95
Mir337	4.74	5.25	5.07	5.03
PART1_1	3.91	4.14	3.9	4.31
Gm10319	4.3	4.28	4.09	4.42
4930518I15Rik	4.15	3.97	4.26	4.45
Inpp5k	4.98	5.4	5.2	5.27
Gm22359	4.4	5.28	4.45	4.89
Vmn2r46	4.39	4.65	4.32	4.48
Gm13722	5.01	4.7	4.33	4.81
Gm11857	5.04	4.35	4.99	4.69
Gm25157	5.67	9.76	10.12	5.12
Gm22941	5.67	9.76	10.12	5.12
Gm22258	5.67	9.76	10.12	5.12
Vmn1r5	4.12	4.38	3.72	4.1
Kif22	4.23	4.38	4.1	4.46
Ppic	4.83	4.73	5.39	4.86
Prr19	3.91	4.35	4.04	4.7
Gm15197	4.2	4.61	4.15	4.51
Cct2	5.69	5.32	6.41	5.43
Gm11756	4.06	4.48	4.15	4.5
Gm26826	4.39	4.97	4.32	4.73
4933406I18Rik	4.08	4.13	3.93	4.28
Gm19651	4.47	4.25	4.02	4.49

Rpl21-ps5	8.05	8.21	8.69	7.28
Krtap6-3	4.09	3.98	4.14	4.78
Krtap13	3.51	3.83	3.76	4.15
Gm6420	3.65	3.67	3.62	3.95
Gm21370	3.97	3.95	3.68	3.84
Hist2h2aa1	6.56	5.7	6.67	5.89
A230065N10Rik	3.98	4.22	3.73	4.23
Slc22a26	4.27	4.34	3.97	4.6
Hist1h2bm	4.44	4.24	4	3.99
Vmn1r82	4.43	4.01	3.72	4.15
Rpl3-ps1	13.97	13.13	13.98	12.03
Gm13137	4.78	4.81	5.29	4.47
Olfr683	4.32	4.6	4.12	4.73
Gal3st2	4.14	4.54	4.27	4.39
Gm27362	3.95	4.68	4.08	4.71
Hipk3	6.43	6.18	6.91	6.18
Map2k7	5.11	5.41	5.77	5.37
Glce	4.61	4.62	4.93	4.48
Cdc42bpa	5.65	5.69	6.92	5.99
Wbp4	5.55	5.83	6.18	5.88
Dicer1	4.7	4.91	5.19	4.83
Gm24836	3.91	4.37	3.91	4.37
Mir491	4.16	4.4	3.91	4.62
Mknk2	4.75	4.82	5.38	4.9
Olfr1140	4.54	4.59	4.27	4.88
Trim40	4.19	4.23	4.1	4.54
Eea1	4.94	5.17	5.47	4.95
Gm12401	4.61	4.99	4.74	4.37
Erv3	4.04	3.85	3.84	4.33
Hsd11b2	3.63	3.91	3.54	3.99
Hsd17b12	4.86	4.93	5.36	4.64
Tril	4.39	4.55	5.17	4.44
Mir684-1	8.34	8.65	11.26	8.63
Ankib1	4.97	5.11	5.44	4.76
Gm11943	4.06	4.22	3.91	4.82
Gm15694	4.33	5.22	4.81	4.57
Gm17030	4.56	4.62	4.22	4.88
Gm25248	4.92	5.05	5.35	5.12
Tmem5	4.49	4.61	4.76	4.41
Mir669m-2	12.23	12.3	14.79	12.22
Gm14906	4.03	4.29	3.88	4.4
LOC100862007	4.63	5.29	5.46	5.55
Gm16755	4.58	4.78	5.18	5.06
Gm6987	5.74	5.22	6.04	4.98

Catsperb	4.18	4.24	4.1	4.41
Gm25262	9.37	9.43	9.69	9.35
Gm17695	4.3	4.21	3.78	4.1
Gm5145	4.29	4.41	3.92	4.12
D1Ertd448e	4.88	4.4	4.66	4.53
Vapa	5.89	5.59	6.65	5.27
Vmn2r50	4.47	4.8	4.36	4.56
Mir669n	7.48	7.37	9.24	7.71
Cdk19	6.01	6.08	7.27	6.13
Gm11403	3.9	4.21	4.15	4.59
Setd7	4.82	4.77	5.11	4.79
B930078G14Rik	3.55	3.32	3.27	3.58
Synrg	4.88	5.01	5.36	5.04
Sgol2b	4.19	4.27	4.16	4.62
Gm26624	3.96	4.46	4.09	4.41
Gm20505	4.09	3.96	3.77	4.08
Gm20435	4.09	3.96	3.77	4.08
Abca1	5.07	5	5.55	4.91
Rpl23a-ps1	6.09	5.32	6.52	5.81
Fam234b	4.9	4.98	5.21	4.8
Gm9934	3.79	3.77	3.78	4.22
Ndfip1	5.71	5.99	6.18	5.51
Gm5272	4.47	5	4.75	4.8
Gm23214	4.02	4.41	4.61	4.44
Ndrg4	6.77	6.43	6.95	6.23
Gm25036	5.84	5.71	6.06	5.51
AA667203	4.01	4.48	4.22	4.34
LOC100861914	4.53	4.48	4.22	4.57
LOC100862270	4.53	4.48	4.22	4.57
Gm14305	6.92	6.07	7.4	5.17
Trgj2	4.25	4.49	4.05	4.48
Vmn2r87	4.05	4.76	4.31	4.34
4930402F11Rik	4.37	4.4	4.23	4.6
Gm22435	4.25	4.31	3.97	4.69
Gm13454	3.8	3.91	3.7	4.15
Gm21910	4.36	4.16	3.88	4.17
Slc6a1	7.24	5.77	7.32	5.72
Six3os1_6	5.56	5.65	5.91	5.55
Gm13012	4.16	4.02	4.07	4.77
Smim22	4.56	4.59	4.86	4.72
Gm23485	4.75	5.55	5.29	5.19
Ccdc3	4.59	4.18	4.06	4.42
Psmc3	6.16	6.01	6.37	5.8
Gm23184	3.9	4.17	3.67	4.03

1810007D17Rik	4.62	4.64	4.69	5.04
Gm10857	3.78	4.15	3.72	3.96
Olfr986	3.95	4.04	3.75	4.03
5830462I19Rik	4.19	4.14	3.99	3.87
Acat1	5.34	5.59	5.69	4.86
Gm27525	11.15	10.36	11.04	9.21
Smg7	4.89	4.99	5.5	4.95
Nog	4.01	3.99	3.73	4.19
Zfp524	3.9	3.76	4.4	4
Scn11a	3.94	4.07	3.89	4.17
Gm11651	3.75	4.04	3.86	4.28
Miox	3.86	4.06	3.78	4.2
Gm26596	3.9	4.07	3.88	4.21
Gm9059	4.05	4.07	3.98	4.38
Gm26007	4.23	4.21	3.99	4.47
Ict1	4.83	4.54	4.84	4.5
Rpl30-ps1	18	17.52	18.09	14.09
Psmb5	6.51	6.14	6.78	5.46
Gm16214	4.16	3.97	3.85	4.15
Olfr484	5.09	4.77	5.25	4.68
Gm5878	3.63	3.99	3.46	3.89
1700071K01Rik	5.1	5.11	5.25	4.86
Gm14905	4.21	3.99	3.89	4.15
Gm24160	4.77	5.17	5.15	4.69
Gm11858	4.5	4.51	4.13	4.77
Gm23121	4.17	4.36	4.26	4.62
Abcd2	4.23	4.63	4.61	4.4
Gm10086	8.05	6.96	7.73	6.46
Urm1	4.51	4.72	4.78	4.48
Mfap4	4.53	4.36	4.14	4.49
Gm7551	10.53	10.8	12.21	11.22
Gm25294	4.11	4.26	3.9	4.55
Mir6937	5.23	5.47	6.15	5.74
Tmem144	4.18	4.33	4.03	4.61
Gm22498	4.6	5.37	5.31	5.22
Mtch1	6.79	6.77	7.37	6.41
Olfr680-ps1	4.6	4.27	4.24	4.14
Mir24-2	4.8	5.23	4.77	4.66
Tnpo3	4.64	5.11	5.19	4.64
Copb2	5.74	5.56	6.06	5.37
Vmn2r102	4.36	4.44	4	4.26
Gm24010	4.44	4.44	3.69	4.58
2310026I22Rik	4.41	4.34	4.14	4.56
Tob1	5.22	4.94	5.06	5.02

Gm26716	4.26	4.88	4.9	4.71
Gm26978	5.59	5.33	5.41	6.45
Nkx6-3	3.99	4.39	4.07	4.57
Olfr1404	4.07	4.18	4.04	4.39
Gm15567	5.56	5.37	5.23	6.03
Syt11	5.83	5.31	5.85	5.08
Gm7104	4.39	3.83	3.98	4.39
Gm8359	3.9	4.18	4.15	3.9
Gm13624	5.43	5.65	5.4	5.7
Trav14n-1	4.26	4.78	3.86	4.2
Gm19752	7.67	5.85	9.01	5.67
Nr1h5	3.94	4.25	3.94	4.14
Naa50	5.16	4.98	5.55	4.91
Gm15933	4.02	4.2	3.94	4.84
Mob3a	4.32	4.14	4.02	4.04
Katnbl1	4.6	4.54	4.89	4.54
G630016G05Rik	4.58	4.07	4.09	3.95
Tpm3-rs2	5.06	4.97	5.51	5.23
Gm11099	5.18	5.07	4.29	5.56
Gm11011	5.55	6.29	5.07	6.08
Gm24910	4.65	4.44	4.51	5.03
Gm13915	4.41	4.95	4.17	4.16
Mir7085	4.89	5.08	5.31	5.02
Vmn2r10	4.22	4.09	4.02	4.32
Gm13005	4.02	3.97	3.94	4.67
Grm1	4.25	4.5	4.64	4.47
Pea15a	6.75	6.15	7.31	6.03
Prnd	4.88	4.83	4.94	4.51
1700071G01Rik	4.04	3.95	3.85	4.24
Olfr6	4.08	3.76	4.02	3.89
Sult1c2	4.18	4.41	4.19	4.42
Nrd1	6.43	6.39	6.43	5.75
8030443G20Rik	4	4.28	3.94	4.24
Olfr952	4.22	5	3.96	4.81
Rab5a	5.96	6.09	6.4	5.93
1700025K24Rik	4.12	4.25	3.95	4.05
B830017H08Rik	4.31	4.39	4.47	4.7
Gm15628	4.91	4.65	4.69	5.22
Olfr1048	3.73	3.82	3.79	4.25
Higd1a	5.63	5.7	6.14	5.71
5033403H07Rik	4.3	4.47	4.13	4.71
9830166K06Rik	4.05	4.3	4.1	4.63
n-R5s8	4.99	5.3	5.04	5.74
Prr23a1	3.92	4.39	3.56	4.3

Adam1b	3.98	3.91	3.78	4.26
Gpr173	4.29	4.1	4.3	4.57
Rbmy	4.36	4.38	4.19	4.54
Gm16226	4.08	3.95	3.86	4.25
Pyroxd1	4.18	4.59	4.25	4.45
Gpr89	4.65	4.15	4.2	4.35
Gm18159	3.82	3.99	3.79	4.33
Gm22553	3.87	4.6	3.6	4.53
Gm6822	6.1	6.66	6.27	4.9
Rpl31-ps7	7.57	6.83	7.86	6.49
Vmn2r15	3.97	4.43	4.08	4.53
1700061J23Rik	4.29	4.39	4.31	4.63
Spaca7	4.32	4.49	4.01	4.2
Vimp	4.41	4.64	5.65	4.56
Rps27l	5.43	4.76	5.17	4.93
Serpinb6d	4.13	4.12	3.93	4.39
Max	7.2	5.47	6.21	5.65
Gm17249	4	4.08	3.81	4.21
Slxl1	4.17	4.43	4.3	4.95
2310047D07Rik	4.65	4.28	4.09	4.45
Gm24348	3.8	3.97	3.82	4.48
Dkkl1	4.37	4.51	4.14	4.63
Gm15962	4.56	4.98	4.72	4.45
Cask	5.23	5.19	5.54	4.99
Gm20782	4.33	4.24	4.66	5.31
Akap10	4.78	4.53	5.15	4.78
Gm3187	5.18	4.74	4.52	4.71
Igkv1-35	4.42	4.61	4.06	4.6
Gm12359	4.15	4.24	3.95	4.03
Olfr1079	4.61	4.41	4.18	4.58
4930551O13Rik	3.98	4.22	3.85	4.33
Gm22296	4.16	3.84	3.8	4.15
Rad51ap1	4.45	4.38	4.05	4.44
Ankrd13a	5.2	5.14	5.47	4.96
Gm24382	4.19	4.68	4.17	4.44
Olfr199	4.13	4.6	4.08	4.51
Stard13	4.37	4.49	4.76	4.4
Glrp1	5.02	4.76	4.99	5.07
Gm17250	4.26	4.53	3.98	4.44
Gm20481	4.16	4.05	3.83	3.97
Gm27607	5.07	5.31	4.9	5.08
Gm12276	5.68	5.39	6.34	5.6
Hnrnpul1	5.29	4.93	5.34	4.92
Gm24988	4.14	5.21	3.93	4.42

Gm9753	3.79	4.09	3.7	4.29
Gm22973	4.51	5.16	4.62	5.61
Vmn1r-ps100	4.04	4.46	3.8	4.22
Rps19-ps14	5.44	5.22	5.22	5.04
Btnl10	4.01	4.16	3.78	4.13
Ahcyl1	6.38	6.42	6.67	6.09
Mir665	4.8	4.06	4.58	3.82
Has2	4.61	4.95	4.99	4.88
Mysm1	4.52	4.74	5.1	4.74
Chmp1b	4.61	4.62	5.01	4.11
Gm23562	4.13	4.22	4.73	3.78
Csnka2ip	3.74	4.17	3.85	4.21
Gm6083	4.1	3.85	4.06	4.4
Eif3s6-ps1	6.5	6.44	6.88	5.17
Mir6381	5.14	4.8	5.54	4.8
Gm13149	5.59	5.69	6.11	5.27
A230072I06Rik	3.88	4.16	4.09	4.4
Mrps5	4.5	4.67	4.87	4.84
Gm27385	4.35	4.66	3.85	4.63
Atp2c1	4.96	5.03	5.25	4.87
Olfr2	3.79	3.74	3.4	3.88
Mir466l	4.39	4.27	4.74	4.15
Birc6	5.49	5.31	5.65	5.22
Mmp1b	3.93	4.11	3.91	4.31
Ube2uos	4.14	4.37	3.82	4.13
Gm24750	4.39	4.39	4.01	4.52
Ankrd37	4.32	4.46	4.63	4.71
Gm24680	3.95	4.15	4.6	4.05
Fam73a	4.96	4.73	5.08	4.39
Gm24296	3.79	3.91	3.83	4.3
Gm14308	6.9	6.82	7.71	5.63
Gm14016	4.57	4.23	4.33	4.87
Vmn2r-ps76	3.66	3.93	3.94	4.11
Gm27038	4.78	4.63	6.16	4.63
Sdc3	5.05	4.83	5.19	5.01
Gm9800	7.75	7.28	7.7	6.8
1810020O05Rik	4.16	3.9	4.02	4.81
Gm26410	4.32	4.33	3.95	4.59
Mir883a	4.51	4.65	4.05	4.17
Gm17146	6.32	7.03	6.57	6.12
Ascl2	3.85	3.93	3.89	4.15
Stk19-ps1	4.49	4.29	4.8	4.41
Olfr297	3.77	4.3	3.87	3.91
Rpl27	5.24	5.2	5.68	5.41

Frmd4b	5.11	5.12	5.55	4.86
Gm4459	4.26	4.22	4.83	4.16
Gm11852	6.47	5.94	6.85	5.73
Rhd	4.08	4.17	3.97	4.37
4930540M05Rik	4.15	4.73	4.1	4.28
Gm25558	4.33	4.08	3.8	4.66
Gm17786	8.7	7.51	8.99	7.69
Olfr178	4.43	3.52	4.34	4.52
Mir2136	5.61	5.7	5.34	5.03
Olfr1201	4.34	4.46	3.87	4.27
Gm15398	4.02	4.12	3.83	4.08
Mir6902	3.97	3.97	4.01	4.78
Pdxdc1	4.83	4.8	5.38	4.76
Adcy10	4.17	4.2	4	4.31
Fgfrl1	4.09	4.51	4.52	4.3
Gm14560	4.6	4.2	4.35	4.79
Gm13820	3.86	4.15	3.77	3.85
A1cf	4.37	4.46	4.2	4.63
Gyg	4.23	4.16	4.86	4.2
Idh3a	5.27	5.36	5.76	5.4
Gm25003	6.57	6.41	7.85	6.7
Gm24919	3.91	4.17	3.97	3.74
Olfr916	4.17	4.18	3.79	3.91
2310026L22Rik	4.02	4.37	3.79	4.1
Gm12231	5.55	4.96	5.51	4.76
Gm13473	4.54	5.09	4.56	4.97
Olfr1443	3.82	4.71	3.92	4.11
Olfr538	4.3	4.45	4	4.08
Gm26701	5.78	4.98	6.32	4.85
Gm14393	7.05	5.92	7.75	6.14
Gm24258	5.21	5.03	5.5	5.29
A330009N23Rik	4.17	4.28	4.31	4.68
Rgs7	4.73	5.47	5.38	5.06
Sfpq	6.9	6.79	7.49	5.87
Ina	4	5.32	4.67	4.46
Ppp1r12a	7.06	7.34	8.19	6.7
Igkv8-24	3.72	4.4	3.83	3.92
Gm14218	3.91	4.32	3.6	4.17
Olfr992	3.52	4.25	3.76	4.45
Gm22750	4.7	4.73	4.3	5.15
Gm20267	4.58	4.35	4.65	4.54
Gm14580	4.34	4.66	4.2	4.43
Gm26467	4.39	5.37	4.62	4.66
Gm25087	4.39	5.37	4.62	4.66

LOC100861931	4.81	5.01	5.14	5.24
Rgag4	3.92	3.96	3.74	4.07
H2afy3	3.9	3.74	3.74	4.22
9330104G04Rik	4.21	4.13	4.13	4.39
Gm13688	4.8	4.49	5.57	4.71
Mcts1	4.47	4.43	4.4	4.13
Phgr1	3.93	3.97	3.64	4.04
Gm26352	4.31	4.82	4.33	4.34
Gm14682	3.79	4.02	3.96	4.39
Hectd1	5.24	4.89	5.47	5.02
Gm26526	3.98	4.07	3.63	4.33
Asic3	4.01	4.24	4.02	4.26
Mrgpra8	4.07	4.35	3.8	4.04
Nek9	5.09	4.97	5.42	4.89
Ndufv2	5.84	6.04	6.83	5.65
Tgfbrap1	4.43	4.55	4.89	4.51
Mir1932	3.77	3.79	3.68	3.98
Nup88	5.03	4.99	5.33	5.04
Gm4340	4.4	4.1	4.66	4.28
Gm23463	3.93	4.48	4.01	4.23
9130208D14Rik	4.74	4.45	4.33	4.4
4933414I15Rik	4.04	4.09	4.07	4.45
Gm20234	5.17	4.4	5	4.24
Gm21837	4.4	4.19	3.78	4.17
Gm21906	4.4	4.19	3.78	4.17
Ighv1-23	3.83	4.36	3.91	4.12
Gm15200	4.16	4.37	4.12	4.43
Akt3	10.93	10.78	11.92	9.86
Fgf15	3.78	3.8	3.56	3.97
Fam101b	3.86	4.03	3.91	4.67
Gm23700	8.56	8.4	9.65	7.73
Fabp7	5.11	4.58	6.48	5.17
Gm14805	4.1	4.38	3.92	4.31
1500035N22Rik	5.14	4.93	5.23	4.91
Gm16574	5.17	5.47	5.38	5.56
1700042G15Rik	4.04	4.06	4.04	4.4
Gm14416	7.53	6.18	8.06	6.41
Cpped1	3.93	4.44	4.1	4.11
Pcdhb15	4.39	4.19	4.67	4.38
Atp2a2	8.34	8.5	9.36	7.74
LOC100862055	4.26	5.24	4.48	4.3
Gm20094	7.8	7.11	8.72	6.38
Gm13898	3.87	4.4	4.09	4.47
Hdac2	5.64	5.16	5.66	5.05

Gm14751	4	4.01	4.22	4.47
Spop	5.44	5.3	5.88	5.26
Gm26789	3.94	4.21	3.69	4.27
Iapp	4.08	4.72	3.95	4.03
Gm13229	5.19	4.84	5.79	5
Gm3053	4.22	4.22	4.18	4.73
Gm21731	3.91	4.46	3.93	4.07
Olfr832	3.87	3.99	3.52	4.34
Gm11814	5.29	5.58	5.59	5.8
Snord58b	3.88	4.53	4.52	4.19
Gm22016	4.6	4.77	4.25	5.36
Gm13058	4.26	3.86	3.86	4.27
Gm6309	3.89	4.12	3.9	4.04
Gm26257	4.33	4.4	4.03	4.64
Slmap	4.98	4.88	5.88	4.89
1700018F24Rik	3.82	4.03	3.88	4.22
Txnl4b	4.61	4.65	4.72	5.02
Gm11446	4.38	4.36	4.26	4.54
Gm11263	8.46	6.01	7.9	5.71
Gm933	4.23	4.09	4.38	4.08
Hmgcs2	5.71	5.28	5.61	4.99
Mir7015	4.22	4.33	5.18	4.8
4930455B14Rik	4.38	4.35	4.16	4.5
LOC100861932	4.72	4.91	5.47	5.21
Golgb1	5.32	5.27	5.43	4.91
Gm23231	4.76	4.81	4.48	5.32
Gm12376	4.25	4.81	4.11	4.44
LOC100861650	6.43	5.96	6.39	5.97
Gm23272	5.8	6.32	6.54	5.79
Vmn1r-ps152	3.91	4.13	3.99	4.6
Gm8394	5	4.62	4.38	4.22
Gm18522	4.42	4.17	3.89	4.28
Gm11333	4.28	4.4	3.87	4
Orm2	4.27	4.44	4.37	4.62
Gm25541	4.31	4.65	3.96	4.7
Gm8229	4.63	4.48	4.14	4.64
Dap	5.33	5.12	5.44	5.07
Gm13475	5.38	5.02	4.44	4.85
Pdpr	4.32	4.36	4.78	4.38
Gm14600	4.03	4.19	3.91	4.35
Gm21809	4.33	4.2	3.72	4.4
Dnajb8	4.04	3.91	3.82	4.24
Gm12324	3.92	3.97	3.91	4.57
Zfp935	5.16	6.51	5.44	5.18

Hspb9	4.28	4.6	4.5	4.79
Mup-ps8	4.24	4.28	4.03	4.65
LOC100862206	3.77	3.94	3.64	4.07
Cdkn2aip	4.44	4.4	4.65	4.89
0610031O16Rik	4.02	4.16	3.91	4.31
AI593442	4.49	4.79	4.95	4.36
Stau2	5.25	5.23	5.57	5.19
Olfr362	4.06	4.11	3.69	4.09
Gm24337	4.46	4.26	4.48	4.73
LOC100861642	8.69	7.26	10.06	6.72
4933429K18Rik	3.96	3.9	3.94	4.41
Gm20806	4.45	4.53	4.37	4.19
Gm23564	3.86	4.12	4.8	3.66
Gm14891	4	3.78	3.75	4.09
Vmn1r17	4.24	3.94	3.69	4.04
Vmn2r-ps16	3.91	4.1	3.97	4.43
Gm25491	4.44	4.46	4.3	4.65
Gm10478	4.87	4.89	4.97	5.4
Mir743	4.4	4.3	4.82	4.03
4933406G16Rik	4.16	4.15	4.04	4.37
Tmem207	4.18	4.2	3.88	4.15
Olfr9	4.45	4.24	4.23	4.04
Cyp4a29-ps1	3.84	4.31	3.91	4.24
Amotl1	4.66	4.63	5.07	4.63
Gm21693	4.16	4.41	4.22	4.53
Dnajb5	4.47	4.73	4.96	4.44
Gm9104	8.85	8.43	9.11	7.88
LOC100862172	4.64	4.58	4	4.76
Heatr5b	4.61	4.53	4.8	4.41
Slc22a8	4.95	4.88	6.3	4.94
Olfr1105	4.02	4.01	3.68	4.14
Tmem59l	5.04	4.91	5.52	4.84
Olfr1384	3.99	4.21	3.87	4.4
Gm17032	3.97	4.82	4.26	4.7
Gtf2h5	4.86	5.04	5.62	5.05
Gm15028	3.95	4.29	4.02	4.51
Vmn2r-ps8	4.31	4.01	3.75	4.56
B4galt1	3.96	4.27	3.93	4.04
S100a16	6.11	6.16	6.6	5.87
Jmy	4.61	4.87	5.43	4.82
Gm20401	3.79	3.81	4.54	3.72
Gm22293	4.25	4.51	4.06	4.89
Mir466h	5.8	5.95	7.35	6.07
Gm24207	5.35	5.77	4.62	5.19

Dbndd1	3.91	4.07	3.72	3.89
Gm23035	4.24	4.28	3.81	3.85
Gng4	4.46	4.27	4.55	4.63
Gm22013	3.92	3.73	3.88	4.31
Trafd1	4.78	4.88	5.07	4.75
Rgs9	8.19	7.33	7.74	5.39
Gm12058	5.94	4.53	5.82	4.5
Sh3glb1	5.34	5.64	6.1	5.48
Sprr2d	3.95	4.14	3.7	4.01
Gm26787	3.88	4.52	4.27	4.18
Cgref1	4.69	4.55	4.3	4.64
Ifitm3	4.66	4.03	4.83	4.66
Gm11335	4.17	4.22	3.95	4.5
4921514A10Rik	4.45	4.56	4.78	4.77
Gm10490	4.53	5.04	4.54	4.46
Gm11433	4	4.04	4.6	4.22
Gm5456	5.1	4.51	4.64	4.61
Fam208a	4.51	4.57	4.91	4.54
Chchd3	5.98	5.18	6.15	4.95
Gm14308	6.88	6.54	7.82	5.94
Scrn2	4.25	4.51	4.15	4.34
Gm17079	4.47	4.41	4.2	4.81
Igfbp3	5.05	4.93	5.63	4.83
9430020K01Rik	8.87	7.94	9.74	7.08
Gm9493	11.18	9.73	13.1	9.75
LOC100862376	4.43	4.21	3.76	4.21
C030005K06Rik	4.09	4.11	4.36	4.26
Platr7	4.41	4.21	3.97	4.42
LOC100862049	5.08	5	5.74	5.28
Gm5388	4.21	4.59	4.23	4.59
Prl7b1	4.11	4.7	3.94	4.18
Gm25162	4.21	4.89	4.55	4.84
Gm13632	5.57	5.03	6.32	4.6
Gm24977	4.81	4.51	4.43	5.01
Iws1	5.08	5.17	5.5	5.09
Ift20	5.63	5.75	5.9	5.37
Olfr156	4.61	4.95	4.77	5.07
Traj54	6.34	7.51	7.19	7.53
Gm12450	4.85	4.78	4.52	4.4
Olfr26	4.4	4.18	3.91	4.31
Gm14451	3.97	3.9	3.82	4.49
Gm24927	4.53	5.6	6.44	4.35
Gm10364	5.66	5.73	5.81	5.32
Snord65	5.41	5.49	5.67	5.46

Gm15393	5.46	4.39	5.98	4.65
Gm12467	4.37	4.55	3.79	3.69
Gpr149	3.96	4.48	3.96	4.53
Ptcd2	4.64	4.47	5.18	4.32
Gm25681	4.02	4.61	3.92	4.02
Odc1	5.01	4.95	5.55	4.56
Gm20630	4.17	4.77	4.27	4.5
Gm3712	4.57	4.85	4.03	4.39
Olfr1061	3.84	4.22	3.6	4.59
Gm4133	3.78	4.03	3.97	4.46
Gm4133	3.78	4.03	3.97	4.46
Cops3	5.18	5.12	5.65	5.1
Gm15682	9.48	9.15	9.43	7.63
Tubal3	4.14	4.74	4.11	4.74
Gm10377	4.63	4.75	4.42	4.44
Pitpna	5.53	5.65	5.84	5.7
Gm25167	4.29	5.09	4.9	4.67
Gm22448	4.45	4.77	4.07	4.14
Mxra8os	4.47	4.87	4.15	4.64
Eml1	4.43	4.58	4.69	4.45
Gm13043	3.91	4.15	3.87	4.31
Gm16442	4.08	4.1	3.81	4.47
1700001C02Rik	4.16	4.5	4.21	4.49
Gm17169	4.01	4.24	3.74	3.95
Skp1a	5.57	5.61	6.35	5.12
Hk1os	4.1	4.21	4.04	4.43
Gm10007	4.29	4.35	3.96	4.2
Gm4911	4.32	4.71	4.34	4.21
Olfr566	4	4.23	3.99	4.07
S100g	3.49	4.15	3.54	3.91
Ptp4a2	7.45	7.16	8.09	7.06
Champ1	4.22	4.57	4.41	4.31

Table S6. Differentially expressed genes for the low glycemic diet (LGD) with soluble epoxide hydrolase inhibitor (sEHI) compared to without sEHI.

Gene Symbol	Description	Fold Change	P-value
Gm14429	predicted gene 14429 [Source:MGI Symbol;Acc:MGI:3649510]	-6.79	3.37E-06
Erdr1	erythroid differentiation regulator 1	-6.13	4.28E-06
H2afv	H2A histone family, member V	-2.25	8.00E-06
Gprin3	GPRIN family member 3	-2.49	8.11E-06
		-2.24	9.69E-06
Nedd8	neural precursor cell expressed, developmentally down-regulated gene 8	-2.35	1.48E-05
Gm19852	PREDICTED: predicted gene, 19852 (Gm19852), miscRNA.	-4.49	1.77E-05
Gm23553	predicted gene, 23553	-2.18	2.03E-05
Gm5346	predicted gene 5346	2.01	2.60E-05
Gm21887	predicted gene, 21887 [Source:MGI Symbol;Acc:MGI:5434051]	-2.33	3.03E-05
		-2.48	3.68E-05
Gm14894	predicted gene 14894 [Source:MGI Symbol;Acc:MGI:3705757]	2.15	3.76E-05
		-2.71	4.37E-05
Gtf3c6	general transcription factor IIIC, polypeptide 6, alpha	-4.68	5.20E-05
Gm23989	predicted gene, 23989 [Source:MGI Symbol;Acc:MGI:5453766]	-8.08	5.26E-05
Gm24132	predicted gene, 24132 [Source:MGI Symbol;Acc:MGI:5453909]	-3.07	5.47E-05
Larp4b	La ribonucleoprotein domain family, member 4B	-2.36	6.57E-05
		2.15	7.36E-05
		-3.71	7.85E-05
Gm19491	PREDICTED: predicted gene, 19491 (Gm19491), miscRNA.	-7.32	9.06E-05
Gm25529	predicted gene, 25529 [Source:MGI Symbol;Acc:MGI:5455306]	-4.62	9.15E-05
Fnta	farnesyltransferase, CAAX box, alpha	-2.9	9.96E-05
Hnrnpa0	heterogeneous nuclear ribonucleoprotein A0	-3.1	0.0001
Wdr48	WD repeat domain 48	-2.15	0.0001
		2.23	0.0001
Gm22702	predicted gene, 22702 [Source:MGI Symbol;Acc:MGI:5452479]	-2.46	0.0001
Gm19789	PREDICTED: predicted gene, 19789 (Gm19789), miscRNA.	-2.41	0.0001
		-6.2	0.0001
		-2.21	0.0001
Mbip	MAP3K12 binding inhibitory protein 1	-2.53	0.0002

Gm22117	predicted gene, 22117 [Source:MGI Symbol;Acc:MGI:5451894]	-10.67	0.0002
		-3.27	0.0002
Gm25325	predicted gene, 25325 [Source:MGI Symbol;Acc:MGI:5455102]	-2.56	0.0002
Matr3	matrin 3	-2.49	0.0002
Mir6338	microRNA 6338	2.02	0.0002
LOC100862313	PREDICTED: uncharacterized LOC100862313, transcript variant 1 (LOC100862313), miscRNA.; PREDICTED: uncharacterized LOC100862313, transcript variant 2 (LOC100862313), miscRNA.	-6.12	0.0002
Atp13a5	ATPase type 13A5	-2.23	0.0002
Papola	poly (A) polymerase alpha; Synthetic construct Mus musculus clone IMAGE:100062187, MGC:190402 poly (A) polymerase alpha (Papola) mRNA, encodes complete protein.	-3.68	0.0002
Mir669d	microRNA 669d [Source:MGI Symbol;Acc:MGI:3783382]	-4.16	0.0002
Gm13803	predicted gene 13803	-3.4	0.0002
Fam168b	family with sequence similarity 168, member B	-2.02	0.0002
Vps41	vacuolar protein sorting 41 (yeast)	-2.08	0.0002
Mir466d	microRNA 466d	-3.51	0.0003
		-15.98	0.0003
Mir669l	microRNA 669l [Source:MGI Symbol;Acc:MGI:3837025]	-2.34	0.0003
Rxrg	retinoid X receptor gamma	-2.32	0.0003
Gm8991	predicted pseudogene 8991 [Source:MGI Symbol;Acc:MGI:3644227]	-6.05	0.0003
Gm15167	predicted gene 15167 [Source:MGI Symbol;Acc:MGI:3705805]	-2.06	0.0003
		-5.95	0.0003
Drd2	dopamine receptor D2	-3.41	0.0003
Gm15446	predicted gene 15446	-2.42	0.0003
Gm22030	predicted gene, 22030 [Source:MGI Symbol;Acc:MGI:5451807]	-29.09	0.0003
2310036O22Rik	RIKEN cDNA 2310036O22 gene	-2.54	0.0003
		-3.11	0.0003
Ndn	necdin	-14.79	0.0003
Gm23181	predicted gene, 23181 [Source:MGI Symbol;Acc:MGI:5452958]	-2.32	0.0004
Kidins220	kinase D-interacting substrate 220	-3.41	0.0004
Thoc1	THO complex 1	-2.19	0.0004
		-8.52	0.0004
Mir7223	microRNA 7223	2.5	0.0004

		2.99	0.0004
692-3; Ftl1; Ftl2	microRNA 692-3; ferritin light polypeptide 1; ferritin light polypeptide 2, pseudogene; ferritin light chain 1 (Ftl1), mRNA.	-2.47	0.0004
2410015M20Ril	RIKEN cDNA 2410015M20 gene	-2.51	0.0004
		-4.17	0.0005
Gm23134	predicted gene, 23134 [Source:MGI Symbol;Acc:MGI:5452911]	-19.38	0.0005
		-2.25	0.0005
		-6.39	0.0005
		-2.85	0.0005
		2.25	0.0005
Supt4a	suppressor of Ty 4A	-3.86	0.0005
Mir667	microRNA 667	-2.8	0.0005
		-2.41	0.0005
		-2.41	0.0005
		-2.41	0.0005
		-2.41	0.0005
		2.08	0.0006
Gm26050	predicted gene, 26050 [Source:MGI Symbol;Acc:MGI:5455827]	-6.22	0.0006
Aff4	AF4/FMR2 family, member 4	-2.05	0.0006
		-16.06	0.0006
Smim11	small integral membrane protein 11	-2.34	0.0006
Nxf1; Tmem223	nuclear RNA export factor 1; transmembrane protein 223	-2.21	0.0006
Gm5601	predicted pseudogene 5601	-3.71	0.0006
		-3.09	0.0006
		-2.93	0.0006
Vmn1r11	vomeronasal 1 receptor 11	2.53	0.0006
Larp7	La ribonucleoprotein domain family, member 7; Synthetic construct Mus musculus clone IMAGE:100016333, MGC:184518 La ribonucleoprotein domain family, member 7 (Larp7) mRNA, encodes complete protein.	-2.15	0.0006
Armc1	armadillo repeat containing 1	-2.12	0.0006
Gm14420	predicted gene 14420	-2.74	0.0006
Prpf18	PRP18 pre-mRNA processing factor 18 homolog (yeast)	-2.53	0.0007
		-3.6	0.0007
		-5.58	0.0007
		-3.16	0.0007
		-3.06	0.0007

; Mir297a-3; M	microRNA 297b; microRNA 297a-3; microRNA 297a-4	-4.17	0.0007
Serpine2	serine (or cysteine) peptidase inhibitor, clade E, member 2	-2.25	0.0007
		-2.49	0.0008
		2.21	0.0008
Gm14392	predicted gene 14392 [Source:MGI Symbol;Acc:MGI:3649574]; predicted gene 14392	-2.61	0.0008
Otud6b	OTU domain containing 6B	-3.47	0.0008
LOC100861894	PREDICTED: uncharacterized LOC100861894 (LOC100861894), miscRNA.	-2.04	0.0008
Gm2962	predicted pseudogene 2962 [Source:MGI Symbol;Acc:MGI:3781140]	-15.87	0.0008
		-4.81	0.0008
Gm25246	predicted gene, 25246 [Source:MGI Symbol;Acc:MGI:5455023]	-2.45	0.0009
Tra2b	transformer 2 beta homolog (Drosophila)	-2.42	0.0009
Ubxn4	UBX domain protein 4	-2.02	0.0009
		2.09	0.0009
Alg11	asparagine-linked glycosylation 11 (alpha-1,2-mannosyltransferase)	-2.28	0.0009
Gm5428	predicted gene 5428 [Source:MGI Symbol;Acc:MGI:3647789]; predicted gene 5428	-4.08	0.0009
		-3.08	0.0009
		-3.46	0.001
		-3.46	0.001
		-99.43	0.001
Gm16261	predicted gene 16261 [Source:MGI Symbol;Acc:MGI:3826544]	-2.78	0.001
Vti1b	vesicle transport through interaction with t-SNAREs 1B	-2.86	0.001
Cp	ceruloplasmin	-2.79	0.001
		-4.35	0.001
		-2.5	0.001
		-2.89	0.001
Gm4540	predicted gene 4540 [Source:MGI Symbol;Acc:MGI:3782724]	-4.94	0.0011
Gm22734	predicted gene, 22734	-2.09	0.0011
Gm17268	predicted gene, 17268 [Source:MGI Symbol;Acc:MGI:4936902]	2.75	0.0011
Gm8358	predicted gene 8358 [Source:MGI Symbol;Acc:MGI:3648202]	-3.75	0.0011
Gm24556	predicted gene, 24556 [Source:MGI Symbol;Acc:MGI:5454333]	-2.56	0.0011

Gm27975	predicted gene, 27975 [Source:MGI Symbol;Acc:MGI:5531357]	2.2	0.0011
		-4.99	0.0012
Wnk1	WNK lysine deficient protein kinase 1	-2.06	0.0012
Gm20372	PREDICTED: predicted gene, 20372 (Gm20372), miscRNA.	-4.8	0.0012
LOC10086193	PREDICTED: uncharacterized LOC100861936 (LOC100861936), miscRNA.	-3.4	0.0012
Gm25559	predicted gene, 25559 [Source:MGI Symbol;Acc:MGI:5455336]	-14.85	0.0012
Cldn34d	claudin 34D	2	0.0012
Gm25567	predicted gene, 25567	-3.96	0.0012
Uqcrb	ubiquinol-cytochrome c reductase binding protein	-4.2	0.0012
Gm24056	predicted gene, 24056 [Source:MGI Symbol;Acc:MGI:5453833]	-4.77	0.0012
Tpt1-ps5	tumor protein, translationally-controlled, pseudogene 5 [Source:MGI Symbol;Acc:MGI:2664999]	-4.56	0.0012
Gm8858	predicted gene 8858 [Source:MGI Symbol;Acc:MGI:3648007]	-2.76	0.0012
Ppp1cc	protein phosphatase 1, catalytic subunit, gamma isoform	-3.12	0.0012
		-3.25	0.0012
Gm25249	predicted gene, 25249 [Source:MGI Symbol;Acc:MGI:5455026]	-2.92	0.0012
Chmp5	charged multivesicular body protein 5	-2.28	0.0012
Mid1	midline 1	-3.35	0.0012
Tpt1-ps6	tumor protein, translationally-controlled, pseudogene 6	-2.03	0.0012
		-8.54	0.0012
Zfp442	zinc finger protein 442 (Zfp442), mRNA.; zinc finger protein 442	-2.09	0.0012
Tspyl2	TSPY-like 2	-3.08	0.0012
naset2a; Rnaset	ribonuclease T2A; ribonuclease T2B (Rnaset2b), mRNA.; ribonuclease T2B, mRNA (cDNA clone MGC:27782 IMAGE:3156549), complete cds.; ribonuclease T2B, mRNA (cDNA clone MGC:107248 IMAGE:6703307), complete cds.; ribonuclease T2B, mRNA (cDNA clone MGC:117616 IMAGE:30527517), complete cds.; ribonuclease T2B	-3.01	0.0012
Arl2bp	ADP-ribosylation factor-like 2 binding protein	-2.04	0.0013
Amd2; Amd1	S-adenosylmethionine decarboxylase 2; S-adenosylmethionine decarboxylase 1	-3.43	0.0013
Zfc3h1	zinc finger, C3H1-type containing	-2.1	0.0013

Gm5457	predicted pseudogene 5457 [Source:MGI Symbol;Acc:MGI:3646665]	-16.65	0.0013
		-3.73	0.0013
Igkv4-90	immunoglobulin kappa chain variable 4-90	2.49	0.0013
		-2.1	0.0013
Cdc42	cell division cycle 42	-2.58	0.0013
Ddah1	dimethylarginine dimethylaminohydrolase 1	-2.17	0.0013
Gm12481	predicted gene 12481	-2.63	0.0013
Gabarapl2	gamma-aminobutyric acid (GABA) A receptor-associated protein-like 2	-2.31	0.0014
Got2	glutamatic-oxaloacetic transaminase 2, mitochondrial; glutamate oxaloacetate transaminase 2, mitochondrial	-2.36	0.0014
		2.07	0.0014
		-2.42	0.0014
Pten	phosphatase and tensin homolog	-3.58	0.0014
Arl5a	ADP-ribosylation factor-like 5A	-2.95	0.0014
Slco1c1	solute carrier organic anion transporter family, member 1c1	-3.66	0.0014
Gm10813	predicted gene 10813 [Source:MGI Symbol;Acc:MGI:3708613]	2.55	0.0014
)417; RP24-458	predicted gene 20417 [Source:MGI Symbol;Acc:MGI:5141882]; novel transcript	-7.46	0.0015
Rps6-ps1	ribosomal protein S6, pseudogene 1 [Source:MGI Symbol;Acc:MGI:3809093]	-6.31	0.0015
		2.04	0.0015
Gm16238	predicted gene 16238 [Source:MGI Symbol;Acc:MGI:3802104]	-2.42	0.0015
		-31.81	0.0015
Gm27248	predicted gene 27248 [Source:MGI Symbol;Acc:MGI:5521091]	-3.06	0.0016
		-2.46	0.0016
Zeb1	zinc finger E-box binding homeobox 1	-2.6	0.0016
Olfr692	olfactory receptor 692	2.06	0.0016
Gm22015	predicted gene, 22015 [Source:MGI Symbol;Acc:MGI:5451792]	-3.46	0.0016
		-4.12	0.0016
H2afz	H2A histone family, member Z	-3.92	0.0016
Gm6023	predicted gene 6023 [Source:MGI Symbol;Acc:MGI:3645815]	-3.13	0.0017
Ptprg	protein tyrosine phosphatase, receptor type, G; Synthetic construct Mus musculus clone IMAGE:100068354, MGC:195898 protein tyrosine phosphatase, receptor type, G (Ptprg) mRNA, encodes complete protein.	-2.75	0.0017

Cd47	CD47 antigen (Rh-related antigen, integrin-associated signal transducer)	-2.39	0.0017
4434; 0610010E	predicted gene 14308 (Gm14308), mRNA.; predicted gene 14430 (Gm14430), mRNA.; predicted gene 14434 (Gm14434), mRNA.; RIKEN cDNA 0610010B08 gene (0610010B08Rik), mRNA.; predicted gene 4724 (Gm4724), mRNA.; predicted gene 11007; RIKEN cDNA 0610010B08 gene	-3.41	0.0017
		-2.57	0.0017
Gm14418	predicted gene 14418 [Source:MGI Symbol;Acc:MGI:3702408]	-2.99	0.0018
Mir494	microRNA 494	-3.06	0.0018
		-2.39	0.0019
		-2.5	0.0019
		-4.01	0.0019
Rps15a-ps5	ribosomal protein S15A, pseudogene 5	-2.96	0.0019
Gm14334	predicted gene 14334 [Source:MGI Symbol;Acc:MGI:3649938]	2.12	0.0019
Sumo1	small ubiquitin-like modifier 1; SMT3 suppressor of mif two 3 homolog 1 (yeast)	-4.45	0.002
Gm15030	predicted gene 15030 [Source:MGI Symbol;Acc:MGI:3705338]	3.47	0.002
Gm8203	predicted pseudogene 8203	-4.6	0.002
		-4.18	0.002
Gm14405	predicted gene 14405 (Gm14405), non-coding RNA.; predicted gene 14405	-2.58	0.002
Chmp7	charged multivesicular body protein 7	-2.33	0.002
Gm10282	predicted pseudogene 10282 [Source:MGI Symbol;Acc:MGI:3704312]	-2.83	0.0021
Agfg1	ArfGAP with FG repeats 1	-2.13	0.0021
Gm19595	PREDICTED: predicted gene, 19595 (Gm19595), miscRNA.	-13.72	0.0021
Eif5; Snora28	eukaryotic translation initiation factor 5; small nucleolar RNA, H/ACA box 28	-2.02	0.0021
Gm22713	predicted gene, 22713 [Source:MGI Symbol;Acc:MGI:5452490]	2.1	0.0021
		-6.14	0.0021
Gm20253	PREDICTED: predicted gene, 20253 (Gm20253), miscRNA.	-2.73	0.0021
Mir669b	microRNA 669b	-4.79	0.0021
Paip2	polyadenylate-binding protein-interacting protein 2	-4.38	0.0021
		2.15	0.0021
		-4.64	0.0022

		2.16	0.0022
		-3.17	0.0022
Gm23315	predicted gene, 23315 [Source:MGI Symbol;Acc:MGI:5453092]	2.15	0.0022
Gm13163	predicted gene 13163 [Source:MGI Symbol;Acc:MGI:3651199]	-2.06	0.0022
Tra2a	transformer 2 alpha homolog (Drosophila)	-2.91	0.0022
A130040M12Ri	RIKEN cDNA A130040M12 gene (A130040M12Rik), non-coding RNA.	-4.57	0.0022
m14391; Gm67	predicted gene 14391; predicted gene 6710	-2.04	0.0022
Gm23853	predicted gene, 23853 [Source:MGI Symbol;Acc:MGI:5453630]	2.17	0.0022
		-7.94	0.0022
Ppp1r1b	protein phosphatase 1, regulatory (inhibitor) subunit 1B	-2.03	0.0023
		2.05	0.0023
		-5.1	0.0023
Gm12251	predicted gene 12251	-3.09	0.0023
		-5.46	0.0023
		-2.86	0.0023
Mir692-2	microRNA 692-2	-2.18	0.0023
Gm3272	predicted pseudogene 3272	-3.63	0.0023
		-5.22	0.0023
Abhd2	abhydrolase domain containing 2	-2.21	0.0024
		-2.03	0.0024
Slc35b1	solute carrier family 35, member B1	-2.26	0.0024
LOC100861675	PREDICTED: uncharacterized LOC100861675 (LOC100861675), miscRNA.	-2.95	0.0024
		-2.96	0.0024
Olfr875	olfactory receptor 875	2.05	0.0024
Gm23190	predicted gene, 23190 [Source:MGI Symbol;Acc:MGI:5452967]	-25.6	0.0024
Gm5136	predicted gene 5136; predicted gene 5136 (Gm5136), mRNA.	2.02	0.0024
		-3.14	0.0024
Gm2026	predicted gene 2026 [Source:MGI Symbol;Acc:MGI:3780195]; predicted gene 2026	-4.21	0.0024
Tardbp	TAR DNA binding protein	-4.23	0.0024
		-4.18	0.0024
Nostrin	nitric oxide synthase trafficker	-2.09	0.0025
		-2.01	0.0025
Gm24482	predicted gene, 24482 [Source:MGI Symbol;Acc:MGI:5454259]	-2.56	0.0025
Zfp638	zinc finger protein 638	-3.07	0.0025

Gm13249	predicted gene 13249	-2.43	0.0025
		-4.91	0.0025
		2.42	0.0025
Kif5b	kinesin family member 5B	-2.35	0.0025
Selt	selenoprotein T	-2.67	0.0025
LOC100862094	PREDICTED: uncharacterized LOC100862094 (LOC100862094), miscRNA.	-5.25	0.0025
Gm22403	predicted gene, 22403 [Source:MGI Symbol;Acc:MGI:5452180]	-2.48	0.0025
		2.3	0.0026
Gm22232	predicted gene, 22232 [Source:MGI Symbol;Acc:MGI:5452009]	-4.45	0.0026
Hacd2	3-hydroxyacyl-CoA dehydratase 2	-2.1	0.0026
Gm6265	predicted pseudogene 6265	-3.09	0.0026
m14391; Gm67	predicted gene 14391 (Gm14391), transcript variant 1, mRNA.; predicted gene 6710 (Gm6710), mRNA.; predicted gene 14391 (Gm14391), transcript variant 2, mRNA.; predicted gene 6710; predicted gene 14391	-2.8	0.0026
Zfp932	zinc finger protein 932	-2.79	0.0026
		-3.33	0.0026
		2.06	0.0026
		-7.23	0.0027
Gm12529	predicted gene 12529 [Source:MGI Symbol;Acc:MGI:3651544]	-2.54	0.0027
		-2.93	0.0027
Gm14421	predicted gene 14421 [Source:MGI Symbol;Acc:MGI:3652254]	-3.43	0.0027
Gm24054	predicted gene, 24054 [Source:MGI Symbol;Acc:MGI:5453831]	-2.04	0.0028
Gm14269	predicted gene 14269 [Source:MGI Symbol;Acc:MGI:3650060]	-3.26	0.0028
Tgoln1; Tgoln2	trans-golgi network protein; trans-golgi network protein 2; Synthetic construct Mus musculus clone IMAGE:100063853, MGC:193320 trans-golgi network protein 2 (Tgoln2) mRNA, encodes complete protein.	-2.17	0.0028
Mir669f	microRNA 669f [Source:MGI Symbol;Acc:MGI:3783384]	-2.02	0.0028
		3.86	0.0028
Mff	mitochondrial fission factor	-2.81	0.0028
Gm16060	predicted pseudogene 16060 [Source:MGI Symbol;Acc:MGI:3783246]	-3.89	0.0028
		2.15	0.0028

		-11.33	0.0028
4930480E11Rik	RIKEN cDNA 4930480E11 gene	2.21	0.0028
1810022K09Rik	RIKEN cDNA 1810022K09 gene	-3.47	0.0029
Impact	impact, RWD domain protein; imprinted and ancient	-4.41	0.0029
		-2.69	0.0029
Pten	phosphatase and tensin homolog	-3.43	0.003
Btbd1	BTB (POZ) domain containing 1	-2.39	0.003
Mir6239	microRNA 6239	-2.02	0.003
Gm14180	predicted gene 14180 [Source:MGI Symbol;Acc:MGI:3650017]	-2.19	0.003
Gm24646	predicted gene, 24646 [Source:MGI Symbol;Acc:MGI:5454423]	-4.11	0.003
Gm12183	predicted gene 12183	-2.3	0.003
Gm26233	predicted gene, 26233 [Source:MGI Symbol;Acc:MGI:5456010]	-5.68	0.003
Gm14411	predicted gene 14411	-2.66	0.0031
Pde2a	phosphodiesterase 2A, cGMP-stimulated	-2.08	0.0031
Ivns1abp	influenza virus NS1A binding protein	-2.56	0.0032
		-2.4	0.0032
		2.09	0.0032
Gm25911	predicted gene, 25911 [Source:MGI Symbol;Acc:MGI:5455688]	-86.15	0.0032
		-6.55	0.0032
2480; RP23-39	predicted gene 12480 [Source:MGI Symbol;Acc:MGI:3651952]; novel transcript	-2.37	0.0032
Mir684-2	microRNA 684-2; microRNA 684-2 (Mir684-2), microRNA.	-451.28	0.0032
		2.59	0.0033
Mir466j	microRNA 466j [Source:MGI Symbol;Acc:MGI:3783376]	-8.07	0.0033
Gm15921	predicted gene 15921 [Source:MGI Symbol;Acc:MGI:3801933]	-5.23	0.0033
		-2.8	0.0034
		-27.88	0.0034
		-2.18	0.0034
Gm2310	predicted gene 2310 [Source:MGI Symbol;Acc:MGI:3780481]	-5.21	0.0034
Insl5	insulin-like 5	-2.14	0.0034
LOC100862107	PREDICTED: uncharacterized LOC100862107 (LOC100862107), miscRNA.	-2.1	0.0034
Gm3355	predicted gene 3355 [Source:MGI Symbol;Acc:MGI:3781533]	-3.24	0.0035
Gm5564	predicted gene 5564 [Source:MGI Symbol;Acc:MGI:3645018]	-2.08	0.0035

Gm24557	predicted gene, 24557 [Source:MGI Symbol;Acc:MGI:5454334]	-10.55	0.0035
Gm25233	predicted gene, 25233	-4.35	0.0035
LOC10086221(	PREDICTED: uncharacterized LOC100862216, transcript variant 1 (LOC100862216), miscRNA.; PREDICTED: uncharacterized LOC100862216, transcript variant 2 (LOC100862216), miscRNA.	-4.9	0.0035
Vmn1r74	vomeronasal 1 receptor 74	2.16	0.0035
Gm10232	predicted pseudogene 10232	-3.61	0.0035
Gm14434; 061(	predicted gene 14308 (Gm14308), mRNA.; predicted gene 14430 (Gm14430), mRNA.; predicted gene 14434; RIKEN cDNA 0610010B08 gene; predicted gene 4724	-2.25	0.0036
Gm23334	predicted gene, 23334 [Source:MGI Symbol;Acc:MGI:5453111]	-9.21	0.0036
Cdk13	cyclin-dependent kinase 13	-3.92	0.0036
		-2.25	0.0036
Aplp2	amyloid beta (A4) precursor-like protein 2	-2.49	0.0036
Gm22694	predicted gene, 22694 [Source:MGI Symbol;Acc:MGI:5452471]	-2.61	0.0036
		-2.5	0.0036
Gm25506	predicted gene, 25506 [Source:MGI Symbol;Acc:MGI:5455283]	-2.54	0.0036
Gm12960	predicted gene 12960	-2.52	0.0036
Gm12943	predicted gene 12943 [Source:MGI Symbol;Acc:MGI:3650497]	-3.78	0.0037
		2.03	0.0037
Gm23487	predicted gene, 23487	-2.93	0.0037
		-5.06	0.0037
Gm25128	predicted gene, 25128 [Source:MGI Symbol;Acc:MGI:5454905]	-17.17	0.0037
Gm13798	predicted gene 13798 [Source:MGI Symbol;Acc:MGI:3652036]	-2.45	0.0037
Gm7381	predicted gene 7381 [Source:MGI Symbol;Acc:MGI:3643743]	-5.07	0.0037
Hist1h2bc	histone cluster 1, H2bc	-2.95	0.0038
Gm23644	predicted gene, 23644 [Source:MGI Symbol;Acc:MGI:5453421]	2.08	0.0038
Lmo3	LIM domain only 3	-2.1	0.0038
		-3.4	0.0039
Akap8	A kinase (PRKA) anchor protein 8	-2.8	0.0039

Rock1	Rho-associated coiled-coil containing protein kinase 1; Synthetic construct Mus musculus clone IMAGE:100062871, MGC:190980 Rho-associated coiled-coil containing protein kinase 1 (Rock1) mRNA, encodes complete protein.	-2.22	0.0039
Gm8840	predicted gene 8840 [Source:MGI Symbol;Acc:MGI:3647579]	-2.14	0.0039
Clptm1	cleft lip and palate associated transmembrane protein 1	-2.4	0.004
Gm10254	predicted gene 10254 [Source:MGI Symbol;Acc:MGI:3708673]	-9.1	0.004
		-2.85	0.0041
Gm2174	predicted gene 2174 [Source:MGI Symbol;Acc:MGI:3780344]	-3.5	0.0041
Gm24245	predicted gene, 24245 [Source:MGI Symbol;Acc:MGI:5454022]	-185.44	0.0041
Gm24270	predicted gene, 24270 [Source:MGI Symbol;Acc:MGI:5454047]	-185.44	0.0041
LOC100861752	PREDICTED: uncharacterized LOC100861752 (LOC100861752), miscRNA.	-2.9	0.0042
C1qb	complement component 1, q subcomponent, beta polypeptide	-2.04	0.0042
Gm24187	predicted gene, 24187 [Source:MGI Symbol;Acc:MGI:5453964]	-744.88	0.0042
		-2.25	0.0042
Gpr158	G protein-coupled receptor 158; Synthetic construct Mus musculus clone IMAGE:100062508, MGC:190898 G protein-coupled receptor 158 (Gpr158) mRNA, encodes complete protein.	-2.69	0.0042
		-2.16	0.0042
27243; RP23-35	predicted gene 27243 [Source:MGI Symbol;Acc:MGI:5521086]; novel transcript	-2.96	0.0042
		-2.21	0.0043
; 0610010B08R	predicted gene 14308 (Gm14308), mRNA.; predicted gene 14430 (Gm14430), mRNA.; predicted gene 14434 (Gm14434), mRNA.; RIKEN cDNA 0610010B08 gene (0610010B08Rik), mRNA.; predicted gene 4724 (Gm4724), mRNA.; predicted gene 11007; predicted gene 2007; predicted gene 14308	-2.01	0.0043
20899; Gapdh-p	predicted gene, 20899; glyceraldehyde-3-phosphate dehydrogenase, pseudogene 15 [Source:MGI Symbol;Acc:MGI:5434255]	-2.6	0.0043

Gm25071	predicted gene, 25071 [Source:MGI Symbol;Acc:MGI:5454848]	-12.62	0.0044
		-2.07	0.0044
		-2.07	0.0044
Gm11703	predicted gene 11703	-6.4	0.0044
Gm23278	predicted gene, 23278 [Source:MGI Symbol;Acc:MGI:5453055]	-3.33	0.0044
Gm20349	PREDICTED: predicted gene, 20349 (Gm20349), miscRNA.	-5.02	0.0044
Rps15a-ps7	ribosomal protein S15A, pseudogene 7	-4.32	0.0044
D8Ertd738e	DNA segment, Chr 8, ERATO Doi 738, expressed	-2.83	0.0045
		-3.48	0.0046
Mir466o	microRNA 466o	-33.08	0.0046
Rps6-ps3	ribosomal protein S6, pseudogene 3 [Source:MGI Symbol;Acc:MGI:3643565]	-5.76	0.0046
Gm23881	predicted gene, 23881 [Source:MGI Symbol;Acc:MGI:5453658]	-9.53	0.0046
Cltc	clathrin, heavy polypeptide (Hc)	-3.48	0.0046
Rpl35a-ps2	ribosomal protein L35A, pseudogene 2	-6.82	0.0046
		-2.25	0.0047
Gm3839	predicted pseudogene 3839	-2.4	0.0047
Vmn1r84	vomeronasal 1 receptor 84	2.3	0.0048
Atp6ap2	ATPase, H+ transporting, lysosomal accessory protein 2	-2.37	0.0048
		2.28	0.0048
AY036118	cDNA sequence AY036118	-3.32	0.0048
Utp14b; Acsl3	UTP14, U3 small nucleolar ribonucleoprotein, homolog B (yeast); acyl-CoA synthetase long-chain family member 3	-2.46	0.0048
Gm10146	predicted gene 10146 [Source:MGI Symbol;Acc:MGI:3704367]	-2.18	0.0048
Gm24834	predicted gene, 24834	-5.33	0.0048
S1pr1	sphingosine-1-phosphate receptor 1	-2.08	0.0049
		-16.92	0.0049
Slc25a5	solute carrier family 25 (mitochondrial carrier, adenine nucleotide translocator), member 5	-2.47	0.0049
		2.02	0.0049
		-4.86	0.005
		-2.21	0.005
Gm8662	predicted gene 8662 [Source:MGI Symbol;Acc:MGI:3645694]	-5.61	0.005
Mir466k	microRNA 466k [Source:MGI Symbol;Acc:MGI:3783377]	-8.33	0.005

LOC100505062	PREDICTED: uncharacterized LOC100505062 (LOC100505062), miscRNA.	-3.29	0.005
LOC100861900	PREDICTED: uncharacterized LOC100861900 (LOC100861900), miscRNA.	-2.1	0.005
Gng7	guanine nucleotide binding protein (G protein), gamma 7	-4.8	0.005
Gm5528	predicted gene 5528	-2.8	0.0051
Gm19425	PREDICTED: predicted gene, 19425 (Gm19425), miscRNA.	-7	0.0051
		-3.27	0.0051
Arxes2	adipocyte-related X-chromosome expressed sequence 2	-4.91	0.0051
		-2.01	0.0051
Rpl35a-ps5	ribosomal protein L35A, pseudogene 5	-11.32	0.0052
Tmem47	transmembrane protein 47	-2.59	0.0052
Usp33	ubiquitin specific peptidase 33; Synthetic construct Mus musculus clone IMAGE:100015772, MGC:183431 ubiquitin specific peptidase 33 (Usp33) mRNA, encodes complete protein.	-2.04	0.0052
Gm26135	predicted gene, 26135 [Source:MGI Symbol;Acc:MGI:5455912]	-2.75	0.0052
Rpl21-ps14	ribosomal protein L21, pseudogene 14 [Source:MGI Symbol;Acc:MGI:3648110]	-4.7	0.0052
Gm11443	predicted gene 11443 [Source:MGI Symbol;Acc:MGI:3651160]	-2.32	0.0053
Mir684-1	microRNA 684-1 (Mir684-1), microRNA.; microRNA 684-1	-482.47	0.0053
Mir684-1	microRNA 684-1 (Mir684-1), microRNA.; microRNA 684-1	-482.47	0.0053
Mir684-1	microRNA 684-1 (Mir684-1), microRNA.; microRNA 684-1	-482.47	0.0053
Mir684-1	microRNA 684-1 (Mir684-1), microRNA.; microRNA 684-1	-482.47	0.0053
Mir684-1	microRNA 684-1 (Mir684-1), microRNA.; microRNA 684-1	-482.47	0.0053
		-9.26	0.0053
		-3.34	0.0053
Gm2214	predicted gene 2214 [Source:MGI Symbol;Acc:MGI:3780384]	-3.25	0.0053
Fam174a	family with sequence similarity 174, member A	-2.45	0.0054
Mir297-1	microRNA 297-1	-16.66	0.0054
		-2.88	0.0054
1008; RP23-83(	predicted gene 11008 [Source:MGI Symbol;Acc:MGI:3779224]; novel transcript	-6.51	0.0054

		-3.4	0.0054
		-2.39	0.0054
		-3.58	0.0055
Ppp1cb	protein phosphatase 1, catalytic subunit, beta isoform; protein phosphatase 1, catalytic subunit, beta isoform (Ppp1cb), mRNA.	-3.37	0.0055
		-5.15	0.0055
Gm12643	predicted gene 12643 [Source:MGI Symbol;Acc:MGI:3649676]	-2.51	0.0055
Mir7681	microRNA 7681	2.06	0.0056
Gm14405	predicted gene 14405	-2.4	0.0057
		-6.45	0.0057
		-2.26	0.0058
Gm9703	predicted gene 9703 [Source:MGI Symbol;Acc:MGI:3780110]	-17.63	0.0058
		-2.94	0.0058
Gm14325	predicted gene 14325	-2.28	0.0058
Gm23018	predicted gene, 23018 [Source:MGI Symbol;Acc:MGI:5452795]	-2.08	0.0058
		-3.58	0.0058
Gm12760	predicted gene 12760 [Source:MGI Symbol;Acc:MGI:3649974]	-2.01	0.0059
Gm3625	predicted gene 3625	-7.25	0.0059
		-2.38	0.0059
Gm22677	predicted gene, 22677 [Source:MGI Symbol;Acc:MGI:5452454]	-2.31	0.0059
		-2.11	0.0059
Hspa4	heat shock protein 4	-2.24	0.006
		-3.17	0.006
		-3.03	0.006
		2.21	0.006
Gm22048	predicted gene, 22048	-4.25	0.006
Gm19475	PREDICTED: predicted gene, 19475 (Gm19475), mRNA.	-2.11	0.0061
Gm17709	predicted gene, 17709	-2.86	0.0061
Olfr384	olfactory receptor 384	2.13	0.0061
Pts	6-pyruvoyl-tetrahydropterin synthase	-2.93	0.0061
		-2.71	0.0062
Gm13331	predicted gene 13331 [Source:MGI Symbol;Acc:MGI:3649454]	-2.63	0.0062
		-9.27	0.0062
Cwc15	CWC15 homolog (S. cerevisiae)	-2.06	0.0063
Gm7735	predicted gene 7735 [Source:MGI Symbol;Acc:MGI:3649168]	-2.54	0.0063

		-2.36	0.0063
Gm15484	predicted gene 15484 [Source:MGI Symbol;Acc:MGI:3705726]	-3.96	0.0063
Gm27989	predicted gene, 27989 [Source:MGI Symbol;Acc:MGI:5531371]	-2.4	0.0063
		3.05	0.0063
		-2.22	0.0063
Szrd1	SUZ RNA binding domain containing 1; SUZ RNA binding domain containing 1 (Szrd1), transcript variant 2, mRNA.	-2.14	0.0064
		-3.69	0.0064
Ttc3	tetratricopeptide repeat domain 3; Synthetic construct Mus musculus clone IMAGE:100068069, MGC:195686 tetratricopeptide repeat domain 3 (Ttc3) mRNA, encodes complete protein.	-2.33	0.0064
		2.66	0.0064
Gm11567	predicted gene 11567	2.03	0.0064
Ranbp2-ps8	RAN binding protein 2, pseudogene 8 [Source:MGI Symbol;Acc:MGI:5521016]	-2.17	0.0064
		-6.23	0.0065
		-7.08	0.0065
Gm5093	predicted gene 5093	-2.29	0.0066
Rgs4	regulator of G-protein signaling 4	-2.68	0.0066
		-3.84	0.0067
Gm10175	predicted gene 10175 [Source:MGI Symbol;Acc:MGI:3704287]	-2.91	0.0067
Ighv10-1	immunoglobulin heavy variable 10-1	3.18	0.0067
Rpl34-ps1	ribosomal protein L34, pseudogene 1	-2.72	0.0067
Mir684-1	microRNA 684-1 (Mir684-1), microRNA.; microRNA 684-1	-471.83	0.0068
Eprs	glutamyl-prolyl-tRNA synthetase	-2.5	0.0069
Gm8880	predicted gene 8880 [Source:MGI Symbol;Acc:MGI:3648900]	-3.77	0.0069
		-2.76	0.0069
Eif3s6-ps1	eukaryotic translation initiation factor 3, subunit 6, pseudogene 1	-3.29	0.0069
Gm19752	PREDICTED: predicted gene, 19752 (Gm19752), miscRNA.	-10.12	0.007
Gm4883	predicted gene 4883	-2.15	0.007
Cdk17	cyclin-dependent kinase 17	-2.36	0.007
		-2.65	0.0071
Rps12-ps5	ribosomal protein S12, pseudogene 5	-3.04	0.0071
		-2.35	0.0071
		2.04	0.0071

Gm25935	predicted gene, 25935 [Source:MGI Symbol;Acc:MGI:5455712]	-2.81	0.0071
LOC100503279	PREDICTED: uncharacterized LOC100503279 (LOC100503279), miscRNA.	-6.03	0.0071
		-11.54	0.0071
Gm20094	PREDICTED: predicted gene, 20094 (Gm20094), mRNA.	-5.05	0.0072
		-9.32	0.0072
Gm19474	PREDICTED: predicted gene, 19474, transcript variant 2 (Gm19474), miscRNA.	-8.18	0.0072
Nucks1	nuclear casein kinase and cyclin-dependent kinase substrate 1	-2.56	0.0072
Morf4l1	mortality factor 4 like 1	-2.66	0.0072
		-9.87	0.0072
Gm14412	predicted gene 14412 [Source:MGI Symbol;Acc:MGI:3652251]	-4.55	0.0072
Gm9104	predicted gene 9104 [Source:MGI Symbol;Acc:MGI:3645365]	-2.35	0.0072
		-4.01	0.0073
Mir466f-4	microRNA 466f-4 [Source:MGI Symbol;Acc:MGI:3783374]	-7.32	0.0073
Hnrnpa3; Gm679	heterogeneous nuclear ribonucleoprotein A3; heterogeneous nuclear ribonucleoprotein A3 pseudogene	-3.2	0.0073
Rpl21-ps5	ribosomal protein L21, pseudogene 5 [Source:MGI Symbol;Acc:MGI:3647087]	-2.66	0.0073
Gm10086	predicted pseudogene 10086 [Source:MGI Symbol;Acc:MGI:3642342]	-2.41	0.0073
LOC100861984	PREDICTED: uncharacterized LOC100861984 (LOC100861984), miscRNA.	-2.19	0.0073
Gm15644	predicted gene 15644 [Source:MGI Symbol;Acc:MGI:3783088]	-4.2	0.0073
Bub3	budding uninhibited by benzimidazoles 3 homolog (S. cerevisiae)	-2.58	0.0073
Diaph2	diaphanous related formin 2	-2.63	0.0073
Prps1	phosphoribosyl pyrophosphate synthetase 1	-2.87	0.0074
Gm19868	PREDICTED: predicted gene, 19868 (Gm19868), miscRNA.	-3.15	0.0074
Olfr394	olfactory receptor 394; Synthetic construct Mus musculus clone IMAGE:100014922, MGC:180310 olfactory receptor 394 (Olfr394) mRNA, encodes complete protein.	2.05	0.0074
LOC100862384	PREDICTED: uncharacterized LOC100862384 (LOC100862384), miscRNA.	-2.82	0.0074

Gm14434; 061(	predicted gene 14308 (Gm14308), mRNA.; predicted gene 14430 (Gm14430), mRNA.; predicted gene 14434 (Gm14434), mRNA.; RIKEN cDNA 0610010B08 gene (0610010B08Rik), mRNA.; predicted gene 4724 (Gm4724), mRNA.; predicted gene 14308	-4.21	0.0074
Mir466f-3	microRNA 466f-3	-16.33	0.0074
Rpl3-ps1	ribosomal protein L3, pseudogene 1	-3.86	0.0075
		-2.54	0.0075
Meis2	Meis homeobox 2	-2.62	0.0075
Lrrc4c	leucine rich repeat containing 4C	-3.24	0.0075
Srsf7	serine/arginine-rich splicing factor 7	-2.02	0.0076
Pcm1	pericentriolar material 1	-2.45	0.0077
Gm22016	predicted gene, 22016	2.16	0.0077
Gm14240	predicted gene 14240 [Source:MGI Symbol;Acc:MGI:3650911]	-2.21	0.0077
Gm3601	PREDICTED: predicted gene 3601 (Gm3601), miscRNA.	-50.6	0.0077
Gm16355	predicted gene 16355 [Source:MGI Symbol;Acc:MGI:3840115]	-4.4	0.0077
		-2.7	0.0078
Gm5481	PREDICTED: predicted gene 5481 (Gm5481), mRNA.	-5.72	0.0078
Gvin1; Gm4070	GTPase, very large interferon inducible 1; predicted gene 4070 (Gm4070), transcript variant 1, mRNA.; predicted gene 4070 (Gm4070), transcript variant 2, mRNA.; GTPase, very large interferon inducible 1 (Gvin1), transcript variant 1, mRNA.; Synthetic construct Mus musculus clone IMAGE:100069525, MGC:199427 GTPase, very large interferon inducible 1 (Gvin1) mRNA, encodes complete protein.	-2.16	0.0078
Psmc5	protease (prosome, macropain) 26S subunit, ATPase 5	-2.04	0.0079
Skp1a	S-phase kinase-associated protein 1A	-2.34	0.0079
Gm27525	predicted gene, 27525 [Source:MGI Symbol;Acc:MGI:5530907]	-3.58	0.0079
		-2.05	0.008
Gm24588	predicted gene, 24588 [Source:MGI Symbol;Acc:MGI:5454365]	-2.48	0.008
Gm15682	predicted gene 15682	-3.48	0.0081
Unc13c	unc-13 homolog C (C. elegans)	-2.18	0.0082
5430403G16Rik	RIKEN cDNA 5430403G16 gene [Source:MGI Symbol;Acc:MGI:1924450]	-3.16	0.0082

Gm23990	predicted gene, 23990 [Source:MGI Symbol;Acc:MGI:5453767]	-2.14	0.0082
Gm4024	PREDICTED: predicted pseudogene 4024 (Gm4024), mRNA.	-2.29	0.0082
Psmb5; Mir686	proteasome (prosome, macropain) subunit, beta type 5; microRNA 686	-2.48	0.0084
Gm8822	predicted gene 8822	-3.26	0.0084
Gm13370	predicted gene 13370 [Source:MGI Symbol;Acc:MGI:3651012]	-5.5	0.0084
Gm10240	predicted gene 10240 [Source:MGI Symbol;Acc:MGI:3704454]	-5.5	0.0084
Gm14434; 061(	predicted gene 14308 (Gm14308), mRNA.; predicted gene 14430 (Gm14430), mRNA.; predicted gene 14434 (Gm14434), mRNA.; RIKEN cDNA 0610010B08 gene (0610010B08Rik), mRNA.; predicted gene 4724 (Gm4724), mRNA.; predicted gene 14434; predicted gene 14308	-3.69	0.0084
LOC100861833	PREDICTED: uncharacterized LOC100861833 (LOC100861833), miscRNA.	-9.37	0.0084
Sfpq	splicing factor proline/glutamine rich (polypyrimidine tract binding protein associated)	-3.07	0.0084
Gm9493	predicted gene 9493 [Source:MGI Symbol;Acc:MGI:3779903]	-10.2	0.0084
Eogt	EGF domain-specific O-linked N-acetylglucosamine (GlcNAc) transferase	-2.39	0.0084
m14431; Gm88	predicted gene 14431 (Gm14431), transcript variant 2, mRNA.; predicted gene 8898; predicted gene 14431; predicted gene 8898 (Gm8898), transcript variant 1, mRNA.	-2.04	0.0085
431; Gm8898; G	predicted gene 14431; predicted gene 8898 (Gm8898), transcript variant 2, mRNA.; predicted gene 14431 (Gm14431), transcript variant 1, mRNA.; predicted gene 8898; predicted gene 4245	-2.04	0.0085
Ppp1r12a	protein phosphatase 1, regulatory (inhibitor) subunit 12A	-2.79	0.0085
		4.21	0.0086
Arhgef9	CDC42 guanine nucleotide exchange factor (GEF) 9	-2.71	0.0086
Gm8062	predicted pseudogene 8062 [Source:MGI Symbol;Acc:MGI:3643433]	-3.28	0.0087
		-2.08	0.0087
Gm26070	predicted gene, 26070 [Source:MGI Symbol;Acc:MGI:5455847]	-3.03	0.0088
Gm8580	ribosomal protein L29 pseudogene	-3.02	0.0088
Pea15a	phosphoprotein enriched in astrocytes 15A	-2.43	0.0089

		2.53	0.0089
Gm26599	predicted gene, 26599 [Source:MGI Symbol;Acc:MGI:5477093]	2.09	0.0089
		-2.04	0.009
		-3.41	0.009
Gm4596	predicted gene 4596 [Source:MGI Symbol;Acc:MGI:3782779]	-2.64	0.009
Gm12469	predicted gene 12469 [Source:MGI Symbol;Acc:MGI:3652114]	-5.61	0.009
Gm15464	predicted gene 15464 [Source:MGI Symbol;Acc:MGI:3646025]	-5.87	0.009
Lce1a1	late cornified envelope 1A1	2.02	0.0091
		-4.33	0.0091
		-5.66	0.0092
Ccng1	cyclin G1	-2.16	0.0092
Gm7887	predicted gene 7887 [Source:MGI Symbol;Acc:MGI:3648251]	-3.3	0.0092
Gm26809	predicted gene, 26809 [Source:MGI Symbol;Acc:MGI:5477303]	-2.01	0.0092
Rpl31-ps7	ribosomal protein L31, pseudogene 7	-2.58	0.0092
		-2.44	0.0092
		-2.2	0.0093
Rpl30-ps1	ribosomal protein L30, pseudogene 1	-15.97	0.0093
Gm12411	predicted gene 12411	-4.45	0.0093
		-3.23	0.0093
		-3.1	0.0093
Clk3	CDC-like kinase 3	-2.61	0.0095
Gm11826	predicted gene 11826 [Source:MGI Symbol;Acc:MGI:3651142]	-4.09	0.0095
Kdm7a	lysine (K)-specific demethylase 7A	-2.1	0.0095
Gm19974	PREDICTED: predicted gene, 19974 (Gm19974), miscRNA.	-2.61	0.0095
Gm4705	predicted gene 4705	-3.42	0.0095
Gm14305	predicted gene 14305	-4.69	0.0095
Gm24463	predicted gene, 24463 [Source:MGI Symbol;Acc:MGI:5454240]	-4.77	0.0096
Mir6380	microRNA 6380 [Source:MGI Symbol;Acc:MGI:5530738]	-2.04	0.0096
Gm23388	predicted gene, 23388 [Source:MGI Symbol;Acc:MGI:5453165]	-61.14	0.0096
Ash1l	ash1 (absent, small, or homeotic)-like (Drosophila)	-2.52	0.0096
9430020K01Rik	RIKEN cDNA 9430020K01 gene	-6.29	0.0096
Sept11	septin 11	-2.54	0.0096

Gm12372	predicted gene 12372 [Source:MGI Symbol;Acc:MGI:3650289]	-3.23	0.0097
Ptp4a2	protein tyrosine phosphatase 4a2	-2.03	0.0097
Gm22696	predicted gene, 22696 [Source:MGI Symbol;Acc:MGI:5452473]	-2.71	0.0097
Gm22565	predicted gene, 22565 [Source:MGI Symbol;Acc:MGI:5452342]	-2.12	0.0097
Gm6109	PREDICTED: predicted gene 6109 (Gm6109), mRNA.	-3.63	0.0097
Itpka	inositol 1,4,5-trisphosphate 3-kinase A	-2.22	0.0098
Nudc	nuclear distribution gene C homolog (Aspergillus)	-2.13	0.0098
Gm6206	predicted pseudogene 6206	-2.24	0.0098
Gm4479	predicted gene 4479 [Source:MGI Symbol;Acc:MGI:3782663]	-3.05	0.0098
Gm8172	predicted pseudogene 8172 [Source:MGI Symbol;Acc:MGI:3644930]	-11.82	0.0098
Grm5	glutamate receptor, metabotropic 5	-2	0.0098
Taok1	TAO kinase 1; Synthetic construct Mus musculus clone IMAGE:100069464, MGC:199366 TAO kinase 1 (Taok1) mRNA, encodes complete protein.	-2.02	0.0099
Gm24627	predicted gene, 24627 [Source:MGI Symbol;Acc:MGI:5454404]	-25.29	0.0099
Vimp	VCP-interacting membrane protein	-2.14	0.0099
Gm10031	predicted pseudogene 10031	-3.11	0.0099
Gm19660	PREDICTED: predicted gene, 19660, transcript variant 1 (Gm19660), miscRNA.	-4.36	0.0099
Gm13433	predicted gene 13433 [Source:MGI Symbol;Acc:MGI:3651454]	-3.59	0.01
Gm12837	predicted gene 12837 [Source:MGI Symbol;Acc:MGI:3649425]	2.02	0.01
		-2.49	0.01
ir684-1; Gm134	microRNA 684-1 (Mir684-1), microRNA.; predicted gene 13430; microRNA 684-1	-6.21	0.01
Gm16416	predicted gene 16416 [Source:MGI Symbol;Acc:MGI:3646635]	-3.85	0.0101
Gm9166	predicted gene 9166 [Source:MGI Symbol;Acc:MGI:3648553]	-2.2	0.0101
Gm5614	predicted gene 5614	-2.71	0.0101
Id2	inhibitor of DNA binding 2	-3.67	0.0101
Psme2b	protease (prosome, macropain) activator subunit 2B	-3.14	0.0101
		-2.3	0.0102
Hypk	huntingtin interacting protein K	-2.85	0.0102
		-6.01	0.0102
Gm12666	predicted gene 12666	-2.41	0.0102

		-3.65	0.0103
		2.32	0.0103
		-10.94	0.0103
LOC100862198	PREDICTED: uncharacterized LOC100862198 (LOC100862198), miscRNA.	-3.14	0.0104
Kcnab1	potassium voltage-gated channel, shaker-related subfamily, beta member 1	-3.25	0.0104
Ddx5; Mir3064	DEAD (Asp-Glu-Ala-Asp) box polypeptide 5; microRNA 3064	-2.56	0.0104
		2.52	0.0105
Rab2a	RAB2A, member RAS oncogene family	-4.84	0.0105
Atp2b1	ATPase, Ca++ transporting, plasma membrane 1	-3.96	0.0106
		-2.99	0.0106
Gm25410	predicted gene, 25410 [Source:MGI Symbol;Acc:MGI:5455187]	-2.8	0.0106
Marf1	meiosis arrest female 1	-2.12	0.0107
Eif4e	eukaryotic translation initiation factor 4E	-2.91	0.0107
Gm17786	predicted gene, 17786 [Source:MGI Symbol;Acc:MGI:5009950]	-2.47	0.0107
Gm10916	predicted gene 10916 [Source:MGI Symbol;Acc:MGI:3779123]	-2.64	0.0107
Rgs9	regulator of G-protein signaling 9	-5.08	0.0108
Rab10	RAB10, member RAS oncogene family	-3.27	0.0108
3632; RP24-96I	predicted gene 13632 [Source:MGI Symbol;Acc:MGI:3651357]; novel transcript	-3.29	0.0108
Gm12117	predicted gene 12117 [Source:MGI Symbol;Acc:MGI:3652019]	-2.4	0.0109
14929; RP23-79	predicted gene 14929 [Source:MGI Symbol;Acc:MGI:3705167]; novel transcript	-6.41	0.0109
		2.43	0.0109
Mir297a-2	microRNA 297a-2	-26.18	0.0109
		-5.98	0.0109
Gpr88	G-protein coupled receptor 88	-5.59	0.0109
Bcr	breakpoint cluster region	-2.23	0.0109
		2.01	0.011
Gm23637	predicted gene, 23637 [Source:MGI Symbol;Acc:MGI:5453414]	-4.06	0.011
		-3.71	0.0111
Adrb2	adrenergic receptor, beta 2	-3.34	0.0111
Gm15739	predicted gene 15739 [Source:MGI Symbol;Acc:MGI:3783181]	3.74	0.0112
Gm16089	predicted gene 16089 [Source:MGI Symbol;Acc:MGI:3801884]	-4.9	0.0112
Rpl3-ps2	ribosomal protein L3, pseudogene 2	-8.09	0.0112

Rps3a3	ribosomal protein S3A3 [Source:MGI Symbol;Acc:MGI:3643406]	-8.53	0.0113
Gm16021	predicted gene 16021 [Source:MGI Symbol;Acc:MGI:3802111]	-2.23	0.0113
Mir297a-3	microRNA 297a-3 [Source:MGI Symbol;Acc:MGI:3718465]	-2.4	0.0114
		2.12	0.0114
Cdk19	cyclin-dependent kinase 19	-2.2	0.0115
Gm11253	predicted gene 11253 [Source:MGI Symbol;Acc:MGI:3649981]	-2.4	0.0115
		-2.07	0.0115
		-16.61	0.0116
		-4.58	0.0116
Rps23-ps2	ribosomal protein S23, pseudogene 2	-5.86	0.0116
Gm7206	predicted pseudogene 7206 [Source:MGI Symbol;Acc:MGI:3646634]	-9.01	0.0116
Gm26683	predicted gene, 26683 [Source:MGI Symbol;Acc:MGI:5477177]	-2.08	0.0117
Gm26904	predicted gene, 26904 [Source:MGI Symbol;Acc:MGI:5477398]	-2.09	0.0117
Ndufv2	NADH dehydrogenase (ubiquinone) flavoprotein 2	-2.26	0.0117
		2.31	0.0118
		4.99	0.0118
Chchd3	coiled-coil-helix-coiled-coil-helix domain containing 3	-2.29	0.0118
Akt3	thymoma viral proto-oncogene 3	-4.19	0.0118
Gm23551	predicted gene, 23551 [Source:MGI Symbol;Acc:MGI:5453328]	-23.7	0.0119
		-3.29	0.0119
		-2.19	0.012
Gm13680	predicted gene 13680 [Source:MGI Symbol;Acc:MGI:3652285]	-5.15	0.012
LOC100861882	PREDICTED: uncharacterized LOC100861882, transcript variant 1 (LOC100861882), miscRNA.; PREDICTED: uncharacterized LOC100861882, transcript variant 2 (LOC100861882), miscRNA.	-12.87	0.0121
Gm27397	predicted gene, 27397 [Source:MGI Symbol;Acc:MGI:5530779]	-2.73	0.0121
Gm5830	predicted pseudogene 5830 [Source:MGI Symbol;Acc:MGI:3647375]	-2.15	0.0121
Gm14326	predicted gene 14326	-10.89	0.0121
		-11.75	0.0121
Anapc10	anaphase promoting complex subunit 10	-3.35	0.0122
Atxn10	ataxin 10	-2.79	0.0123

Gm10154	predicted gene 10154 [Source:MGI Symbol;Acc:MGI:3642271]	-2.78	0.0123
Bag1	BCL2-associated athanogene 1	-4.77	0.0123
Tspan3	tetraspanin 3	-2.17	0.0123
		-2.56	0.0124
Gm7363	predicted gene 7363 [Source:MGI Symbol;Acc:MGI:3646407]	-4.23	0.0124
		-2.24	0.0124
		-23.69	0.0126
		-5.65	0.0126
Gm14409	predicted gene 14409 [Source:MGI Symbol;Acc:MGI:3649811]	-2.39	0.0127
		-2.98	0.0127
		2.42	0.0128
		-2.01	0.0128
Med13	mediator complex subunit 13	-2.02	0.0129
		-2.08	0.0129
Rpl3	ribosomal protein L3	-7.32	0.013
26978; RP23-4I	predicted gene, 26978 [Source:MGI Symbol;Acc:MGI:5504093]; novel transcript, sense intronic to Rbpms	2.05	0.013
		-14.87	0.013
Vapa	vesicle-associated membrane protein, associated protein A	-2.61	0.013
Gm16200	predicted gene 16200 [Source:MGI Symbol;Acc:MGI:3801928]	-2.71	0.0131
Scn4b	sodium channel, type IV, beta	-2.17	0.0131
Gm27178	predicted gene 27178 [Source:MGI Symbol;Acc:MGI:5521021]	-2.02	0.0132
LOC100861642	PREDICTED: uncharacterized LOC100861642 (LOC100861642), miscRNA.	-10.13	0.0132
Gm6767	predicted gene 6767 [Source:MGI Symbol;Acc:MGI:3645053]	-3.71	0.0132
		-3.48	0.0133
		-2.57	0.0133
Gm10182	predicted pseudogene 10182 [Source:MGI Symbol;Acc:MGI:3704327]	-3.36	0.0133
LOC100862175	PREDICTED: uncharacterized LOC100862175 (LOC100862175), miscRNA.	-2.58	0.0133
Mir467h	microRNA 467h [Source:MGI Symbol;Acc:MGI:3783381]	-3.42	0.0134
		-4.83	0.0134
Mgp	matrix Gla protein	-4.08	0.0135

Gprasp2	G protein-coupled receptor associated sorting protein 2	-2.14	0.0135
Gm11263	predicted gene 11263 [Source:MGI Symbol;Acc:MGI:3651899]	-4.57	0.0135
LOC10086208	PREDICTED: uncharacterized LOC100862081 (LOC100862081), miscRNA.	-2.55	0.0136
		-2.14	0.0137
		-3.74	0.0138
		-3	0.0139
Atp2a2	ATPase, Ca++ transporting, cardiac muscle, slow twitch 2	-3.09	0.0139
Gm26469	predicted gene, 26469 [Source:MGI Symbol;Acc:MGI:5456246]	-8.43	0.014
		-4.6	0.0141
Pde10a	phosphodiesterase 10A	-2.53	0.0141
Gm13422	predicted gene 13422 [Source:MGI Symbol;Acc:MGI:3650167]	-2.56	0.0142
Gm23700	predicted gene, 23700 [Source:MGI Symbol;Acc:MGI:5453477]	-3.78	0.0142
		-2.38	0.0143
		-2.27	0.0143
Gm16100	predicted gene 16100 [Source:MGI Symbol;Acc:MGI:3802060]	-3.59	0.0143
		2.17	0.0143
		-3.92	0.0143
Gm22824	predicted gene, 22824 [Source:MGI Symbol;Acc:MGI:5452601]	-2.43	0.0144
Gm27430	predicted gene, 27430 [Source:MGI Symbol;Acc:MGI:5530812]	-2.19	0.0144
LOC10086208	PREDICTED: uncharacterized LOC100862086 (LOC100862086), miscRNA.	-4.99	0.0144
Gm22197	predicted gene, 22197 [Source:MGI Symbol;Acc:MGI:5451974]	-3.2	0.0145
Rpl30-ps8	ribosomal protein L30, pseudogene 8 [Source:MGI Symbol;Acc:MGI:3643509]	-14.64	0.0145
Rpl23a-ps2	ribosomal protein L23A, pseudogene 2	-3.34	0.0146
		2.07	0.0146
nbp1l; Mir765	formin binding protein 1-like; microRNA 7657	-2.01	0.0147
Gm10243	predicted gene 10243	-4.94	0.0147
Fkbp3	FK506 binding protein 3	-6.94	0.0147
		2.56	0.0148
Gm27892	predicted gene, 27892 [Source:MGI Symbol;Acc:MGI:5531274]	-3.18	0.0148

Gm19579	PREDICTED: predicted gene, 19579 (Gm19579), miscRNA.	-11.28	0.0148
Fam189a2	family with sequence similarity 189, member A2	-2.04	0.0148
		-16.77	0.0148
Gm27704	predicted gene, 27704 [Source:MGI Symbol;Acc:MGI:5531086]	-4.71	0.0149
Ranbp9	RAN binding protein 9	-2.51	0.0149
Rpl14-ps1	ribosomal protein L14, pseudogene 1 [Source:MGI Symbol;Acc:MGI:3710579]	-5.47	0.015
Rpl30-ps9	ribosomal protein L30, pseudogene 9	-15.06	0.015
Gm25040	predicted gene, 25040 [Source:MGI Symbol;Acc:MGI:5454817]	-54.66	0.015
		-2.83	0.015
Gm10045	predicted pseudogene 10045	-4.25	0.015
Gm25157	predicted gene, 25157 [Source:MGI Symbol;Acc:MGI:5454934]	-31.92	0.0151
Gm22941	predicted gene, 22941 [Source:MGI Symbol;Acc:MGI:5452718]	-31.92	0.0151
Gm22258	predicted gene, 22258 [Source:MGI Symbol;Acc:MGI:5452035]	-31.92	0.0151
; Gm14434; 061	predicted gene 14308; predicted gene 14430; predicted gene 14432; predicted gene 14434 (Gm14434), mRNA.; RIKEN cDNA 0610010B08 gene (0610010B08Rik), mRNA.; predicted gene 4724 (Gm4724), mRNA.; predicted gene 4631; predicted gene 4724; RIKEN cDNA 0610010B08 gene	-2.45	0.0151
Mir669d-2	microRNA 669d-2 [Source:MGI Symbol;Acc:MGI:4834278]	-2.44	0.0152
		-2.64	0.0152
		2.2	0.0153
Gm27731	predicted gene, 27731 [Source:MGI Symbol;Acc:MGI:5531113]	-3.21	0.0154
		-3.3	0.0154
Gm5210	predicted gene 5210 [Source:MGI Symbol;Acc:MGI:3644379]	-3.03	0.0154
Pfdn5	prefoldin 5	-3.66	0.0154
		2.02	0.0155
		-2.13	0.0155
Rpl21-ps3	ribosomal protein L21, pseudogene 3 [Source:MGI Symbol;Acc:MGI:3646431]	-2.21	0.0155
		-2.6	0.0156
5298; RP23-232	predicted gene 15298 [Source:MGI Symbol;Acc:MGI:3705276]; putative novel transcript	-4.06	0.0156

		5.24	0.0156
Pcnp	PEST proteolytic signal containing nuclear protein	-2.01	0.0157
Gm22715	predicted gene, 22715 [Source:MGI Symbol;Acc:MGI:5452492]	-2.32	0.0157
		-2.17	0.0157
		2.13	0.0157
Rps12-ps24	ribosomal protein S12, pseudogene 24	-3.69	0.0157
Gm25401	predicted gene, 25401 [Source:MGI Symbol;Acc:MGI:5455178]	-2.11	0.0157
		-2.42	0.0158
		-3.16	0.0158
Gm16111	predicted gene 16111 [Source:MGI Symbol;Acc:MGI:3801919]	-2.41	0.0158
Gm24148	predicted gene, 24148	-2.03	0.0159
Gm22205	predicted gene, 22205 [Source:MGI Symbol;Acc:MGI:5451982]	-23.17	0.0159
Gm14393	predicted gene 14393	-3.03	0.0159
Gm2004	predicted gene 2004	-2.89	0.016
Mir5622	microRNA 5622	2	0.016
Slc6a1	solute carrier family 6 (neurotransmitter transporter, GABA), member 1	-3.03	0.0162
		-2.77	0.0162
Rpl36a-ps3	ribosomal protein L36A, pseudogene 3	-3.29	0.0162
		-7.38	0.0163
Rpl21-ps12	ribosomal protein L21, pseudogene 12 [Source:MGI Symbol;Acc:MGI:3648214]	-2.95	0.0163
Gap43	growth associated protein 43	-3.04	0.0163
		-3.01	0.0164
		-2.37	0.0164
Gm26701	predicted gene, 26701 [Source:MGI Symbol;Acc:MGI:5477195]	-2.76	0.0164
Rab28	RAB28, member RAS oncogene family	-2.35	0.0164
Clk1	CDC-like kinase 1; Synthetic construct Mus musculus clone IMAGE:100064101, MGC:193472 CDC-like kinase 1 (Clk1) mRNA, encodes complete protein.; CDC-like kinase 1 (Clk1), transcript variant 2, non-coding RNA.	-3.06	0.0164
Gm6822	predicted pseudogene 6822	-2.58	0.0165
Supt4b	predicted gene 3258	-2.27	0.0165
Gm6570	predicted gene 6570	-6.72	0.0165
Gm27440	predicted gene, 27440 [Source:MGI Symbol;Acc:MGI:5530822]	-4.21	0.0165
Gm25041	predicted gene, 25041 [Source:MGI Symbol;Acc:MGI:5454818]	-2.24	0.0165

Gm22243	predicted gene, 22243 [Source:MGI Symbol;Acc:MGI:5452020]	-5.85	0.0165
Gm13604	predicted gene 13604 [Source:MGI Symbol;Acc:MGI:3652203]	-3.74	0.0166
		-2.22	0.0167
Camk4	calcium/calmodulin-dependent protein kinase IV	-2.21	0.0167
		-2.41	0.0168
		-2.71	0.0168
Vmn1r-ps123	vomeronasal 1 receptor, pseudogene 123 [Source:MGI Symbol;Acc:MGI:4439075]	2.17	0.0168
		-4.27	0.0168
Rpl35a-ps7	ribosomal protein L35A, pseudogene 7	-3.2	0.0168
Qk	quaking	-2.1	0.0168
Gatm	glycine amidinotransferase (L-arginine:glycine amidinotransferase)	-2.58	0.0169
Ndufs4	NADH dehydrogenase (ubiquinone) Fe-S protein 4	-2.41	0.0169
		-2.83	0.0169
Mir669m-1	microRNA 669m-1	-2.34	0.017
15656; AC1177	predicted gene 15656 [Source:MGI Symbol;Acc:MGI:3783099]; Novel Transcript	2.21	0.017
Gm27450	predicted gene, 27450 [Source:MGI Symbol;Acc:MGI:5530832]	-2.85	0.017
		-5.64	0.017
Ogt	O-linked N-acetylglucosamine (GlcNAc) transferase (UDP-N-acetylglucosamine:polypeptide-N-acetylglucosaminyl transferase); O-linked N-acetylglucosamine (GlcNAc) transferase (UDP-N-acetylglucosamine:polypeptide-N-acetylglucosaminyl transferase) (Ogt), mRNA.	-4.49	0.0171
.4041; RP23-37	predicted gene 14041 [Source:MGI Symbol;Acc:MGI:3651081]; novel transcript	2.02	0.0171
Gdi1	guanosine diphosphate (GDP) dissociation inhibitor 1	-3.87	0.0171
		-2.3	0.0171
		-3.37	0.0172
Gm27341	predicted gene, 27341 [Source:MGI Symbol;Acc:MGI:5530723]	-4.76	0.0173
Gm27038	predicted gene, 27038 [Source:MGI Symbol;Acc:MGI:5504153]	-2.88	0.0173
		-3.11	0.0174
Gm6758	predicted gene 6758 [Source:MGI Symbol;Acc:MGI:3643216]	-2.01	0.0175
Mir466h	microRNA 466h	-11.33	0.0175
Rps11-ps2	ribosomal protein S11, pseudogene 2	-2.54	0.0175
		-3.08	0.0175

Slc6a11	solute carrier family 6 (neurotransmitter transporter, GABA), member 11	-2.46	0.0176
Nedd4	neural precursor cell expressed, developmentally down-regulated 4	-2.8	0.0176
Gm11852	predicted gene 11852 [Source:MGI Symbol;Acc:MGI:3649491]	-2.17	0.0176
Gm12191	predicted gene 12191 [Source:MGI Symbol;Acc:MGI:3651293]	-16.15	0.0177
Atp5g3	ATP synthase, H+ transporting, mitochondrial F0 complex, subunit C3 (subunit 9)	-2.03	0.0177
		-3.2	0.0177
Rpl7	ribosomal protein L7	-2.08	0.0177
Itm2b	integral membrane protein 2B	-11.11	0.0178
Gm23237	predicted gene, 23237 [Source:MGI Symbol;Acc:MGI:5453014]	-2.76	0.0179
Gm22461	predicted gene, 22461 [Source:MGI Symbol;Acc:MGI:5452238]	-2.31	0.018
		-4.14	0.018
		2.27	0.0181
Igfbp7	insulin-like growth factor binding protein 7	-2.84	0.0183
Npm1	nucleophosmin 1	-2.38	0.0183
Gm9174	predicted pseudogene 9174 [Source:MGI Symbol;Acc:MGI:3643688]	-6.43	0.0184
Gm13653	predicted gene 13653	-3.2	0.0184
Gm27437	predicted gene, 27437 [Source:MGI Symbol;Acc:MGI:5530819]	-4.91	0.0184
Gm26199	predicted gene, 26199	-4.95	0.0185
Rpl21-ps7	ribosomal protein L21, pseudogene 7 [Source:MGI Symbol;Acc:MGI:3646135]	-2.54	0.0185
Gm19831	PREDICTED: predicted gene, 19831, transcript variant 1 (Gm19831), miscRNA.; PREDICTED: predicted gene, 19831, transcript variant 2 (Gm19831), miscRNA.; PREDICTED: predicted gene, 19831, transcript variant 3 (Gm19831), miscRNA.; PREDICTED: predicted gene, 19831, transcript variant 4 (Gm19831), miscRNA.; PREDICTED: predicted gene, 19831 (Gm19831), miscRNA.	-28.56	0.0185
Gm22474	predicted gene, 22474 [Source:MGI Symbol;Acc:MGI:5452251]	-4.83	0.0185
Vmn1r198	vomeronasal 1 receptor 198	2.24	0.0186
Gm24587	predicted gene, 24587 [Source:MGI Symbol;Acc:MGI:5454364]	-2.18	0.0186
		2.07	0.0186

		2	0.0187
		-2.43	0.0187
Gm16380	predicted pseudogene 16380 [Source:MGI Symbol;Acc:MGI:3643564]	-2.28	0.0187
Gm13835	predicted gene 13835	-13.76	0.0187
Gm6987	predicted pseudogene 6987	-2.1	0.0188
Sf3b1	splicing factor 3b, subunit 1	-2.27	0.0188
		-2.3	0.0188
		-12.36	0.0189
Apoo-ps	apolipoprotein O, pseudogene [Source:MGI Symbol;Acc:MGI:3649039]	-3.99	0.019
Gm22389	predicted gene, 22389	2.66	0.019
Lrrc7	leucine rich repeat containing 7	-2.26	0.0191
Gm27910	predicted gene, 27910 [Source:MGI Symbol;Acc:MGI:5531292]	-5.31	0.0192
Gm6433	predicted gene 6433 [Source:MGI Symbol;Acc:MGI:3645394]	-2.37	0.0192
		-2.39	0.0192
Gm2389	predicted pseudogene 2389 [Source:MGI Symbol;Acc:MGI:3780557]	-2.35	0.0192
Mir467e	microRNA 467e [Source:MGI Symbol;Acc:MGI:3718541]	-2.09	0.0192
LOC10086231[illegible]	PREDICTED: uncharacterized LOC100862318, transcript variant 1 (LOC100862318), miscRNA.; PREDICTED: uncharacterized LOC100862318, transcript variant 2 (LOC100862318), miscRNA.; PREDICTED: uncharacterized LOC100862318, transcript variant 3 (LOC100862318), miscRNA.	-2.06	0.0193
Rps18-ps1	ribosomal protein S18, pseudogene 1	-2.99	0.0193
Gm14416	predicted gene 14416	-3.15	0.0193
		-5.74	0.0193
Gm23564	predicted gene, 23564 [Source:MGI Symbol;Acc:MGI:5453341]	-2.2	0.0194
Gm24510	predicted gene, 24510 [Source:MGI Symbol;Acc:MGI:5454287]	-2.25	0.0195
		-2.21	0.0196
D030029J20Ri[illegible]	PREDICTED: RIKEN cDNA D030029J20 gene (D030029J20Rik), miscRNA.	-2.72	0.0196
Efr3b	EFR3 homolog B (S. cerevisiae)	-3.23	0.0197
		-2.12	0.0197
Rps4x	ribosomal protein S4, X-linked; ribosomal protein S4, X-linked (Rps4x), mRNA.	-2.53	0.0197
		-2.66	0.0197
Pitpnb	phosphatidylinositol transfer protein, beta	-2.71	0.0197

Gm16415	predicted pseudogene 16415 [Source:MGI Symbol;Acc:MGI:3647855]	-2.35	0.0198
Gm13641	predicted gene 13641 [Source:MGI Symbol;Acc:MGI:3651867]	-2.74	0.0198
		-9.15	0.0198
		-3.37	0.0199
		-3.56	0.02
Gm8618	predicted gene 8618	-3.54	0.02
		-2.85	0.02
Rps12-ps19	ribosomal protein S12, pseudogene 19 [Source:MGI Symbol;Acc:MGI:3652063]	-3.33	0.02
LOC100862063	PREDICTED: uncharacterized LOC100862063 (LOC100862063), miscRNA.	-2.3	0.02
Gm16409	predicted gene 16409 [Source:MGI Symbol;Acc:MGI:3646708]	-7.09	0.02
Gm27839	predicted gene, 27839 [Source:MGI Symbol;Acc:MGI:5531221]	-5.35	0.0201
LOC100861832	PREDICTED: uncharacterized LOC100861832 (LOC100861832), miscRNA.	-2.14	0.0201
Polr2k	polymerase (RNA) II (DNA directed) polypeptide K	-2.18	0.0202
Rps10-ps2	ribosomal protein S10, pseudogene 2	-2.05	0.0202
Gm23029	predicted gene, 23029	-2.21	0.0202
		-7.05	0.0203
Gm13268	predicted gene 13268	-2.85	0.0203
Gm22607	predicted gene, 22607 [Source:MGI Symbol;Acc:MGI:5452384]	2.5	0.0203
Gm26202	predicted gene, 26202 [Source:MGI Symbol;Acc:MGI:5455979]	-2.69	0.0204
4632427E13Rik	RIKEN cDNA 4632427E13 gene	-2.33	0.0204
		-5.36	0.0205
Gm5451	predicted gene 5451	-6.32	0.0206
359; AF357425	RNA imprinted and accumulated in nucleus; snoRNA AF357355; snoRNA AF357359; snoRNA AF357425; snoRNA DQ267100; snoRNA DQ267101; snoRNA AF357359 (AF357359), small nucleolar RNA.	-2.21	0.0207
Gm4778	predicted gene 4778 [Source:MGI Symbol;Acc:MGI:3643869]	2.02	0.0208
		-2.68	0.0208
		-2.9	0.0208
Itih5	inter-alpha (globulin) inhibitor H5	-2.04	0.0208
		-6.3	0.0208
		-2.05	0.0209

18O10Rik; Gm	RIKEN cDNA 2210418O10 gene [Source:MGI Symbol;Acc:MGI:1924208]; predicted gene, 17258 [Source:MGI Symbol;Acc:MGI:4936892]	-2.12	0.0209
		-2.02	0.0209
Gm23434	predicted gene, 23434 [Source:MGI Symbol;Acc:MGI:5453211]	2.71	0.021
Hnrnph1	heterogeneous nuclear ribonucleoprotein H1	-2.06	0.021
		-3.1	0.0211
Gm16354	predicted gene 16354 [Source:MGI Symbol;Acc:MGI:3840116]	-3.74	0.0211
Ubr3	ubiquitin protein ligase E3 component n-recognin 3 (Ubr3), transcript variant 1, mRNA.; ubiquitin protein ligase E3 component n-recognin 3	-2.01	0.0212
Gm6274	predicted gene 6274 [Source:MGI Symbol;Acc:MGI:3645367]	-2.84	0.0213
Gm27219	predicted gene 27219 [Source:MGI Symbol;Acc:MGI:5521062]	-2.25	0.0213
Gpr37l1	G protein-coupled receptor 37-like 1	-2.06	0.0213
Gm4468	predicted gene 4468 [Source:MGI Symbol;Acc:MGI:3782652]	-2.21	0.0213
Gm23364	predicted gene, 23364 [Source:MGI Symbol;Acc:MGI:5453141]	-2.5	0.0214
Gm24025	predicted gene, 24025 [Source:MGI Symbol;Acc:MGI:5453802]	-2.5	0.0214
Rpl21-ps6	ribosomal protein L21, pseudogene 6 [Source:MGI Symbol;Acc:MGI:3643554]	-2.1	0.0214
		2.03	0.0214
		-2.08	0.0215
Gm9719	predicted gene 9719 [Source:MGI Symbol;Acc:MGI:3780128]	-4.3	0.0216
Gm7429	predicted pseudogene 7429	-9.75	0.0216
4681; LOC1008	predicted gene 14681 [Source:MGI Symbol;Acc:MGI:3705734]; PREDICTED: uncharacterized LOC100861774 (LOC100861774), miscRNA.	-7.42	0.0217
Sparc	secreted acidic cysteine rich glycoprotein	-2.28	0.0217
Gm22838	predicted gene, 22838 [Source:MGI Symbol;Acc:MGI:5452615]	3.14	0.0219
		-2.73	0.022
n14288; Gm144	predicted gene 14288; predicted gene 14440 (Gm14440), mRNA.; predicted gene 14440	-3.46	0.022
Gm14633	predicted gene 14633 [Source:MGI Symbol;Acc:MGI:3705509]	-4.98	0.022

Gm11531	predicted gene 11531 [Source:MGI Symbol;Acc:MGI:3650260]	-4.92	0.022
Nrbp2	nuclear receptor binding protein 2	-2.56	0.0221
Micu3	mitochondrial calcium uptake family, member 3	-2.1	0.0221
Tomm20	translocase of outer mitochondrial membrane 20 homolog (yeast)	-3.17	0.0221
Gm5776	predicted gene 5776	-3.64	0.0222
Rap2a	RAS related protein 2a	-2.06	0.0222
		-2.46	0.0223
		2.58	0.0223
Adam25	a disintegrin and metallopeptidase domain 25 (testase 2)	2.63	0.0223
Hsp90b1	heat shock protein 90, beta (Grp94), member 1	-3.54	0.0224
Gm24093	predicted gene, 24093	-3.03	0.0224
Nkain2	Na+/K+ transporting ATPase interacting 2	-2.11	0.0225
Cfdp1	craniofacial development protein 1	-3.31	0.0225
		2.03	0.0226
Gm15309	predicted gene 15309 [Source:MGI Symbol;Acc:MGI:3705458]	-2.54	0.0226
Gm22854	predicted gene, 22854 [Source:MGI Symbol;Acc:MGI:5452631]	-2.76	0.0226
		2.35	0.0227
Gm25171	predicted gene, 25171 [Source:MGI Symbol;Acc:MGI:5454948]	-35.43	0.0228
Gm25110	predicted gene, 25110 [Source:MGI Symbol;Acc:MGI:5454887]	-35.43	0.0228
Gm25775	predicted gene, 25775 [Source:MGI Symbol;Acc:MGI:5455552]	-35.43	0.0228
Gm24795	predicted gene, 24795 [Source:MGI Symbol;Acc:MGI:5454572]	-35.43	0.0228
Gm12174	predicted gene 12174 [Source:MGI Symbol;Acc:MGI:3651722]	-23.8	0.0228
Mir466f-1	microRNA 466f-1 [Source:MGI Symbol;Acc:MGI:3718533]	-2.15	0.0229
		-4.23	0.0229
Acbd6	acyl-Coenzyme A binding domain containing 6	-2.52	0.023
		-3.01	0.0231
Gm25297	predicted gene, 25297 [Source:MGI Symbol;Acc:MGI:5455074]	-6.01	0.0231
Dynlt1a	dynein light chain Tctex-type 1A; dynein light chain Tctex-type 1A (Dynlt1a), mRNA.	-2.63	0.0231
Rps19bp1	ribosomal protein S19 binding protein 1	-2.03	0.0233
Vmn1r89	vomeronasal 1 receptor 89	2	0.0234
Polr2g	polymerase (RNA) II (DNA directed) polypeptide G	-2.07	0.0235

Spin1	spindlin 1; spindlin 1 (Spin1), transcript variant 1, mRNA.	-2.67	0.0235
Gm10177	predicted gene 10177	-11.87	0.0235
Gm14322	predicted gene 14322	-3.02	0.0235
Gm15616	predicted gene 15616 [Source:MGI Symbol;Acc:MGI:3783061]	-3.02	0.0236
Gm25508	predicted gene, 25508	-2.59	0.0237
Gm10224	predicted pseudogene 10224	-3.68	0.0237
Gm8927	predicted gene 8927 [Source:MGI Symbol;Acc:MGI:3643156]	-3.12	0.0238
Gm10343	predicted gene 10343 [Source:MGI Symbol;Acc:MGI:3642690]	-9.89	0.0239
		-4.06	0.0239
n14288; Gm144	predicted gene 14288 (Gm14288), mRNA.; predicted gene 14435	-2.06	0.0239
Slc22a8	solute carrier family 22 (organic anion transporter), member 8	-2.57	0.024
		-2.55	0.024
		-3.49	0.0241
Rps24-ps3	ribosomal protein S24, pseudogene 3	-6.36	0.0241
		-3.15	0.0241
Rpl30-ps10	ribosomal protein L30, pseudogene 10	-13.97	0.0242
Gm24192	predicted gene, 24192 [Source:MGI Symbol;Acc:MGI:5453969]	-2.05	0.0243
Hnrnpk	heterogeneous nuclear ribonucleoprotein K	-2.03	0.0243
Rps15a-ps3	ribosomal protein S15A, pseudogene 3 [Source:MGI Symbol;Acc:MGI:3650886]	-4.51	0.0245
Rpl17-ps1	ribosomal protein L17, pseudogene 1	-2.62	0.0245
		-4.46	0.0247
Gm16363	predicted gene 16363 [Source:MGI Symbol;Acc:MGI:3840139]	-2.43	0.0247
		-2.02	0.0247
Gm10653	ribsomal protein S2 pseudogene; predicted gene 10653	-2.63	0.0248
Gm26475	predicted gene, 26475 [Source:MGI Symbol;Acc:MGI:5456252]	-23.74	0.0249
		-2.74	0.0249
Gm10247	predicted gene 10247 [Source:MGI Symbol;Acc:MGI:3641838]	-6.17	0.0249
Gm8264	PREDICTED: predicted gene 8264 (Gm8264), mRNA.	-2.33	0.0249
Gm8137	PREDICTED: predicted gene 8137 (Gm8137), mRNA.	-2.33	0.0249
Dstn	destrin	-2.13	0.0252

Rps12-ps1	ribosomal protein S12, pseudogene 1	-2.76	0.0252
		-2.31	0.0253
Gstm1	glutathione S-transferase, mu 1	-2.57	0.0253
Gm7308	predicted pseudogene 7308 [Source:MGI Symbol;Acc:MGI:3644558]	-4.01	0.0254
		-2.75	0.0254
Bmpr2	bone morphogenetic protein receptor, type II (serine/threonine kinase); Synthetic construct Mus musculus clone IMAGE:100062725, MGC:190942 bone morphogenic protein receptor, type II (serine/threonine kinase) (Bmpr2) mRNA, encodes complete protein.	-4.02	0.0255
Gdi2	guanosine diphosphate (GDP) dissociation inhibitor 2	-2.64	0.0256
Gm24144	predicted gene, 24144 [Source:MGI Symbol;Acc:MGI:5453921]	-2.31	0.0256
Gm24766	predicted gene, 24766 [Source:MGI Symbol;Acc:MGI:5454543]	-19.41	0.0256
Gm25817	predicted gene, 25817 [Source:MGI Symbol;Acc:MGI:5455594]	-19.41	0.0256
		-3.38	0.0256
Immp1l	IMP1 inner mitochondrial membrane peptidase-like (S. cerevisiae)	-8.7	0.0257
Gm24927	predicted gene, 24927 [Source:MGI Symbol;Acc:MGI:5454704]	-4.24	0.0257
Gm12508	predicted gene 12508	-3.05	0.0257
Mal	myelin and lymphocyte protein, T cell differentiation protein	-2.64	0.0257
LOC432823	similar to hypothetical protein MGC37588, mRNA (cDNA clone MGC:28125 IMAGE:3980327), complete cds.	-4.96	0.0258
		-2.01	0.0258
Gm11099	predicted gene 11099 [Source:MGI Symbol;Acc:MGI:3779335]	2.42	0.0258
Rpl9-ps6	ribosomal protein L9, pseudogene 6 [Source:MGI Symbol;Acc:MGI:3642682]	-5.05	0.0259
Slc32a1	solute carrier family 32 (GABA vesicular transporter), member 1	-2.08	0.0259
Gm14284	predicted gene 14284 [Source:MGI Symbol;Acc:MGI:3650419]	-2.1	0.0259
Mir5109	microRNA 5109 (Mir5109), microRNA.	-2.4	0.0259
Mir669m-2	microRNA 669m-2	-5.94	0.026
Gm23072	predicted gene, 23072 [Source:MGI Symbol;Acc:MGI:5452849]	-2	0.026

BC002163	NADH dehydrogenase Fe-S protein 5 pseudogene; cDNA sequence BC002163	-2.66	0.026
		2.33	0.0261
Gm23445	predicted gene, 23445 [Source:MGI Symbol;Acc:MGI:5453222]	2.25	0.0262
Mir669n	microRNA 669n [Source:MGI Symbol;Acc:MGI:3837039]	-2.89	0.0262
Gm22188	predicted gene, 22188 [Source:MGI Symbol;Acc:MGI:5451965]	-24.31	0.0262
Hsbp1	heat shock factor binding protein 1	-12.39	0.0262
Gm14387	predicted gene 14387	-2.1	0.0263
		-5.88	0.0263
Rps3a2	ribosomal protein S3A2 [Source:MGI Symbol;Acc:MGI:3642853]	-7.65	0.0263
Gm10071	predicted gene 10071 [Source:MGI Symbol;Acc:MGI:3642685]; predicted gene 10071	-2.68	0.0263
		2.19	0.0263
		2.36	0.0263
		-2.19	0.0265
Rpl9-ps4	ribosomal protein L9, pseudogene 4 [Source:MGI Symbol;Acc:MGI:3704410]	-7.11	0.0267
		2.43	0.0268
Rps18-ps3	ribosomal protein S18, pseudogene 3 [Source:MGI Symbol;Acc:MGI:3642474]	-3.64	0.0269
Mir466h	microRNA 466h [Source:MGI Symbol;Acc:MGI:3718537]	-2.43	0.0269
Rpl9-ps7	ribosomal protein L9, pseudogene 7	-3.95	0.027
Gm5446	predicted gene 5446 [Source:MGI Symbol;Acc:MGI:3644854]	-2.45	0.0271
Rpl4	ribosomal protein L4	-3.19	0.0271
Gm8054	predicted pseudogene 8054 [Source:MGI Symbol;Acc:MGI:3643693]	-3.44	0.0271
Sepw1	selenoprotein W, muscle 1	-2.24	0.0271
Gm11688	predicted gene 11688 [Source:MGI Symbol;Acc:MGI:3650868]	-2.53	0.0271
Gm17014	predicted gene 17014	-2.12	0.0273
Olfr591	olfactory receptor 591	2.19	0.0274
		2.04	0.0274
Gm9794	PREDICTED: predicted pseudogene 9794 (Gm9794), miscRNA.	-3.61	0.0274
Ccnyl1	cyclin Y-like 1	-2.54	0.0274
		-2.04	0.0275

Slco1a4	solute carrier organic anion transporter family, member 1a4; Synthetic construct Mus musculus clone IMAGE:100062527, MGC:190906 solute carrier organic anion transporter family, member 1a4 (Slco1a4) mRNA, encodes complete protein.	-3.15	0.0276
Gm25020	predicted gene, 25020 [Source:MGI Symbol;Acc:MGI:5454797]	-2.06	0.0276
		2.35	0.0277
Gm23215	predicted gene, 23215 [Source:MGI Symbol;Acc:MGI:5452992]	-16.01	0.0277
Gm23882	predicted gene, 23882 [Source:MGI Symbol;Acc:MGI:5453659]	-3.37	0.0277
Rpl21-ps13	ribosomal protein L21-ps13 [Source:MGI Symbol;Acc:MGI:3649022]	-2.08	0.0278
Rpl10a-ps1	ribosomal protein L10A, pseudogene 1	-4.83	0.0278
		-2.75	0.0279
		2.06	0.028
Ubqln2	ubiquilin 2	-2.29	0.0281
Gm28020	predicted gene, 28020 [Source:MGI Symbol;Acc:MGI:5531402]	-2.36	0.0281
		-2.43	0.0282
Gm14401	predicted gene 14401 [Source:MGI Symbol;Acc:MGI:3650075]	-2.9	0.0283
Gm10029	predicted gene 10029	-9.49	0.0283
Mir130c	microRNA 130c	-2.15	0.0284
15253; RP23-22	predicted gene 15253; protein phosphatase 1, regulatory (inhibitor) subunit 11 (Ppp1r11) pseudogene	-2.91	0.0284
Cdr1	cerebellar degeneration related antigen 1	-11.49	0.0285
Gm8112	predicted gene 8112	-2.3	0.0285
Gm8158	PREDICTED: predicted gene 8158 (Gm8158), mRNA.	-2.3	0.0285
Rbm39	RNA binding motif protein 39	-2.27	0.0285
Gm11687	predicted gene 11687 [Source:MGI Symbol;Acc:MGI:3652085]	-2.89	0.0285
		3.28	0.0286
		-2.28	0.0287
		-12.58	0.0288
Gm27459	predicted gene, 27459 [Source:MGI Symbol;Acc:MGI:5530841]	-2.81	0.0288
Rpl19-ps1	ribosomal protein L19, pseudogene 1	-3.18	0.0288
Gm3617	predicted gene 3617 [Source:MGI Symbol;Acc:MGI:3781793]	-3.25	0.0289
Gm9385	predicted pseudogene 9385	-2.36	0.0291

Gm7964	predicted gene 7964 [Source:MGI Symbol;Acc:MGI:3646150]	-2.85	0.0293
		2.03	0.0294
Gm22851	predicted gene, 22851 [Source:MGI Symbol;Acc:MGI:5452628]	-15.06	0.0295
Gm5777	predicted gene 5777 [Source:MGI Symbol;Acc:MGI:3645433]	-2.05	0.0295
Gm23935	predicted gene, 23935 [Source:MGI Symbol;Acc:MGI:5453712]	-34.19	0.0295
		-2.46	0.0295
		-2.94	0.0296
Gm23810	predicted gene, 23810 [Source:MGI Symbol;Acc:MGI:5453587]	-2.33	0.0296
Ppp1r2-ps4	protein phosphatase 1, regulatory (inhibitor) subunit 2, pseudogene 4	-2.11	0.0296
Dctn4	dynactin 4	-2.68	0.0297
Rpsa-ps10	ribosomal protein SA, pseudogene 10 [Source:MGI Symbol;Acc:MGI:3704228]	-5	0.0297
		-2.48	0.0297
Rpl15-ps3	ribosomal protein L15, pseudogene 3	-3.82	0.0297
Gm10155	predicted gene 10155 [Source:MGI Symbol;Acc:MGI:3642914]	-3.44	0.0298
Gm10260	predicted gene 10260 [Source:MGI Symbol;Acc:MGI:3642298]	-2.95	0.0298
Gm5762	predicted gene 5762 [Source:MGI Symbol;Acc:MGI:3647840]	-2.32	0.0298
Zmym4	zinc finger, MYM-type 4	-2.71	0.0298
Gm13469	predicted gene 13469 [Source:MGI Symbol;Acc:MGI:3649386]	-2.14	0.0298
Gm5773	predicted pseudogene 5773 [Source:MGI Symbol;Acc:MGI:3645792]	2.12	0.0298
Gm25042	predicted gene, 25042 [Source:MGI Symbol;Acc:MGI:5454819]	-42.02	0.0298
Gm14295	predicted gene 14295	-2.85	0.0298
Gm7665	predicted pseudogene 7665 [Source:MGI Symbol;Acc:MGI:3645651]	-2.13	0.0299
Rap1b	RAS related protein 1b	-3.67	0.0299
Gm26905	predicted gene, 26905 [Source:MGI Symbol;Acc:MGI:5477399]	-6.72	0.0299
Gm23578	predicted gene, 23578 [Source:MGI Symbol;Acc:MGI:5453355]	-2.47	0.03
Rps3a1	ribosomal protein S3A1	-4.74	0.03
		-6.75	0.03
		-5.65	0.03

Gm14296	predicted gene 14296	-2.12	0.03
Gm11011	predicted gene 11011 [Source:MGI Symbol;Acc:MGI:3779229]	2.01	0.03
Gm22631	predicted gene, 22631 [Source:MGI Symbol;Acc:MGI:5452408]	-15.19	0.0301
Gm25350	predicted gene, 25350 [Source:MGI Symbol;Acc:MGI:5455127]	-22.1	0.0301
Gm22128	predicted gene, 22128 [Source:MGI Symbol;Acc:MGI:5451905]	-22.1	0.0301
Gm26246	predicted gene, 26246 [Source:MGI Symbol;Acc:MGI:5456023]	-22.1	0.0301
Gm25597	predicted gene, 25597 [Source:MGI Symbol;Acc:MGI:5455374]	-22.1	0.0301
Gm26201	predicted gene, 26201 [Source:MGI Symbol;Acc:MGI:5455978]	-22.1	0.0301
Gm22812	predicted gene, 22812 [Source:MGI Symbol;Acc:MGI:5452589]	-22.1	0.0301
Gm26433	predicted gene, 26433 [Source:MGI Symbol;Acc:MGI:5456210]	-22.1	0.0301
Gm23357	predicted gene, 23357 [Source:MGI Symbol;Acc:MGI:5453134]	-22.1	0.0301
Gm22110	predicted gene, 22110 [Source:MGI Symbol;Acc:MGI:5451887]	-22.1	0.0301
Gm23724	predicted gene, 23724 [Source:MGI Symbol;Acc:MGI:5453501]	-22.1	0.0301
Gm23549	predicted gene, 23549 [Source:MGI Symbol;Acc:MGI:5453326]	-22.1	0.0301
Gm26223	predicted gene, 26223 [Source:MGI Symbol;Acc:MGI:5456000]	-22.1	0.0301
Gm23524	predicted gene, 23524 [Source:MGI Symbol;Acc:MGI:5453301]	-22.1	0.0301
Gm26136	predicted gene, 26136 [Source:MGI Symbol;Acc:MGI:5455913]	-22.1	0.0301
Gm24658	predicted gene, 24658 [Source:MGI Symbol;Acc:MGI:5454435]	-22.1	0.0301
Gm23359	predicted gene, 23359 [Source:MGI Symbol;Acc:MGI:5453136]	-22.1	0.0301
Gm24742	predicted gene, 24742 [Source:MGI Symbol;Acc:MGI:5454519]	-22.1	0.0301
Gm23696	predicted gene, 23696 [Source:MGI Symbol;Acc:MGI:5453473]	-22.1	0.0301
Gm26094	predicted gene, 26094 [Source:MGI Symbol;Acc:MGI:5455871]	-22.1	0.0301

Kcnd2	potassium voltage-gated channel, Shal-related family, member 2	-2.81	0.0302
Gm14263	predicted gene 14263 [Source:MGI Symbol;Acc:MGI:3649393]	-2.26	0.0303
Gm21954	predicted gene, 21954 [Source:MGI Symbol;Acc:MGI:5439423]	-2.71	0.0305
		-2.01	0.0306
		-3.68	0.0307
		3.77	0.0308
		-2.05	0.0309
Gm25074	predicted gene, 25074 [Source:MGI Symbol;Acc:MGI:5454851]	-10.27	0.031
Rpl21-ps4	ribosomal protein L21, pseudogene 4 [Source:MGI Symbol;Acc:MGI:3646825]	-2.34	0.031
		-2.06	0.0312
Gpm6b	glycoprotein m6b	-2.32	0.0312
Rpl30-ps11	ribosomal protein L30, pseudogene 11	-3.63	0.0313
Gm17150	predicted gene 17150 [Source:MGI Symbol;Acc:MGI:4937977]	-6.91	0.0314
		-2.42	0.0314
F20Rik; 261000	cadherin 11 pseudogene; RIKEN cDNA 6820431F20 gene	-3.14	0.0315
		2.4	0.0315
Gm24674	predicted gene, 24674 [Source:MGI Symbol;Acc:MGI:5454451]	2.31	0.0315
Zfp960	zinc finger protein 960	-3.39	0.0315
Rpl17-ps4	ribosomal protein L17, pseudogene 4	-3.03	0.0315
Gm23089	predicted gene, 23089 [Source:MGI Symbol;Acc:MGI:5452866]	-23.69	0.0316
Gm24711	predicted gene, 24711 [Source:MGI Symbol;Acc:MGI:5454488]	-23.69	0.0316
Gm26188	predicted gene, 26188 [Source:MGI Symbol;Acc:MGI:5455965]	-23.69	0.0316
Gm3160	predicted pseudogene 3160 [Source:MGI Symbol;Acc:MGI:3781339]	-2.26	0.0317
Gm25033	predicted gene, 25033 [Source:MGI Symbol;Acc:MGI:5454810]	-10.41	0.0317
Olfr1055	olfactory receptor 1055	-2.79	0.0317
Gm25839	predicted gene, 25839 [Source:MGI Symbol;Acc:MGI:5455616]	2.24	0.0317
Gm23876	predicted gene, 23876 [Source:MGI Symbol;Acc:MGI:5453653]	-7.12	0.0318
Rps6-ps4	ribosomal protein S6, pseudogene 4	-10.66	0.0318
		-3.48	0.032

Gm12058	predicted gene 12058 [Source:MGI Symbol;Acc:MGI:3652312]	-2.49	0.032
Gm6177	predicted gene 6177	-2.6	0.0322
Gm15497	predicted gene 15497 [Source:MGI Symbol;Acc:MGI:3782944]	-2.03	0.0324
		-2.24	0.0324
		-2.01	0.0324
		-2.61	0.0324
		-2.69	0.0325
Pde1b	phosphodiesterase 1B, Ca2+-calmodulin dependent; phosphodiesterase 1B, Ca2+-calmodulin dependent (Pde1b), mRNA.	-2.96	0.0325
Nap1l1	nucleosome assembly protein 1-like 1	-2.57	0.0325
Rpl30-ps2	ribosomal protein L30, pseudogene 2	-3.9	0.0325
		-2.66	0.0327
		2.24	0.0327
Gm26303	predicted gene, 26303 [Source:MGI Symbol;Acc:MGI:5456080]	-2.11	0.0327
Gm15198	predicted gene 15198 [Source:MGI Symbol;Acc:MGI:3705463]	-3.02	0.0328
Rpl21-ps8	ribosomal protein L21, pseudogene 8	-3.31	0.0329
		-6.66	0.033
Gm23716	predicted gene, 23716 [Source:MGI Symbol;Acc:MGI:5453493]	-2.23	0.033
Krtap4-7	keratin associated protein 4-7	2.2	0.033
l1; Snord116l2;	small nucleolar RNA, C/D box 116-like 1 (Snord116l1), small nucleolar RNA.; small nucleolar RNA, C/D box 116-like 2; small nucleolar RNA, C/D box 116 cluster	-24.74	0.0331
l1; Snord116l2;	small nucleolar RNA, C/D box 116-like 1 (Snord116l1), small nucleolar RNA.; small nucleolar RNA, C/D box 116-like 2 (Snord116l2), small nucleolar RNA.; small nucleolar RNA, C/D box 116 cluster	-24.74	0.0331
l1; Snord116l2;	small nucleolar RNA, C/D box 116-like 1 (Snord116l1), small nucleolar RNA.; small nucleolar RNA, C/D box 116-like 2 (Snord116l2), small nucleolar RNA.; small nucleolar RNA, C/D box 116 cluster	-24.74	0.0331
l1; Snord116l2;	small nucleolar RNA, C/D box 116-like 1 (Snord116l1), small nucleolar RNA.; small nucleolar RNA, C/D box 116-like 2 (Snord116l2), small nucleolar RNA.; small nucleolar RNA, C/D box 116 cluster	-24.74	0.0331

l1; Snord116l2;	small nucleolar RNA, C/D box 116-like 1 (Snord116l1), small nucleolar RNA.; small nucleolar RNA, C/D box 116-like 2 (Snord116l2), small nucleolar RNA.; small nucleolar RNA, C/D box 116 cluster	-24.74	0.0331
l1; Snord116l2;	small nucleolar RNA, C/D box 116-like 1 (Snord116l1), small nucleolar RNA.; small nucleolar RNA, C/D box 116-like 2 (Snord116l2), small nucleolar RNA.; small nucleolar RNA, C/D box 116 cluster	-24.74	0.0331
l1; Snord116l2;	small nucleolar RNA, C/D box 116-like 1 (Snord116l1), small nucleolar RNA.; small nucleolar RNA, C/D box 116-like 2 (Snord116l2), small nucleolar RNA.; small nucleolar RNA, C/D box 116 cluster	-24.74	0.0331
l1; Snord116l2;	small nucleolar RNA, C/D box 116-like 1; small nucleolar RNA, C/D box 116-like 2 (Snord116l2), small nucleolar RNA.; small nucleolar RNA, C/D box 116 cluster	-24.74	0.0331
l1; Snord116l2;	small nucleolar RNA, C/D box 116-like 1 (Snord116l1), small nucleolar RNA.; small nucleolar RNA, C/D box 116-like 2 (Snord116l2), small nucleolar RNA.; small nucleolar RNA, C/D box 116 cluster	-24.74	0.0331
Snord116l2	small nucleolar RNA, C/D box 116-like 2	-24.74	0.0331
Snord116l1	small nucleolar RNA, C/D box 116-like 1	-24.74	0.0331
Rpsa-ps2	ribosomal protein SA, pseudogene 2 [Source:MGI Symbol;Acc:MGI:3643356]	-2.99	0.0332
		-3.14	0.0334
Gm20077	PREDICTED: predicted gene, 20077 (Gm20077), miscRNA.	-7.21	0.0334
Mir466m	microRNA 466m [Source:MGI Symbol;Acc:MGI:4834277]	-2.06	0.0335
Gria1	glutamate receptor, ionotropic, AMPA1 (alpha 1)	-2.11	0.0335
		-2.85	0.0335
Gm16073	predicted gene 16073 [Source:MGI Symbol;Acc:MGI:3802177]	-2.06	0.0335
		-2.98	0.0335
m6984; Cbx3-p	predicted gene 6984; chromobox 3, pseudogene 6 [Source:MGI Symbol;Acc:MGI:3646710]	-2.38	0.0336
		-3.13	0.0336
Gm14648	predicted gene 14648 [Source:MGI Symbol;Acc:MGI:3705508]	-5.01	0.0336

Gm23970	predicted gene, 23970 [Source:MGI Symbol;Acc:MGI:5453747]	2.26	0.0336
Ndufa4	NADH dehydrogenase (ubiquinone) 1 alpha subcomplex, 4	-2.59	0.0337
		-8.81	0.0337
Rab26os	RAB26, member RAS oncogene family, opposite strand	-3.21	0.0338
Gm10335	predicted gene 10335	-4.4	0.0338
		-2.35	0.034
Gm14017	predicted gene 14017 [Source:MGI Symbol;Acc:MGI:3702155]	-2.23	0.0341
		-2.23	0.0341
		-3.27	0.0342
Gm11450	predicted gene 11450 [Source:MGI Symbol;Acc:MGI:3651345]	-6.72	0.0343
Gm4853	predicted pseudogene 4853	-11.83	0.0343
Nt5c2	5-nucleotidase, cytosolic II; 5'-nucleotidase, cytosolic II	-2.6	0.0343
		-2.13	0.0343
Gm26032	predicted gene, 26032 [Source:MGI Symbol;Acc:MGI:5455809]	-19.56	0.0344
Gm22046	predicted gene, 22046 [Source:MGI Symbol;Acc:MGI:5451823]	-19.56	0.0344
Gm25471	predicted gene, 25471 [Source:MGI Symbol;Acc:MGI:5455248]	-19.56	0.0344
Gm25210	predicted gene, 25210 [Source:MGI Symbol;Acc:MGI:5454987]	-19.56	0.0344
Gm10923	predicted gene 10923	-2.88	0.0344
Gm5879	predicted gene 5879	-2.89	0.0345
		-6.75	0.0346
Vsig2	V-set and immunoglobulin domain containing 2	-2.45	0.0346
		2.48	0.0348
Gm26412	predicted gene, 26412 [Source:MGI Symbol;Acc:MGI:5456189]	-2.87	0.0348
Gm11975	predicted gene 11975 [Source:MGI Symbol;Acc:MGI:3650057]	-2.32	0.0349
Phxr4	per-hexamer repeat gene 4	-2.47	0.0349
Gm24591	predicted gene, 24591 [Source:MGI Symbol;Acc:MGI:5454368]	-2.23	0.0349
Rps8-ps2	ribosomal protein S8, pseudogene 2	-3.13	0.035
Eif4a-ps4	eukaryotic translation initiation factor 4A, pseudogene 4	-3.29	0.035
Gm26502	predicted gene, 26502 [Source:MGI Symbol;Acc:MGI:5456279]	-10.62	0.0351

Gm23313	predicted gene, 23313 [Source:MGI Symbol;Acc:MGI:5453090]	-10.62	0.0351
Gm23446	predicted gene, 23446 [Source:MGI Symbol;Acc:MGI:5453223]	-10.62	0.0351
Gm10020	predicted pseudogene 10020	-5.78	0.0352
Gm22173	predicted gene, 22173 [Source:MGI Symbol;Acc:MGI:5451950]	-15.07	0.0352
Bin1	bridging integrator 1	-2.02	0.0354
Gm10576	predicted gene 10576	-4.65	0.0355
Rgs5	regulator of G-protein signaling 5	-2.33	0.0356
Gm23862	predicted gene, 23862 [Source:MGI Symbol;Acc:MGI:5453639]	-23.92	0.0357
Gm14335	predicted gene 14335 [Source:MGI Symbol;Acc:MGI:3801741]	-2.57	0.0357
Mir342	microRNA 342	2.37	0.0357
Mir342	microRNA 342	2.37	0.0357
Gm16284	predicted gene 16284 [Source:MGI Symbol;Acc:MGI:3826541]	-3	0.0358
Gm22992	predicted gene, 22992 [Source:MGI Symbol;Acc:MGI:5452769]	-22.61	0.0359
Gm24092	predicted gene, 24092 [Source:MGI Symbol;Acc:MGI:5453869]	-22.61	0.0359
Gm25123	predicted gene, 25123 [Source:MGI Symbol;Acc:MGI:5454900]	-22.61	0.0359
Gm22103	predicted gene, 22103 [Source:MGI Symbol;Acc:MGI:5451880]	-22.61	0.0359
Gm25892	predicted gene, 25892 [Source:MGI Symbol;Acc:MGI:5455669]	-22.61	0.0359
Gm23620	predicted gene, 23620 [Source:MGI Symbol;Acc:MGI:5453397]	-22.61	0.0359
Gm25353	predicted gene, 25353 [Source:MGI Symbol;Acc:MGI:5455130]	-22.61	0.0359
		-3.03	0.0361
		2.26	0.0362
		-4	0.0364
2010107E04Rik	RIKEN cDNA 2010107E04 gene	-4.35	0.0364
Gm25896	predicted gene, 25896 [Source:MGI Symbol;Acc:MGI:5455673]	-3.25	0.0364
Gnal	guanine nucleotide binding protein, alpha stimulating, olfactory type; guanine nucleotide binding protein, alpha stimulating, olfactory type (Gnal), transcript variant 2, mRNA.	-4	0.0366
Gm6548	eukaryotic translation elongation factor 1 alpha 1 pseudogene	-2.8	0.0366

Gm23050	predicted gene, 23050 [Source:MGI Symbol;Acc:MGI:5452827]	2.3	0.0366
Gm20091	predicted gene, 20091	-9.97	0.0367
Rps15	ribosomal protein S15	-3.05	0.0368
		-7.38	0.037
Mir5115	microRNA 5115 (Mir5115), microRNA.	-2.16	0.037
431F20Rik; Gm	RIKEN cDNA 6820431F20 gene, mRNA (cDNA clone MGC:67188 IMAGE:6825946), complete cds.; predicted gene, 21092; cadherin 11 pseudogene	-2.58	0.0373
Hist1h4h	histone cluster 1, H4h	-3.28	0.0373
Mir341	microRNA 341	-4.82	0.0374
		-4.06	0.0374
Gm12778	predicted gene 12778 [Source:MGI Symbol;Acc:MGI:3649899]	-5.08	0.0377
1640; RP23-35	predicted gene 11640 [Source:MGI Symbol;Acc:MGI:3649238]; novel transcript	-6.3	0.0377
Gm4202	predicted gene 4202 [Source:MGI Symbol;Acc:MGI:3782379]	-2.58	0.0379
Gm6563	predicted pseudogene 6563 [Source:MGI Symbol;Acc:MGI:3646907]	-2.52	0.038
Gm22990	predicted gene, 22990 [Source:MGI Symbol;Acc:MGI:5452767]	-2.58	0.038
		2.25	0.0381
Gm24598	predicted gene, 24598 [Source:MGI Symbol;Acc:MGI:5454375]	-5.83	0.0382
17-ps10; Gm10	ribosomal protein L17, pseudogene 10 [Source:MGI Symbol;Acc:MGI:3642871]; predicted pseudogene 10294	-3.02	0.0384
Gm23631	predicted gene, 23631 [Source:MGI Symbol;Acc:MGI:5453408]	-2.39	0.0385
Gm6576	predicted gene 6576 [Source:MGI Symbol;Acc:MGI:3646644]	-7.55	0.0387
		-2.02	0.0388
		2.12	0.0389
		-5.18	0.0389
		-6.67	0.0389
		-6.67	0.0389
ir684-1; Gm102	microRNA 684-1; predicted pseudogene 10241	-7.52	0.039
Mir7077	microRNA 7077	2.25	0.039
Gm5070	predicted gene 5070 [Source:MGI Symbol;Acc:MGI:3643219]	-3.43	0.0391
Gm19731	PREDICTED: predicted gene, 19731 (Gm19731), miscRNA.	-2.73	0.0391

Gm25474	predicted gene, 25474 [Source:MGI Symbol;Acc:MGI:5455251]	-3.72	0.0393
Gm22047	predicted gene, 22047 [Source:MGI Symbol;Acc:MGI:5451824]	-3.72	0.0393
Gm23619	predicted gene, 23619 [Source:MGI Symbol;Acc:MGI:5453396]	-3.72	0.0393
		-3.08	0.0393
Mir466f-1	microRNA 466f-1	-2.32	0.0394
		-19.29	0.0395
Gm24683	predicted gene, 24683 [Source:MGI Symbol;Acc:MGI:5454460]	-2.21	0.0396
811; 2610005L	predicted gene, 21811; cadherin 11 pseudogene	-3.4	0.0397
Psip1	PC4 and SFRS1 interacting protein 1	-2.05	0.0398
Gm23051	predicted gene, 23051	-2.83	0.0399
Erc2	ELKS/RAB6-interacting/CAST family member 2	-2.69	0.0401
Gm19743	PREDICTED: predicted gene, 19743 (Gm19743), miscRNA.	-12.85	0.0401
		-2.95	0.0401
Gm23767	predicted gene, 23767 [Source:MGI Symbol;Acc:MGI:5453544]	-12.34	0.0401
Gm6747	PREDICTED: predicted gene 6747, transcript variant 2 (Gm6747), mRNA.	-2.87	0.0401
		-2.27	0.0402
		-10.67	0.0402
		-3.25	0.0404
Gm7327	predicted gene 7327	-3.17	0.0406
Rpl35a-ps4	ribosomal protein 35A, pseudogene 4	-8.81	0.0407
Rpl35a-ps6	ribosomal protein L35A, pseudogene 6	-8.81	0.0407
Rpl35a-ps3	ribosomal protein L35A, pseudogene 3	-8.81	0.0407
Ddx26b	DEAD/H (Asp-Glu-Ala-Asp/His) box polypeptide 26B	-2.11	0.0407
Cnot6	CCR4-NOT transcription complex, subunit 6	-2.52	0.0408
Rps2-ps5	ribosomal protein S2, pseudogene 5	-2.71	0.0408
Gpm6a	glycoprotein m6a	-5.05	0.0409
		-5.86	0.0409
Gm15393	predicted gene 15393 [Source:MGI Symbol;Acc:MGI:3705387]	-2.51	0.041
Uqcrh	ubiquinol-cytochrome c reductase hinge protein	-2.49	0.041
Gm7226	PREDICTED: predicted gene 7226 (Gm7226), mRNA.	-3.86	0.0412
Gm6483	predicted gene 6483 [Source:MGI Symbol;Acc:MGI:3644574]	-2.07	0.0412
Gm25063	predicted gene, 25063 [Source:MGI Symbol;Acc:MGI:5454840]	-3.59	0.0413

		-2.49	0.0415
Gm4184	PREDICTED: predicted gene 4184 (Gm4184), mRNA.	-6.76	0.0415
Gm8649	predicted gene 8649 [Source:MGI Symbol;Acc:MGI:3644257]	-5.7	0.0416
		-3.34	0.0416
Mir467c	microRNA 467c	-6.23	0.0419
Gm25510	predicted gene, 25510 [Source:MGI Symbol;Acc:MGI:5455287]	-2.8	0.0421
Gm22131	predicted gene, 22131 [Source:MGI Symbol;Acc:MGI:5451908]	-14.64	0.0422
Gm3531	predicted pseudogene 3531 [Source:MGI Symbol;Acc:MGI:3781708]	-3.98	0.0422
		2.2	0.0423
Xist	inactive X specific transcripts	-2.58	0.0424
Srsf6	serine/arginine-rich splicing factor 6	-2.35	0.0425
		-5.53	0.0426
Gm26097	predicted gene, 26097 [Source:MGI Symbol;Acc:MGI:5455874]	-6.88	0.0426
Traj14	T cell receptor alpha joining 14 [Source:MGI Symbol;Acc:MGI:4439581]	3.55	0.0427
Ntsr2	neurotensin receptor 2	-2.28	0.0429
		-11.69	0.043
Gm25513	predicted gene, 25513 [Source:MGI Symbol;Acc:MGI:5455290]	2.08	0.043
		2.32	0.043
Gm10051	predicted pseudogene 10051	-2.33	0.0432
Gm15776	predicted gene 15776	-2.19	0.0433
Crebbp	CREB binding protein; Synthetic construct Mus musculus clone IMAGE:100069545, MGC:199447 CREB binding protein (Crebbp) mRNA, encodes complete protein.	-2.04	0.0433
Gm5621	predicted gene 5621	-6.8	0.0434
Gm3362	predicted pseudogene 3362	-8.79	0.0435
		-2.37	0.0435
Gm23943	predicted gene, 23943 [Source:MGI Symbol;Acc:MGI:5453720]	-22.52	0.0435
Gm24811	predicted gene, 24811 [Source:MGI Symbol;Acc:MGI:5454588]	-3.79	0.0435
Dnajb9	DnaJ (Hsp40) homolog, subfamily B, member 9	-3.05	0.0435
Gm14323	predicted gene 14323 [Source:MGI Symbol;Acc:MGI:3650742]	-2.8	0.0435
Gm14303	predicted gene 14303	-4.94	0.0436
Rpl36-ps2	ribosomal protein L36, pseudogene 2	-2.73	0.0437

		2.05	0.0439
		-3.56	0.0439
		-2.98	0.0441
Gm6472	predicted pseudogene 6472 [Source:MGI Symbol;Acc:MGI:3648396]	-2.28	0.0441
Gm10480	predicted gene 10480 [Source:MGI Symbol;Acc:MGI:3642435]; predicted gene 10480	-2.6	0.0443
Sar1b	SAR1 gene homolog B (S. cerevisiae)	-3.17	0.0444
Acot13	acyl-CoA thioesterase 13	-2.23	0.0445
Eef2	eukaryotic translation elongation factor 2	-2.35	0.0445
		-2.71	0.0447
		-2.22	0.0447
Gm23153	predicted gene, 23153	-2.27	0.0448
Gm25000	predicted gene, 25000 [Source:MGI Symbol;Acc:MGI:5454777]	-4.66	0.0448
Gm25361	predicted gene, 25361 [Source:MGI Symbol;Acc:MGI:5455138]	-2.06	0.0448
Gm22862	predicted gene, 22862 [Source:MGI Symbol;Acc:MGI:5452639]	-5.94	0.0449
Gm22468	predicted gene, 22468 [Source:MGI Symbol;Acc:MGI:5452245]	-51.03	0.0449
Rps12-ps11	ribosomal protein S12, pseudogene 11	-3.21	0.0451
Rps12-ps15	ribosomal protein S12, pseudogene 15	-3.21	0.0451
LOC100862223	PREDICTED: 40S ribosomal protein S12-like (LOC100862223), mRNA.	-3.21	0.0451
Rps12-ps13	ribosomal protein S12, pseudogene 13	-3.21	0.0451
Gm14287	predicted gene 14287 [Source:MGI Symbol;Acc:MGI:3650189]	-2.12	0.0451
Gm23086	predicted gene, 23086 [Source:MGI Symbol;Acc:MGI:5452863]	-2.21	0.0451
Rpl14-ps1	ribosomal protein L14, pseudogene 1	-4.27	0.0456
Stxbp3-ps	syntaxin-binding protein 3, pseudogene	-2.38	0.0456
Ntrk2	neurotrophic tyrosine kinase, receptor, type 2; neurotrophic tyrosine kinase, receptor, type 2 (Ntrk2), transcript variant 1, mRNA.	-2.18	0.0456
Rps12-ps6	ribosomal protein S12, pseudogene 6	-3.06	0.0458
Rps12-ps18	ribosomal protein S12, pseudogene 18	-3.06	0.0458
Rps12-ps17	ribosomal protein S12, pseudogene 17	-3.06	0.0458
		-2.41	0.0458
Crh	corticotropin releasing hormone	-2.35	0.0458
		-2.3	0.0459
Rpl19-ps11	ribosomal protein L19, pseudogene 11	-3.86	0.0459
Gm11971	predicted gene 11971 [Source:MGI Symbol;Acc:MGI:3650054]	-3.41	0.046

Gm27626	predicted gene, 27626 [Source:MGI Symbol;Acc:MGI:5531008]	-4.85	0.046
		-3	0.0461
Tbc1d19	TBC1 domain family, member 19	-2.03	0.0461
		-5.35	0.0461
Gm25003	predicted gene, 25003	-2.21	0.0461
Gm24366	predicted gene, 24366 [Source:MGI Symbol;Acc:MGI:5454143]	-3.45	0.0463
Gm14279	predicted gene 14279	-3.57	0.0465
Gm22776	predicted gene, 22776 [Source:MGI Symbol;Acc:MGI:5452553]	-10.97	0.0467
		2.2	0.0468
LOC100861862	PREDICTED: uncharacterized LOC100861862 (LOC100861862), miscRNA.	-3.37	0.0469
		-2.14	0.0469
Gm14494	predicted gene 14494	-2.35	0.047
Gm23550	predicted gene, 23550 [Source:MGI Symbol;Acc:MGI:5453327]	-21.73	0.047
Gm10268	predicted gene 10268 [Source:MGI Symbol;Acc:MGI:3642637]	-4.24	0.0471
Gm23792	predicted gene, 23792 [Source:MGI Symbol;Acc:MGI:5453569]	2.05	0.0472
		-2.72	0.0472
Rps2-ps10	ribosomal protein S2, pseudogene 10 [Source:MGI Symbol;Acc:MGI:3645604]	-5.53	0.0472
Slc1a2	solute carrier family 1 (glial high affinity glutamate transporter), member 2	-2.01	0.0474
Gm11060	predicted gene 11060 [Source:MGI Symbol;Acc:MGI:3779284]	-3.87	0.0474
		10.12	0.0475
Gm16199	predicted gene 16199 [Source:MGI Symbol;Acc:MGI:3801797]	-5.82	0.0475
Rpl7a-ps11	ribosomal protein L7A, pseudogene 11	-2	0.0476
		-4.72	0.0476
Gm10689	predicted gene 10689 [Source:MGI Symbol;Acc:MGI:3642613]	-3.22	0.0476
Gm9864	predicted gene 9864 [Source:MGI Symbol;Acc:MGI:3708663]	-4.05	0.0477
ir1957a; Mir19:	microRNA 1957a [Source:MGI Symbol;Acc:MGI:3837121]; microRNA 1957	-4.73	0.0479
Rps2-ps6	ribosomal protein S2, pseudogene 6 [Source:MGI Symbol;Acc:MGI:3644876]	-6.92	0.0479
Gm5963	predicted pseudogene 5963	-7.83	0.0479

Gm11942	predicted gene 11942 [Source:MGI Symbol;Acc:MGI:3650608]	-3.4	0.0479
Luc7l3	LUC7-like 3 (S. cerevisiae)	-2.31	0.048
Gm13392	predicted gene 13392	-3.16	0.0481
Gm25754	predicted gene, 25754 [Source:MGI Symbol;Acc:MGI:5455531]	-43.39	0.0483
Gm24802	predicted gene, 24802 [Source:MGI Symbol;Acc:MGI:5454579]	-43.39	0.0483
Gm22419	predicted gene, 22419 [Source:MGI Symbol;Acc:MGI:5452196]	-43.39	0.0483
Gm22112	predicted gene, 22112 [Source:MGI Symbol;Acc:MGI:5451889]	-43.39	0.0483
Gm24744	predicted gene, 24744 [Source:MGI Symbol;Acc:MGI:5454521]	-43.39	0.0483
Gm26137	predicted gene, 26137 [Source:MGI Symbol;Acc:MGI:5455914]	-43.39	0.0483
Gm22029	predicted gene, 22029 [Source:MGI Symbol;Acc:MGI:5451806]	-43.39	0.0483
Gm25347	predicted gene, 25347 [Source:MGI Symbol;Acc:MGI:5455124]	-43.39	0.0483
Gm24567	predicted gene, 24567 [Source:MGI Symbol;Acc:MGI:5454344]	-43.39	0.0483
Gm22991	predicted gene, 22991 [Source:MGI Symbol;Acc:MGI:5452768]	-43.39	0.0483
Gm23304	predicted gene, 23304 [Source:MGI Symbol;Acc:MGI:5453081]	-43.39	0.0483
Gm25774	predicted gene, 25774 [Source:MGI Symbol;Acc:MGI:5455551]	-43.39	0.0483
Gm25778	predicted gene, 25778 [Source:MGI Symbol;Acc:MGI:5455555]	-43.39	0.0483
Gm26248	predicted gene, 26248 [Source:MGI Symbol;Acc:MGI:5456025]	-43.39	0.0483
Gm23844	predicted gene, 23844 [Source:MGI Symbol;Acc:MGI:5453621]	-43.39	0.0483
Gm23177	predicted gene, 23177 [Source:MGI Symbol;Acc:MGI:5452954]	-43.39	0.0483
Gm26190	predicted gene, 26190 [Source:MGI Symbol;Acc:MGI:5455967]	-43.39	0.0483
Gm22102	predicted gene, 22102 [Source:MGI Symbol;Acc:MGI:5451879]	-43.39	0.0483
Gm24193	predicted gene, 24193 [Source:MGI Symbol;Acc:MGI:5453970]	-43.39	0.0483
Gm25208	predicted gene, 25208 [Source:MGI Symbol;Acc:MGI:5454985]	-43.39	0.0483

Gm25228	predicted gene, 25228 [Source:MGI Symbol;Acc:MGI:5455005]	-43.39	0.0483
Gm24427	predicted gene, 24427 [Source:MGI Symbol;Acc:MGI:5454204]	-43.39	0.0483
Gm22346	predicted gene, 22346 [Source:MGI Symbol;Acc:MGI:5452123]	-43.39	0.0483
Gm23606	predicted gene, 23606 [Source:MGI Symbol;Acc:MGI:5453383]	-43.39	0.0483
Gm24617	predicted gene, 24617 [Source:MGI Symbol;Acc:MGI:5454394]	-43.39	0.0483
Gm24484	predicted gene, 24484 [Source:MGI Symbol;Acc:MGI:5454261]	-43.39	0.0483
Gm24535	predicted gene, 24535 [Source:MGI Symbol;Acc:MGI:5454312]	-4.1	0.0483
		-2.11	0.0483
Gm10115	predicted gene 10115	-3.46	0.0485
		-18.31	0.0485
Gm5786	predicted pseudogene 5786 [Source:MGI Symbol;Acc:MGI:3645003]	-8.62	0.0486
Dnajc15	DnaJ (Hsp40) homolog, subfamily C, member 15	-2.01	0.0487
Gm15013	predicted gene 15013 [Source:MGI Symbol;Acc:MGI:3644991]	-2.11	0.0487
Gm25544	predicted gene, 25544 [Source:MGI Symbol;Acc:MGI:5455321]	-3.85	0.0488
5243; RP24-484	predicted gene 16243 [Source:MGI Symbol;Acc:MGI:3801905]; novel transcript	-6.08	0.0489
Gm19494	PREDICTED: predicted gene, 19494, transcript variant 1 (Gm19494), miscRNA.; PREDICTED: predicted gene, 19494, transcript variant 2 (Gm19494), miscRNA.; PREDICTED: predicted gene, 19494, transcript variant 3 (Gm19494), miscRNA.	-2.21	0.0489
Gm9762	predicted pseudogene 9762 [Source:MGI Symbol;Acc:MGI:3704220]	-2.82	0.0491
Ctsb	cathepsin B	-2.09	0.0491
Gm10039	predicted pseudogene 10039	-3.34	0.0493
Gm23193	predicted gene, 23193 [Source:MGI Symbol;Acc:MGI:5452970]	-2.32	0.0494
Fabp7	fatty acid binding protein 7, brain	-2.47	0.0495
Apold1	apolipoprotein L domain containing 1	-2.2	0.0495
		4.92	0.0495
		4.92	0.0495
Gm15796	predicted gene 15796 [Source:MGI Symbol;Acc:MGI:3801844]	-4.92	0.0496

Mir297c	microRNA 297c [Source:MGI Symbol;Acc:MGI:3718506]	-2.14	0.0497
Abcb1a	ATP-binding cassette, sub-family B (MDR/TAP), member 1A	-2.23	0.0499

Table S7. Differentially expressed genes for the high glycemic diet (HGD) with soluble epoxide hydrolase inhibitor (sEHI) compared to without sEHI.

Gene Symbol	Description	Fold Change	P-value
Gm25617	predicted gene, 25617 [Source:MGI Symbol;Acc:MGI:5455394]	2.85	3.55E-05
		2.01	4.34E-05
Gm24373	predicted gene, 24373 [Source:MGI Symbol;Acc:MGI:5454150]	2	0.0001
Gm23253	predicted gene, 23253 [Source:MGI Symbol;Acc:MGI:5453030]	-2.48	0.0002
Ighv5-15	immunoglobulin heavy variable 5-15	2.4	0.0002
		2.04	0.0003
		2	0.0004
		-23.72	0.0006
		2.23	0.0007
Gm12571	predicted gene 12571 [Source:MGI Symbol;Acc:MGI:3649675]	-2.28	0.0008
		-2	0.001
		-2.31	0.0011
Gm27030; RP24-365B9.1	predicted gene, 27030 [Source:MGI Symbol;Acc:MGI:5504145]; novel transcript	2.78	0.0013
		-3.37	0.0015
		-2.18	0.0018
		-3.2	0.0021
Mir697	microRNA 697	2.09	0.0022
		2.87	0.0022
Gm10356	predicted gene 10356 [Source:MGI Symbol;Acc:MGI:3641761]	-2.13	0.0024
		2.08	0.0026
Gm12238	predicted gene 12238	2.38	0.0027
Pcna-ps2	proliferating cell nuclear antigen pseudogene 2 [Source:MGI Symbol;Acc:MGI:97505]	-3.43	0.0027
Ighv1-63	immunoglobulin heavy variable V1-63	2.84	0.0027
Gm22519	predicted gene, 22519 [Source:MGI Symbol;Acc:MGI:5452296]	2.36	0.0028
		2.24	0.0028
Gm23134	predicted gene, 23134 [Source:MGI Symbol;Acc:MGI:5452911]	-10.99	0.0028
AF357426	snoRNA AF357426; snoRNA AF357426 (AF357426), small nucleolar RNA.	-9.37	0.0029
Gm12696	predicted gene 12696 [Source:MGI Symbol;Acc:MGI:3649288]	-2.16	0.003
Gm11295	predicted gene 11295 [Source:MGI Symbol;Acc:MGI:3651595]	3.31	0.0032
Gm23159	predicted gene, 23159 [Source:MGI Symbol;Acc:MGI:5452936]	4.43	0.0035

Gm24791	predicted gene, 24791 [Source:MGI Symbol;Acc:MGI:5454568]	2.22	0.0035
		-2.59	0.0037
		-2.93	0.004
		4.63	0.0042
Gm23042	predicted gene, 23042 [Source:MGI Symbol;Acc:MGI:5452819]	2.27	0.0043
		-2.55	0.0043
		2.17	0.0045
Gm20678	predicted gene 20678 [Source:MGI Symbol;Acc:MGI:5313125]	2.72	0.0045
		-2.36	0.0045
		19.33	0.0046
		-2.62	0.0058
Gm19595	PREDICTED: predicted gene, 19595 (Gm19595), miscRNA.	-9.84	0.0063
Snord82	small nucleolar RNA, C/D box 82	3.03	0.0068
Hist2h2aa1; Hist2h2aa2	histone cluster 2, H2aa1 (Hist2h2aa1), mRNA.; histone cluster 2, H2aa2; histone cluster 2, H2aa1, mRNA (cDNA clone MGC:5956 IMAGE:3582122), complete cds.; histone cluster 2, H2aa1, mRNA (cDNA clone MGC:73680 IMAGE:1448126), complete cds.; histone cluster 2, H2aa1, mRNA (cDNA clone MGC:107211 IMAGE:6771160), complete cds.; histone cluster 2, H2aa1, mRNA (cDNA clone MGC:182884 IMAGE:9087498), complete cds.; histone cluster 2, H2aa1, mRNA (cDNA clone MGC:182887 IMAGE:9087501), complete cds.; Synthetic construct Mus musculus clone IMAGE:100064049, MGC:193420 histone cluster 2, H2aa1 (Hist2h2aa1) mRNA, encodes complete protein.; histone cluster 2, H2aa1	-2.65	0.0069
Ighv1-50	immunoglobulin heavy variable 1-50	2.28	0.007
Gm24001	predicted gene, 24001 [Source:MGI Symbol;Acc:MGI:5453778]	-2.19	0.0074
Actr1a	ARP1 actin-related protein 1A, centractin alpha	2.63	0.0075
		-2.59	0.0077
		2.18	0.0077
Gm19609	PREDICTED: predicted gene, 19609 (Gm19609), miscRNA.	2.11	0.0085
Gm22966	predicted gene, 22966 [Source:MGI Symbol;Acc:MGI:5452743]	2.1	0.0089
Asphd2	aspartate beta-hydroxylase domain containing 2	-2.81	0.0089
		4.66	0.0089
Gm12396	predicted gene 12396	-4.34	0.0092
Gm11969	predicted gene 11969 [Source:MGI Symbol;Acc:MGI:3650290]	-5.07	0.0093

LOC100862384	PREDICTED: uncharacterized LOC100862384 (LOC100862384), miscRNA.	-2.65	0.0093
Psenen	presenilin enhancer 2 homolog (C. elegans)	-3.26	0.0094
Gm25525	predicted gene, 25525 [Source:MGI Symbol;Acc:MGI:5455302]	2.59	0.0095
Gm10222	predicted gene 10222 [Source:MGI Symbol;Acc:MGI:3642643]	26.75	0.0095
		-2.98	0.0099
Traj54	T cell receptor alpha joining 54 [Source:MGI Symbol;Acc:MGI:4937113]	2.25	0.01
Cldn34c4	claudin 34C4	2.27	0.0101
mt-Tr	mitochondrially encoded tRNA arginine [Source:MGI Symbol;Acc:MGI:102476]	46.18	0.0101
Gm23905	predicted gene, 23905 [Source:MGI Symbol;Acc:MGI:5453682]	-2.02	0.0103
Max	Max protein	-3.32	0.0106
Gm6542	predicted gene 6542 [Source:MGI Symbol;Acc:MGI:3644649]	2.23	0.0107
		-2.04	0.0108
Gm7125	predicted pseudogene 7125 [Source:MGI Symbol;Acc:MGI:3645829]	-2.43	0.0112
Gm23600	predicted gene, 23600 [Source:MGI Symbol;Acc:MGI:5453377]	982.21	0.0114
		2.21	0.0114
		2.12	0.0115
		-2.04	0.0117
		-2.16	0.0119
Gm22044	predicted gene, 22044 [Source:MGI Symbol;Acc:MGI:5451821]	2.28	0.0121
Ina	internexin neuronal intermediate filament protein, alpha	2.49	0.013
Gm14769	predicted gene 14769 [Source:MGI Symbol;Acc:MGI:3708088]	3.24	0.0132
		2.07	0.0134
		-3.92	0.0146
mt-Tq	mitochondrially encoded tRNA glutamine [Source:MGI Symbol;Acc:MGI:102477]	12.43	0.0147
		-2.34	0.015
Mir6917	microRNA 6917	2.05	0.0152
		2.35	0.0154
Gm25357	predicted gene, 25357 [Source:MGI Symbol;Acc:MGI:5455134]	29.89	0.0162
		-3.21	0.0166
Traj14	T cell receptor alpha joining 14 [Source:MGI Symbol;Acc:MGI:4439581]	16.56	0.017
Gm12338	cytochrome c oxidase, subunit VIIc pseudogene	-2.25	0.0171
Gm6750	PREDICTED: predicted gene 6750 (Gm6750), mRNA.	-2.22	0.0173

		-2.32	0.0174
Psmb7	proteasome (prosome, macropain) subunit, beta type 7	-2.24	0.0175
		2.02	0.0176
Mir875	microRNA 875	2.26	0.0176
AY036118	cDNA sequence AY036118	-2.22	0.0178
Gm13326	predicted gene 13326 [Source:MGI Symbol;Acc:MGI:3650196]	2.43	0.0179
Gm24187	predicted gene, 24187 [Source:MGI Symbol;Acc:MGI:5453964]	-132.49	0.0182
		2.68	0.0185
Gm13341	predicted gene 13341 [Source:MGI Symbol;Acc:MGI:3650228]	4.52	0.0187
Gm14472	predicted gene 14472 [Source:MGI Symbol;Acc:MGI:3650138]	-2.24	0.0189
Gm23785	predicted gene, 23785 [Source:MGI Symbol;Acc:MGI:5453562]	-2.77	0.0192
Gm24988	predicted gene, 24988 [Source:MGI Symbol;Acc:MGI:5454765]	2.11	0.0194
Gm24539	predicted gene, 24539 [Source:MGI Symbol;Acc:MGI:5454316]	-2.89	0.0195
Gm24245	predicted gene, 24245 [Source:MGI Symbol;Acc:MGI:5454022]	-81.17	0.0196
Gm24270	predicted gene, 24270 [Source:MGI Symbol;Acc:MGI:5454047]	-81.17	0.0196
Txnip	thioredoxin interacting protein	-3.27	0.0196
		2.05	0.0196
Rorb	RAR-related orphan receptor beta	2.19	0.0199
		2.36	0.0202
Gm3788	predicted gene 3788 [Source:MGI Symbol;Acc:MGI:3781961]	2.45	0.0203
Ighv7-1	immunoglobulin heavy variable 7-1 [Source:MGI Symbol;Acc:MGI:4439622]	2.35	0.0203
Gm22974	predicted gene, 22974 [Source:MGI Symbol;Acc:MGI:5452751]	2.63	0.0205
		-2.45	0.0209
		-6.05	0.0209
Gm19974	PREDICTED: predicted gene, 19974 (Gm19974), miscRNA.	-2.12	0.0222
Mcam	melanoma cell adhesion molecule	-2.92	0.0225
		2.69	0.0225
		-2.23	0.0229
Gm26704	predicted gene, 26704 [Source:MGI Symbol;Acc:MGI:5477198]	2.09	0.0232
		2.01	0.0232
		2.33	0.0233

mt-Tg	mitochondrially encoded tRNA glycine [Source:MGI Symbol;Acc:MGI:102486]	71.98	0.0238
		2.97	0.0244
		2.1	0.025
		2.17	0.025
mt-Tp	mitochondrially encoded tRNA proline [Source:MGI Symbol;Acc:MGI:102478]	2.62	0.0252
Dstn	destrin	-2.14	0.0253
		-2.14	0.0254
LOC100861675	PREDICTED: uncharacterized LOC100861675 (LOC100861675), miscRNA.	-2.16	0.0258
Mir6994	microRNA 6994	3.53	0.0263
		2.3	0.0265
Gm8062	predicted pseudogene 8062 [Source:MGI Symbol;Acc:MGI:3643433]	-2.83	0.0266
		2.04	0.0266
Mir1950	microRNA 1950	2.09	0.0266
Mir125b-2	microRNA 125b-2	2.31	0.0266
Gm14719	predicted gene 14719 [Source:MGI Symbol;Acc:MGI:3705771]	-2.04	0.0267
Gm19491	PREDICTED: predicted gene, 19491 (Gm19491), miscRNA.	-2.4	0.0272
Gm11866	predicted gene 11866 [Source:MGI Symbol;Acc:MGI:3650240]	2.06	0.0273
Gm22266	predicted gene, 22266 [Source:MGI Symbol;Acc:MGI:5452043]	2.02	0.0275
		-2.29	0.0278
		2.64	0.0284
Gm13162	predicted pseudogene 13162 [Source:MGI Symbol;Acc:MGI:3701119]	3.09	0.0287
Spa17	sperm autoantigenic protein 17	2.04	0.0287
		-3.01	0.0288
Gm25189	predicted gene, 25189 [Source:MGI Symbol;Acc:MGI:5454966]	2.58	0.0292
Gm24095	predicted gene, 24095 [Source:MGI Symbol;Acc:MGI:5453872]	-3.34	0.0296
mt-Tn	mitochondrially encoded tRNA asparagine [Source:MGI Symbol;Acc:MGI:102479]	13.18	0.0296
Gm26397	predicted gene, 26397 [Source:MGI Symbol;Acc:MGI:5456174]	2.94	0.0303
Gm25911	predicted gene, 25911 [Source:MGI Symbol;Acc:MGI:5455688]	-20.91	0.0305
		2.29	0.0305
		4.87	0.0313
		-2.02	0.0314

Gm14284	predicted gene 14284 [Source:MGI Symbol;Acc:MGI:3650419]	-2.43	0.0315
		-2.58	0.0321
2010007H06Rik	RIKEN cDNA 2010007H06 gene [Source:MGI Symbol;Acc:MGI:1917099]	-2.18	0.0326
Gm5265	predicted pseudogene 5265 [Source:MGI Symbol;Acc:MGI:3643416]	-2.2	0.0327
Stom	stomatin	-2.24	0.0328
Gm23442	predicted gene, 23442 [Source:MGI Symbol;Acc:MGI:5453219]	2.75	0.0331
Mir1955	microRNA 1955	-4.79	0.0333
Snord14e	small nucleolar RNA, C/D box 14E	6.48	0.0333
		-2.25	0.034
		-2.67	0.034
Gm5436	predicted pseudogene 5436 [Source:MGI Symbol;Acc:MGI:3643291]	2.36	0.0346
Gm22747	predicted gene, 22747 [Source:MGI Symbol;Acc:MGI:5452524]	2.66	0.0346
		2.16	0.0347
Gm16111	predicted gene 16111 [Source:MGI Symbol;Acc:MGI:3801919]	-2.97	0.0348
		2	0.0349
		2	0.0349
		2	0.0349
Olfr947-ps1	olfactory receptor 947, pseudogene 1	2.02	0.035
Gm6900	predicted gene 6900 [Source:MGI Symbol;Acc:MGI:3645052]	-2.31	0.0352
Gm23610	predicted gene, 23610 [Source:MGI Symbol;Acc:MGI:5453387]	4.71	0.0354
Gm22259	predicted gene, 22259 [Source:MGI Symbol;Acc:MGI:5452036]	2.14	0.0355
		-6.16	0.0361
Gm26087	predicted gene, 26087 [Source:MGI Symbol;Acc:MGI:5455864]	2	0.0362
Gm22933	predicted gene, 22933 [Source:MGI Symbol;Acc:MGI:5452710]	3.58	0.0364
		3.2	0.0365
Zfp935	zinc finger protein 935	2.55	0.0372
Gm24103	predicted gene, 24103 [Source:MGI Symbol;Acc:MGI:5453880]	2.74	0.0388
		2.25	0.039
		2.04	0.0392
Gm23963	predicted gene, 23963 [Source:MGI Symbol;Acc:MGI:5453740]	4.42	0.0395
		-8.93	0.0406
		-8.93	0.0406

Pias1	protein inhibitor of activated STAT 1	-2.21	0.0418
Vdac3	voltage-dependent anion channel 3	-3.19	0.0435
		2.42	0.0436
		4.13	0.0436
Gm11440	predicted gene 11440 [Source:MGI Symbol;Acc:MGI:3649803]	-2.41	0.0442
		5.07	0.0442
Gm8730	predicted pseudogene 8730 [Source:MGI Symbol;Acc:MGI:3644565]	-2.52	0.0452
Mir1942	microRNA 1942	2.43	0.0456
Gm24666	predicted gene, 24666 [Source:MGI Symbol;Acc:MGI:5454443]	-2.29	0.046
Mir466f-1	microRNA 466f-1	-2.1	0.0461
Gm23105	predicted gene, 23105 [Source:MGI Symbol;Acc:MGI:5452882]	4.31	0.0468
Rlf	rearranged L-myc fusion sequence	-3.27	0.047
		2.23	0.0472
Ranbp9	RAN binding protein 9	2.21	0.0477
ND3	NADH dehydrogenase subunit 3	3.67	0.0478
LOC100861967	PREDICTED: uncharacterized LOC100861967, transcript variant 1 (LOC100861967), miscRNA.; PREDICTED: uncharacterized LOC100861967, transcript variant 2 (LOC100861967), miscRNA.; PREDICTED: uncharacterized LOC100861967, transcript variant 3 (LOC100861967), miscRNA.	-2.12	0.0478
		3.06	0.0483
Sgtb	small glutamine-rich tetratricopeptide repeat (TPR)-containing, beta	-2.08	0.0495
Gm26203	predicted gene, 26203 [Source:MGI Symbol;Acc:MGI:5455980]	2.04	0.0497

Table S8. Effect of the low glycemic diet (LGD) with and without soluble epoxide hydrolase inhibitor (sEHI) on the expression of microRNAs (miRNAs) in hippocampal microvessels.

Gene Symbol	Fold Change	P-value
Mir6338	2.02	0.0002
Mir669d	-4.16	0.0002
Mir466d	-3.51	0.0003
Mir669l	-2.34	0.0003
Mir7223	2.5	0.0004
Mir667	-2.8	0.0005
Mir494	-3.06	0.0018
Mir669b	-4.79	0.0021
Mir692-2	-2.18	0.0023
Mir669f	-2.02	0.0028
Mir6239	-2.02	0.003
Mir684-2	-451.28	0.0032
Mir466j	-8.07	0.0033
Mir466o	-33.08	0.0046
Mir466k	-8.33	0.005
Mir684-1	-482.47	0.0053
Mir297-1	-16.66	0.0054
Mir7681	2.06	0.0056
Mir684-1	-471.83	0.0068
Mir466f-4	-7.32	0.0073
Mir466f-3	-16.33	0.0074
Mir6380	-2.04	0.0096
Mir297a-2	-26.18	0.0109
Mir297a-3	-2.4	0.0114
Mir669m-1	-2.34	0.017
Mir467e	-2.09	0.0192
Mir5622	2	0.016
Mir467h	-3.42	0.0134
Mir669d-2	-2.44	0.0152
Mir466f-1	-2.15	0.0229
Mir5109	-2.4	0.0259
Mir669n	-2.89	0.0262
Mir466h	-11.33	0.0175
Mir130c	-2.15	0.0284
Mir466m	-2.06	0.0335
Mir342	2.37	0.0357
Mir342	2.37	0.0357
Mir5115	-2.16	0.037
Mir341	-4.82	0.0374
Mir7077	2.25	0.039
Mir466f-1	-2.32	0.0394
Mir467c	-6.23	0.0419
Mir297c	-2.14	0.0497
Mir692-3	-2.47	0.0004
Mir297b	-4.17	0.0007
Mir297a-3	-4.17	0.0007
Mir297a-4	-4.17	0.0007

Mir686	-2.48	0.0084
Mir684-1	-6.21	0.01
Mir669m-2	-5.94	0.026
Mir7657	-2.01	0.0147
Mir1957	-4.73	0.0479
Mir466h	-2.43	0.0269

Table S9. Effect of the low glycemic diet (LGD) with and without soluble epoxide hydrolase inhibitor (sEHI) on the expression of small nucleolar RNAs (snoRNAs) in hippocampal microvessels.

Gene Symbol	Fold Change	P-value
Gm23181	-2.32	0.0004
Gm24556	-2.56	0.0011
Gm24056	-4.77	0.0012
Gm22713	2.1	0.0021
Gm23315	2.15	0.0022
Gm14391	-2.04	0.0022
Gm24646	-4.11	0.003
Gm14308	-2.25	0.0036
Gm25506	-2.54	0.0036
Gm25128	-17.17	0.0037
Gm23644	2.08	0.0038
Gm23278	-3.33	0.0044
Gm26070	-3.03	0.0088
Gm25410	-2.8	0.0106
Gm25401	-2.11	0.0157
Gm22205	-23.17	0.0159
Gm26202	-2.69	0.0204
Gm23434	2.71	0.021
Gm24192	-2.05	0.0243
Gm24144	-2.31	0.0256
Gm24927	-4.24	0.0257
Gm23072	-2	0.026
Gm23445	2.25	0.0262
Gm28020	-2.36	0.0281
Gm23716	-2.23	0.033
Snord116	-24.74	0.0331
Gm25896	-3.25	0.0364
Gm24598	-5.83	0.0382
Gm26097	-6.88	0.0426
Gm25513	2.08	0.043
Gm23792	2.05	0.0472
Gm24535	-4.1	0.0483

Table S10. Effect of the low glycemic diet (LGD) with and without soluble epoxide hydrolase inhibitor (sEHI) on the expression of long noncoding RNAs (lncRNAs) in hippocampal microvessels.

Gene Symbol	Fold Change	P-value
Gm10115	-3.46	0.0485
Gm13632	-3.29	0.0108
Gm14041	2.02	0.0171
Gm11008	-6.51	0.0054
Gm27243	-2.96	0.0042
Gm12480	-2.37	0.0032
4632427E13Rik	-2.33	0.0204
Gm26978	2.05	0.013
Gm15656	2.21	0.017
Gm11640	-6.3	0.0377
Gm3625	-7.25	0.0059
Rian	-2.21	0.0207
Gm16243	-6.08	0.0489
Gm2310	-5.21	0.0034
Rab26os	-3.21	0.0338
Xist	-2.58	0.0424
Gm14929	-6.41	0.0109
Gm15298	-4.06	0.0156

Table S11. Low and high glycemic diets composition.

**Component** (g/kg)	LGD (Teklad Envigo TD.08485)	HGD (Teklad Envigo TD.05230)
Casein	195	195
DL-Methionine	3	3
Sucrose	120	341
Corn Starch	432.99	211.99
Maltodextrin	100	100
Anhydrous Milkfat	37.2	37.2
Soybean Oil	12.8	12.8
Cellulose	50	50
Mineral Mix, (AIN-76 170915)	35	35
Calcium Carbonate	4	4
Vitamin Mix (Teklad 40060)	10	10
Ethoxyquin (antioxidant)	0.01	0.01
